# Systemic Investigation of Promoter-wide Methylome and Genome Variations in Gout

**DOI:** 10.3390/ijms21134702

**Published:** 2020-07-01

**Authors:** Chia-Chun Tseng, Man Chun Wong, Wei-Ting Liao, Chung-Jen Chen, Su-Chen Lee, Jeng-Hsien Yen, Shun-Jen Chang

**Affiliations:** 1 Graduate Institute of Clinical Medicine, College of Medicine, Kaohsiung Medical University, Kaohsiung 80708, Taiwan; 990331kmuh@gmail.com (C.-C.T.); jehsye@cc.kmu.edu.tw (J.-H.Y.); 2 Division of Rheumatology, Department of Internal Medicine, Kaohsiung Medical University Hospital, Kaohsiung 80756, Taiwan; 3 Department of Biotechnology, College of Life Science, Kaohsiung Medical University, Kaohsiung 80708, Taiwan; harry556123@gmail.com; 4 Department of Medical Research, Kaohsiung Medical University Hospital, Kaohsiung 80756, Taiwan; 5 Department of Internal Medicine, Kaohsiung Municipal Ta-Tung Hospital, Kaohsiung 80145, Taiwan; chungjencgmh@gmail.com; 6 Laboratory Diagnosis of Medicine, College of Medicine, Kaohsiung Medical University, Kaohsiung 80708, Taiwan; sclee@kmu.edu.tw; 7 Institute of Biomedical Sciences, National Sun Yat-Sen University, Kaohsiung 80424, Taiwan; 8 Department of Biological Science and Technology, National Chiao-Tung University, Hsinchu 30010, Taiwan; 9 Department of Kinesiology, Health and Leisure Studies, National University of Kaohsiung, Kaohsiung 81148, Taiwan

**Keywords:** gout, inflammation, methylation, interleukin-1β

## Abstract

Current knowledge of gout centers on hyperuricemia. Relatively little is known regarding the pathogenesis of gouty inflammation. To investigate the epigenetic background of gouty inflammation independent of hyperuricemia and its relationship to genetics, 69 gout patients and 1455 non-gout controls were included. Promoter-wide methylation was profiled with EPIC array. Whole-genome sequencing data were included for genetic and methylation quantitative trait loci (meQTL) analyses and causal inference tests. Identified loci were subjected to co-methylation analysis and functional localization with DNase hypersensitivity and histone marks analysis. An expression database was queried to clarify biologic functions of identified loci. A transcription factor dataset was integrated to identify transcription factors coordinating respective expression. In total, seven CpG loci involved in interleukin-1β production survived genetic/meQTL analyses, or causal inference tests. None had a significant relationship with various metabolic traits. Additional analysis suggested gouty inflammation, instead of hyperuricemia, provides the link between these CpG sites and gout. Six (*PGGT1B*, *INSIG1*, *ANGPTL2*, *JNK1*, *UBAP1*, and *RAPTOR*) were novel genes in the field of gout. One (*CNTN5*) was previously associated with gouty inflammation. Transcription factor mapping identified several potential transcription factors implicated in the link between differential methylation, interleukin-1β production, and gouty inflammation. In conclusion, this study revealed several novel genes specific to gouty inflammation and provided enhanced insight into the biological basis of gouty inflammation.

## 1. Introduction

Gout is the most common inflammatory arthritis with an increasing prevalence worldwide and is associated with numerous comorbidities, such as increased body mass index, elevated blood glucose, and hypercholesterolemia [1,2]. Despite its increasing health and economic burdens, gout remains a poorly controlled disease state, and current gout therapy is complicated with increased cardiovascular risk [3]. Improved understanding of gout and therapeutic advances are urgently needed.

Studies suggest that gout develops in two steps: hyperuricemia-driven monosodium urate crystal deposition and crystal-induced gouty inflammation [3,4,5,6]. In the first step of hyperuricemia, the serum uric acid elevates, creating a hyperuricemic status that promotes crystal deposition. In the second step of gouty inflammation, monosodium urate crystals induce inflammation that is experienced as a gout attack [3,4,5,6]. Several studies suggest that these two steps involve distinct pathogenesis. First, clinical observations indicate that not all hyperuricemia cases develop gout [3]. Next, past genetic studies that quantify the relative contribution of genetic and environmental factors on phenotypic variance show that individual variability in gout has a negligible contribution from genetic factors [7]. In contrast, individual differences in hyperuricemia are significantly influenced by genetic factors [7]. Moreover, loci associated with the second step of gouty inflammation constitute a distinct group different from urate metabolism genes [6]. Thus, there are some specific pathogeneses involved in the second step of gouty inflammation.

Numerous studies have contributed to our understanding and therapeutic advances in gout. However, compared with hyperuricemia, our understanding of gouty inflammation is rather limited. Gout susceptibility loci identified in past studies are dominated by proteins involved in urate metabolism, such as *ABCG2*, *SLC2A9*, SLC22A12, and *SLC17A1* [3]. Furthermore, current gout medications mainly target hyperuricemia [3]. Regarding the steps of gouty inflammation, interleukin-1β (IL-1β) is the most well-established cytokine, with augmented IL-1β contributing to gouty inflammation [3].

Aberrant DNA methylation has been implicated in inflammatory diseases [8]. DNA methylation is a common epigenetic mechanism used by cells to modulate a gene. Hypomethylated promoter DNA is associated with active transcription, whereas hypermethylated promoter DNA leads to decreased transcription [9]. DNA methylation has been suggested to explain how the environment interacts with the host to facilitate disease development and acts as potential mechanisms linking environmental exposures to risk of diseases. Nonetheless, whether DNA methylation participates in gouty inflammation and its relationship with genetics are not completely understood.

Taking into account all of these considerations, we conducted a promoter-wide methylation study of gout and explored the relationship between methylation changes and genetics. This study presents the most comprehensive genetic and methylation profiling of gout and may be relevant for other diseases implicating genetics and epigenetics.

## 2. Results

A total of 69 patients with gout and 1455 non-gout controls who had concurrent methylation and whole-genome sequencing data were included for methylation analyses and genetic/meQTL analyses. Among those with gout, most were males (86.96%; Table S1). The subjects with gout were older (mean ± standard deviation, 52.58 ± 10.98 years vs 49.16 ± 11.15 years, *p* = 0.0128) and had a higher concentration of uric acid (7.13 ± 1.96 mg/dL vs 5.53 ± 1.39 mg/dL, *p* < 0.0001), higher glycosylated hemoglobin (HbA_1c_; 5.96% ± 0.78% vs 5.71% ± 0.73%, *p* = 0.0063), and higher body mass index (26.05 ± 3.99 vs 24.26 ± 3.57, *p* < 0.0001) (Table S1). Previous studies also demonstrated similar associations between sex, age, body mass index, blood sugar, and gout [1,2].

**Figure 1:**
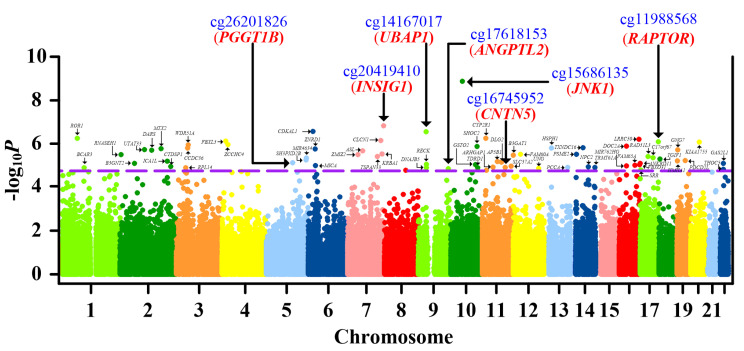
Manhattan plot of the promoter-wide methylation association in gout. X-axis shows chromosomal positions. Y-axis shows minus log_10_*P* of differential methylation tests for probed CpG sites. The dashed line indicates the false discovery rate significance threshold of 0.05. The 66 CpG sites passing multiple corrections are labeled with corresponding gene names. CpGs retained in the final analysis (cg26201826, cg20419410, cg17618153, cg15686135, cg14167017, cg11988568, and cg16745952) and corresponding genes (*PGGT1B*, *INSIG1*, *ANGPTL2*, *JNK1*, *UBAP1*, *RAPTOR*, and *CNTN5*) are highlighted with blue and red, respectively. The associations between CpG methylation and gout are calculated with multiple regression, correcting for sex, age, smoking history (total pack-years), smoking status, alcohol consumption, and blood cell subsets.

After identifying CpG located in promoters (including TSS1500, TSS200, and 5′UTR; see methods), we found 66 significant loci with a false discovery rate < 0.05 (Figure 1, Table 1, Table S2) after correcting for sex, age, smoking history (total pack-years), smoking status, alcohol consumption, and cell subsets. When we analyzed protein–protein interaction of genes mapped by these 66 significant loci (Figure S1, Step 2a), several hub genes with corresponding actions on IL-1β were highlighted (Figure S2). This was compatible with the role of IL-1β in driving gouty inflammation [3]. Thus, we conducted a literature review to identify CpG sites located in genes that regulated IL-1β or were involved in gouty inflammation (Figure S1, Step 2b). Nine CpG sites located in IL-1β-regulating genes or genes implicated in gouty inflammation were identified (Table 1) [6,10,11,12,13,14,15,16,17,18,19,20,21,22].

As gout was associated with increased body mass index, elevated blood sugar, and hypercholesterolemia [1,2], we examined the specificity of these nine CpG methylation sites to gout (Figure S1, Step 2c). When we explored the relationship of CpG site methylation with these metabolic phenotypes, none of these nine CpG sites displayed epigenetic associations with levels of body mass index (Figure S4A), HbA_1c_ (Figure S4B), and total cholesterol (Figure S4C). This evidence collectively demonstrated the specificity of these nine CpG methylation sites to gout.

**Figure 2:**
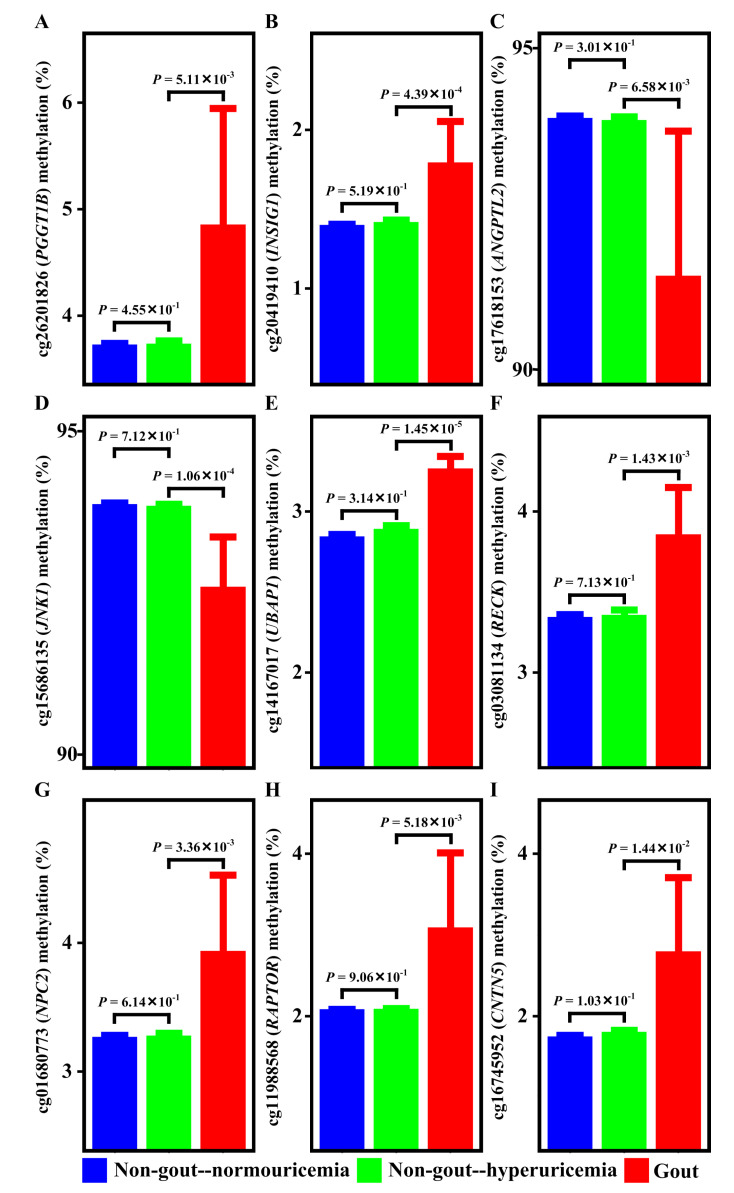
Methylation of *PGGT1B*, *INSIG1*, *ANGPTL2*, *JNK1*, *UBAP1*, *RECK*, *NPC2*, *RAPTOR*, and *CNTN5* in normouricemia, hyperuricemia, and gout. Methylation levels of *PGGT1B* (**A**), *INSIG1* (**B**), *ANGPTL2* (**C**), *JNK1* (**D**), *UBAP1* (**E**), *RECK* (**F**), *NPC2* (**G**), *RAPTOR* (**H**), and *CNTN5* (**I**) are similar between normouricemia and hyperuricemia patients. However, methylation levels of *PGGT1B*, *INSIG1*, *ANGPTL2*, *JNK1*, *UBAP1*, *RECK*, *NPC2*, *RAPTOR*, and *CNTN5* are different between hyperuricemia and gout. The methylation differences between groups are estimated with linear regression, correcting for sex, age, smoking history (total pack-years), smoking status, alcohol consumption, and blood cell subsets.

Gout progresses through two steps: hyperuricemia and gouty inflammation [3,4,5,6]. To clarify whether these CpG methylation sites are associated with gout through hyperuricemia or gouty inflammation, we examined the methylation of CpG sites in normouricemia, hyperuricemia, and gout (Figure S1, Step 2d). As shown in Figure 2, as patients transited from normouricemia to hyperuricemia, the methylation of *PGGT1B* (Figure 2A), *INSIG1* (Figure 2B), *ANGPTL2* (Figure 2C), *JNK1* (Figure 2D), *UBAP1* (Figure 2E), *RECK* (Figure 2F), *NPC2* (Figure 2G), *RAPTOR* (Figure 2H), and *CNTN5* (Figure 2I) remained the same. However, when patients transited from hyperuricemia to gout, methylation of *PGGT1B*, *INSIG1*, *ANGPTL2*, *JNK1*, *UBAP1*, *RECK*, *NPC2*, *RAPTOR*, and *CNTN5* changed (Figure 2A–I). Methylation alterations occurred in the transition from hyperuricemia to gout. These suggested that epigenetic associations of *PGGT1B*, *INSIG1*, *ANGPTL2*, *JNK1*, *UBAP1*, *RECK*, *NPC2*, *RAPTOR*, and *CNTN5* with gout came from the gouty inflammation step rather than the hyperuricemia step. This was further supported by a literature review demonstrating no overlap between these nine loci and previously identified uric acid-associated loci (Table S3; Figure S1, Step 2e).

### 2.1. Relationship between PGGT1B, INSIG1, ANGPTL2, JNK1, UBAP1, RAPTOR, and CNTN5 Methylation and Gout Not Confounded by Genetic Variants

Previous studies found a local correlation between genetic variants and DNA methylation levels (meQTL) [23,24]. To exclude genetic determinants confounding the observed epigenetic association between CpG methylation and gout, we first conducted genetic and meQTL analyses to identify variants that were associated with CpG methylation and gout (Figure S1, Step 3a). For variants simultaneously associated with methylation and gout, the relationship between variant and gout was possibly methylation mediated (Figure S3). Therefore, we applied causal inference tests [23] that explored relationships between variants, methylation, and gout among genetic variants associated with methylation and gout simultaneously. Causal inference tests explored whether genetic variations caused gout through regulating methylation, in other words, to assess the potential regulatory chain of causal genetic variant–mediator (CpG methylation)–outcome (gout) (Figure S3). If the results of the causal inference test (CIT) were significant (*p* < 0.05), then CpG methylation mediated the relationship between variant and gout. Otherwise, the variant confounded the associations between methylation and gout (Figure S1, Step 3b). For details, please refer to the supplementary methods.

**Figure 3:**
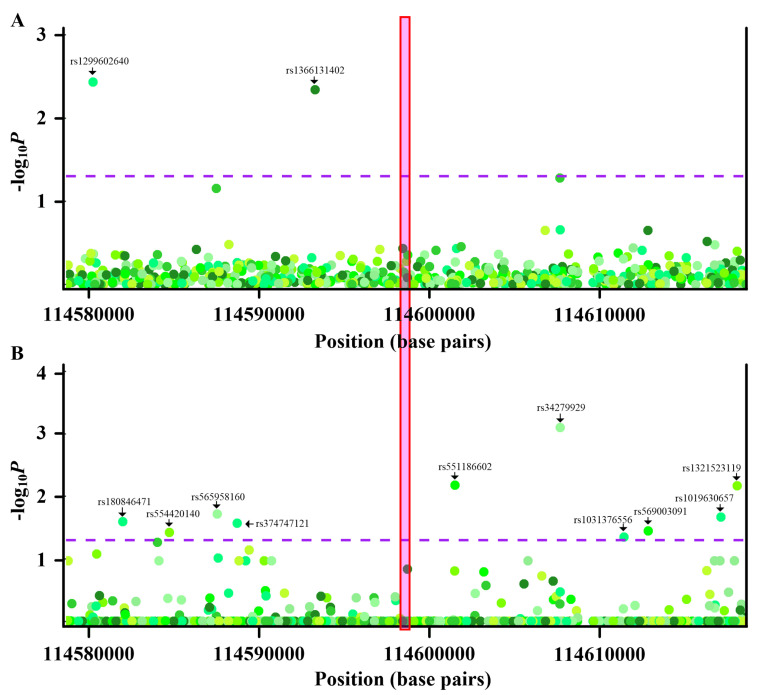
Genetic and methyl-quantitative trait locus (meQTL) analysis of cg26201826 (*PGGT1B*). (**A**) Regional association plots of nearby variants with methylation levels of cg26201826. X-axis represents positions on the respective chromosome. Y-axis represents negative log_10_*P* of associations between variants and cg26201826 methylation. The variants with *p* values less than the threshold are labeled with corresponding rs numbers. The associations between variants and CpG methylation are calculated with multiple regression, correcting for sex, age, smoking history (total pack-years), smoking status, alcohol consumption, and blood cell subsets. (**B**) Regional association plots of nearby variants with gout. X-axis represents positions on the respective chromosome. Y-axis represents negative log_10_*P* of associations between variants and gout. The variants with *p* values less than threshold are labeled with corresponding rs numbers. Every point is one variant colored with a respective hue, with different colors implying different variants. The dashed purple lines indicate the significance threshold (*p* = 0.05), and the red box highlights the location of cg26201826. The associations between variants and gout are calculated with multiple regression, correcting for sex, age, smoking history (total pack-years), smoking status, alcohol consumption, and blood cell subsets.

As shown in Figure 3, although some nearby variants were associated with *PGGT1B* methylation (Figure 3A) or gout (Figure 3B), no variants were concurrently associated with *PGGT1B* methylation and gout. Thus, a common variant did not underlie the observed epigenetic associations between *PGGT1B* methylation and gout. Similar conclusions were obtained regarding *INSIG1*, *ANGPTL2*, *JNK1*, and *CNTN5* (Figures S5–S7 and S10). Two variants (rs1189081296 and rs962251804) were simultaneously associated with *UBAP1* methylation and gout (Figure S8A,B). Causal inference testes showed that *UBAP1* methylation mediated relationships between variants (rs1189081296 and rs962251804) and gout (Figure S8C). Likewise, while rs754012543 was associated with *RAPTOR* methylation and gout (Figure S9A,B), a causal inference test revealed that *RAPTOR* methylation was the mediator between rs754012543 and gout (Figure S9C). Regarding *RECK* and *NPC2*, rs186201319 and rs539604468 confounded the relationship between *RECK* methylation and gout and between *NPC2* methylation and gout, respectively (Table 1, Table S4). Taken together, these results suggested that relationships between *PGGT1B*, *INSIG1*, *ANGPTL2*, *JNK1*, *UBAP1, RAPTOR,* and *CNTN5* methylation and gout were not confounded by genetic mediators. Therefore, these seven CpG sites located in seven genes (*PGGT1B*, *INSIG1*, *ANGPTL2*, *JNK1*, *UBAP1, RAPTOR,* and *CNTN5*) were reserved for the following analysis.

### 2.2. Less Evidence of Epigenetic Association with Gout from Co-methylated Cpgs

Given that the degree to which the CpG methylation state is spatially correlated [24,25,26], and clusters of co-methylated CpG sites may be of biological relevance [27], we tested these possibilities in gout (Figure S1, Step 4a).

**Figure 4:**
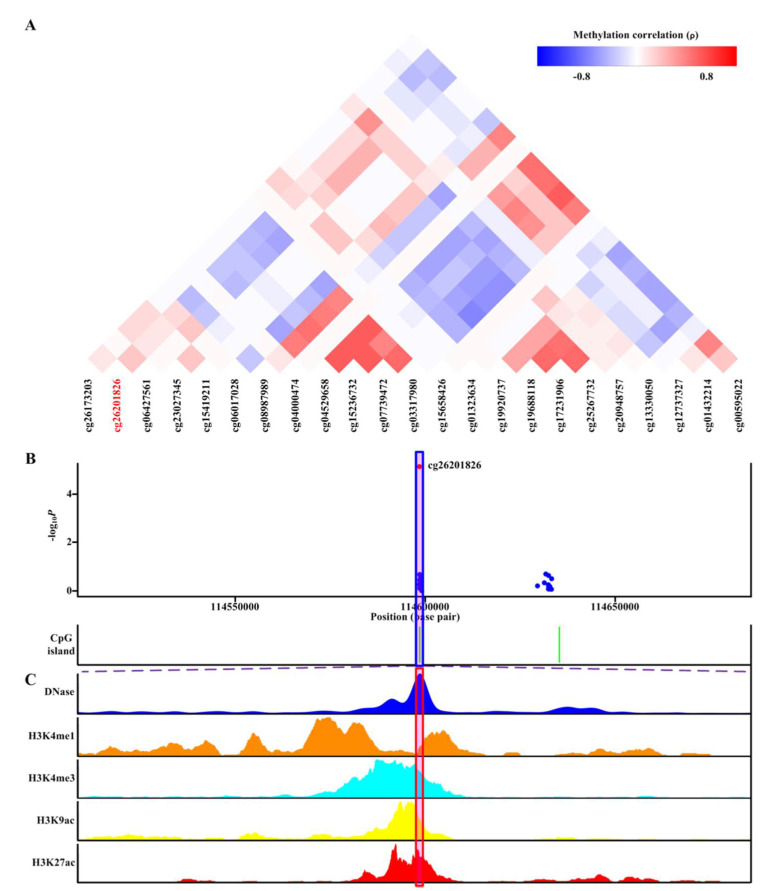
Co-methylation analysis and functional localization of cg26201826 (*PGGT1B*). (**A**) Patterns of co-methylation at the CpG sites surrounding cg26201826. (**B**) Regional association results along with position of nearby CpG islands (green). cg26201826 (highlighted in shaded box) is located in CpG islands. (**C**) Functional annotation of cg26201826. DNase hypersensitive sites derived by DNase-seq (DNase Track) and histone marks surrounding cg26201826 (H3K4me1, H3K4me3, H3K9ac, and H3K27ac tracks) in monocytes are shown. DNase hypersensitivity, H3K4me1, H3K4me3, H3K9ac, and H3K27ac histone marks are associated with active regulatory elements.

Of the seven differentially methylated CpG sites located in seven genes (*PGGT1B*, *INSIG1*, *ANGPTL2*, *JNK1*, *UBAP1, RAPTOR,* and *CNTN5*) passing genetic/meQTL analyses or causal inference tests, corresponding CpG probes located within the nearby promoter and 5′UTR available in EPIC BeadChip array were shown (Figure 4A, Figures S11A–S16A). As shown in Figure 4A, none of the nearby CpG sites demonstrated evidence of co-methylation with cg26201826 located in *PGGT1B* (*p* ≥ 0.8) [28]. Furthermore, when we analyzed the association between methylation of nearby CpG sites and gout, none of nearby CpG probes showed strength of epigenetic associations with gout analogous to cg26201826 (Figure 4B). Similar phenomena occurred in cg20419410 (*INSIG1*; Figure S11A,B), cg17618153 (*ANGPTL2*; Figure S12A,B), cg15686135 (*JNK1*; Figure S13A,B), cg14167017 (*UBAP1*; Figure S14A,B), cg11988568 (*RAPTOR*; Figure S15A,B), and cg16745952 (*CNTN5*; Figure S16A,B).

### 2.3. Functional Localization of Differentially Methylated CpG Loci in Regulatory Elements

To assess whether the seven differentially methylated CpG sites passing genetic and meQTL analyses or causal inference tests had functional potential, we utilized the epigenetic data around each differentially methylated CpG in monocytes using the WashU epigenome browser (Figure S1, Step 4b). We chose DNase, H3K4me1, H3K4me3, H3K9ac, and H3K27ac as annotation marks because all of them were associated with active regulatory regions (see supplementary methods).

cg26201826 (*PGGT1B*) was located in DNase hypersensitivity sites of monocytes derived by DNase-seq (Figure 4C). Furthermore, cg26201826 (*PGGT1B*) was located in a transcriptional regulatory region containing multiple histone marks characteristic of active regulatory elements (H3K4me1, H3K4me3, H3K9ac, and H3K27ac) in monocytes (Figure 4C). Together, these features suggested regulatory potentials of cg26201826 (*PGGT1B*) in monocytes. Similar results for the other six differentially methylated CpG sites (cg20419410 (*INSIG1*), cg17618153 (*ANGPTL2*), cg15686135 (*JNK1*), cg14167017 (*UBAP1*), cg11988568 (*RAPTOR*), and cg16745952 (*CNTN5*)) were observed, with each overlapping with DNase hypersensitivity sites and various histone marks of active regulatory elements (H3K4me1, H3K4me3, H3K9ac, and H3K27ac) in monocytes (Figures S11C–S16C). Altogether, this evidence supported that these seven differentially methylated CpG sites located in seven genes (*PGGT1B*, *INSIG1*, *ANGPTL2*, *JNK1*, *UBAP1, RAPTOR,* and *CNTN5*) all displayed regulatory potential in monocytes.

### 2.4. Transcription Factor Mapping of Differentially Methylated CpG Sites

Because differential methylation mediated transcription dysregulation through altered transcription factor binding [29], we utilized MoLoTool and ReMap to identify potential involved transcription factors [30,31]. Several transcription factors that bound cg26201826 (*PGGT1B*), cg20419410 (*INSIG1*), cg17618153 (*ANGPTL2*), cg15686135 (*JNK1*), cg14167017 (*UBAP1*), cg11988568 (*RAPTOR*), and cg16745952 (*CNTN5*) were identified (Table S5–S15, Figure S17). No transcription factors that bound cg17618153 (*ANGPTL2*), cg15686135 (*JNK1*), and cg16745952 (*CNTN5*) were identified by ReMap (Figure S17). When we examined methylation of these transcription factors, some displayed methylation changes at nominal significance (*p* < 0.05) but none survived multiple corrections (all had a false discovery rate >0.05; Table S16).

## 3. Discussion

In gout patients, seven aberrant DNA methylation sites that were mapped to seven genes (*PGGT1B, INSIG1, ANGPTL2, JNK1, UBAP1, RAPTOR,* and *CNTN5*) and survived genetic and meQTL analyses or causal inference tests were discovered (Table 1). The seven genes were not uric acid-associated genes (Table S3). Additionally, methylation of the seven CpG sites remained the same when patients transited from normouricemia to hyperuricemia but changed during the transition from hyperuricemia to gout (Figure 2). Moreover, methylation of the seven CpG sites had no relationship with gout comorbidities (Figure S4). These observations suggested their specific associations with gout arose from gouty inflammation instead of hyperuricemia. Moreover, these aberrant DNA methylation sites were located in an open chromatin structure and overlapped with active regulatory region histone marks (Figure 4C, Figures S11C–S16C), supporting their potential in the regulation of gene expression. Transcription factor mapping also identified several potential transcription factors mediating their relationship with gout.

Utilizing the cell subset frequency estimated from methylation, we observed a comparable distribution of cellular subsets in the blood of gout patients similar to non-gout controls (Figure S18). In light of cell subsets changes correlating with disease activity [32], one possible reason for the apparent lack of a difference was the absence of arthritis during this study’s sample collection. This requires further investigation in the future.

In this study, several variants located in *UBAP1* and *RAPTOR* were associated with gout, and causal inference tests revealed that the effects of genotypes on gout appeared to be mediated by CpG methylation changes (Table 1, Figures S8 and S9). These two genes have not been previously implicated in gout through genome-wide association studies. A similar phenomenon occurred in past research, with novel genes not previously implicated in disease identified through causal inference tests [23].

The seven differentially methylated CpG sites surviving genetic and meQTL analyses or causal inference tests exhibited absolute methylation difference between 0.38% and 1.38%, well within the range of 0.1–3.7% [33] and 0.12–11.6% [34] observed in past methylation studies. Prior reports indicated that trait-associated methylation changes were predominantly of small magnitude [33,34] and tended to be subtle and long-lasting, with stronger but short-lived gene expression alterations [35]. Accumulating evidence further suggested that subtle methylation changes as little as 0.1% may be translated to gene expression changes [36]. These observations collectively supported the biological relevance of methylation alterations identified in this study.

**Figure 5:**
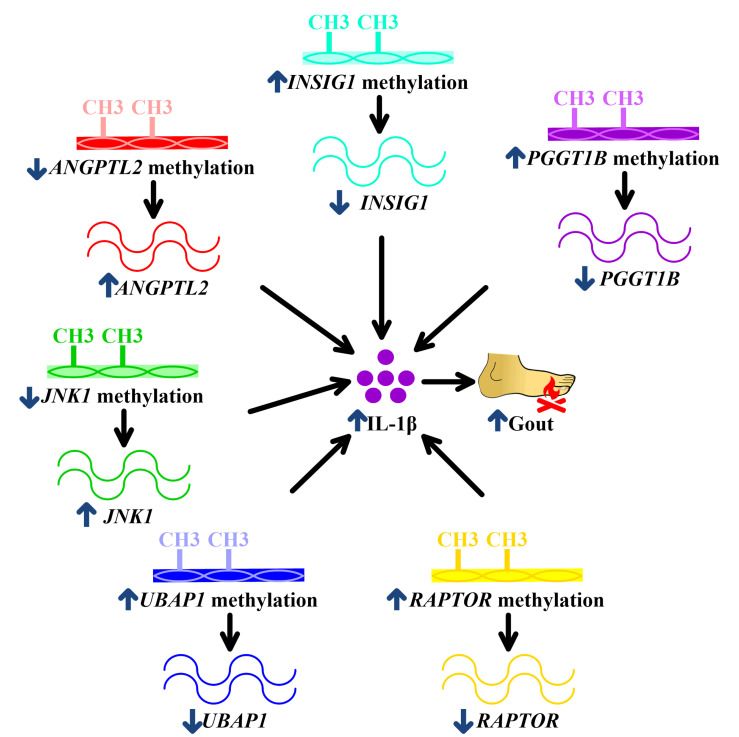
Potential mechanisms underlying associations of *PGGT1B*, *INSIG1*, *ANGPTL2*, *JNK1*, *UBAP*1, and *RAPTOR* methylation with gouty inflammation. *PGGT1B* hypermethylation in gout decreases *PGGT1B,* *INSIG1* hypermethylation in gout reduces *INSIG1*, *ANGPTL2* hypomethylation enhances *ANGPTL2*, hypomethylated *JNK1* increases *JNK1*, *UBAP*1 hypermethylation downregulates *UBAP1*, and hypermethylated *RAPTOR* represses *RAPTOR*. All of these culminate in augmented IL-1β production, facilitating gouty inflammation. The blue arrows mean the change of methylation or expression or development of gout. The black arrows mean the consequence of methylation or expression alterations.

The seven aberrant DNA methylation sites were mapped to seven genes (*PGGT1B*, *INSIG1*, *ANGPTL2*, *JNK1*, *UBAP1*, *RAPTOR,* and *CNTN5*) that have not been elucidated in the field of gout except *CNTN5*. *PGGT1B* suppresses IL-1β release in macrophages [10]. In the context of hypermethylation-decreased transcription [9], hypermethylated *PGGT1B* might result in reduced *PGGT1B* transcription, subsequently augmenting IL-1β production in macrophages and facilitating gout (Table 1, Figure 5). Given that *INSIG1* ameliorates IL-1β release in macrophages [11], *INSIG1* hypermethylation results in attenuated *INSIG1* levels, leading to exaggerated IL-1β expression, the central driver in gouty inflammation (Table 1, Figure 5) [37]. *ANGPTL2* stimulates IL-1β expression in macrophages [12]. In association with hypomethylation-increased transcription [9], hypomethylated *ANGPTL2* thus could increase *ANGPTL2* expression and, following IL-1β expression in macrophages, perpetuate gouty inflammation (Table 1, Figure 5). *JNK1* is also known as *MAPK8* [13]. *JNK1* is required for IL-1β production in macrophages [14]. Consequently, hypomethylated *JNK1* might contribute to increased *JNK1,* accentuating IL-1β production in macrophages and presenting as gout clinically (Table 1, Figure 5). In the matter of *UBAP1*, *UBAP1* is expressed in macrophages and downregulates IL-1β (Table S6 of [15,16]). Therefore, hypermethylation of *UBAP1* potentially reduces *UBAP1* transcription and intensifies IL-1β production, exacerbating gout (Table 1, Figure 5). Also known as *RPTOR, RAPTOR* is expressed in macrophages and has been demonstrated to abrogate IL-1β production [21,22]. Consequently, hypermethylated *RAPTOR* lowers *RAPTOR* transcription, increases IL-1β levels, and promotes gouty inflammation (Table 1, Figure 5). Regarding *CNTN5*, little was known about its biological function. However, it was reported to participate in gouty inflammation and treatment response of rheumatoid arthritis and Crohn’s disease, two diseases involving macrophages [6,38,39,40,41]. Interestingly, *CNTN5* was downregulated when monocytes differentiated into macrophages (Figure S19), the key contributor to gouty inflammation [3,42]. These findings collectively supported the potential of *CNTN5* in regulating gouty inflammation, although the mechanistic link between *CNTN5* and gouty inflammation needs to be explored in the future.

Transcription factors mapping identified several transcription factors, some of which (*ATF2*, *BRD4*, *CEBPA, CEBPB, CLOCK*, *ELK4*, *FOS*, *KLF6*, *KLF9*, *KMT2A*(*MLL1*), *MED1*, *NR1H4*(*FXR*), *RELA*(*p65*), *RUNX1*, *RXR*, *SP1*, *SPI1*(*PU.1*), *STAT1*, and *VDR*) were reported in the literature to regulate IL-1β (Figure S17). Furthermore, *BRD4* was implicated in gouty inflammation in the past [43]. The involved signaling pathways of these transcription factors in gouty inflammation deserve further studies.

Given the roles of *PGGT1B*, *INSIG1*, *ANGPTL2*, *JNK1*, *UBAP1*, *RAPTOR*, and *CNTN5* in regulating IL-1β or gouty inflammation and differential methylation of these genes in gout, the next important topic is the consequence of manipulating the respective signaling pathways. Interestingly, some studies explored the relationships between molecules, *INSIG1* and *ANGPTL2,* and gout. Coenzyme Q10 and epigallocatechin gallate (EGCG), which increased *INSIG1*, ameliorated gouty inflammation [44,45,46,47]. However, leptin receptor deficiency reduced *ANGPTL2,* while leptin promoted gouty inflammation [48,49]. Taking into account these observations, the results of this study can provide a starting point in the search for novel therapeutic targets of gout.

If common genetic variation did not contribute to the epigenetic associations of *PGGT1B, INSIG1*, *ANGPTL2*, *JNK1*, and *CNTN5* with gout, it would be interesting to clarify mechanisms underlying the differential methylation of these CpG sites in gout. Prior research suggested that risk of gout was lowered by consumption of coffee, which inhibited DNA methylation [50,51]. Similarly, omega-3 fatty acid, which decreased DNA methylation [52], displayed negative association with gout [53]. However, legumes, which increased methylation, reduced gout [54,55]. Therefore, it was possible that some environment factors modulated DNA methylation and subsequently impacted gout. The exact etiologies contributing to differential methylation of *PGGT1B*, *INSIG1*, *ANGPTL2*, *JNK1*, and *CNTN5* still need to be elucidated.

The major strength of this study compared with previous DNA methylation studies was that potential confounding factors, including sex, age, smoking history (total pack-years), smoking status, alcohol consumption, and cell subsets [56,57,58], were all considered. Past DNA methylation studies did not adjust adequately for these important confounders [59], which limited the validity of the conclusions. In this study, EPIC BeadChip array, instead of a sequencing platform, was utilized to measure methylation of leukocytes. EPIC BeadChip array has been validated as a very reliable genomic platform for determining DNA methylation patterns in the human genome [60]. Although experiments with next-generation sequencing approaches might offer more comprehensive coverage of genome-wide CpG sites [61], the more expensive cost, larger DNA requirement, significantly increased sample processing time [61], and the amount of sequencing required to achieve precision similar to that obtained in a methylation array [62] all have made next-generation sequencing approaches difficult to implement for most research budgets. Currently, most epigenome-wide studies (~99%) have been exclusively performed on methylation arrays [62].

Some potential drawbacks of this study could be improved in future research. Even though promoter methylation influenced gene expression [9], we did not evaluate the correlation between *PGGT1B*, *INSIG1*, *ANGPTL2*, *JNK1*, *UBAP1*, *RAPTOR*, and *CNTN5* methylation and respective gene expressions. Functional correlation between methylation and expression of these genes may provide additional information to support our hypothesis. Additionally, future replication studies using a different measuring technology to validate the methylation of these novel CpG sites could strengthen the results presented in this work. Moreover, measuring transcription factors identified in this study (Figure S17) and chromatin immunoprecipitation (ChIP) for these transcription factors would provide more clues about the mechanistic link between *PGGT1B*, *INSIG1*, *ANGPTL2*, *JNK1*, *UBAP1*, *RAPTOR*, and *CNTN5* methylation and gout. Finally, in this research, the blood leukocytes were chosen to study methylation due to the well-established roles of leukocytes in gout [63] and accessibility of peripheral leukocytes, which was also the main tissue type utilized in most methylation studies [33,64]. However, some evidence showed that chondrocytes and fibroblast-like synoviocytes also produced inflammatory mediators and might contribute to gouty inflammation [65,66]. Future studies utilizing alternative tissues to address these possibilities are required.

In conclusion, this study provided evidence of aberrant methylation changes of *PGGT1B*, *INSIG1*, *ANGPTL2*, *JNK1*, *UBAP1*, *RAPTOR*, and *CNTN5* in gout. These genes were associated with gout through gouty inflammation rather than hyperuricemia. Their roles in gouty inflammation should be further explored in the future. These integrative epigenomic and genomic results will enhance our understanding of gout pathophysiology, potentially bridging the gap between predisposition to gout and gout pathogenesis. Furthermore, systemic investigation of methylation profiles in different diseases employing a similar analysis pipeline may reveal the extent to which genetics and epigenetics play a causal role in diseases.

## 4. Materials and Methods

The study utilized samples from Taiwan Biobank, a biomedical research database for the development of precision medicine [67]. The analysis of this study included the following: Step 1, promoter-wide methylation profiling; Step 2, identifying CpG specific to gouty inflammation; Step 3, genetic and methylation quantitative trait loci (meQTL) analysis/causal inference test; Step 4, co-methylation analysis/functional localization; and Step 5, transcription factor mapping (Figure S1), as described in the supplementary methods. The study protocol was approved by the Institutional Review Board (TSMHIRB 17-122-B).

## Figures and Tables

**Table 1:** Significant CpG sites that were mapped to genes implicated in IL-1β production or gouty inflammation.

CpG Site	Δβ ^a^	*P*	Chr	Position ^b^	Gene ^c^	Genomic Features	Genetic and meQTL ^d^	CIT ^e^	Reference ^f^
Implicated in IL-1β Production in Macrophages										
cg26201826	1.15%	7.27 × 10^−6^	5	114598579	*PGGT1B*	TSS200	Y	-	↓[10]
cg20419410	0.39%	1.48 × 10^−7^	7	155089803	*INSIG1*	5′UTR	Y	-	↓[11]
cg17618153	−1.38%	1.36 × 10^−5^	9	129874991	*ANGPTL2*	5′UTR	Y	-	↑[12]
cg15686135	−1.24%	1.32 × 10^−9^	10	49542423	*JNK1* (*MAPK8*) [13]	5′UTR	Y	-	↑[14]
Expressed in Macrophages and Implicated in IL-1β Production										
cg14167017	0.38%	2.78 × 10^−7^	9	34178925	*UBAP1*	TSS200	N	Y	↓Table S6 of [15,16]
cg03081134	0.49%	8.77 × 10^−6^	9	36036806	*RECK*	TSS200	N	N	↓[17,18]
cg01680773	0.67%	1.13 × 10^−5^	14	74960124	*NPC2*	TSS200	N	N	↓[19,20]
cg11988568	1.04%	7.84 × 10^−7^	17	78518917	*RAPTOR* (*RPTOR*) [21]	5′UTR	N	Y	↓[21,22]
Implicated in Gouty Inflammation										
cg16745952	0.96%	1.22 × 10^−5^	11	98891665	*CNTN5*	TSS200	Y	-	[6]

Chr: chromosome. ^a^ Methylation levels of gout minus methylation levels of non-gout estimated with linear regression, after adjusting for sex, age, smoking history (total pack-years), smoking status, alcohol consumption, and cell subsets. ^b^ Positions of the CpG sites in hg19. ^c^ Gene names and their alias. ^d^ Y denotes CpG sites surviving genetic and meQTL analysis (without genetic variants concomitantly associated with CpG methylation and gout). N denotes CpG sites failing genetic and meQTL analysis (with genetic variants concomitantly associated with CpG methylation and gout). ^e^ Y denotes CpG sites passing causal inference tests (CITs), N denotes CpG sites not passing CITs. ^f^ References supporting the role of mapped genes in IL-1β production/macrophage expression/gouty inflammation, and ↑ means the gene increases IL-1β production while ↓ means the gene decreases IL-1β production.

## Supplementary Materials

Supplementary materials can be found at https://www.mdpi.com/1422-0067/21/13/4702/s1.

## Abbreviations

CIT	causal inference test
ChIP	chromatin immunoprecipitation
EGCG	epigallocatechin gallate
HbA_1c_	glycosylated hemoglobin
IL-1β	interleukin-1β
meQTL	methylation quantitative trait loci
