# Supplementary material for: Systemic Investigation of Promoter-wide Methylome and Genome Variations in Gout
Source: Int J Mol Sci. 2020 Jul 1;21(13):4702. doi: 10.3390/ijms21134702 (PMC7369819; doi:10.3390/ijms21134702)
Supplement: Supplementary file 1 [file ijms-21-04702-s001.pdf]

International Journal of
Molecular Sciences

Supplementary Methods

Participants Selection

All of the samples in this study were obtained from the Taiwan Biobank (TWB), a cohort established to facilitate translational research in the biomedical field, improve public health in the Taiwanese community, and advance our knowledge of the relationships among epigenetics, the environment, and the etiology/progression of diseases [1]. This cohort was recruited from the general Taiwanese population aged 30 to 70 years and has been utilized in numerous genetic and epigenetic studies, including gout [2–4]. During recruitment, all TWB patients who provided informed consent to undergo measurement of peripheral blood leukocytes DNA methylation and sequencing of DNA were extracted. The study protocol was approved by the Institutional Review Board (TSMHIRB 17-122-B) and the TWB is governed by the Ethics and Governance Council (EGC) and the Ministry of Health and Welfare in Taiwan. All of the experiments were conducted in accordance with relevant guidelines and regulations.

Our study cohort comprised 69 patients with self-reported gout and 1455 patients who self-reported the absence of gout who had methylation array and whole genome sequencing data until October, 2018. All of the participants reported themselves as Han Chinese. Previous studies utilized a similar method of self-reported gout to conduct genetic studies [3,5–7] and suggested that self-reporting of physician-diagnosed gout had high sensitivity and precision for genetic studies of gout [8].

Bisulfite Conversion and DNA Methylation Measurement

The included participants' peripheral blood was collected into sodium citrate tubes. DNA was extracted with a Chemagic™ Prime™ instrument that was an automated chemical extraction machine that used magnetized rods to separate nucleic acids from solutions. The DNA length was measured using a Fragment Analyzer (Agilent) with the purity assessed utilizing the optical density (OD) at 260/280. Samples with an OD 260/280 ratio of 1.6-2.0 were considered to be pure and stored at -80 °C for following analysis. Obtained DNA was treated with sodium bisulfite using the EZ DNA methylation kit (Zymo Research, CA, USA) for bisulfite conversion. DNA methylation was quantified using HumanMethylationEPIC (EPIC) BeadChip (Illumina) [9]. Samples were randomized on the BeadChip to avoid batch effects. The experiment was conducted according to the manufacturer's standard protocol.

Whole Genome Sequencing

Genomic DNA was extracted and purified from peripheral blood with standard protocols. Whole genome sequencing was conducted on the Illumina Hiseq platform. We obtained an average of 8.6 Gb of mappable sequence data per individual. DNA sequence reads were mapped to the hg19 reference genome with Isaac version 01.13.10.21. The region of interests was covered at a minimum of 30×. Variant calling was conducted with Isaac Variant Caller version 2.0.17, Grouper version 1.4.2, and CNVseg version 2.2.4. Alleles were annotated with ANNOVAR version 2014Jul14. Complete assembly of the genome in the regions of interests was obtained in all of the participants.

Marker Selection

In the MethylationEPIC platforms, CpG markers were classified based on their chromosome location and the feature category gene region as per University of California (UCSC) annotation (TSS200, TSS1500, 5'UTR, first Exon, Body, 3'UTR, and intergenic). In this classification system, the TSS200 category included the region between 0 and 200 bases upstream from the transcriptional start site (TSS); the TSS1500 category contained 201 to 1500 bases upstream TSS [10]; 5'UTR included the region between the TSS and the start site (ATG); CpGs within the first exon of a gene were considered as the first Exon category; CpGs downstream from the first exon including intronic regions until the

stop codon were classified as gene body; CpGs located downstream from the stop codon until the poly A signal were considered as 3'UTR; and CpGs that were not classified in any of the previous categories were annotated as intergenic. Since contributions of environmental influences to DNA methylation peaked in the vicinity of transcription start sites [11] and binding sites of transcription factors, the readers and effectors of DNA methylation, occurred near transcription start sites [12,13], regions close to transcription start sites were more likely to be functional compared with those that lay far from transcription start sites. Past studies also confirmed a key role for the region proximal to transcription start sites in transcriptional regulation [14], and transcriptional silencing occurred when DNA region near transcription start site, including transcription start site upstream area and 5'UTR, became heavily methylated [15–17]. Hence, we focused our analysis on CpG sites located in TSS1500, TSS200, and 5'UTR that was broadly defined as promoters in past studies [18]. CpG sites of X and Y chromosome were excluded since X chromosomes underwent inactivation through methylation in females [19].

Methylation Data Processing and Analysis

Raw Idat files containing fluorescence intensity data were loaded. The intensity of methylated and unmethylated probe values was used to generate methylation β-values that were used for all of the downstream analyses. Human hg19 genomes were downloaded from the UCSC Genome Browser website (https://genome.ucsc.edu/). All of the downstream analyses were conducted using the hg19/GRCh37 human genome assembly. Minfi version 1.18.2 [20] was employed to load, annotate probes, and analyze the relationship between CpG sites and gout.

Methylation results from EPIC array were analyzed according to previously reported approaches (Figure S1, Step 1) [21]. Quality control at the probe level was conducted by computing a detection P value relative to control probes. Probes with non-significant detection ($P > 0.05$) for 5% or more of the samples were excluded (Figure S1, Step 1b). Furthermore, we removed probes annotated to sex chromosomes (Figure S1, Step 1c), non-CpG probes (Figure S1, Step 1d), probes containing single nucleotide polymorphisms (SNPs) (minor allele frequency ≥ 5%), probes with SNPs at the single base extension (minor allele frequency ≥ 5%), and probes with an SNP at the CpG site (minor allele frequency ≥ 5%) (Figure S1, Step 1e) [21]. Finally, we excluded 40,377 cross-reactive probes previously identified in the MethylationEPIC BeadChip (Figure S1, Step 1f) [21]. Data were further preprocessed using functional normalization with principal components from control probes to adjust for technical variation (Figure S1, Step 1g) [21]. Qualified probes were included in the following analyses.

To evaluate associations between methylation and gout, a linear regression model was used to identify the differentially methylated probes by testing the association of every CpG site with gout, correcting for sex, age, smoking history (total pack-years), smoking status, alcohol consumption, and blood cell subsets (Figure S1, Step 1h), similar to past approaches [22,23]. Non-smokers included those who never smoked or did not continuously smoke for six months or more. Former smokers were those who continuously smoked for a minimum of six months but were not smoking at the time that data were collected, while current smokers included those who ceaselessly smoked for six months or more and were still smoking. Alcohol consumption categories comprised non-drinkers (those who did not drink alcohol or drank <150 cc per week for six months), former drinkers (those who quitted alcohol for more than six months) and current drinkers (those whose weekly alcohol consumption for six consecutive months was at least 150 cc), according to the definition from Taiwan Biobank questionnaires [2].

The proportions of various cell types were inferred with minfi [24,25]. Minfi used informative CpG probes of past studies to estimate the proportions of T- (CD8, CD4), NK-, and B-lymphocytes, monocytes, and total granulocytes [24]. Differential methylation associations between gout and non-gout were corrected for multiple testing using a Benjamini-Hochberg method [26]. The threshold of significance levels was set at 5 % as described previously [27].

Identification of CpG Sites Specifically Associated with Gouty Inflammation

To gain insight about these differentially methylated CpG sites, we built protein-protein interaction network on the target genes mapped by differentially methylated CpG sites with NetworkAnalyst (Figure S1, Step 2a) [28] and visualized with Cytoscape [29]. NetworkAnalyst utilized machine learning and Walktrap algorithms and integrated protein-protein interaction data from IMEx Interactome database to identify important genes (hubs) that played critical roles in the biological networks [29]. NetworkAnalyst showed that many hub genes were interleukin-1β (IL-1β)-regulating genes (Figure S2). Thus we conducted a literature search to clarify biologic functions of differentially methylated genes. CpG sites mapped to genes regulating IL-1β, the key player in gouty inflammation [30], or participating in gouty inflammation in past studies were retained for the following analysis (Figure S1, Step 2b).

Additionally, gout was associated with numerous metabolic comorbidities, including increased body mass index, elevated glycated hemoglobin (HbA_{1c}), and hypercholesterolemia [31,32]. To test the specificity of identified CpG methylation in gout, we explored associations between DNA methylation and body mass index, HbA_{1c}, and total cholesterol, adjusting for sex, age, smoking history (total pack-years), smoking status, alcohol consumption, and cell proportions inferred with minfi (Figure S1, Step 2c) [24,25].

To further exclude the contribution of these CpG sites to progression from normouricemia to hyperuricemia, we investigated whether methylation was related to hyperuricemic status in non-gout participants, using sex, age, smoking history (total pack-years), smoking status, alcohol consumption, and blood cell subsets as covariates (Figure S1, Step 2d).

Comparison with Past Genome-Wide Association Studies (GWAS) Results

To compare study results with past uric acid-associated risk loci, we conducted a systematic literature search. A systematic literature search of PubMed (on October 1, 2019) identified studies addressing the relationship between genetic loci and uric acid levels. The following search terms were used: 'GWAS' and 'uric acid'. Studies were included if they were written in English and contained data on loci associated with uric acid levels. No limitation was placed on ethnicity. Review articles and case reports were excluded. Selected articles were screened for potential uric acid-associated loci. Only genome-wide significant loci ($P < 5 \times 10^{-8}$) related to uric acid levels were retained (Figure S1, Step 2e).

Genetic and Methylation Quantitative Trait loci (meQTL) Analysis and Causal Inference Test

To eliminate epigenetic associations between gout and CpG site methylation confounded by genetic factors, we conducted methyl-quantitative trait locus (meQTL) analyses and causal inference tests of variants within 20000 base pairs of CpG, similar to previous studies (Figure S1, Step 3) [33,34]. Briefly, we first retrieved variant information from UCSC dbSNP version 151 track from UCSC. Variants that were located within 20000 base pairs of CpG of interest were reserved. Genotyping results of respective variants were obtained from whole genome sequencing. Next, we evaluated the associations between genetic variants and methylation level of a CpG site, adjusting for sex, age, smoking history (total pack-years), smoking status, alcohol consumption, and cell compositions. Additionally, we also tested associations between variants and gout, adjusting for sex, age, smoking history (total pack-years), smoking status, alcohol consumption, and cell proportions, and plotted regional variant results from analysis.

For variants of concomitant associations with CpG methylation and gout, differential methylation potentially mediated genetic association between these variants and gout (Figure S3), as demonstrated in past study of rheumatoid arthritis [34]. Thus, we conducted causal inference test (Figure S1, Step 3b) [34,35]. The causal inference test involved a series of conditional probability tests to determine if the association between variant and gout was mediated by CpG methylation, in this case, by testing for the following conditions: (a) variant was associated with gout; (b) variant was associated with CpG methylation after adjusting for gout; (c) CpG methylation was associated with

gout after adjusting for variant; and (4) variant was independent of gout after adjusting for CpG methylation. These four conditions had to be met to produce a significant causal inference test that supported the causal structure of an variant-CpG methylation-gout relationship (Figure S3). If not, variants possibly confounded associations of CpG methylation with gout.

Methylation Correlation of Methylated CpG Positions

After identifying CpG sites whose methylation was associated with gouty inflammation, we applied coMET to estimate DNA methylation correlation between CpG sites (Figure S1, Step 4a) [36]. We searched for nearby CpG sites located in TSS1500, TSS200, and 5'UTR. A regional plot of the epigenetic-phenotype association results, the estimated DNA methylation correlation between CpG sites (co-methylation), and the genomic context was generated to visualize the results.

Functional Localization of Differentially Methylated CpG Sites in Monocytes

To characterize the biological relevance of differentially methylated CpG sites, the WashU epigenome browser (https://epigenomegateway.wustl.edu/) was utilized as the annotation database to inspect chromatin modifications and histone acetylation patterns of monocytes in the vicinity of CpG sites of interest [37]. We retrieved DNase footprinting patterns in the region of our association results since DNase hypersensitivity implied open chromatin structure, the usual characteristics of active regulatory elements [38]. CpG regions were also aligned against ChIP-seq data for acetylated and methylated lysine variants of histone H3 (H3K4me1, H3K4me3, H3K9ac, and H3K27ac), the histone code of active regulatory regions [39]. Locations of differentially methylated sites with respect to DNase and histone code of monocytes was visualized in WashU's epigenome browser (Figure S1, Step 4b).

Transcription Factor Mapping

Altered DNA methylation leads to transcriptional dysregulation through altered transcription factor binding [40]. To identify potential involved transcription factors, we combined MoLoTool with ReMap (Figure S1, Step 5) [41,42]. For the CpG sites surviving above analysis, we downloaded DNA sequence around each surviving CpG site from Ensembl BioMart (GRCh38.p13 assembly) and executed MoLoTool to identify transcription factors that bound surviving CpG sites (Figure S1, Step 5a) [41,43]. We utilized the recommended default threshold of *P*-value 0.0005 [41]. We also used ReMap that collected high-quality transcription factor binding regions from thousands of ChIP-seq and ChIP-exo datasets [42]. We retrieved transcription factor binding sites in monocytes/macrophages (CD14+ cells [44], macrophage, monocyte-derived macrophage (MDM), MOLM-13 [44,45], MOLM-14 [45,46], monocyte, NOMO1 [47], MV4-11 [44,48], THP-1 [44,47], and U-937 [49]) in the ReMap database and intersected transcription factor binding sites of monocytes/macrophages with surviving CpG sites (Figure S1, Step 5b). Transcription factors identified by MoLoTool and ReMap were summarized and plotted.

Gene Expression Omnibus (GEO) Data Analysis

To elucidate the potential functions of differentially methylated genes, we retrieved past expression microarray data from the GEO repository for further analysis. Briefly, GEOquery was utilized to download a corresponding SeriesMatrix file and platform annotation files via FTP from corresponding GEO datasets [50]. Expression levels of various genes were extracted from downloaded SeriesMatrix files that were inputs of differential expression analysis. Limma Bioconductor libraries were used to calculate the statistical significance of expression level changes of interested genes between different conditions [51].

References

1. Lin, J.C.; Fan, C.T.; Liao, C.C.; Chen, Y.S. Taiwan Biobank: making cross-database convergence possible in the Big Data era. *Gigascience.* **2018**, *7*, 1-4.

2. Tantoh, D.M.; Wu, M.F.; Ho, C.C.; Lung, C.C.; Lee, K.J.; Nfor, O.N.; Liaw, Y.C.; Hsu, S.Y.; Chen, P.H.; Lin, C., et al. SOX2 promoter hypermethylation in non-smoking Taiwanese adults residing in air pollution areas. *Clin. Epigenetics.* **2019**, *11*, 46.
3. Lee, M.G.; Hsu, T.C.; Chen, S.C.; Lee, Y.C.; Kuo, P.H.; Yang, J.H.; Chang, H.H.; Lee, C.C. Integrative Genome-Wide Association Studies of eQTL and GWAS Data for Gout Disease Susceptibility. *Sci. Rep.* **2019**, *9*, 4981.
4. Lin, W.Y.; Chan, C.C.; Liu, Y.L.; Yang, A.C.; Tsai, S.J.; Kuo, P.H. Performing different kinds of physical exercise differentially attenuates the genetic effects on obesity measures: Evidence from 18,424 Taiwan Biobank participants. *PLoS. Genet.* **2019**, *15*, e1008277.
5. Richardson, T.G.; Harrison, S.; Hemani, G.; Davey Smith, G. An atlas of polygenic risk score associations to highlight putative causal relationships across the human phenome. *Elife.* **2019**, *8*, e43657.
6. Jing, J.; Ekici, A.B.; Sitter, T.; Eckardt, K.U.; Schaeffner, E.; Li, Y.; Kronenberg, F.; Köttgen, A.; Schultheiss, U.T. Genetics of serum urate concentrations and gout in a high-risk population, patients with chronic kidney disease. *Sci. Rep.* **2018**, *8*, 13184.
7. Woodward, O.M.; Köttgen, A.; Coresh, J.; Boerwinkle, E.; Guggino, W.B.; Köttgen, M. Identification of a urate transporter, ABCG2, with a common functional polymorphism causing gout. *Proc. Natl. Acad. Sci USA.* **2009**, *106*, 10338-10342.
8. Cadzow, M.; Merriman, T.R.; Dalbeth, N. Performance of gout definitions for genetic epidemiological studies: analysis of UK Biobank. *Arthritis. Res. Ther.* **2017**, *19*, 181.
9. Moran, S.; Arribas, C.; Esteller, M. Validation of a DNA methylation microarray for 850,000 CpG sites of the human genome enriched in enhancer sequences. *Epigenomics.* **2016**, *8*, 389-399.
10. Saare, M.; Modhukur, V.; Suhorutshenko, M.; Rajashekar, B.; Rekker, K.; Sõritsa, D.; Karro, H.; Soplepmann, P.; Sõritsa, A.; Lindgren, C.M., et al. The influence of menstrual cycle and endometriosis on endometrial methylome. *Clin. Epigenetics.* **2016**, *8*, 2.
11. Hannon, E.; Knox, O.; Sugden, K.; Burrage, J.; Wong, C.C.Y.; Belsky, D.W.; Corcoran, D.L.; Arseneault, L.; Moffitt, T.E.; Caspi, A., et al. Characterizing genetic and environmental influences on variable DNA methylation using monozygotic and dizygotic twins. *PLoS. Genet.* **2018**, *14*, e1007544.
12. Zhu, H.; Wang, G.; Qian, J. Transcription factors as readers and effectors of DNA methylation. *Nat. Rev. Genet.* **2016**, *17*, 551-565.
13. Mariño-Ramírez, L.; Spouge, J.L.; Kanga, G.C.; Landsman, D. Statistical analysis of over-represented words in human promoter sequences. *Nucleic. Acids. Res.* **2004**, *32*, 949-958.
14. Schlosberg, C.E.; VanderKraats, N.D.; Edwards, J.R. Modeling complex patterns of differential DNA methylation that associate with gene expression changes. *Nucleic. Acids. Res.* **2017**, *45*, 5100-5111.
15. Swanton, C.; Nicke, B.; Downward, J. RNA interference, DNA methylation, and gene silencing: a bright future for cancer therapy? *Lancet. Oncol.* **2004**, *5*, 653-654.
16. Su, Z.; Xia, J.; Zhao, Z. Functional complementation between transcriptional methylation regulation and post-transcriptional microRNA regulation in the human genome. *BMC. Genomics.* **2011**, *12 Suppl 5*, S15.
17. Lim, K.H.; Park, E.S.; Kim, D.H.; Cho, K.C.; Kim, K.P.; Park, Y.K.; Ahn, S.H.; Park, S.H.; Kim, K.H.; Kim, C.W., et al. Suppression of interferon-mediated anti-HBV response by single CpG methylation in the 5'-UTR of TRIM22. *Gut.* 2018, *67*, 166-178.

18. Lokk, K.; Modhukur, V.; Rajashekar, B.; Märtens, K.; Mägi, R.; Kolde, R.; Koltšina, M.; Nilsson, T.K.; Vilo, J.; Salumets, A., et al. DNA methylome profiling of human tissues identifies global and tissue-specific methylation patterns. *Genome. Biol.* **2014**, *15*, r54.
19. Shvetsova, E.; Sofronova, A.; Monajemi, R.; Gagalova, K.; Draisma, H.H.M.; White, S.J.; Santen, G.W.E.; Chuva de Sousa Lopes, S.M.; Heijmans, B.T.; van Meurs, J., et al. Skewed X-inactivation is common in the general female population. *Eur. J. Hum. Genet.* **2019**, *27*, 455-465.
20. Krzyzewska, I.M.; Ensink, J.B.M.; Nawijn, L.; Mul, A.N.; Koch, S.B.; Venema, A.; Shankar, V.; Frijling, J.L.; Veltman, D.J.; Lindauer, R.J.L., et al. Genetic variant in CACNA1C is associated with PTSD in traumatized police officers. *Eur. J. Hum. Genet.* **2018**, *26*, 247-257.
21. Cardenas, A.; Sordillo, J.E.; Rifas-Shiman, S.L.; Chung, W.; Liang, L.; Coull, B.A.; Hivert, M.F.; Lai, P.S.; Forno, E.; Celedón, J.C., et al. The nasal methylome as a biomarker of asthma and airway inflammation in children. *Nat. Commun.* **2019**, *10*, 3095.
22. Joehanes, R.; Just, A.C.; Marioni, R.E.; Pilling, L.C.; Reynolds, L.M.; Mandaviya, P.R.; Guan, W.; Xu, T.; Elks, C.E.; Aslibekyan, S., et al. Epigenetic Signatures of Cigarette Smoking. *Circ. Cardiovasc. Genet.* **2016**, *9*, 436-447.
23. Xu, K.; Montalvo-Ortiz, J.L.; Zhang, X.; Southwick, S.M.; Krystal, J.H.; Pietrzak, R.H.; Gelernter, J. Epigenome-Wide DNA Methylation Association Analysis Identified Novel Loci in Peripheral Cells for Alcohol Consumption Among European American Male Veterans. *Alcohol. Clin. Exp. Res.* **2019**, *43*, 2111-2121.
24. Fortin, J.P.; Triche, T.J.; Hansen, K.D. Preprocessing, normalization and integration of the Illumina HumanMethylationEPIC array with minfi. *Bioinformatics.* **2017**, *33*, 558-560.
25. Morrison, F.G.; Logue, M.W.; Guetta, R.; Maniates, H.; Stone, A.; Schichman, S.A.; McGlinchey, R.E.; Milberg, W.P.; Miller, M.W.; Wolf, E.J. Investigation of bidirectional longitudinal associations between advanced epigenetic age and peripheral biomarkers of inflammation and metabolic syndrome. *Aging (Albany NY).* **2019**, *11*, 3487-3504.
26. Benjamini, Y.; Hochberg, Y. Controlling the false discovery rate: a practical and powerful approach to multiple testing. *J. R. Stat. Soc. B.* **1995,** *57*, 289-300.
27. Johansson, A.; Palli, D.; Masala, G.; Grioni, S.; Agnoli, C.; Tumino, R.; Giurdanella, M.C.; Fasanelli, F.; Sacerdote, C.; Panico, S., et al. Epigenome-wide association study for lifetime estrogen exposure identifies an epigenetic signature associated with breast cancer risk. *Clin. Epigenetics.* **2019**, *11*, 66.
28. Li, J.; Yuan, X.; March, M.E.; Yao, X.; Sun, Y.; Chang, X.; Hakonarson, H.; Xia, Q.; Meng, X. Identification of Target Genes at Juvenile Idiopathic Arthritis GWAS Loci in Human Neutrophils. *Front. Genet.* **2019**, *10*, 181.
29. Xia, J.; Benner, M.J.; Hancock, R.E. NetworkAnalyst--integrative approaches for protein-protein interaction network analysis and visual exploration. *Nucleic. Acids. Res.* **2014**, *42*, W167-174.
30. Punzi, L.; Scanu, A.; Spinella, P.; Galozzi, P.; Oliviero, F. One year in review 2018: gout. *Clin. Exp. Rheumatol.* **2019**, *37*, 1-11.
31. Pisaniello, H.L.; Lester, S.; Gonzalez-Chica, D.; Stocks, N.; Longo, M.; Sharplin, G.R.; Dal Grande, E.; Gill, T.K.; Whittle, S.L.; Hill, C.L. Gout prevalence and predictors of urate-lowering therapy use: results from a population-based study. *Arthritis. Res. Ther.* **2018**, *20*, 143.
32. Lee, C.H.; Sung, N.Y.; Lee, J.; Bae, S.C. Factors associated with gout in South Koreans: analysis using the National Health Insurance Corporation and the National Health Screening Exam databases. *Clin. Rheumatol.* **2013**, *32*, 829-837.

33. Zhi, D.; Aslibekyan, S.; Irvin, M.R.; Claas, S.A.; Borecki, I.B.; Ordovas, J.M.; Absher, D.M.; Arnett, D.K. SNPs located at CpG sites modulate genome-epigenome interaction. *Epigenetics.* **2013**, *8*, 802-806.
34. Liu, Y.; Aryee, M.J.; Padyukov, L.; Fallin, M.D.; Hesselberg, E.; Runarsson, A.; Reinius, L.; Acevedo, N.; Taub, M.; Ronninger, M., et al. Epigenome-wide association data implicate DNA methylation as an intermediary of genetic risk in rheumatoid arthritis. *Nat. Biotechnol.* **2013**, *31*, 142-147.
35. Zhu, H.; Wu, L.F.; Mo, X.B.; Lu, X.; Tang, H.; Zhu, X.W.; Xia, W.; Guo, Y.F.; Wang, M.J.; Zeng, K.Q., et al. Rheumatoid arthritis-associated DNA methylation sites in peripheral blood mononuclear cells. *Ann. Rheum. Dis.* **2019**, *78*, 36-42.
36. Martin, T.C.; Yet, I.; Tsai, P.C.; Bell, J.T. coMET: visualisation of regional epigenome-wide association scan results and DNA co-methylation patterns. *BMC. Bioinformatics.* **2015**, *16*, 131.
37. Brozovic, M.; Dantec, C.; Dardaillon, J.; Dauga, D.; Faure, E.; Gineste, M.; Louis, A.; Naville, M.; Nitta, K.R.; Piette, J., et al. ANISEED 2017: extending the integrated ascidian database to the exploration and evolutionary comparison of genome-scale datasets. *Nucleic. Acids. Res.* **2018**, *46*, D718-D725.
38. Song, L.; Crawford, G.E. DNase-seq: a high-resolution technique for mapping active gene regulatory elements across the genome from mammalian cells. *Cold. Spring. Harb. Protoc.* **2010**, *2010*, pdb.prot5384.
39. Gusmao, E.G.; Dieterich, C.; Zenke, M.; Costa, I.G. Detection of active transcription factor binding sites with the combination of DNase hypersensitivity and histone modifications. *Bioinformatics.* **2014**, *30*, 3143-3151.
40. Mahmood, N.; Rabbani, S.A. DNA Methylation Readers and Cancer: Mechanistic and Therapeutic Applications. *Front. Oncol.* **2019**, *9*, 489.
41. Kulakovskiy, I.V.; Vorontsov, I.E.; Yevshin, I.S.; Sharipov, R.N.; Fedorova, A.D.; Rumynskiy, E.I.; Medvedeva, Y.A.; Magana-Mora, A.; Bajic, V.B.; Papatsenko, D.A., et al. HOCOMOCO: towards a complete collection of transcription factor binding models for human and mouse via large-scale ChIP-Seq analysis. *Nucleic. Acids. Res.* **2018**, *46*, D252-D259.
42. Chèneby, J.; Ménétrier, Z.; Mestdagh, M.; Rosnet, T.; Douida, A.; Rhalloussi, W.; Bergon, A.; Lopez, F.; Ballester, B. ReMap 2020: a database of regulatory regions from an integrative analysis of Human and Arabidopsis DNA-binding sequencing experiments. *Nucleic. Acids. Res.* **2020**, *48*, D180-D188.
43. Yates, A.D.; Achuthan, P.; Akanni, W.; Allen, J.; Alvarez-Jarreta, J.; Amode, M.R.; Armean, I.M.; Azov, A.G.; Bennett, R.; Bhai, J., et al. Ensembl 2020. *Nucleic Acids Res* **2020**, *48*, D682-D688.
44. John, S.; Chen, H.; Deng, M.; Gui, X.; Wu, G.; Chen, W.; Li, Z.; Zhang, N.; An, Z.; Zhang, C.C. A Novel Anti-LILRB4 CAR-T Cell for the Treatment of Monocytic AML. *Mol. Ther.* **2018**, *26*, 2487-2495.
45. Bredholt, T.; Dimba, E.A.; Hagland, H.R.; Wergeland, L.; Skavland, J.; Fossan, K.O.; Tronstad, K.J.; Johannessen, A.C.; Vintermyr, O.K.; Gjertsen, B.T. Camptothecin and khat (Catha edulis Forsk.) induced distinct cell death phenotypes involving modulation of c-FLIPL, Mcl-1, procaspase-8 and mitochondrial function in acute myeloid leukemia cell lines. *Mol. Cancer.* **2009**, *8*, 101.
46. Balachandran, C.; Emi, N.; Arun, Y.; Yamamoto, N.; Duraipandiyan, V.; Inaguma, Y.; Okamoto, A.; Ignacimuthu, S.; Al-Dhabi, N.A.; Perumal, P.T. In vitro antiproliferative activity of 2,3-dihydroxy-9,10-anthraquinone induced apoptosis against COLO320 cells through cytochrome c release caspase mediated pathway with PI3K/AKT and COX-2 inhibition. *Chem. Biol. Interact.* **2016**, *249*, 23-35.
47. Wang, Q.; Imamura, R.; Motani, K.; Kushiyama, H.; Nagata, S.; Suda, T. Pyroptotic cells externalize eat-me and release find-me signals and are efficiently engulfed by macrophages. *Int. Immunol.* **2013**, *25*, 363-372.

48. Poh, A.R.; Love, C.G.; Masson, F.; Preaudet, A.; Tsui, C.; Whitehead, L.; Monard, S.; Khakham, Y.; Burstroem, L.; Lessene, G., et al. Inhibition of Hematopoietic Cell Kinase Activity Suppresses Myeloid Cell-Mediated Colon Cancer Progression. *Cancer. Cell.* **2017**, *31*, 563-575.e565.

49. Mironov, M.E.; Oleshko, O.S.; Pokrovskii, M.A.; Rybalova, T.V.; Pechurov, V.K.; Pokrovskii, A.G.; Cheresis, S.V.; Mishinov, S.V.; Stupak, V.V.; Shults, E.E. 6-(4'-Aryl-1',2',3'-triazolyl)-spirostan-3,5-diols and 6-(4'-Aryl-1',2',3'-triazolyl)-7-hydroxyspirosta-1,4-dien-3-ones: Synthesis and analysis of their cytotoxicity. *Steroids.* **2019**, *151*, 108460.

50. Davis, S.; Meltzer, P.S. GEOquery: a bridge between the Gene Expression Omnibus (GEO) and BioConductor. *Bioinformatics.* **2007**, *23*, 1846-1847.

51. Smyth, G.K. Linear models and empirical bayes methods for assessing differential expression in microarray experiments. *Stat. Appl. Genet. Mol. Biol.* **2004,** *3*, Article3.

Table S1. Characteristics of study participants.

Characteristics	Participants with Gout (*n* = 69)	Participants without Gout (*n* = 1455)	*p*
Age (years)	52.58 ± 10.98	49.16 ± 11.15	0.0128
Sex, male/female	60/9	683/772	<0.0001
Uric acid (mg/dl)	7.13 ± 1.96	5.53 ± 1.39	<0.0001
Hyperuricemia (uric acid >6 mg/dl)	NA	494 (33.95%)	-
Total cholesterol (mg/dl)	196.70 ± 35.78	194.50 ± 35.49	0.6199
HbA_{1c} (%)	5.96 ± 0.78	5.71 ± 0.73	0.0063
Body mass index	26.05 ± 3.99	24.26 ± 3.57	<0.0001

The results were expressed as mean ± standard deviation. HbA_{1C}: glycosylated hemoglobin. NA: not applicable.

Table S2. Significant CpG sites that were mapped to genes not implicated in IL-1β production nor gouty inflammation.

CpG site	Δβ[a]	*P*	Chr	Position[b]	Gene[c]	Genomic Features	Biologic functions
cg10881514	0.89%	5.50×10^{-7}	1	64239618	*ROR1*	TSS200	regulates cells proliferation and apoptosis [1], migration [2], invasion [3], and drug resistance [4]
cg11168446	0.87%	1.25×10^{-5}	1	94312877	*BCAR3*	TSS200	is associated with proliferation, migration, invasion, drug resistance of cancer [5], and contributes to cancer prognosis, diabetes, and body mass index [6–8]
cg07173100	1.09%	3.07×10^{-6}	2	3606140	*RNASEH1*	TSS1500	degrades RNA:DNA hybrid, rescues DNA damage and replication stress [9–11], and is implicated in progressive external ophthalmoplegia (PEO), mitochondrial encephalomyopathy, and type 1 diabetes [12–14]
cg08086731	-1.41%	7.93×10^{-6}	2	62429249	*B3GNT2*	5'UTR	reduces proliferation of T cells/B cells [15] and contributes to rheumatoid arthritis and psoriatic arthritis [16,17]
cg01334522	-0.80%	1.84×10^{-6}	2	105720678	*UTAT33*	TSS1500	unknown
cg18110444	1.04%	1.94×10^{-6}	2	136743460	*DARS*	TSS1500	is associated with leukodystrophy hypomyelination with brainstem and spinal cord involvement and leg spasticity (HBSL) [18]
cg19648955	0.87%	1.68×10^{-6}	2	177134095	*MTX2*	TSS200	regulates apoptosis [19] and microfilament formation [20]
cg14706579	1.28%	6.34×10^{-6}	2	203736710	*ICA1L*	TSS1500	is implicated in spermiogenesis [21] and cardiovascular disease [22,23]
cg11932445	0.86%	1.09×10^{-5}	2	219263228	*CTDSP1*	TSS1500	blocks cell cycle progression, angiogenesis, tumorigenesis [24,25], and regulates triacylglycerol synthesis [26]
cg08986653	0.92%	1.25×10^{-5}	3	40498475	*RPL14*	TSS1500	loss of heterozygosity in lung and oral cancer [27] and promotes cancer cells migration and invasion [28]
cg07285807	-1.58%	1.53×10^{-6}	3	49235826	*CCDC36*	TSS200	is essential for DNA double strand break formation [29]
cg21194499	0.74%	1.12×10^{-6}	3	52188578	*WDR51A*	5'UTR	is associated with fat mass, insulin resistance, and dwarfism [30]
cg12250142	-0.94%	7.47×10^{-7}	4	15657883	*FBXL5*	TSS1500	participates in iron metabolism, cell proliferation, migration, invasion [31,32], cytotoxic drug resistance, DNA damage response [33,34], protein ubiquitination and degradation [35,36], and is associated with Parkinson's disease [37]
cg01120514	1.18%	1.07×10^{-6}	4	25314414	*ZCCHC4*	5'UTR	mediates ribosomal RNA methylation [38] and attention deficit hyperactivity disorder risk [39]
cg15926342	0.86%	5.41×10^{-6}	5	171881757	*SH3PXD2B*	TSS1500	drives extracellular matrix degradation, cell migration, Frank-Ter Haar syndrome, and glaucoma [40–42]

cg23066234	1.03%	4.50×10^{-6}	5	174178135	*MIR4634*	TSS1500	is differentially expressed in rheumatoid arthritis [43]
cg09341695	-1.15%	2.71×10^{-7}	6	20545304	*CDKAL1*	5'UTR	is associated with insulin secretion and diabetes risk [44–46], inhibits growth hormone synthesis [47], affects anti-tumor necrosis factor (TNF) response [48], cholesterol efflux [49], adipocyte differentiation and mitochondrial function [50,51], vertebral fracture and synovitis-acne-pustulosis-hyperostosis-osteitis (SAPHO) syndrome [52,53]
cg00609363	1.03%	1.69×10^{-6}	6	30028955	*ZNRD1*	TSS200	modulates tumor growth [54], drug resistance [55], and is associated with human immunodeficiency virus (HIV) disease progression [56] and aspirin-induced bronchoconstriction [57]
cg10750989	0.50%	9.96×10^{-6}	6	31371330	*MICA*	TSS200	participates in activation of NK cells and T cells and type 1 diabetes [58–60]
cg17234073	1.23%	3.08×10^{-6}	7	44788095	*ZMIZ2*	TSS1500	promotes cell growth [61]
cg27528426	0.35%	2.07×10^{-6}	7	65540851	*ASL*	5'UTR	is involved in nitric oxide (NO) production and urea cycle disorder [62,63], and modulates cell growth [64], apoptosis, migration [65], and angiogenesis [62]
cg00269643	0.94%	3.79×10^{-6}	7	128784416	*TSPAN33*	TSS1500	regulates ADAM10 function and Notch signaling [66]
cg00213189	-1.01%	7.01×10^{-7}	7	143012820	*CLCN1*	TSS1500	is involved in various neuromuscular disorder, including myotonia congenita [67], paroxysmal kinesigenic dyskinesia [68], and statin-associated myopathy [69]
cg23371350	-0.95%	2.90×10^{-6}	7	149416674	*KRBA1*	5'UTR	is implicated in familial early-onset dementia [70]
cg21169617	0.90%	1.24×10^{-5}	9	34989434	*DNAJB5*	TSS1500	regulates cardiac hypertrophy [71]
cg13332774	0.30%	9.05×10^{-6}	10	106028628	*GSTO2*	TSS200	contributes to zygotic development [72], malignancy susceptibility and prognosis [73–78], neurologic disorders [79–82], pulmonary function and related disorder [83–85], cardiovascular diseases [86], hepatic failure [87], organ transplant rejection [88,89], hypothyroidism [90], arsenic-induced skin lesions [91], end-stage renal disease [92], and cataract [93]
cg08295826	-0.79%	1.33×10^{-6}	10	112706782	*SHOC2*	5'UTR	regulates cancer cells autophagy, proliferation, migration, invasion [94–96], neurite outgrowth and neurodevelopment disorder [97,98]
cg16550453	-1.44%	1.27×10^{-5}	10	115939018	*TDRD1*	TSS200	functions in piRNA biogenesis, retrotransposon silencing [99], and determines risk of spermatogenic impairment [100]
cg03544918	0.51%	5.50×10^{-7}	11	14913944	*CYP2R1*	TSS200	is implicated in ricket [101], multiple sclerosis [102], hepatitis B treatment response [103], Vogt-Koyanagi-Harada disease [104], cardiovascular disease [105,106], diabetes [107,108], cell proliferation [109], intractability of Graves disease [110], and asthma [111]

cg11558328	0.97%	1.17×10^{-5}	11	46722331	*ARHGAP1* (*CDC42GAP*) (112)	TSS1500	regulates proliferation, migration, invasion [112], epithelial-to-mesenchymal transition [113], differentiation [114], aging [115], apoptosis [116], and platelet secretion [117]
cg11766552	1.11%	6.41×10^{-6}	11	65547881	*AP5B1*	TSS200	is involved in atopic march and psoriasis [118,119]
cg10043393	-1.44%	6.94×10^{-6}	11	83354605	*DLG2* (*PSD93*) [120]	5'UTR	inhibits cell division, migration, and is implicated in hemangioblastoma [121,122]; regulates distribution of ion channels within neuronal membranes [123], stabilizes synapse and neuron network activity [120,124], is associated with various aspects of neuropsychiatric development [125–128], schizophrenia, bipolar, autism spectrum disorder [128–130], Parkinson's disease, migraine [131,132], and incriminated in phospholipid level and glucose homeostasis [133,134], myopia, primary open-angle glaucoma, and chronic obstructive pulmonary disease [135,136]
cg19649564	0.93%	1.08×10^{-5}	11	124932892	*SLC37A2*	TSS200	is upregulated upon macrophage differentiation [137]
cg21638242	-1.56%	3.28×10^{-6}	11	134257617	*B3GAT1*	5'UTR	is involved in N-glycosylation, non-syndromic facial cleft, and psychosis [138–140]
cg06405045	0.90%	3.02×10^{-6}	12	31479141	*FAM60A*	TSS200	regulates DNA methylation and histone acetylation [141,142], cell proliferation, migration, invasion [143], and is associated with diabetes risk [144]
cg03916864	0.87%	1.34×10^{-5}	12	109535384	*UNG*	TSS1500	contributes to class-switch recombination [145] and chemotherapy resistance [146]
cg10362742	1.07%	1.53×10^{-6}	13	31736223	*HSPH1* (*HSP105*, *HSP110*) (147-148)	TSS200	regulates cancer growth [147], apoptosis [148], and surface protein expression [149]
cg23235217	0.36%	1.21×10^{-5}	13	100741026	*PCCA*	TSS1500	contributes to propionic acidemia [150]
cg10769343	0.96%	3.03×10^{-6}	14	24617188	*PSME2* (*PA28β*) (151)	TSS1500	is involved in carcinogenesis [151]
cg13774948	1.11%	1.49×10^{-6}	14	53019342	*TXNDC16*	TSS200	an endoplasmic reticulum (ER) luminal glycoprotein [152]
cg24547356	0.15%	1.23×10^{-5}	14	103995608	*TRMT61A*	5'UTR	is involved in RNA methylation [153]
cg00570642	1.02%	1.26×10^{-6}	16	30022576	*DOC2A*	TSS200	is involved in exocytosis and insulin secretion [154,155], brain development, and schizophrenia [156,157]
cg18129863	1.06%	1.02×10^{-5}	16	30886426	*MIR762HG*	TSS1500	unknown
cg18315103	0.46%	9.61×10^{-6}	16	67571472	*FAM65A*	5'UTR	regulates Golgi reorientation during cell migration [158]
cg10274403	1.07%	6.12×10^{-7}	16	84178812	*LRRC50* (*DNAAF1*) [159]	TSS200	acts as a tumor suppressor [159] and mutation leads to primary ciliary dyskinesia [160]

cg10810352	0.27%	8.98×10^{-6}	16	87417434	*FBXO31*	TSS200	participates in various aspects of tumorigenesis [161–164] and neuronal morphogenesis and migration [165]
cg04329264	0.46%	7.82×10^{-6}	16	89557219	*ANKRD11*	TSS1500	contributes to neural development [166,167] and neurodevelopment disorder [168], various aspects of tumorigenesis [169,170] and bone homeostasis [171]
cg26963795	1.32%	1.56×10^{-5}	17	2206215	*SRR* (*Serine racemase*) [172]	TSS1500	participates in cells migration, invasion, neovascularization [172,173], neurologic functions (synaptic plasticity, cerebellar development, neuron activation, long-term potentiation, sleep regulation [174–178]), and is implicated in various neuropsychiatric disorder (post-traumatic disorder, sociality deficits, substance abuse, depression [179–182], migraine, neuropathic pain [183,184], cognitive decline [185]) and glucose homeostasis, diabetes, and diabetic retinopathy [185–187]
cg11295144	1.02%	3.85×10^{-6}	17	33447001	*RAD51L3* (*RAD51D*) [188]	TSS200	protects against chromosome damage and is associated with hepatocellular carcinoma [188,189]
cg24142633	-1.06%	4.39×10^{-6}	17	54893277	*C17orf67*	TSS200	determines body height and predisposition to adolescent idiopathic scoliosis [190,191]
cg09288233	1.00%	4.88×10^{-6}	18	3449455	*TGIF1* (*TGIF*) [192]	TSS1500	modulates transforming growth factor (TGF)-β signaling [193], proliferation, migration, invasion [194,195], angiogenesis [196], bone remodeling, chondrogenesis [197,198], fibrosis [199], metabolism [200–202], and contributes to neurodevelopment disorder [203,204], chronic otitis media [192], colistin nephrotoxicity [205], and myopia [206]
cg21486148	-0.74%	6.03×10^{-6}	19	1070688	*HMHA1*	TSS1500	regulates proliferation, migration, invasion, and endothelial barrier function [207,208]
cg14700108	-1.69%	1.90×10^{-6}	19	2637722	*GNG7*	5'UTR	inhibits proliferation and differentiation [209,210], and is implicated in end-stage kidney disease [211], and neuropsychiatric disorder such as stroke [212] and depression [213]
cg10105623	-0.72%	5.93×10^{-6}	19	34893958	*PDCD2L*	TSS1500	promotes β cell apoptosis [214]
cg15592828	-0.85%	8.16×10^{-7}	20	36890518	*KIAA1755*	TSS1500	modulates levels of eicosapentaenoate [215]
cg17758280	0.85%	7.80×10^{-6}	22	29702766	*GAS2L1*	TSS1500	mediates centrosome dynamics and disjunction [216]
cg04483623	0.84%	1.51×10^{-5}	22	29949575	*THOC5*	5'UTR	is responsible for nuclear RNA export [217] and regulates cell death, differentiation, proliferation, migration [218,219], synapse maintenance [220], glucose and high density lipoproteins (HDL) levels [221,222]

Chr: chromosome. [a]Methylation levels of gout minus methylation levels of non-gout after adjusting for sex, age, smoking history (total pack-years), smoking status, alcohol consumption, and cell subsets. [b]Positions of the CpG sites in hg19. [c]Gene names and their alias.

References

1. Wang, H.L.; Liu, Y.C.; Long, M.P.; Zheng, C.; Yang, J.H. Blocking ROR1 enhances the roles of erlotinib in lung adenocarcinoma cell lines. Oncol. Lett. 2019, 18, 2977-2984.
2. Fultang, N.; Illendula, A.; Chen, B.; Wu, C.; Jonnalagadda, S.; Baird, N.; Klase, Z.; Peethambaran, B. Strictinin, a novel ROR1-inhibitor, represses triple negative breast cancer survival and migration via modulation of PI3K/AKT/GSK3ß activity. PLoS. One. 2019, 14, e0217789.
3. Wu, X.; Yan, T.; Hao, L.; Zhu, Y. Wnt5a induces ROR1 and ROR2 to activate RhoA in esophageal squamous cell carcinoma cells. Cancer. Manag. Res. 2019, 11, 2803-2815.
4. Karvonen, H.; Barker, H.; Kaleva, L.; Niininen, W.; Ungureanu, D. Molecular Mechanisms Associated with ROR1-Mediated Drug Resistance: Crosstalk with Hippo-YAP/TAZ and BMI-1 Pathways. Cells. 2019, 8, 812.
5. Meng, X.; Liu, J.; Wang, H.; Chen, P.; Wang, D. MicroRNA-126-5p downregulates BCAR3 expression to promote cell migration and invasion in endometriosis. Mol. Cell. Endocrinol. 2019, 494, 110486.
6. Guo, J.; Canaff, L.; Rajadurai, C.V.; Fils-Aimé, N.; Tian, J.; Dai, M.; Korah, J.; Villatoro, M.; Park, M.; Ali, S., et al. Breast cancer anti-estrogen resistance 3 inhibits transforming growth factor β/Smad signaling and associates with favorable breast cancer disease outcomes. Breast. Cancer. Res. 2014, 16, 476.
7. Schierding, W.; O'Sullivan, J.M. Connecting SNPs in Diabetes: A Spatial Analysis of Meta-GWAS Loci. Front. Endocrinol (Lausanne). 2015, 6, 102.
8. Chu, S.H.; Loucks, E.B.; Kelsey, K.T.; Gilman, S.E.; Agha, G.; Eaton, C.B.; Buka, S.L.; Huang, Y.T. Sex-specific epigenetic mediators between early life social disadvantage and adulthood BMI. Epigenomics. 2018, 10, 707-722.
9. Lima, W.F.; Murray, H.M.; Damle, S.S.; Hart, C.E.; Hung, G.; De Hoyos, C.L.; Liang, X.H.; Crooke, S.T. Viable RNaseH1 knockout mice show RNaseH1 is essential for R loop processing, mitochondrial and liver function. Nucleic. Acids. Res. 2016, 44, 5299-5312.
10. De Magis, A.; Manzo, S.G.; Russo, M.; Marinello, J.; Morigi, R.; Sordet, O.; Capranico, G. DNA damage and genome instability by G-quadruplex ligands are mediated by R loops in human cancer cells. Proc. Natl. Acad. Sci USA. 2019, 116, 816-825.
11. Silva, B.; Pentz, R.; Figueira, A.M.; Arora, R.; Lee, Y.W.; Hodson, C.; Wischnewski, H.; Deans, A.J.; Azzalin, C.M. FANCM limits ALT activity by restricting telomeric replication stress induced by deregulated BLM and R-loops. Nat. Commun. 2019, 10, 2253.
12. Carreño-Gago, L.; Blázquez-Bermejo, C.; Díaz-Manera, J.; Cámara, Y.; Gallardo, E.; Martí, R.; Torres-Torronteras, J.; García-Arumí, E. Identification and Characterization of New RNASEH1 Mutations Associated With PEO Syndrome and Multiple Mitochondrial DNA Deletions. Front. Genet. 2019, 10, 576.
13. Reyes, A.; Melchionda, L.; Nasca, A.; Carrara, F.; Lamantea, E.; Zanolini, A.; Lamperti, C.; Fang, M.; Zhang, J.; Ronchi, D., et al. RNASEH1 Mutations Impair mtDNA Replication and Cause Adult-Onset Mitochondrial Encephalomyopathy. Am. J. Hum. Genet. 2015, 97, 186-193.
14. Pineda-Trujillo, N.; Rodríguez-Acevedo, A.; Rodríguez, A.; Ruíz-Linares, A.; Bedoya, G.; Rivera, A.; Alfaro, J.M. RNASEH1 gene variants are associated with autoimmune type 1 diabetes in Colombia. J. Endocrinol. Invest. 2018, 41, 755-764.
15. Togayachi, A.; Kozono, Y.; Kuno, A.; Ohkura, T.; Sato, T.; Hirabayashi, J.; Ikehara, Y.; Narimatsu, H. Beta3GnT2 (B3GNT2), a major polylactosamine synthase: analysis of B3GNT2-deficient mice. Methods. Enzymol. 2010, 479, 185-204.
16. Okada, Y.; Terao, C.; Ikari, K.; Kochi, Y.; Ohmura, K.; Suzuki, A.; Kawaguchi, T.; Stahl, E.A.; Kurreeman, F.A.; Nishida, N., et al. Meta-analysis identifies nine new loci associated with rheumatoid arthritis in the Japanese population. Nat. Genet. 2012, 44, 511-516.
17. Aterido, A.; Cañete, J.D.; Tornero, J.; Ferrándiz, C.; Pinto, J.A.; Gratacós, J.; Queiró, R.; Montilla, C.; Torre-Alonso, J.C.; Pérez-Venegas, J.J., et al. Genetic variation at the glycosaminoglycan metabolism pathway contributes to the risk of psoriatic arthritis but not psoriasis. Ann. Rheum. Dis. 2019, 78, 355-364.
18. Fröhlich, D.; Suchowerska, A.K.; Voss, C.; He, R.; Wolvetang, E.; von Jonquieres, G.; Simons, C.; Fath, T.; Housley, G.D.; Klugmann, M. Expression Pattern of the Aspartyl-tRNA Synthetase DARS in the Human Brain. Front. Mol. Neurosci. 2018, 11, 81.
19. Cartron, P.F.; Petit, E.; Bellot, G.; Oliver, L.; Vallette, F.M. Metaxins 1 and 2, two proteins of the mitochondrial protein sorting and assembly machinery, are essential for Bak activation during TNF alpha triggered apoptosis. Cell. Signal. 2014, 26, 1928-1934.

20. Wilkins, S.J.; Yoong, S.; Verkade, H.; Mizoguchi, T.; Plowman, S.J.; Hancock, J.F.; Kikuchi, Y.; Heath, J.K.; Perkins, A.C. Mtx2 directs zebrafish morphogenetic movements during epiboly by regulating microfilament formation. Dev. Biol. 2008, 314, 12-22.
21. He, J.; Xia, M.; Tsang, W.H.; Chow, K.L.; Xia, J. ICA1L forms BAR-domain complexes with PICK1 and is crucial for acrosome formation in spermiogenesis. J. Cell. Sci. 2015, 128, 3822-3836.
22. Wang, X.; Mo, X.; Zhang, H.; Zhang, Y.; Shen, Y. Identification of Phosphorylation Associated SNPs for Blood Pressure, Coronary Artery Disease and Stroke from Genome-wide Association Studies. Curr. Mol. Med. 2019, 19, 731-738.
23. Chung, J.; Marini, S.; Pera, J.; Norrving, B.; Jimenez-Conde, J.; Roquer, J.; Fernandez-Cadenas, I.; Tirschwell, D.L.; Selim, M.; Brown, D.L., et al. Genome-wide association study of cerebral small vessel disease reveals established and novel loci. Brain. 2019, 142, 3176-3189.
24. Zhu, Y.; Lu, Y.; Zhang, Q.; Liu, J.J.; Li, T.J.; Yang, J.R.; Zeng, C.; Zhuang, S.M. MicroRNA-26a/b and their host genes cooperate to inhibit the G1/S transition by activating the pRb protein. Nucleic. Acids. Res. 2012, 40, 4615-4625.
25. Liao, P.; Wang, W.; Li, Y.; Wang, R.; Jin, J.; Pang, W.; Chen, Y.; Shen, M.; Wang, X.; Jiang, D., et al. Palmitoylated SCP1 is targeted to the plasma membrane and negatively regulates angiogenesis. Elife. 2017, 6, e22058.
26. Wang, H.; Luo, J.; He, Q.; Yao, D.; Wu, J.; Loor, J.J. miR-26b promoter analysis reveals regulatory mechanisms by lipid-related transcription factors in goat mammary epithelial cells. J. Dairy. Sci. 2017, 100, 5837-5849.
27. Shriver, S.P.; Shriver, M.D.; Tirpak, D.L.; Bloch, L.M.; Hunt, J.D.; Ferrell, R.E.; Siegfried, J.M. Trinucleotide repeat length variation in the human ribosomal protein L14 gene (RPL14): localization to 3p21.3 and loss of heterozygosity in lung and oral cancers. Mutat. Res. 1998, 406, 9-23.
28. Feng, Y.; Ma, J.; Fan, H.; Liu, M.; Zhu, Y.; Li, Y.; Tang, H. TNF-α-induced lncRNA LOC105374902 promotes the malignant behavior of cervical cancer cells by acting as a sponge of miR-1285-3p. Biochem. Biophys. Res. Commun. 2019, 513, 56-63.
29. Stanzione, M.; Baumann, M.; Papanikos, F.; Dereli, I.; Lange, J.; Ramlal, A.; Tränkner, D.; Shibuya, H.; de Massy, B.; Watanabe, Y., et al. Meiotic DNA break formation requires the unsynapsed chromosome axis-binding protein IHO1 (CCDC36) in mice. Nat. Cell. Biol. 2016, 18, 1208-1220.
30. Rzehak, P.; Covic, M.; Saffery, R.; Reischl, E.; Wahl, S.; Grote, V.; Weber, M.; Xhonneux, A.; Langhendries, J.P.; Ferre, N., et al. DNA-Methylation and Body Composition in Preschool Children: Epigenome-Wide-Analysis in the European Childhood Obesity Project (CHOP)-Study. Sci. Rep. 2017, 7, 14349.
31. Johnson, N.B.; Deck, K.M.; Nizzi, C.P.; Eisenstein, R.S. A synergistic role of IRP1 and FBXL5 proteins in coordinating iron metabolism during cell proliferation. J. Biol. Chem. 2017, 292, 15976-15989.
32. He, Z.J.; Li, W.; Chen, H.; Wen, J.; Gao, Y.F.; Liu, Y.J. miR-1306-3p targets FBXL5 to promote metastasis of hepatocellular carcinoma through suppressing snail degradation. Biochem. Biophys. Res. Commun. 2018, 504, 820-826.
33. Wu, W.D.; Wang, M.; Ding, H.H.; Qiu, Z.J. FBXL5 attenuates RhoGDI2-induced cisplatin resistance in gastric cancer cells. Eur. Rev. Med. Pharmacol. Sci. 2016, 20, 2551-2557.
34. Chen, Z.W.; Liu, B.; Tang, N.W.; Xu, Y.H.; Ye, X.Y.; Li, Z.M.; Niu, X.M.; Shen, S.P.; Lu, S.; Xu, L. FBXL5-mediated degradation of single-stranded DNA-binding protein hSSB1 controls DNA damage response. Nucleic. Acids. Res. 2014, 42, 11560-11569.
35. Zhang, N.; Liu, J.; Ding, X.; Aikhionbare, F.; Jin, C.; Yao, X. FBXL5 interacts with p150Glued and regulates its ubiquitination. Biochem. Biophys. Res. Commun. 2007, 359, 34-39.
36. Machado-Oliveira, G.; Guerreiro, E.; Matias, A.C.; Facucho-Oliveira, J.; Pacheco-Leyva, I.; Bragança, J. FBXL5 modulates HIF-1α transcriptional activity by degradation of CITED2. Arch. Biochem. Biophys. 2015, 576, 61-72.
37. Gerez, J.A.; Prymaczok, N.C.; Rockenstein, E.; Herrmann, U.S.; Schwarz, P.; Adame, A.; Enchev, R.I.; Courtheoux, T.; Boersema, P.J.; Riek, R., et al. A cullin-RING ubiquitin ligase targets exogenous α-synuclein and inhibits Lewy body-like pathology. Sci. Transl. Med. 2019, 11, eaau6722.
38. Ma, H.; Wang, X.; Cai, J.; Dai, Q.; Natchiar, S.K.; Lv, R.; Chen, K.; Lu, Z.; Chen, H.; Shi, Y.G., et al. N6-Methyladenosine methyltransferase ZCCHC4 mediates ribosomal RNA methylation. Nat. Chem. Biol. 2019, 15, 88-94.

39. Zayats, T.; Jacobsen, K.K.; Kleppe, R.; Jacob, C.P.; Kittel-Schneider, S.; Ribasés, M.; Ramos-Quiroga, J.A.; Richarte, V.; Casas, M.; Mota, N.R., et al. Exome chip analyses in adult attention deficit hyperactivity disorder. Transl. Psychiatry. 2016, 6, e923.
40. Buschman, M.D.; Bromann, P.A.; Cejudo-Martin, P.; Wen, F.; Pass, I.; Courtneidge, S.A. The novel adaptor protein Tks4 (SH3PXD2B) is required for functional podosome formation. Mol. Biol. Cell. 2009, 20, 1302-1311.
41. Bögel, G.; Gujdár, A.; Geiszt, M.; Lányi, Á.; Fekete, A.; Sipeki, S.; Downward, J.; Buday, L. Frank-ter Haar syndrome protein Tks4 regulates epidermal growth factor-dependent cell migration. J. Biol. Chem. 2012, 287, 31321-31329.
42. Mao, M.; Hedberg-Buenz, A.; Koehn, D.; John, S.W.; Anderson, M.G. Anterior segment dysgenesis and early-onset glaucoma in nee mice with mutation of Sh3pxd2b. Invest. Ophthalmol. Vis. Sci. 2011, 52, 2679-2688.
43. Wang, W.; Zhang, Y.; Zhu, B.; Duan, T.; Xu, Q.; Wang, R.; Lu, L.; Jiao, Z. Plasma microRNA expression profiles in Chinese patients with rheumatoid arthritis. Oncotarget. 2015, 6, 42557-42568.
44. Steinthorsdottir, V.; Thorleifsson, G.; Reynisdottir, I.; Benediktsson, R.; Jonsdottir, T.; Walters, G.B.; Styrkarsdottir, U.; Gretarsdottir, S.; Emilsson, V.; Ghosh, S., et al. A variant in CDKAL1 influences insulin response and risk of type 2 diabetes. Nat. Genet. 2007, 39, 770-775.
45. Musambil, M.; Siddiqui, K. Genetics and genomics studies in type 2 diabetes: A brief review of the current scenario in the Arab region. Diabetes. Metab. Syndr. 2019, 13, 1629-1632.
46. Wang, K.; Chen, Q.; Feng, Y.; Yang, H.; Wu, W.; Zhang, P.; Wang, Y.; Ko, J.; Zhao, F.; Du, W., et al. Single Nucleotide Polymorphisms in CDKAL1 Gene Are Associated with Risk of Gestational Diabetes Mellitus in Chinese Population. J. Diabetes. Res. 2019, 2019, 3618103.
47. Takesue, Y.; Wei, F.Y.; Fukuda, H.; Tanoue, Y.; Yamamoto, T.; Chujo, T.; Shinojima, N.; Yano, S.; Morioka, M.; Mukasa, A., et al. Regulation of growth hormone biosynthesis by Cdk5 regulatory subunit associated protein 1-like 1 (CDKAL1) in pituitary adenomas. Endocr. J. 2019, 66, 807-816.
48. Coto-Segura, P.; Batalla, A.; González-Fernández, D.; Gómez, J.; Santos-Juanes, J.; Queiro, R.; Alonso, B.; Iglesias, S.; Coto, E. CDKAL1 gene variants affect the anti-TNF response among Psoriasis patients. Int. Immunopharmacol. 2015, 29, 947-949.
49. Cheon, E.J.; Cha, D.H.; Cho, S.K.; Noh, H.M.; Park, S.; Kang, S.M.; Gee, H.Y.; Lee, S.H. Novel association between CDKAL1 and cholesterol efflux capacity: Replication after GWAS-based discovery. Atherosclerosis. 2018, 273, 21-27.
50. Take, K.; Waki, H.; Sun, W.; Wada, T.; Yu, J.; Nakamura, M.; Aoyama, T.; Yamauchi, T.; Kadowaki, T. CDK5 Regulatory Subunit-Associated Protein 1-like 1 Negatively Regulates Adipocyte Differentiation through Activation of Wnt Signaling Pathway. Sci. Rep. 2017, 7, 7326.
51. Palmer, C.J.; Bruckner, R.J.; Paulo, J.A.; Kazak, L.; Long, J.Z.; Mina, A.I.; Deng, Z.; LeClair, K.B.; Hall, J.A.; Hong, S., et al. Cdkal1, a type 2 diabetes susceptibility gene, regulates mitochondrial function in adipose tissue. Mol. Metab. 2017, 6, 1212-1225.
52. Zhou, H.; Mori, S.; Ishizaki, T.; Takahashi, A.; Matsuda, K.; Koretsune, Y.; Minami, S.; Higashiyama, M.; Imai, S.; Yoshimori, K., et al. Genetic risk score based on the prevalence of vertebral fracture in Japanese women with osteoporosis. Bone. Rep. 2016, 5, 168-172.
53. Li, N.; Ma, J.; Li, K.; Guo, C.; Ming, L. Different Contributions of CDKAL1, KIF21B, and LRRK2/MUC19 Polymorphisms to SAPHO Syndrome, Rheumatoid Arthritis, Ankylosing Spondylitis, and Seronegative Spondyloarthropathy. Genet. Test. Mol. Biomarkers. 2017, 21, 122-126.
54. Hong, L.; Zhao, Y.; Han, Y.; Guo, W.; Jin, H.; Qiao, T.; Che, Z.; Fan, D. Mechanisms of growth arrest by zinc ribbon domain-containing 1 in gastric cancer cells. Carcinogenesis. 2007, 28, 1622-1628.
55. Shang, Y.; Zhang, Z.; Liu, Z.; Feng, B.; Ren, G.; Li, K.; Zhou, L.; Sun, Y.; Li, M.; Zhou, J., et al. miR-508-5p regulates multidrug resistance of gastric cancer by targeting ABCB1 and ZNRD1. Oncogene. 2014, 33, 3267-3276.
56. An, P.; Goedert, J.J.; Donfield, S.; Buchbinder, S.; Kirk, G.D.; Detels, R.; Winkler, C.A. Regulatory variation in HIV-1 dependency factor ZNRD1 associates with host resistance to HIV-1 acquisition. J. Infect. Dis. 2014, 210, 1539-1548.
57. Pasaje, C.F.; Bae, J.S.; Park, B.L.; Cheong, H.S.; Jang, A.S.; Uh, S.T.; Kim, M.K.; Kim, J.H.; Park, T.J.; Lee, J.S., et al. A possible association between ZNRD1 and aspirin-induced airway bronchoconstriction in a Korean population. J. Investig. Allergol. Clin. Immunol. 2012, 22, 193-200.

58. Bauer, S.; Groh, V.; Wu, J.; Steinle, A.; Phillips, J.H.; Lanier, L.L.; Spies, T. Activation of NK cells and T cells by NKG2D, a receptor for stress-inducible MICA. Science. 1999, 285, 727-729.
59. Boukouaci, W.; Al-Daccak, R.; Dulphy, N.; Lauden, L.; Amokrane, K.; Fortier, C.; Marzais, F.; Bennabi, M.; Peffault de Latour, R.; Socie, G., et al. Soluble MICA-NKG2D interaction upregulates IFN-γ production by activated CD3-CD56+ NK cells: potential impact on chronic graft versus host disease. Hum. Immunol. 2013, 74, 1536-1541.
60. Kawasaki, E.; Matsuura, N.; Eguchi, K. Type 1 diabetes in Japan. Diabetologia. 2006, 49, 828-836.
61. Lee, S.H.; Zhu, C.; Peng, Y.; Johnson, D.T.; Lehmann, L.; Sun, Z. Identification of a novel role of ZMIZ2 protein in regulating the activity of the Wnt/β-catenin signaling pathway. J. Biol. Chem. 2013, 288, 35913-35924.
62. Kho, J.; Tian, X.; Wong, W.T.; Bertin, T.; Jiang, M.M.; Chen, S.; Jin, Z.; Shchelochkov, O.A.; Burrage, L.C.; Reddy, A.K., et al. Argininosuccinate Lyase Deficiency Causes an Endothelial-Dependent Form of Hypertension. Am. J. Hum. Genet. 2018, 103, 276-287.
63. Silvera-Ruiz, S.M.; Arranz, J.A.; Häberle, J.; Angaroni, C.J.; Bezard, M.; Guelbert, N.; Becerra, A.; Peralta, F.; de Kremer, R.D.; Laróvere, L.E. Urea cycle disorders in Argentine patients: clinical presentation, biochemical and genetic findings. Orphanet. J. Rare. Dis. 2019, 14, 203.
64. Hung, Y.H.; Huang, H.L.; Chen, W.C.; Yen, M.C.; Cho, C.Y.; Weng, T.Y.; Wang, C.Y.; Chen, Y.L.; Chen, L.T.; Lai, M.D. Argininosuccinate lyase interacts with cyclin A2 in cytoplasm and modulates growth of liver tumor cells. Oncol. Rep. 2017, 37, 969-978.
65. Premkumar, M.H.; Sule, G.; Nagamani, S.C.; Chakkalakal, S.; Nordin, A.; Jain, M.; Ruan, M.Z.; Bertin, T.; Dawson, B.; Zhang, J., et al. Argininosuccinate lyase in enterocytes protects from development of necrotizing enterocolitis. Am. J. Physiol. Gastrointest. Liver. Physiol. 2014, 307, G347-354.
66. Jouannet, S.; Saint-Pol, J.; Fernandez, L.; Nguyen, V.; Charrin, S.; Boucheix, C.; Brou, C.; Milhiet, P.E.; Rubinstein, E. TspanC8 tetraspanins differentially regulate the cleavage of ADAM10 substrates, Notch activation and ADAM10 membrane compartmentalization. Cell. Mol. Life. Sci. 2016, 73, 1895-1915.
67. Yang, X.; Jia, H.; An, R.; Xi, J.; Xu, Y. Sequence CLCN1 and SCN4A in patients with Nondystrophic myotonias in Chinese populations: Genetic and pedigree analysis of 10 families and review of the literature. Channels (Austin). 2017, 11, 55-65.
68. Wang, H.X.; Li, H.F.; Liu, G.L.; Wen, X.D.; Wu, Z.Y. Mutation Analysis of MR-1, SLC2A1, and CLCN1 in 28 PRRT2-negative Paroxysmal Kinesigenic Dyskinesia Patients. Chin. Med. J (Engl). 2016, 129, 1017-1021.
69. Neřoldová, M.; Stránecký, V.; Hodaňová, K.; Hartmannová, H.; Piherová, L.; Přistoupilová, A.; Mrázová, L.; Vrablík, M.; Adámková, V.; Hubáček, J.A., et al. Rare variants in known and novel candidate genes predisposing to statin-associated myopathy. Pharmacogenomics. 2016, 17, 1405-1414.
70. Alexander, J.; Kalev, O.; Mehrabian, S.; Traykov, L.; Raycheva, M.; Kanakis, D.; Drineas, P.; Lutz, M.I.; Ströbel, T.; Penz, T., et al. Familial early-onset dementia with complex neuropathologic phenotype and genomic background. Neurobiol. Aging. 2016, 42, 199-204.
71. Ago, T.; Liu, T.; Zhai, P.; Chen, W.; Li, H.; Molkentin, J.D.; Vatner, S.F.; Sadoshima, J. A redox-dependent pathway for regulating class II HDACs and cardiac hypertrophy. Cell. 2008, 133, 978-993.
72. Hamilton, L.E.; Suzuki, J.; Aguila, L.; Meinsohn, M.C.; Smith, O.E.; Protopapas, N.; Xu, W.; Sutovsky, P.; Oko, R. Sperm-borne glutathione-S-transferase omega 2 accelerates the nuclear decondensation of spermatozoa during fertilization in mice†. Biol. Reprod. 2019, 101, 368-376.
73. Djukic, T.; Simic, T.; Radic, T.; Matic, M.; Pljesa-Ercegovac, M.; Suvakov, S.; Coric, V.; Pekmezovic, T.; Novakovic, I.; Dragicevic, D., et al. GSTO1*C/GSTO2*G haplotype is associated with risk of transitional cell carcinoma of urinary bladder. Int. Urol. Nephrol. 2015, 47, 625-630.
74. Djukic, T.I.; Savic-Radojevic, A.R.; Pekmezovic, T.D.; Matic, M.G.; Pljesa-Ercegovac, M.S.; Coric, V.M.; Radic, T.M.; Suvakov, S.R.; Krivic, B.N.; Dragicevic, D.P., et al. Glutathione S-transferase T1, O1 and O2 polymorphisms are associated with survival in muscle invasive bladder cancer patients. PLoS. One. 2013, 8, e74724.
75. Masoudi, M.; Saadat, I.; Omidvari, S.; Saadat, M. Association between N142D genetic polymorphism of GSTO2 and susceptibility to colorectal cancer. Mol. Biol. Rep. 2011, 38, 4309-4313.
76. Sharif, M.R.; Sharif, A.; Kheirkhah, D.; Taghavi Ardakan, M.; Soltani, N. Association of GSTO1 A140D and GSTO2 N142D Gene Variations with Breast Cancer Risk. Asian. Pac. J. Cancer. Prev. 2017, 18, 1723-1727.
77. Qu, K.; Liu, S.S.; Wang, Z.X.; Huang, Z.C.; Liu, S.N.; Chang, H.L.; Xu, X.S.; Lin, T.; Dong, Y.F.; Liu, C. Polymorphisms of glutathione S-transferase genes and survival of resected hepatocellular carcinoma patients. World. J. Gastroenterol. 2015, 21, 4310-4322.

78. Pongstaporn, W.; Pakakasama, S.; Sanguansin, S.; Hongeng, S.; Petmitr, S. Polymorphism of glutathione S-transferase Omega gene: association with risk of childhood acute lymphoblastic leukemia. J. Cancer. Res. Clin. Oncol. 2009, 135, 673-678.
79. Allen, M.; Zou, F.; Chai, H.S.; Younkin, C.S.; Miles, R.; Nair, A.A.; Crook, J.E.; Pankratz, V.S.; Carrasquillo, M.M.; Rowley, C.N., et al. Glutathione S-transferase omega genes in Alzheimer and Parkinson disease risk, age-at-diagnosis and brain gene expression: an association study with mechanistic implications. Mol. Neurodegener. 2012, 7, 13.
80. Almaguer-Mederos, L.E.; Almaguer-Gotay, D.; Aguilera-Rodríguez, R.; González-Zaldívar, Y.; Cuello-Almarales, D.; Laffita-Mesa, J.; Vázquez-Mojena, Y.; Zayas-Feria, P.; Rodríguez-Labrada, R.; Velázquez-Pérez, L., et al. Association of glutathione S-transferase omega polymorphism and spinocerebellar ataxia type 2. J. Neurol. Sci. 2017, 372, 324-328.
81. Wahner, A.D.; Glatt, C.E.; Bronstein, J.M.; Ritz, B. Glutathione S-transferase mu, omega, pi, and theta class variants and smoking in Parkinson's disease. Neurosci. Lett. 2007, 413, 274-278.
82. van de Giessen, E.; Fogh, I.; Gopinath, S.; Smith, B.; Hu, X.; Powell, J.; Andersen, P.; Nicholson, G.; Al Chalabi, A.; Shaw, C.E. Association study on glutathione S-transferase omega 1 and 2 and familial ALS. Amyotroph. Lateral. Scler. 2008, 9, 81-84.
83. de Jong, K.; Boezen, H.M.; Hacken, N.H.; Postma, D.S.; Vonk, J.M.; study, L.c. GST-omega genes interact with environmental tobacco smoke on adult level of lung function. Respir. Res. 2013, 14, 83.
84. Piacentini, S.; Polimanti, R.; Iorio, A.; Cortesi, M.; Papa, F.; Rongioletti, M.; Liumbruno, G.M.; Manfellotto, D.; Fuciarelli, M. GSTA1*-69C/T and GSTO2*N142D as asthma- and allergy-related risk factors in Italian adult patients. Clin. Exp. Pharmacol. Physiol. 2014, 41, 180-184.
85. Yanbaeva, D.G.; Wouters, E.F.; Dentener, M.A.; Spruit, M.A.; Reynaert, N.L. Association of glutathione-S-transferase omega haplotypes with susceptibility to chronic obstructive pulmonary disease. Free. Radic. Res. 2009, 43, 738-743.
86. Bilgin, E.; Can Demirdöğen, B.; Türkanoğlu Özçelik, A.; Demirkaya, Ş.; Adalı, O. Association analysis of Glutathione S-transferase omega-1 and omega-2 genetic polymorphisms and ischemic stroke risk in a Turkish population. Neurol. Res. 2019, 41, 118-124.
87. Khosravi, M.; Saadat, I.; Karimi, M.H.; Geramizadeh, B.; Saadat, M. Glutathione S-transferase Omega 2 Genetic Polymorphism and Risk of Hepatic Failure that Lead to Liver Transplantation in Iranian Population. Int. J. Organ. Transplant. Med. 2013, 4, 16-20.
88. Khosravi, M.; Saadat, I.; Karimi, M.H.; Malek Hosseini, S.A. Association of GSTO2 (N142D) Genetic Polymorphism and Acute Rejection of Liver. Int. J. Organ. Transplant. Med. 2016, 7, 183-187.
89. Nekooie-Marnany, N.; Saadat, I.; Karimi, M.H.; Roozbeh, J.; Saadat, M. Influence of GSTO2 (N142D) genetic polymorphism on acute renal rejection. Mol. Biol. Rep. 2013, 40, 4857-4860.
90. Piacentini, S.; Monaci, P.M.; Polimanti, R.; Manfellotto, D.; Fuciarelli, M. GSTO2*N142D gene polymorphism associated with hypothyroidism in Italian patients. Mol. Biol. Rep. 2013, 40, 1967-1971.
91. Luo, L.; Li, Y.; Gao, Y.; Zhao, L.; Feng, H.; Wei, W.; Qiu, C.; He, Q.; Zhang, Y.; Fu, S., et al. Association between arsenic metabolism gene polymorphisms and arsenic-induced skin lesions in individuals exposed to high-dose inorganic arsenic in northwest China. Sci. Rep. 2018, 8, 413.
92. Cimbaljevic, S.; Suvakov, S.; Matic, M.; Pljesa-Ercegovac, M.; Pekmezovic, T.; Radic, T.; Coric, V.; Damjanovic, T.; Dimkovic, N.; Markovic, R., et al. Association of GSTO1 and GSTO2 Polymorphism with Risk of End-Stage Renal Disease Development and Patient Survival. J. Med. Biochem. 2016, 35, 302-311.
93. Stamenkovic, M.; Radic, T.; Stefanovic, I.; Coric, V.; Sencanic, I.; Pljesa-Ercegovac, M.; Matic, M.; Jaksic, V.; Simic, T.; Savic-Radojevic, A. Glutathione S-transferase omega-2 polymorphism Asn142Asp modifies the risk of age-related cataract in smokers and subjects exposed to ultraviolet irradiation. Clin. Exp. Ophthalmol. 2014, 42, 277-283.
94. Xie, C.M.; Sun, Y. The MTORC1-mediated autophagy is regulated by the FBXW7-SHOC2-RPTOR axis. Autophagy. 2019, 15, 1470-1472.
95. Xiao-Pei, H.; Ji-Kuai, C.; Xue, W.; Dong, Y.F.; Yan, L.; Xiao-Fang, Z.; Ya-Min, P.; Wen-Jun, C.; Jiang-Bo, Z. Systematic identification of Celastrol-binding proteins reveals that Shoc2 is inhibited by Celastrol. Biosci. Rep. 2018, 38, BSR20181233.
96. Kaduwal, S.; Jeong, W.J.; Park, J.C.; Lee, K.H.; Lee, Y.M.; Jeon, S.H.; Lim, Y.B.; Min, d.S.; Choi, K.Y. Sur8/Shoc2 promotes cell motility and metastasis through activation of Ras-PI3K signaling. Oncotarget. 2015, 6, 33091-33105.

97. Leon, G.; Sanchez-Ruiloba, L.; Perez-Rodriguez, A.; Gragera, T.; Martinez, N.; Hernandez, S.; Anta, B.; Calero, O.; Garcia-Dominguez, C.A.; Dura, L.M., et al. Shoc2/Sur8 protein regulates neurite outgrowth. PLoS. One. 2014, 9, e114837.
98. Li, X.; Yao, R.; Tan, X.; Li, N.; Ding, Y.; Li, J.; Chang, G.; Chen, Y.; Ma, L.; Wang, J., et al. Molecular and phenotypic spectrum of Noonan syndrome in Chinese patients. Clin. Genet. 2019, 96, 290-299.
99. Zhou, J.; Leu, N.A.; Eckardt, S.; McLaughlin, K.J.; Wang, P.J. STK31/TDRD8, a germ cell-specific factor, is dispensable for reproduction in mice. PLoS. One. 2014, 9, e89471.
100. Zhu, X.B.; Lu, J.Q.; Zhi, E.L.; Zhu, Y.; Zou, S.S.; Zhu, Z.J.; Zhang, F.; Li, Z. Association of a TDRD1 variant with spermatogenic failure susceptibility in the Han Chinese. J. Assist. Reprod. Genet. 2016, 33, 1099-1104.
101. Thacher, T.D.; Levine, M.A. CYP2R1 mutations causing vitamin D-deficiency rickets. J. Steroid. Biochem. Mol. Biol. 2017, 173, 333-336.
102. Scazzone, C.; Agnello, L.; Ragonese, P.; Lo Sasso, B.; Bellia, C.; Bivona, G.; Schillaci, R.; Salemi, G.; Ciaccio, M. Association of CYP2R1 rs10766197 with MS risk and disease progression. J. Neurosci. Res. 2018, 96, 297-304.
103. Thanapirom, K.; Suksawatamnuay, S.; Sukeepaisarnjareon, W.; Tanwandee, T.; Charatcharoenwitthaya, P.; Thongsawat, S.; Leerapun, A.; Piratvisuth, T.; Boonsirichan, R.; Bunchorntavakul, C., et al. Genetic variation in the vitamin D pathway CYP2R1 gene predicts sustained HBeAg seroconversion in chronic hepatitis B patients treated with pegylated interferon: A multicenter study. PLoS. One. 2017, 12, e0173263.
104. Al-Barry, M.A.; Albalawi, A.M.; Sayf, M.A.; Badawi, A.; Afzal, S.; Latif, M.; Samman, M.I.; Basit, S. Sequence analysis of four vitamin D family genes (VDR, CYP24A1, CYP27B1 and CYP2R1) in Vogt-Koyanagi-Harada (VKH) patients: identification of a potentially pathogenic variant in CYP2R1. BMC. Ophthalmol. 2016, 16, 172.
105. Türkanoğlu Özçelik, A.; Öner, T.; Can Demirdöğen, B.; Bek, V.S.; Demirkaya, Ş.; Adalı, O. Genetic polymorphisms of vitamin D3 metabolizing CYP24A1 and CYP2R1 enzymes in Turkish patients with ischemic stroke. Neurol. Res. 2018, 40, 364-371.
106. Sedky, N.K.; Abdel Rahman, M.F.; Hassanein, S.I.; Gad, M.Z. Genetic Variants of CYP2R1 Are Key Regulators of Serum Vitamin D Levels and Incidence of Myocardial Infarction in Middle-Aged Egyptians. Curr. Pharm. Biotechnol. 2018, 19, 265-273.
107. Wang, Y.; Yu, F.; Yu, S.; Zhang, D.; Wang, J.; Han, H.; Sun, H.; Xue, Y.; Ba, Y.; Wang, C., et al. Triangular relationship between CYP2R1 gene polymorphism, serum 25(OH)D. Gene. 2018, 678, 172-176.
108. Ramos-Lopez, E.; Brück, P.; Jansen, T.; Herwig, J.; Badenhoop, K. CYP2R1 (vitamin D 25-hydroxylase) gene is associated with susceptibility to type 1 diabetes and vitamin D levels in Germans. Diabetes. Metab. Res. Rev. 2007, 23, 631-636.
109. Bergadà, L.; Pallares, J.; Maria Vittoria, A.; Cardus, A.; Santacana, M.; Valls, J.; Cao, G.; Fernàndez, E.; Dolcet, X.; Dusso, A.S., et al. Role of local bioactivation of vitamin D by CYP27A1 and CYP2R1 in the control of cell growth in normal endometrium and endometrial carcinoma. Lab. Invest. 2014, 94, 608-622.
110. Inoue, N.; Watanabe, M.; Ishido, N.; Katsumata, Y.; Kagawa, T.; Hidaka, Y.; Iwatani, Y. The functional polymorphisms of VDR, GC and CYP2R1 are involved in the pathogenesis of autoimmune thyroid diseases. Clin. Exp. Immunol. 2014, 178, 262-269.
111. Zhang, Y.; Wang, Z.; Ma, T. Associations of Genetic Polymorphisms Relevant to Metabolic Pathway of Vitamin D3 with Development and Prognosis of Childhood Bronchial Asthma. DNA. Cell. Biol. 2017, 36, 682-692.
112. Li, J.P.; Liu, Y.; Yin, Y.H. ARHGAP1 overexpression inhibits proliferation, migration and invasion of C-33A and SiHa cell lines. Onco. Targets. Ther. 2017, 10, 691-701.
113. Clay, M.R.; Halloran, M.C. Rho activation is apically restricted by Arhgap1 in neural crest cells and drives epithelial-to-mesenchymal transition. Development. 2013, 140, 3198-3209.
114. Hashimoto, K.; Ochi, H.; Sunamura, S.; Kosaka, N.; Mabuchi, Y.; Fukuda, T.; Yao, K.; Kanda, H.; Ae, K.; Okawa, A., et al. Cancer-secreted hsa-miR-940 induces an osteoblastic phenotype in the bone metastatic microenvironment via targeting ARHGAP1 and FAM134A. Proc. Natl. Acad. Sci. USA. 2018, 115, 2204-2209.
115. Florian, M.C.; Dörr, K.; Niebel, A.; Daria, D.; Schrezenmeier, H.; Rojewski, M.; Filippi, M.D.; Hasenberg, A.; Gunzer, M.; Scharffetter-Kochanek, K., et al. Cdc42 activity regulates hematopoietic stem cell aging and rejuvenation. Cell. Stem. Cell. 2012, 10, 520-530.

116. Wang, L.; Yang, L.; Burns, K.; Kuan, C.Y.; Zheng, Y. Cdc42GAP regulates c-Jun N-terminal kinase (JNK)-mediated apoptosis and cell number during mammalian perinatal growth. Proc. Natl. Acad. Sci. USA. 2005, 102, 13484-13489.
117. Gorski, M.M.; Lecchi, A.; Femia, E.A.; La Marca, S.; Cairo, A.; Pappalardo, E.; Lotta, L.A.; Artoni, A.; Peyvandi, F. Complications of whole-exome sequencing for causal gene discovery in primary platelet secretion defects. Haematologica. 2019, 104, 2084-2090.
118. Marenholz, I.; Esparza-Gordillo, J.; Rüschendorf, F.; Bauerfeind, A.; Strachan, D.P.; Spycher, B.D.; Baurecht, H.; Margaritte-Jeannin, P.; Sääf, A.; Kerkhof, M., et al. Meta-analysis identifies seven susceptibility loci involved in the atopic march. Nat. Commun. 2015, 6, 8804.
119. Zuo, X.; Sun, L.; Yin, X.; Gao, J.; Sheng, Y.; Xu, J.; Zhang, J.; He, C.; Qiu, Y.; Wen, G., et al. Whole-exome SNP array identifies 15 new susceptibility loci for psoriasis. Nat. Commun. 2015, 6, 6793.
120. MacLaren, E.J.; Charlesworth, P.; Coba, M.P.; Grant, S.G. Knockdown of mental disorder susceptibility genes disrupts neuronal network physiology in vitro. Mol. Cell. Neurosci. 2011, 47, 93-99.
121. Shao, Y.W.; Wood, G.A.; Lu, J.; Tang, Q.L.; Liu, J.; Molyneux, S.; Chen, Y.; Fang, H.; Adissu, H.; McKee, T., et al. Cross-species genomics identifies DLG2 as a tumor suppressor in osteosarcoma. Oncogene. 2019, 38, 291-298.
122. Ma, D.; Yang, J.; Wang, Y.; Huang, X.; Du, G.; Zhou, L. Whole exome sequencing identified genetic variations in Chinese hemangioblastoma patients. Am. J. Med. Genet. A. 2017, 173, 2605-2613.
123. Leyland, M.L.; Dart, C. An alternatively spliced isoform of PSD-93/chapsyn 110 binds to the inwardly rectifying potassium channel, Kir2.1. J. Biol. Chem. 2004, 279, 43427-43436.
124. Parker, M.J.; Zhao, S.; Bredt, D.S.; Sanes, J.R.; Feng, G. PSD93 regulates synaptic stability at neuronal cholinergic synapses. J. Neurosci. 2004, 24, 378-388.
125. Chen, C.H.; Wang, Y.; Lo, M.T.; Schork, A.; Fan, C.C.; Holland, D.; Kauppi, K.; Smeland, O.B.; Djurovic, S.; Sanyal, N., et al. Leveraging genome characteristics to improve gene discovery for putamen subcortical brain structure. Sci. Rep. 2017, 7, 15736.
126. Reggiani, C.; Coppens, S.; Sekhara, T.; Dimov, I.; Pichon, B.; Lufin, N.; Addor, M.C.; Belligni, E.F.; Digilio, M.C.; Faletra, F., et al. Novel promoters and coding first exons in DLG2 linked to developmental disorders and intellectual disability. Genome. Med. 2017, 9, 67.
127. Vulto-van Silfhout, A.T.; Hehir-Kwa, J.Y.; van Bon, B.W.; Schuurs-Hoeijmakers, J.H.; Meader, S.; Hellebrekers, C.J.; Thoonen, I.J.; de Brouwer, A.P.; Brunner, H.G.; Webber, C., et al. Clinical significance of de novo and inherited copy-number variation. Hum. Mutat. 2013, 34, 1679-1687.
128. Nithianantharajah, J.; Komiyama, N.H.; McKechanie, A.; Johnstone, M.; Blackwood, D.H.; St Clair, D.; Emes, R.D.; van de Lagemaat, L.N.; Saksida, L.M.; Bussey, T.J., et al. Synaptic scaffold evolution generated components of vertebrate cognitive complexity. Nat. Neurosci. 2013, 16, 16-24.
129. Noor, A.; Lionel, A.C.; Cohen-Woods, S.; Moghimi, N.; Rucker, J.; Fennell, A.; Thiruvahindrapuram, B.; Kaufman, L.; Degagne, B.; Wei, J., et al. Copy number variant study of bipolar disorder in Canadian and UK populations implicates synaptic genes. Am. J. Med. Genet. B. Neuropsychiatr. Genet. 2014, 165B, 303-313.
130. Ruzzo, E.K.; Pérez-Cano, L.; Jung, J.Y.; Wang, L.K.; Kashef-Haghighi, D.; Hartl, C.; Singh, C.; Xu, J.; Hoekstra, J.N.; Leventhal, O., et al. Inherited and De Novo Genetic Risk for Autism Impacts Shared Networks. Cell. 2019, 178, 850-866.e826.
131. Wu, H.C.; Chen, C.M.; Chen, Y.C.; Fung, H.C.; Chang, K.H.; Wu, Y.R. DLG2, but not TMEM229B, GPNMB, and ITGA8 polymorphism, is associated with Parkinson's disease in a Taiwanese population. Neurobiol. Aging. 2018, 64, 158.e151-158.e156.
132. Chen, S.P.; Fuh, J.L.; Chung, M.Y.; Lin, Y.C.; Liao, Y.C.; Wang, Y.F.; Hsu, C.L.; Yang, U.C.; Lin, M.W.; Chiou, J.J., et al. Genome-wide association study identifies novel susceptibility loci for migraine in Han Chinese resided in Taiwan. Cephalalgia. 2018, 38, 466-475.
133. Demirkan, A.; van Duijn, C.M.; Ugocsai, P.; Isaacs, A.; Pramstaller, P.P.; Liebisch, G.; Wilson, J.F.; Johansson, Å.; Rudan, I.; Aulchenko, Y.S., et al. Genome-wide association study identifies novel loci associated with circulating phospho- and sphingolipid concentrations. PLoS. Genet. 2012, 8, e1002490.
134. Palmer, N.D.; Mychaleckyj, J.C.; Langefeld, C.D.; Ziegler, J.T.; Williams, A.H.; Bryer-Ash, M.; Bowden, D.W. Evaluation of DLG2 as a positional candidate for disposition index in African-Americans from the IRAS Family Study. Diabetes. Res. Clin. Pract. 2010, 87, 69-76.

135. Matovinovic, E.; Kho, P.F.; Lea, R.A.; Benton, M.C.; Eccles, D.A.; Haupt, L.M.; Hewitt, A.W.; Sherwin, J.C.; Mackey, D.A.; Griffiths, L.R. Genome-wide linkage and association analysis of primary open-angle glaucoma endophenotypes in the Norfolk Island isolate. Mol. Vis. 2017, 23, 660-665.
136. Chen, W.; Brehm, J.M.; Manichaikul, A.; Cho, M.H.; Boutaoui, N.; Yan, Q.; Burkart, K.M.; Enright, P.L.; Rotter, J.I.; Petersen, H., et al. A genome-wide association study of chronic obstructive pulmonary disease in Hispanics. Ann. Am. Thorac. Soc. 2015, 12, 340-348.
137. Kim, J.Y.; Tillison, K.; Zhou, S.; Wu, Y.; Smas, C.M. The major facilitator superfamily member Slc37a2 is a novel macrophage- specific gene selectively expressed in obese white adipose tissue. Am. J. Physiol. Endocrinol. Metab. 2007, 293, E110-120.
138. Huffman, J.E.; Knezevic, A.; Vitart, V.; Kattla, J.; Adamczyk, B.; Novokmet, M.; Igl, W.; Pucic, M.; Zgaga, L.; Johannson, Å., et al. Polymorphisms in B3GAT1, SLC9A9 and MGAT5 are associated with variation within the human plasma N-glycome of 3533 European adults. Hum. Mol. Genet. 2011, 20, 5000-5011.
139. Camargo, M.; Rivera, D.; Moreno, L.; Lidral, A.C.; Harper, U.; Jones, M.; Solomon, B.D.; Roessler, E.; Vélez, J.I.; Martinez, A.F., et al. GWAS reveals new recessive loci associated with non-syndromic facial clefting. Eur. J. Med. Genet. 2012, 55, 510-514.
140. Jeffries, A.R.; Mungall, A.J.; Dawson, E.; Halls, K.; Langford, C.F.; Murray, R.M.; Dunham, I.; Powell, J.F. beta-1,3-Glucuronyltransferase-1 gene implicated as a candidate for a schizophrenia-like psychosis through molecular analysis of a balanced translocation. Mol. Psychiatry. 2003, 8, 654-663.
141. Nabeshima, R.; Nishimura, O.; Maeda, T.; Shimizu, N.; Ide, T.; Yashiro, K.; Sakai, Y.; Meno, C.; Kadota, M.; Shiratori, H., et al. Loss of Fam60a, a Sin3a subunit, results in embryonic lethality and is associated with aberrant methylation at a subset of gene promoters. Elife. 2018, 7, e36435.
142. Muñoz, I.M.; MacArtney, T.; Sanchez-Pulido, L.; Ponting, C.P.; Rocha, S.; Rouse, J. Family with sequence similarity 60A (FAM60A) protein is a cell cycle-fluctuating regulator of the SIN3-HDAC1 histone deacetylase complex. J. Biol. Chem. 2012, 287, 32346-32353.
143. Dong, G.; Mao, Q.; Yu, D.; Zhang, Y.; Qiu, M.; Chen, Q.; Xia, W.; Wang, J.; Xu, L.; Jiang, F. Integrative analysis of copy number and transcriptional expression profiles in esophageal cancer to identify a novel driver gene for therapy. Sci. Rep. 2017, 7, 42060.
144. Imamura, M.; Takahashi, A.; Yamauchi, T.; Hara, K.; Yasuda, K.; Grarup, N.; Zhao, W.; Wang, X.; Huerta-Chagoya, A.; Hu, C., et al. Genome-wide association studies in the Japanese population identify seven novel loci for type 2 diabetes. Nat. Commun. 2016, 7, 10531.
145. Yousif, A.S.; Stanlie, A.; Begum, N.A.; Honjo, T. Opinion: uracil DNA glycosylase (UNG) plays distinct and non-canonical roles in somatic hypermutation and class switch recombination. Int. Immunol. 2014, 26, 575-578.
146. Yan, Y.; Han, X.; Qing, Y.; Condie, A.G.; Gorityala, S.; Yang, S.; Xu, Y.; Zhang, Y.; Gerson, S.L. Inhibition of uracil DNA glycosylase sensitizes cancer cells to 5-fluorodeoxyuridine through replication fork collapse-induced DNA damage. Oncotarget. 2016, 7, 59299-59313.
147. Zappasodi, R.; Ruggiero, G.; Guarnotta, C.; Tortoreto, M.; Tringali, C.; Cavanè, A.; Cabras, A.D.; Castagnoli, L.; Venerando, B.; Zaffaroni, N., et al. HSPH1 inhibition downregulates Bcl-6 and c-Myc and hampers the growth of human aggressive B-cell non-Hodgkin lymphoma. Blood. 2015, 125, 1768-1771.
148. Hosaka, S.; Nakatsura, T.; Tsukamoto, H.; Hatayama, T.; Baba, H.; Nishimura, Y. Synthetic small interfering RNA targeting heat shock protein 105 induces apoptosis of various cancer cells both in vitro and in vivo. Cancer. Sci. 2006, 97, 623-632.
149. Colgan, S.P.; Pitman, R.S.; Nagaishi, T.; Mizoguchi, A.; Mizoguchi, E.; Mayer, L.F.; Shao, L.; Sartor, R.B.; Subjeck, J.R.; Blumberg, R.S. Intestinal heat shock protein 110 regulates expression of CD1d on intestinal epithelial cells. J. Clin. Invest. 2003, 112, 745-754.
150. Al-Hamed, M.H.; Imtiaz, F.; Al-Hassnan, Z.; Al-Owain, M.; Al-Zaidan, H.; Alamoudi, M.S.; Faqeih, E.; Alfadhel, M.; Al-Asmari, A.; Saleh, M.M., et al. Spectrum of mutations underlying Propionic acidemia and further insight into a genotype-phenotype correlation for the common mutation in Saudi Arabia. Mol. Genet. Metab. Rep. 2019, 18, 22-29.
151. Huang, Q.; Lin, W.; Lin, J.; Lin, X. Potential roles for PA28beta in gastric adenocarcinoma development and diagnosis. J. Cancer. Res. Clin. Oncol. 2010, 136, 1275-1282.
152. Harz, C.; Ludwig, N.; Lang, S.; Werner, T.V.; Galata, V.; Backes, C.; Schmitt, K.; Nickels, R.; Krause, E.; Jung, M., et al. Secretion and immunogenicity of the meningioma-associated antigen TXNDC16. J. Immunol. 2014, 193, 3146-3154.

153. Chujo, T.; Suzuki, T. Trmt61B is a methyltransferase responsible for 1-methyladenosine at position 58 of human mitochondrial tRNAs. RNA. 2012, 18, 2269-2276.
154. Sahid, M.N.A.; Liu, S.; Kiyoi, T.; Maeyama, K. Inhibition of the mevalonate pathway by simvastatin interferes with mast cell degranulation by disrupting the interaction between Rab27a and double C2 alpha proteins. Eur. J. Pharmacol. 2017, 814, 255-263.
155. Li, J.; Cantley, J.; Burchfield, J.G.; Meoli, C.C.; Stöckli, J.; Whitworth, P.T.; Pant, H.; Chaudhuri, R.; Groffen, A.J.; Verhage, M., et al. DOC2 isoforms play dual roles in insulin secretion and insulin-stimulated glucose uptake. Diabetologia. 2014, 57, 2173-2182.
156. McCammon, J.M.; Blaker-Lee, A.; Chen, X.; Sive, H. The 16p11.2 homologs fam57ba and doc2a generate certain brain and body phenotypes. Hum. Mol. Genet. 2017, 26, 3699-3712.
157. Glessner, J.T.; Reilly, M.P.; Kim, C.E.; Takahashi, N.; Albano, A.; Hou, C.; Bradfield, J.P.; Zhang, H.; Sleiman, P.M.; Flory, J.H., et al. Strong synaptic transmission impact by copy number variations in schizophrenia. Proc. Natl. Acad. Sci. USA. 2010, 107, 10584-10589.
158. Mardakheh, F.K.; Self, A.; Marshall, C.J. RHO binding to FAM65A regulates Golgi reorientation during cell migration. J. Cell. Sci. 2016, 129, 4466-4479.
159. Litchfield, K.; Levy, M.; Dudakia, D.; Proszek, P.; Shipley, C.; Basten, S.; Rapley, E.; Bishop, D.T.; Reid, A.; Huddart, R., et al. Rare disruptive mutations in ciliary function genes contribute to testicular cancer susceptibility. Nat. Commun. 2016, 7, 13840.
160. Hartill, V.L.; van de Hoek, G.; Patel, M.P.; Little, R.; Watson, C.M.; Berry, I.R.; Shoemark, A.; Abdelmottaleb, D.; Parkes, E.; Bacchelli, C., et al. DNAAF1 links heart laterality with the AAA+ ATPase RUVBL1 and ciliary intraflagellar transport. Hum. Mol. Genet. 2018, 27, 529-545.
161. Huang, H.L.; Jiang, Y.; Wang, Y.H.; Chen, T.; He, H.J.; Liu, T.; Yang, T.; Yang, L.W.; Chen, J.; Song, Z.Q., et al. FBXO31 promotes cell proliferation, metastasis and invasion in lung cancer. Am. J. Cancer. Res. 2015, 5, 1814-1822.
162. Jeffery, J.M.; Kalimutho, M.; Johansson, P.; Cardenas, D.G.; Kumar, R.; Khanna, K.K. FBXO31 protects against genomic instability by capping FOXM1 levels at the G2/M transition. Oncogene. 2017, 36, 1012-1022.
163. Santra, M.K.; Wajapeyee, N.; Green, M.R. F-box protein FBXO31 mediates cyclin D1 degradation to induce G1 arrest after DNA damage. Nature. 2009, 459, 722-725.
164. Li, Y.; Jin, K.; Bunker, E.; Zhang, X.; Luo, X.; Liu, X.; Hao, B. Structural basis of the phosphorylation-independent recognition of cyclin D1 by the SCF. Proc. Natl. Acad. Sci. USA. 2018, 115, 319-324.
165. Vadhvani, M.; Schwedhelm-Domeyer, N.; Mukherjee, C.; Stegmüller, J. The centrosomal E3 ubiquitin ligase FBXO31-SCF regulates neuronal morphogenesis and migration. PLoS. One. 2013, 8, e57530.
166. Gallagher, D.; Voronova, A.; Zander, M.A.; Cancino, G.I.; Bramall, A.; Krause, M.P.; Abad, C.; Tekin, M.; Neilsen, P.M.; Callen, D.F., et al. Ankrd11 is a chromatin regulator involved in autism that is essential for neural development. Dev. Cell. 2015, 32, 31-42.
167. Ka, M.; Kim, W.Y. ANKRD11 associated with intellectual disability and autism regulates dendrite differentiation via the BDNF/TrkB signaling pathway. Neurobiol. Dis. 2018, 111, 138-152.
168. Walz, K.; Cohen, D.; Neilsen, P.M.; Foster, J.; Brancati, F.; Demir, K.; Fisher, R.; Moffat, M.; Verbeek, N.E.; Bjørgo, K., et al. Characterization of ANKRD11 mutations in humans and mice related to KBG syndrome. Hum. Genet. 2015, 134, 181-190.
169. Neilsen, P.M.; Cheney, K.M.; Li, C.W.; Chen, J.D.; Cawrse, J.E.; Schulz, R.B.; Powell, J.A.; Kumar, R.; Callen, D.F. Identification of ANKRD11 as a p53 coactivator. J. Cell. Sci. 2008, 121, 3541-3552.
170. Noll, J.E.; Jeffery, J.; Al-Ejeh, F.; Kumar, R.; Khanna, K.K.; Callen, D.F.; Neilsen, P.M. Mutant p53 drives multinucleation and invasion through a process that is suppressed by ANKRD11. Oncogene. 2012, 31, 2836-2848.
171. Barbaric, I.; Perry, M.J.; Dear, T.N.; Rodrigues Da Costa, A.; Salopek, D.; Marusic, A.; Hough, T.; Wells, S.; Hunter, A.J.; Cheeseman, M., et al. An ENU-induced mutation in the Ankrd11 gene results in an osteopenia-like phenotype in the mouse mutant Yoda. Physiol. Genomics. 2008, 32, 311-321.
172. Pu, Y.; Zhao, F.; Cai, W.; Meng, X.; Li, Y.; Cai, S. MiR-193a-3p and miR-193a-5p suppress the metastasis of human osteosarcoma cells by down-regulating Rab27B and SRR, respectively. Clin. Exp. Metastasis. 2016, 33, 359-372.

173. Wu, M.; Liu, Y.; Zhang, H.; Lian, M.; Chen, J.; Jiang, H.; Xu, Y.; Shan, G.; Wu, S. Intravenous injection of l-aspartic acid β-hydroxamate attenuates choroidal neovascularization via anti-VEGF and anti-inflammation. Exp. Eye. Res. 2019, 182, 93-100.
174. Perez, E.J.; Tapanes, S.A.; Loris, Z.B.; Balu, D.T.; Sick, T.J.; Coyle, J.T.; Liebl, D.J. Enhanced astrocytic d-serine underlies synaptic damage after traumatic brain injury. J. Clin. Invest. 2017, 127, 3114-3125.
175. Zhang, H.; Song, L.; Chang, Y.; Wu, M.; Kuang, X.; Jiang, H.; Wu, S. Potential deficit from decreased cerebellar granule cell migration in serine racemase-deficient mice is reversed by increased expression of GluN2B and elevated levels of NMDAR agonists. Mol. Cell. Neurosci. 2017, 85, 119-126.
176. Mori, H.; Wada, R.; Takahara, S.; Horino, Y.; Izumi, H.; Ishimoto, T.; Yoshida, T.; Mizuguchi, M.; Obita, T.; Gouda, H., et al. A novel serine racemase inhibitor suppresses neuronal over-activation in vivo. Bioorg. Med. Chem. 2017, 25, 3736-3745.
177. Benneyworth, M.A.; Li, Y.; Basu, A.C.; Bolshakov, V.Y.; Coyle, J.T. Cell selective conditional null mutations of serine racemase demonstrate a predominate localization in cortical glutamatergic neurons. Cell. Mol. Neurobiol. 2012, 32, 613-624.
178. Dai, X.; Zhou, E.; Yang, W.; Zhang, X.; Zhang, W.; Rao, Y. D-Serine made by serine racemase in Drosophila intestine plays a physiological role in sleep. Nat. Commun. 2019, 10, 1986.
179. Balu, D.T.; Presti, K.T.; Huang, C.C.Y.; Muszynski, K.; Radzishevsky, I.; Wolosker, H.; Guffanti, G.; Ressler, K.J.; Coyle, J.T. Serine Racemase and D-serine in the Amygdala Are Dynamically Involved in Fear Learning. Biol. Psychiatry. 2018, 83, 273-283.
180. Matveeva, T.M.; Pisansky, M.T.; Young, A.; Miller, R.F.; Gewirtz, J.C. Sociality deficits in serine racemase knockout mice. Brain. Behav. 2019, 9, e01383.
181. Puhl, M.D.; Desai, R.I.; Takagi, S.; Presti, K.T.; Doyle, M.R.; Donahue, R.J.; Landino, S.M.; Bergman, J.; Carlezon, W.A.; Coyle, J.T. N-Methyl-d-aspartate receptor co-agonist availability affects behavioral and neurochemical responses to cocaine: insights into comorbid schizophrenia and substance abuse. Addict. Biol. 2019, 24, 40-50.
182. Dong, C.; Zhang, J.C.; Ren, Q.; Ma, M.; Qu, Y.; Zhang, K.; Yao, W.; Ishima, T.; Mori, H.; Hashimoto, K. Deletion of serine racemase confers D-serine -dependent resilience to chronic social defeat stress. Neurochem. Int. 2018, 116, 43-51.
183. Van der Auwera, S.; Teumer, A.; Hertel, J.; Homuth, G.; Völker, U.; Lucht, M.J.; Degenhardt, F.; Schulze, T.; Rietschel, M.; Nöthen, M.M., et al. The inverse link between genetic risk for schizophrenia and migraine through NMDA (N-methyl-D-aspartate) receptor activation via D-serine. Eur. Neuropsychopharmacol. 2016, 26, 1507-1515.
184. Moon, J.Y.; Choi, S.R.; Roh, D.H.; Yoon, S.Y.; Kwon, S.G.; Choi, H.S.; Kang, S.Y.; Han, H.J.; Kim, H.W.; Beitz, A.J., et al. Spinal sigma-1 receptor activation increases the production of D-serine in astrocytes which contributes to the development of mechanical allodynia in a mouse model of neuropathic pain. Pharmacol. Res. 2015, 100, 353-364.
185. Girard, H.; Potvin, O.; Nugent, S.; Dallaire-Théroux, C.; Cunnane, S.; Duchesne, S.; Initiative, A.s.D.N. Faster progression from MCI to probable AD for carriers of a single-nucleotide polymorphism associated with type 2 diabetes. Neurobiol. Aging. 2018, 64, 157.e111-157.e117.
186. Lockridge, A.D.; Baumann, D.C.; Akhaphong, B.; Abrenica, A.; Miller, R.F.; Alejandro, E.U. Serine racemase is expressed in islets and contributes to the regulation of glucose homeostasis. Islets. 2016, 8, 195-206.
187. Jiang, H.; Du, J.; Song, J.; Li, Y.; Wu, M.; Zhou, J.; Wu, S. Loss-of-function mutation of serine racemase attenuates retinal ganglion cell loss in diabetic mice. Exp. Eye. Res. 2018, 175, 90-97.
188. Jiang, Y.J.; Zhong, J.H.; Zhou, Z.H.; Qiu, M.Q.; Zhou, X.G.; Liu, Y.C.; Huo, R.R.; Liang, X.M.; Chen, Z.; Lin, Q.L., et al. Association between polymorphisms in MicroRNA target sites of RAD51D genes and risk of hepatocellular carcinoma. Cancer. Med. 2019, 8, 2545-2552.
189. Rajesh, P.; Litvinchuk, A.V.; Pittman, D.L.; Wyatt, M.D. The homologous recombination protein RAD51D mediates the processing of 6-thioguanine lesions downstream of mismatch repair. Mol. Cancer. Res. 2011, 9, 206-214.
190. Sovio, U.; Bennett, A.J.; Millwood, I.Y.; Molitor, J.; O'Reilly, P.F.; Timpson, N.J.; Kaakinen, M.; Laitinen, J.; Haukka, J.; Pillas, D., et al. Genetic determinants of height growth assessed longitudinally from infancy to adulthood in the northern Finland birth cohort 1966. PLoS. Genet. 2009, 5, e1000409.

191. Mao, S.; Xu, L.; Zhu, Z.; Qian, B.; Qiao, J.; Yi, L.; Qiu, Y. Association between genetic determinants of peak height velocity during puberty and predisposition to adolescent idiopathic scoliosis. Spine (Phila Pa 1976). 2013, 38, 1034-1039.
192. Tateossian, H.; Morse, S.; Parker, A.; Mburu, P.; Warr, N.; Acevedo-Arozena, A.; Cheeseman, M.; Wells, S.; Brown, S.D. Otitis media in the Tgif knockout mouse implicates TGFβ signalling in chronic middle ear inflammatory disease. Hum. Mol. Genet. 2013, 22, 2553-2565.
193. Guca, E.; Suñol, D.; Ruiz, L.; Konkol, A.; Cordero, J.; Torner, C.; Aragon, E.; Martin-Malpartida, P.; Riera, A.; Macias, M.J. TGIF1 homeodomain interacts with Smad MH1 domain and represses TGF-β signaling. Nucleic. Acids. Res. 2018, 46, 9220-9235.
194. Xiang, G.; Yi, Y.; Weiwei, H.; Weiming, W. TGIF1 promoted the growth and migration of cancer cells in nonsmall cell lung cancer. Tumour. Biol. 2015, 36, 9303-9310.
195. Liu, Z.M.; Tseng, H.Y.; Tsai, H.W.; Su, F.C.; Huang, H.S. Transforming growth factor β-interacting factor-induced malignant progression of hepatocellular carcinoma cells depends on superoxide production from Nox4. Free. Radic. Biol. Med. 2015, 84, 54-64.
196. Gunatillake, T.; Yong, H.E.; Dunk, C.E.; Keogh, R.J.; Borg, A.J.; Cartwright, J.E.; Whitley, G.S.; Murthi, P. Homeobox gene TGIF-1 is increased in placental endothelial cells of human fetal growth restriction. Reproduction. 2016, 152, 457-465.
197. Saito, H.; Gasser, A.; Bolamperti, S.; Maeda, M.; Matthies, L.; Jähn, K.; Long, C.L.; Schlüter, H.; Kwiatkowski, M.; Saini, V., et al. TG-interacting factor 1 (Tgif1)-deficiency attenuates bone remodeling and blunts the anabolic response to parathyroid hormone. Nat. Commun. 2019, 10, 1354.
198. Chen, L.; Jiang, C.; Tiwari, S.R.; Shrestha, A.; Xu, P.; Liang, W.; Sun, Y.; He, S.; Cheng, B. TGIF1 Gene Silencing in Tendon-Derived Stem Cells Improves the Tendon-to-Bone Insertion Site Regeneration. Cell. Physiol. Biochem. 2015, 37, 2101-2114.
199. Sharma, A.; Sinha, N.R.; Siddiqui, S.; Mohan, R.R. Role of 5'TG3'-interacting factors (TGIFs) in Vorinostat (HDAC inhibitor)-mediated Corneal Fibrosis Inhibition. Mol. Vis. 2015, 21, 974-984.
200. Härdfeldt, J.; Hodson, L.; Larsson, L.; Pedrelli, M.; Pramfalk, C. Effects on hepatic lipid metabolism in human hepatoma cells following overexpression of TGFβ induced factor homeobox 1 or 2. Biochim. Biophys. Acta. Mol. Cell. Biol. Lipids. 2019, 1864, 756-762.
201. Parini, P.; Melhuish, T.A.; Wotton, D.; Larsson, L.; Ahmed, O.; Eriksson, M.; Pramfalk, C. Overexpression of transforming growth factor β induced factor homeobox 1 represses NPC1L1 and lowers markers of intestinal cholesterol absorption. Atherosclerosis. 2018, 275, 246-255.
202. Shah, A.; Melhuish, T.A.; Fox, T.E.; Frierson, H.F.; Wotton, D. TGIF transcription factors repress acetyl CoA metabolic gene expression and promote intestinal tumor growth. Genes. Dev. 2019, 33, 388-402.
203. Zhu, J.; Li, S.; Ramelot, T.A.; Kennedy, M.A.; Liu, M.; Yang, Y. Structural insights into the impact of two holoprosencephaly-related mutations on human TGIF1 homeodomain. Biochem. Biophys. Res. Commun. 2018, 496, 575-581.
204. Verrotti, A.; Palka, C.; Prezioso, G.; Alfonsi, M.; Calabrese, G.; Palka, G.; Chiarelli, F. Deletion 18p11.32p11.31 in a Child with Global Developmental Delay and Atypical, Drug-Resistant Absence Seizures. Cytogenet. Genome. Res. 2015, 146, 115-119.
205. Eadon, M.T.; Hause, R.J.; Stark, A.L.; Cheng, Y.H.; Wheeler, H.E.; Burgess, K.S.; Benson, E.A.; Cunningham, P.N.; Bacallao, R.L.; Dagher, P.C., et al. Genetic Variants Contributing to Colistin Cytotoxicity: Identification of TGIF1 and HOXD10 Using a Population Genomics Approach. Int. J. Mol. Sci. 2017, 18, E661.
206. Ahmed, I.; Rasool, S.; Jan, T.; Qureshi, T.; Naykoo, N.A.; Andrabi, K.I. TGIF1 is a potential candidate gene for high myopia in ethnic Kashmiri population. Curr. Eye. Res. 2014, 39, 282-290.
207. Xu, P.; Ma, J.; Zhang, W.; Guo, S.; Jian, Z.; Liu, L.; Wang, G.; Gao, T.; Zhu, G.; Li, C. Multiple pro-tumorigenic functions of the human minor Histocompatibility Antigen-1 (HA-1) in melanoma progression. J. Dermatol. Sci. 2017, 88, 216-224.
208. Amado-Azevedo, J.; Reinhard, N.R.; van Bezu, J.; van Nieuw Amerongen, G.P.; van Hinsbergh, V.W.M.; Hordijk, P.L. The minor histocompatibility antigen 1 (HMHA1)/ArhGAP45 is a RacGAP and a novel regulator of endothelial integrity. Vascul. Pharmacol. 2018, 101, 38-47.
209. Lai, W.S.; Ding, Y.L. GNG7 silencing promotes the proliferation and differentiation of placental cytotrophoblasts in preeclampsia rats through activation of the mTOR signaling pathway. Int. J. Mol. Med. 2019, 43, 1939-1950.

210. Liu, J.; Ji, X.; Li, Z.; Yang, X.; Wang, W.; Zhang, X. G protein γ subunit 7 induces autophagy and inhibits cell division. Oncotarget. 2016, 7, 24832-24847.
211. Guan, M.; Keaton, J.M.; Dimitrov, L.; Hicks, P.J.; Xu, J.; Palmer, N.D.; Ma, L.; Das, S.K.; Chen, Y.I.; Coresh, J., et al. Genome-wide association study identifies novel loci for type 2 diabetes-attributed end-stage kidney disease in African Americans. Hum. Genomics. 2019, 13, 21.
212. Hsieh, C.S.; Huang, P.S.; Chang, S.N.; Wu, C.K.; Hwang, J.J.; Chuang, E.Y.; Tsai, C.T. Genome-Wide Copy Number Variation Association Study of Atrial Fibrillation Related Thromboembolic Stroke. J. Clin. Med. 2019, 8, E332.
213. Schol-Gelok, S.; Janssens, A.C.; Tiemeier, H.; Liu, F.; Lopez-Leon, S.; Zorkoltseva, I.V.; Axenovich, T.I.; van Swieten, J.C.; Uitterlinden, A.G.; Hofman, A., et al. A genome-wide screen for depression in two independent Dutch populations. Biol. Psychiatry. 2010, 68, 187-196.
214. Yin, Y.; Yong, W.; Yu, J.; Zhang, X.; Lin, H.; Zhu, Y.; Han, X. Pdcd2l Promotes Palmitate-Induced Pancreatic Beta-Cell Apoptosis as a FoxO1 Target Gene. PLoS. One. 2016, 11, e0166692.
215. Yazdani, A.; Elsea, S.H.; Schaid, D.J.; Kosorok, M.R.; Dangol, G.; Samiei, A. Genome analysis and pleiotropy assessment using causal networks with loss of function mutation and metabolomics. BMC. Genomics. 2019, 20, 395.
216. Au, F.K.; Jia, Y.; Jiang, K.; Grigoriev, I.; Hau, B.K.; Shen, Y.; Du, S.; Akhmanova, A.; Qi, R.Z. GAS2L1 Is a Centriole-Associated Protein Required for Centrosome Dynamics and Disjunction. Dev. Cell. 2017, 40, 81-94.
217. Mancini, A.; Niemann-Seyde, S.C.; Pankow, R.; El Bounkari, O.; Klebba-Färber, S.; Koch, A.; Jaworska, E.; Spooncer, E.; Gruber, A.D.; Whetton, A.D., et al. THOC5/FMIP, an mRNA export TREX complex protein, is essential for hematopoietic primitive cell survival in vivo. BMC. Biol. 2010, 8, 1.
218. Saran, S.; Tran, D.D.; Ewald, F.; Koch, A.; Hoffmann, A.; Koch, M.; Nashan, B.; Tamura, T. Depletion of three combined THOC5 mRNA export protein target genes synergistically induces human hepatocellular carcinoma cell death. Oncogene. 2016, 35, 3872-3879.
219. Yuan, X.; Zhang, T.; Yao, F.; Liao, Y.; Liu, F.; Ren, Z.; Han, L.; Diao, L.; Li, Y.; Zhou, B., et al. THO Complex-Dependent Posttranscriptional Control Contributes to Vascular Smooth Muscle Cell Fate Decision. Circ. Res. 2018, 123, 538-549.
220. Maeder, C.I.; Kim, J.I.; Liang, X.; Kaganovsky, K.; Shen, A.; Li, Q.; Li, Z.; Wang, S.; Xu, X.Z.S.; Li, J.B., et al. The THO Complex Coordinates Transcripts for Synapse Development and Dopamine Neuron Survival. Cell. 2018, 174, 1436-1449.e1420.
221. Loja-Chango, R.; Salazar-Pousada, D.; Escobar-Valdivieso, G.S.; Ramírez-Morán, C.; Espinoza-Caicedo, J.; Pérez-López, F.R.; Gavilanes, A.W.D.; Chedraui, P. Polymorphism of the THOC5 of the transcription/export multiprotein complex and its correlation with the lipid and metabolic profile in middle-aged women. Gynecol. Endocrinol. 2020, 36, 243-246.
222. Keller, M.; Schleinitz, D.; Förster, J.; Tönjes, A.; Böttcher, Y.; Fischer-Rosinsky, A.; Breitfeld, J.; Weidle, K.; Rayner, N.W.; Burkhardt, R., et al. THOC5: a novel gene involved in HDL-cholesterol metabolism. J. Lipid. Res. 2013, 54, 3170-3176.

Table 3. Uric acid-associated loci with genome-wide significance ($P < 5{\times}10^{-8}$) identified in the past literature.

SNP	Chr	Position[a]	*Gene*	Reference
rs9728345	**1**	145597417	*POLR3C*	[1]
rs11587821	1	145599038	*POLR3C*	[1]
rs10752826	1	145602791	*POLR3C*	[1]
rs4970859	1	145605318	*POLR3C*	[1]
rs12402867	1	145636226	*RNF115*	[1]
rs12724816	1	145639324	*RNF115*	[1]

rs12405132	1	145644984	*RNF115*	[1]
rs2040086	1	145651876	*RNF115*	[1]
rs2318299	1	145662666	*RNF115*	[1]
rs11591191	1	145675931	*RNF115*	[1]
rs12750384	1	145681484	*RNF115*	[1]
rs17354678	1	145689271	*RNF115*	[1]
rs12123298	1	145690472	*RNF115*	[1]
rs17352469	1	145693383	*3′ of CD160*	[1]
rs2231375	1	145696694	*CD160*	[1]
rs1023945	1	145703115	*CD160*	[1]
rs1471628	1	145707057	*CD160*	[1]
rs4970874	1	145709394	*CD160*	[1]
rs744877	1	145714376	*CD160*	[1]
rs9728526	1	145716763	*5′ of CD160*	[1]
rs3753436	1	145718113	*5′ of CD160*	[1]
rs10910845	1	145723120	*5′ of PDZK1*	[1]
rs1967017	1	145723645	*5′ of PDZK1*	[2]
rs1471633	1	145723739	*5′ of PDZK1*	[1]
rs12129861	1	145725689	*5′ of PDZK1*	[3]
rs900347	1	145726727	*5′ of PDZK1*	[1]
rs1298954	1	145730160	*PDZK1*	[1]
rs9659930	1	145730701	*PDZK1*	[1]
rs882210	1	145732946	*PDZK1*	[1]
rs9728619	1	145736438	*PDZK1*	[1]
rs4971059	1	155148781	*KRTCAP2*	[1]
rs3814316	1	155149718	*KRTCAP2*	[1]
rs11264341	1	155151493	*KRTCAP2*	[1]
rs9426886	1	155151754	*KRTCAP2*	[1]
rs4971100	1	155155731	*KRTCAP2*	[1]
rs4072037	1	155162067	*MUC1*	[1]
rs2990245	1	155197462	*3′ of GBA*	[1]
rs189129662	1	201047231	*CACNA1S*	[4]
rs12734001	1	202390914	*PPP1R12B*	[5]

rs1395	2	27424636	*SLC5A6*	[1]
rs13404327	2	27519153	*TRIM54*	[1]
rs13404446	2	27519254	*TRIM54*	[1]
rs4665963	2	27528692	*TRIM54*	[1]
rs4665965	2	27536380	*MPV17*	[1]
rs1049817	2	27550967	*GTF3C2*	[1]
rs3739095	2	27556721	*GTF3C2*	[1]
rs11684134	2	27558252	*GTF3C2*	[1]
rs11689803	2	27566520	*GTF3C2*	[1]
rs6743819	2	27567407	*GTF3C2*	[1]
rs10205219	2	27568565	*GTF3C2*	[1]
rs4665969	2	27574953	*GTF3C2*	[1]
rs6760828	2	27579231	*AC074117.10*	[1]
rs7586601	2	27584666	*AC074117.10*	[1]
rs2280737	2	27589810	*AC074117.10*	[1]
rs7602534	2	27592423	*EIF2B4*	[1]
rs1528533	2	27595756	*SNX17*	[1]
rs13472	2	27600239	*ZNF513*	[1]
rs4582	2	27604279	*PPM1G*	[1]
rs2384629	2	27606098	*PPM1G*	[1]
rs1647284	2	27608115	*PPM1G*	[1]
rs7594812	2	27611469	*PPM1G*	[1]
rs12476704	2	27613031	*PPM1G*	[1]
rs2911712	2	27626945	*PPM1G*	[1]
rs7563162	2	27631191	*PPM1G*	[1]
rs1728918	2	27635463	*5′ of PPM1G*	[6]
rs1060525	2	27635582	*5′ of PPM1G*	[1]
rs2010087	2	27637235	*5′ of PPM1G*	[1]
rs4665976	2	27640325	*5′ of PPM1G*	[1]
rs11675428	2	27642734	*5′ of NRBP1*	[1]
rs1728922	2	27644464	*5′ of NRBP1*	[1]
rs6547626	2	27646770	*5′ of NRBP1*	[1]
rs4665978	2	27648726	*5′ of NRBP1*	[1]

rs780100	2	27652153	*NRBP1*	[1]
rs704791	2	27657167	*NRBP1*	[1]
rs780102	2	27659491	*NRBP1*	[1]
rs1260341	2	27663215	*NRBP1*	[1]
rs1260342	2	27663416	*NRBP1*	[1]
rs4803	2	27667297	*KRTCAP3*	[1]
rs780104	2	27677691	*IFT172*	[1]
rs780106	2	27681598	*IFT172*	[1]
rs780107	2	27684734	*IFT172*	[1]
rs780110	2	27685388	*IFT172*	[1]
rs1647276	2	27688601	*IFT172*	[1]
rs1647266	2	27693485	*IFT172*	[1]
rs780117	2	27698343	*IFT172*	[1]
rs1260345	2	27703495	*IFT172*	[1]
rs2272417	2	27706640	*IFT172*	[1]
rs8395	2	27715207	*FNDC4*	[1]
rs2303369	2	27715416	*FNDC4*	[1]
rs704795	2	27716494	*FNDC4*	[1]
rs780090	2	27718474	*5′ of FNDC4*	[1]
rs813592	2	27721971	*GCKR*	[1]
rs1260320	2	27722416	*GCKR*	[1]
rs2293572	2	27728777	*GCKR*	[1]
rs2293571	2	27729480	*GCKR*	[1]
rs1260326	2	27730940	*GCKR*	[1]
rs3817588	2	27731212	*GCKR*	[1]
rs4425043	2	27733452	*GCKR*	[1]
rs780094	2	27741237	*GCKR*	[1]
rs780093	2	27742603	*GCKR*	[7]
rs780092	2	27743154	*GCKR*	[1]
rs814295	2	27743215	*GCKR*	[1]
rs11681351	2	27743423	*GCKR*	[1]
rs8179252	2	27746832	*3′ of GCKR*	[1]
rs1260333	2	27748624	*3′ of GCKR*	[1]

rs2911711	2	27750546	*3′ of GCKR*	[1]
rs4665987	2	27755825	*5′ of AC109829.1*	[1]
rs4665991	2	27766284	*AC109829.1*	[1]
rs4665382	2	27783801	*AC109829.1*	[1]
rs10208529	2	27786188	*AC109829.1*	[1]
rs4665383	2	27791555	*3′ of AC109829.1*	[1]
rs1919127	2	27801493	*C2orf16*	[1]
rs1919128	2	27801759	*C2orf16*	[1]
rs12478841	2	27811722	*ZNF512*	[1]
rs6760250	2	27812252	*ZNF512*	[1]
rs13022873	2	27815510	*ZNF512*	[1]
rs12467476	2	27825715	*ZNF512*	[1]
rs2384656	2	27832055	*ZNF512*	[1]
rs4666000	2	27839369	*ZNF512*	[1]
rs2068834	2	27839539	*ZNF512*	[1]
rs4666002	2	27840640	*ZNF512*	[1]
rs3749147	2	27851918	*ZNF512*	[1]
rs13002853	2	27853245	*ZNF512*	[1]
rs2272406	2	27892023	*SLC4A1AP*	[1]
rs2178198	2	27895073	*SLC4A1AP*	[1]
rs13023094	2	27910706	*SLC4A1AP*	[1]
rs13030973	2	27928797	*AC074091.13*	[1]
rs6727388	2	27932587	*AC074091.13*	[1]
rs4616435	2	27933642	*AC074091.13*	[1]
rs6727215	2	27934731	*AC074091.13*	[1]
rs13023194	2	27967260	*5′ of MRPL33*	[1]
rs12104449	2	27972833	*5′ of MRPL33*	[1]
rs13030345	2	28003174	*MRPL33*	[1]
rs4401177	2	28344285	*BRE*	[1]
rs17709034	2	28424472	*BRE*	[1]
rs7349418	2	28443050	*BRE*	[1]
rs2311597	2	121305771	*5′ of AC073257.2*	[1]
rs17050272	2	121306440	*5′ of AC073257.2*	[1]

rs2030746	2	121309488	*3′ of AC073257.1*	[1]
rs6706968	2	121310269	*3′ of AC073257.1*	[1]
rs1424949	2	148542963	*5′ of ACVR2A*	[1]
rs12479193	2	148557911	*5′ of ACVR2A*	[1]
rs12472058	2	148558041	*5′ of ACVR2A*	[1]
rs4972326	2	148558848	*5′ of ACVR2A*	[1]
rs7605029	2	148559921	*5′ of ACVR2A*	[1]
rs986508	2	148562031	*5′ of ACVR2A*	[1]
rs13032660	2	148562909	*5′ of ACVR2A*	[1]
rs1863153	2	148564193	*5′ of ACVR2A*	[1]
rs1863152	2	148564405	*5′ of ACVR2A*	[1]
rs6734998	2	148566321	*5′ of ACVR2A*	[1]
rs1991169	2	148566919	*5′ of ACVR2A*	[1]
rs12990959	2	148572160	*5′ of ACVR2A*	[1]
rs11894371	2	148575872	*5′ of ACVR2A*	[1]
rs1014064	2	148612154	*ACVR2A*	[1]
rs10803523	2	148615831	*ACVR2A*	[1]
rs2113793	2	148623453	*ACVR2A*	[1]
rs929939	2	148627528	*ACVR2A*	[1]
rs6711673	2	148643259	*ACVR2A*	[1]
rs13033696	2	148645327	*ACVR2A*	[1]
rs2161983	2	148649386	*ACVR2A*	[1]
rs13019809	2	148650473	*ACVR2A*	[1]
rs1128919	2	148657117	*AC009480.3*	[1]
rs13026650	2	148674201	*ACVR2A*	[1]
rs3764955	2	148674797	*ACVR2A*	[1]
rs1345994	2	148695126	*ORC4*	[1]
rs13012311	2	148707032	*ORC4*	[1]
rs13027200	2	148709653	*ORC4*	[1]
rs12463798	2	148716099	*ORC4*	[1]
rs2307394	2	148716428	*ORC4L*	[1]
rs6729465	2	148723126	*ORC4*	[1]
rs11901963	2	148726771	*ORC4*	[1]

rs13027706	2	148732703	*ORC4*	[1]
rs7594075	2	148739823	*ORC4*	[1]
rs13004041	2	148741621	*ORC4*	[1]
rs12463554	2	148751612	*ORC4*	[1]
rs13008838	2	148754825	*ORC4*	[1]
rs13035475	2	148754862	*ORC4*	[1]
rs13014936	2	148755237	*ORC4*	[1]
rs13022962	2	148756624	*ORC4*	[1]
rs12053401	2	148756668	*ORC4*	[1]
rs13028348	2	148757144	*ORC4*	[1]
rs1598207	2	148757870	*ORC4*	[1]
rs7598361	2	148758479	*ORC4*	[1]
rs2382201	2	148765496	*ORC4*	[1]
rs12989250	2	148776438	*ORC4*	[1]
rs1975748	2	148776859	*ORC4*	[1]
rs1015096	2	148782358	*MBD5*	[1]
rs13007770	2	148784324	*MBD5*	[1]
rs2890915	2	148789543	*MBD5*	[1]
rs144081819	2	170030639	*LRP2*	[4]
rs147287428	2	170063223	*LRP2*	[4]
rs145365776	2	170089947	*LRP2*	[4]
rs200469773	2	170093742	*LRP2*	[4]
rs145669628	2	170096097	*LRP2*	[4]
rs140061784	2	170100029	*LRP2*	[4]
rs2544390	2	170204846	*LRP2*	[3]
rs199592697	3	13896129	*WNT7A*	[4]
rs186459505	3	52941099	*SFMBT1*	[4]
rs2581824	3	53022408	*SFMBT1*	[1]
rs2564934	3	53024580	*SFMBT1*	[1]
rs2564938	3	53026384	*SFMBT1*	[1]
rs11708675	3	53031912	*SFMBT1*	[1]
rs2564919	3	53033295	*SFMBT1*	[1]
rs12635298	3	53033796	*SFMBT1*	[1]

rs2581792	3	53035044	*SFMBT1*	[1]
rs2564917	3	53037695	*SFMBT1*	[1]
rs2581795	3	53038786	*SFMBT1*	[1]
rs1529544	3	53039455	*SFMBT1*	[1]
rs17304694	3	53051577	*SFMBT1*	[1]
rs2581777	3	53054727	*SFMBT1*	[1]
rs2244552	3	53055522	*SFMBT1*	[1]
rs2244461	3	53055842	*SFMBT1*	[1]
rs9847710	3	53062661	*SFMBT1*	[1]
rs2581806	3	53063360	*SFMBT1*	[1]
rs2564956	3	53070462	*SFMBT1*	[1]
rs2581818	3	53071652	*SFMBT1*	[1]
rs6770152	3	53100214	*3′ of AC096887.1*	[1]
rs2581790	3	53101780	*3′ of AC096887.1*	[1]
rs202007714	3	66433522	*SLC25A26*	[4]
rs61746315	3	66433730	*SLC25A26*	[4]
rs201874364	3	185783611	*ETV5*	[4]
rs200263685	3	187451339	*BCL6*	[4]
rs13114077	4	9413105	*5′ of RN5S153*	[1]
rs6811069	4	9415008	*5′ of RN5S153*	[1]
rs1949921	4	9419322	*5′ of RN5S153*	[1]
rs11722202	4	9443894	*5′ of DEFB131*	[1]
rs4974886	4	9503349	*3′ of AC116655.1*	[1]
rs12513044	4	9506678	*3′ of AC116655.1*	[1]
rs11722359	4	9508214	*3′ of AC116655.1*	[1]
rs11737509	4	9511324	*3′ of AC116655.1*	[1]
rs6448742	4	9542759	*3′ of MIR548I2*	[1]
rs12233771	4	9552965	*3′ of MIR548I2*	[1]
rs10013288	4	9559218	*5′ of MIR548I2*	[1]
rs2170252	4	9560062	*5′ of MIR548I2*	[1]
rs13117722	4	9561639	*5′ of MIR548I2*	[1]
rs2061995	4	9561739	*5′ of MIR548I2*	[1]
rs13116764	4	9561759	*5′ of MIR548I2*	[1]

rs13131681	4	9563930	*5′ of MIR548I2*	[1]
rs6448816	4	9564484	*5′ of MIR548I2*	[1]
rs12646317	4	9565838	*5′ of MIR548I2*	[1]
rs11939517	4	9567260	*5′ of MIR548I2*	[1]
rs4102942	4	9568478	*5′ of MIR548I2*	[1]
rs1825043	4	9573740	*5′ of MIR548I2*	[1]
rs7669441	4	9576926	*5′ of MIR548I2*	[1]
rs9683876	4	9584058	*3′ of AC097493.1*	[1]
rs13138961	4	9585147	*3′ of AC097493.1*	[1]
rs12512447	4	9586313	*3′ of AC097493.1*	[1]
rs10049659	4	9586764	*3′ of AC097493.1*	[1]
rs1811570	4	9587999	*3′ of AC097493.1*	[1]
rs6824806	4	9589427	*3′ of AC097493.1*	[1]
rs6815602	4	9592093	*3′ of AC097493.1*	[1]
rs16898588	4	9595751	*3′ of AC097493.1*	[1]
rs6448858	4	9595918	*3′ of AC097493.1*	[1]
rs4974823	4	9602719	*5′ of AC097493.1*	[1]
rs12509677	4	9602724	*5′ of AC097493.1*	[1]
rs13103207	4	9603672	*5′ of AC097493.1*	[1]
rs2077679	4	9608089	*5′ of AC097493.1*	[1]
rs4974853	4	9609115	*5′ of AC097493.1*	[1]
rs7375281	4	9609506	*5′ of AC097493.1*	[1]
rs11724183	4	9609894	*5′ of AC097493.1*	[1]
rs4974812	4	9614598	*5′ of AC097493.1*	[1]
rs11735475	4	9614926	*5′ of AC097493.1*	[1]
rs6838199	4	9621333	*5′ of AC097493.1*	[1]
rs6837106	4	9626304	*5′ of AC097493.1*	[1]
rs10049735	4	9626409	*5′ of AC097493.1*	[1]
rs10005684	4	9626897	*5′ of AC097493.1*	[1]
rs9998663	4	9627053	*5′ of AC097493.1*	[1]
rs13151183	4	9627066	*5′ of AC097493.1*	[1]
rs13107086	4	9628088	*5′ of AC097493.1*	[1]
rs10939427	4	9628538	*5′ of AC097493.1*	[1]

rs7693695	4	9629380	*5′ of AC097493.1*	[1]
rs11939895	4	9630402	*5′ of AC097493.1*	[1]
rs9291589	4	9633410	*5′ of AC097493.1*	[1]
rs6842855	4	9633990	*5′ of AC097493.1*	[1]
rs11731624	4	9637608	*5′ of AC097493.1*	[1]
rs11737243	4	9641272	*5′ of AC097493.1*	[1]
rs11733687	4	9643374	*5′ of AC097493.1*	[1]
rs10939436	4	9647135	*5′ of AC097493.1*	[1]
rs7440232	4	9651744	*5′ of AC097493.1*	[1]
rs7654169	4	9654071	*5′ of AC097493.1*	[1]
rs6812811	4	9672000	*5′ of AC097493.1*	[1]
rs7434744	4	9672688	*5′ of AC097493.1*	[1]
rs10002984	4	9680733	*5′ of AC097493.1*	[1]
rs6858093	4	9681650	*5′ of AC097493.1*	[1]
rs13104360	4	9684804	*5′ of AC097493.1*	[1]
rs7671092	4	9687441	*3′ of SLC2A9*	[1]
rs7658414	4	9687969	*3′ of SLC2A9*	[1]
rs7691759	4	9688014	*3′ of SLC2A9*	[1]
rs6821253	4	9690731	*3′ of SLC2A9*	[1]
rs10022012	4	9693182	*3′ of SLC2A9*	[1]
rs7678732	4	9693253	*3′ of SLC2A9*	[1]
rs12649073	4	9694490	*3′ of SLC2A9*	[1]
rs13103452	4	9694662	*3′ of SLC2A9*	[1]
rs4554078	4	9701544	*3′ of SLC2A9*	[1]
rs10805313	4	9703061	*3′ of SLC2A9*	[1]
rs9685887	4	9706342	*3′ of SLC2A9*	[1]
rs9291607	4	9708798	*3′ of SLC2A9*	[1]
rs6858393	4	9709577	*3′ of SLC2A9*	[1]
rs13136075	4	9714968	*3′ of SLC2A9*	[1]
rs6448974	4	9715105	*3′ of SLC2A9*	[1]
rs9684176	4	9716386	*3′ of SLC2A9*	[1]
rs12501880	4	9717975	*3′ of SLC2A9*	[1]
rs11730320	4	9719901	*3′ of SLC2A9*	[1]

rs11727674	4	9719981	*3′ of SLC2A9*	[1]
rs11724606	4	9720444	*3′ of SLC2A9*	[1]
rs7659176	4	9721066	*3′ of SLC2A9*	[1]
rs10939472	4	9721616	*3′ of SLC2A9*	[1]
rs12645989	4	9722133	*3′ of SLC2A9*	[1]
rs13121465	4	9722275	*3′ of SLC2A9*	[1]
rs13136217	4	9722292	*3′ of SLC2A9*	[1]
rs13128435	4	9723672	*3′ of SLC2A9*	[1]
rs12647851	4	9724519	*3′ of SLC2A9*	[1]
rs2037313	4	9724711	*3′ of SLC2A9*	[1]
rs7685513	4	9728599	*3′ of SLC2A9*	[1]
rs13127001	4	9731215	*3′ of SLC2A9*	[1]
rs6811848	4	9732161	*3′ of SLC2A9*	[1]
rs7684214	4	9734585	*3′ of SLC2A9*	[1]
rs12645163	4	9735520	*3′ of SLC2A9*	[1]
rs7675599	4	9736516	*3′ of SLC2A9*	[1]
rs7667385	4	9736696	*3′ of SLC2A9*	[1]
rs6832128	4	9738151	*3′ of SLC2A9*	[1]
rs6448981	4	9740325	*3′ of SLC2A9*	[1]
rs1818670	4	9745390	*3′ of SLC2A9*	[1]
rs12508809	4	9748034	*3′ of SLC2A9*	[1]
rs6834270	4	9756710	*3′ of SLC2A9*	[1]
rs6834512	4	9756836	*3′ of SLC2A9*	[1]
rs6834697	4	9756914	*3′ of SLC2A9*	[1]
rs11727873	4	9758469	*3′ of SLC2A9*	[1]
rs10011621	4	9760239	*3′ of SLC2A9*	[1]
rs13140817	4	9762975	*3′ of SLC2A9*	[8]
rs11733815	4	9764282	*3′ of SLC2A9*	[1]
rs11734974	4	9764564	*3′ of SLC2A9*	[1]
rs11729600	4	9764686	*3′ of SLC2A9*	[1]
rs7655090	4	9765875	*3′ of SLC2A9*	[1]
rs11731100	4	9767180	*3′ of SLC2A9*	[1]
rs10939504	4	9767370	*3′ of SLC2A9*	[1]

rs11732272	4	9768258	*3′ of SLC2A9*	[1]
rs10939507	4	9768823	*3′ of SLC2A9*	[1]
rs10939514	4	9773196	*SLC2A9*	[1]
rs10939515	4	9773296	*SLC2A9*	[1]
rs10033951	4	9779580	*SLC2A9*	[1]
rs2867383	4	9787935	*SLC2A9*	[1]
rs1850744	4	9790712	*SLC2A9*	[1]
rs7685396	4	9794724	*SLC2A9*	[1]
rs1850739	4	9797343	*SLC2A9*	[1]
rs13106539	4	9797703	*SLC2A9*	[1]
rs6449000	4	9798612	*SLC2A9*	[1]
rs2280207	4	9799776	*SLC2A9*	[1]
rs2280208	4	9800043	*SLC2A9*	[1]
rs938556	4	9802366	*SLC2A9*	[1]
rs1519097	4	9802853	*SLC2A9*	[1]
rs1533615	4	9804524	*SLC2A9*	[1]
rs1519096	4	9805872	*SLC2A9*	[1]
rs7664572	4	9808709	*SLC2A9*	[1]
rs12500086	4	9809859	*SLC2A9*	[1]
rs1519095	4	9810931	*SLC2A9*	[1]
rs2280333	4	9811133	*SLC2A9*	[1]
rs1401438	4	9814456	*SLC2A9*	[1]
rs13148356	4	9817070	*SLC2A9*	[1]
rs1519094	4	9817286	*SLC2A9*	[1]
rs13141706	4	9818345	*SLC2A9*	[1]
rs6855095	4	9819977	*SLC2A9*	[1]
rs1107912	4	9820179	*SLC2A9*	[1]
rs4697892	4	9823837	*SLC2A9*	[1]
rs1980220	4	9824636	*SLC2A9*	[1]
rs4697893	4	9826183	*SLC2A9*	[1]
rs6822889	4	9826757	*SLC2A9*	[1]
rs4621429	4	9826870	*SLC2A9*	[1]
rs4697895	4	9828484	*SLC2A9*	[1]

rs1401440	4	9832512	*SLC2A9*	[1]
rs1464258	4	9834107	*SLC2A9*	[1]
rs16889260	4	9834896	*SLC2A9*	[1]
rs16889264	4	9834999	*SLC2A9*	[1]
rs12505312	4	9837090	*SLC2A9*	[1]
rs10939552	4	9838548	*SLC2A9*	[1]
rs1914874	4	9839490	*SLC2A9*	[6]
rs10031303	4	9841690	*SLC2A9*	[1]
rs950310	4	9842850	*SLC2A9*	[1]
rs10939558	4	9844162	*SLC2A9*	[1]
rs7683831	4	9845079	*SLC2A9*	[1]
rs13141635	4	9845985	*SLC2A9*	[1]
rs1976792	4	9846309	*SLC2A9*	[1]
rs13119059	4	9847860	*SLC2A9*	[1]
rs11721988	4	9848899	*SLC2A9*	[1]
rs2176644	4	9849528	*SLC2A9*	[1]
rs6812007	4	9850220	*SLC2A9*	[1]
rs4697898	4	9851827	*SLC2A9*	[8]
rs4697899	4	9851875	*SLC2A9*	[8]
rs6831796	4	9852898	*SLC2A9*	[1]
rs6826806	4	9856469	*SLC2A9*	[1]
rs4507358	4	9857956	*SLC2A9*	[8]
rs4697900	4	9859976	*SLC2A9*	[1]
rs9684729	4	9862036	*SLC2A9*	[1]
rs4697903	4	9865426	*SLC2A9*	[1]
rs2292917	4	9867277	*SLC2A9*	[1]
rs2139243	4	9867502	*SLC2A9*	[6]
rs883041	4	9868346	*SLC2A9*	[1]
rs13105954	4	9868417	*SLC2A9*	[1]
rs939134	4	9868593	*SLC2A9*	[6]
rs884573	4	9869734	*SLC2A9*	[1]
rs1048252	4	9870535	*SLC2A9*	[1]
rs1568318	4	9871541	*SLC2A9*	[6]

rs16889842	4	9872013	*SLC2A9*	[1]
rs2867394	4	9872475	*SLC2A9*	[1]
rs13148571	4	9873855	*SLC2A9*	[1]
rs12647883	4	9878464	*SLC2A9*	[1]
rs1519098	4	9881158	*SLC2A9*	[1]
rs6824636	4	9881851	*SLC2A9*	[1]
rs6449090	4	9884536	*SLC2A9*	[1]
rs10939599	4	9886860	*SLC2A9*	[1]
rs10939600	4	9886931	*SLC2A9*	[1]
rs9993652	4	9888849	*SLC2A9*	[1]
rs6818572	4	9889448	*SLC2A9*	[1]
rs1107710	4	9890708	*SLC2A9*	[1]
rs938563	4	9890998	*SLC2A9*	[6]
rs938562	4	9891031	*SLC2A9*	[1]
rs10939602	4	9892102	*SLC2A9*	[1]
rs4697692	4	9893197	*SLC2A9*	[6]
rs12644047	4	9893403	*SLC2A9*	[1]
rs7669444	4	9893577	*SLC2A9*	[1]
rs4697693	4	9895860	*SLC2A9*	[1]
rs10939605	4	9896293	*SLC2A9*	[1]
rs4697694	4	9896642	*SLC2A9*	[1]
rs6449097	4	9896734	*SLC2A9*	[1]
rs13115121	4	9897342	*SLC2A9*	[1]
rs13129868	4	9897372	*SLC2A9*	[1]
rs12505366	4	9897520	*SLC2A9*	[1]
rs13122026	4	9898207	*SLC2A9*	[1]
rs6449100	4	9901563	*SLC2A9*	[1]
rs4697910	4	9901874	*SLC2A9*	[1]
rs11732681	4	9902876	*SLC2A9*	[1]
rs11737685	4	9903121	*SLC2A9*	[6]
rs6829755	4	9903518	*SLC2A9*	[1]
rs7694136	4	9908791	*SLC2A9*	[1]
rs2280202	4	9909295	*SLC2A9*	[1]

rs2280205	4	9909923	*SLC2A9*	[9]
rs11734893	4	9910441	*SLC2A9*	[1]
rs13103429	4	9910635	*SLC2A9*	[1]
rs13108825	4	9910663	*SLC2A9*	[1]
rs11722228	4	9915741	*SLC2A9*	[1]
rs4697695	4	9915850	*SLC2A9*	[6]
rs10516194	4	9916209	*SLC2A9*	[1]
rs10805346	4	9920347	*SLC2A9*	[10]
rs874432	4	9920606	*SLC2A9*	[6]
rs6823877	4	9921931	*SLC2A9*	[1]
rs3733591	4	9922130	*SLC2A9*	[11]
rs16890979	4	9922167	*SLC2A9*	[12]
rs938564	4	9922573	*SLC2A9*	[1]
rs734553	4	9923004	*SLC2A9*	[1]
rs6832439	4	9924319	*SLC2A9*	[13]
rs938553	4	9925526	*RP13-560N11.1*	[1]
rs938554	4	9925692	*RP13-560N11.1*	[1]
rs938555	4	9926051	*SLC2A9*	[1]
rs10939614	4	9926613	*SLC2A9*	[1]
rs13129697	4	9926967	*SLC2A9*	[8]
rs6838021	4	9927620	*SLC2A9*	[1]
rs881971	4	9930962	*SLC2A9*	[1]
rs737267	4	9934744	*SLC2A9*	[2]
rs6855911	4	9935910	*SLC2A9*	[6]
rs7670751	4	9938773	*SLC2A9*	[1]
rs4447863	4	9938969	*SLC2A9*	[14]
rs938558	4	9939205	*SLC2A9*	[1]
rs4511996	4	9939818	*SLC2A9*	[1]
rs5028843	4	9940806	*SLC2A9*	[1]
rs4697913	4	9941262	*SLC2A9*	[1]
rs7675964	4	9941434	*SLC2A9*	[1]
rs4697698	4	9942577	*SLC2A9*	[6]
rs7669296	4	9942642	*SLC2A9*	[1]

rs149454410	4	9943624	*SLC2A9*	[4]
rs4292327	4	9943700	*SLC2A9*	[1]
rs12498742	4	9944052	*SLC2A9*	[1]
rs6449144	4	9944650	*SLC2A9*	[1]
rs4235346	4	9945296	*SLC2A9*	[6]
rs4697700	4	9945792	*SLC2A9*	[6]
rs4697701	4	9946095	*SLC2A9*	[1]
rs16891234	4	9946163	*SLC2A9*	[1]
rs4475146	4	9946656	*SLC2A9*	[1]
rs2018643	4	9947121	*SLC2A9*	[6]
rs1122141	4	9947278	*SLC2A9*	[1]
rs4621431	4	9947590	*SLC2A9*	[1]
rs4339211	4	9947658	*SLC2A9*	[1]
rs7694997	4	9947811	*SLC2A9*	[1]
rs7686538	4	9948077	*SLC2A9*	[1]
rs4580649	4	9948461	*SLC2A9*	[1]
rs998676	4	9948564	*SLC2A9*	[1]
rs998675	4	9948829	*SLC2A9*	[6]
rs12498150	4	9950537	*SLC2A9*	[1]
rs12498956	4	9950705	*SLC2A9*	[6]
rs13328050	4	9951120	*SLC2A9*	[6]
rs1079128	4	9951221	*SLC2A9*	[1]
rs9993410	4	9951264	*SLC2A9*	[1]
rs11723439	4	9951819	*SLC2A9*	[1]
rs4235347	4	9951956	*SLC2A9*	[1]
rs4697914	4	9952266	*SLC2A9*	[8]
rs4455410	4	9953297	*SLC2A9*	[6]
rs4560411	4	9953361	*SLC2A9*	[1]
rs4447861	4	9953940	*SLC2A9*	[1]
rs4459990	4	9954005	*SLC2A9*	[1]
rs9994266	4	9954450	*SLC2A9*	[6]
rs7376948	4	9954708	*SLC2A9*	[1]
rs7375587	4	9954758	*SLC2A9*	[1]

rs7378305	4	9954893	*SLC2A9*	[1]
rs7375599	4	9954918	*SLC2A9*	[6]
rs7378340	4	9955198	*SLC2A9*	[6]
rs4519796	4	9955936	*SLC2A9*	[1]
rs4311316	4	9955971	*SLC2A9*	[6]
rs4481233	4	9956079	*SLC2A9*	[6]
rs4314284	4	9956096	*SLC2A9*	[6]
rs4312757	4	9956145	*SLC2A9*	[1]
rs6814664	4	9956228	*SLC2A9*	[1]
rs6449155	4	9956547	*SLC2A9*	[1]
rs6449156	4	9956712	*SLC2A9*	[1]
rs17245436	4	9958169	*SLC2A9*	[1]
rs17185835	4	9958180	*SLC2A9*	[1]
rs17185870	4	9958214	*SLC2A9*	[1]
rs11724510	4	9958583	*SLC2A9*	[1]
rs6815001	4	9958662	*SLC2A9*	[1]
rs6849717	4	9958719	*SLC2A9*	[1]
rs6849729	4	9958732	*SLC2A9*	[1]
rs6843873	4	9958788	*SLC2A9*	[1]
rs6850143	4	9958924	*SLC2A9*	[1]
rs6844316	4	9958977	*SLC2A9*	[1]
rs6834893	4	9959123	*SLC2A9*	[1]
rs10001964	4	9959275	*SLC2A9*	[1]
rs4515163	4	9959603	*SLC2A9*	[1]
rs6449157	4	9960442	*SLC2A9*	[1]
rs6449159	4	9960498	*SLC2A9*	[1]
rs7672947	4	9961368	*SLC2A9*	[1]
rs17245723	4	9962218	*SLC2A9*	[1]
rs11942223	4	9962765	*SLC2A9*	[1]
rs6823361	4	9963127	*SLC2A9*	[1]
rs6836706	4	9964251	*SLC2A9*	[1]
rs6850684	4	9964380	*SLC2A9*	[1]
rs10018204	4	9964570	*SLC2A9*	[1]

rs6839490	4	9965000	*SLC2A9*	[6]
rs6856127	4	9965443	*SLC2A9*	[1]
rs6840802	4	9965633	*SLC2A9*	[6]
rs6449171	4	9965998	*SLC2A9*	[6]
rs6449172	4	9966036	*SLC2A9*	[1]
rs6449173	4	9966105	*SLC2A9*	[1]
rs6847019	4	9966249	*SLC2A9*	[1]
rs7442295	4	9966380	*SLC2A9*	[6]
rs6449174	4	9966422	*SLC2A9*	[6]
rs9998811	4	9966477	*SLC2A9*	[1]
rs7658170	4	9966593	*SLC2A9*	[6]
rs6449175	4	9966610	*SLC2A9*	[1]
rs7663097	4	9966791	*SLC2A9*	[1]
rs7676733	4	9966956	*SLC2A9*	[1]
rs10017674	4	9967053	*SLC2A9*	[1]
rs7435196	4	9967556	*SLC2A9*	[1]
rs6449176	4	9967843	*SLC2A9*	[1]
rs6449178	4	9968684	*SLC2A9*	[6]
rs6449179	4	9969117	*SLC2A9*	[1]
rs7677710	4	9969517	*SLC2A9*	[1]
rs7683283	4	9969974	*SLC2A9*	[1]
rs7376960	4	9970570	*SLC2A9*	[1]
rs6449183	4	9970691	*SLC2A9*	[1]
rs4292328	4	9970962	*SLC2A9*	[1]
rs4473653	4	9971058	*SLC2A9*	[1]
rs7439210	4	9971749	*SLC2A9*	[1]
rs13132625	4	9972163	*SLC2A9*	[1]
rs13103690	4	9972778	*SLC2A9*	[1]
rs13103879	4	9972879	*SLC2A9*	[1]
rs6852441	4	9973744	*SLC2A9*	[1]
rs6449201	4	9973894	*SLC2A9*	[1]
rs6449202	4	9974043	*SLC2A9*	[1]
rs1071988	4	9974638	*SLC2A9*	[1]

rs4505821	4	9978094	*SLC2A9*	[1]
rs62295971	4	9978142	*SLC2A9*	[11]
rs16868246	4	9978305	*SLC2A9*	[1]
rs13103497	4	9979262	*SLC2A9*	[1]
rs13144899	4	9979302	*SLC2A9*	[1]
rs13125476	4	9979614	*SLC2A9*	[1]
rs11723970	4	9980462	*SLC2A9*	[1]
rs11722229	4	9980697	*SLC2A9*	[1]
rs882223	4	9981625	*SLC2A9*	[1]
rs4364264	4	9981683	*SLC2A9*	[1]
rs13131257	4	9981889	*SLC2A9*	[13]
rs13145758	4	9981997	*SLC2A9*	[1]
rs13125029	4	9982029	*SLC2A9*	[1]
rs13125209	4	9982044	*SLC2A9*	[1]
rs13115193	4	9982191	*SLC2A9*	[1]
rs13125646	4	9982330	*SLC2A9*	[14]
rs10003001	4	9984475	*SLC2A9*	[1]
rs10033612	4	9985006	*SLC2A9*	[1]
rs11723591	4	9985398	*SLC2A9*	[1]
rs7660895	4	9985445	*SLC2A9*	[13]
rs7680126	4	9985596	*SLC2A9*	[6]
rs17246501	4	9985710	*SLC2A9*	[6]
rs9992406	4	9986288	*SLC2A9*	[1]
rs6849736	4	9986783	*SLC2A9*	[1]
rs6836200	4	9986915	*SLC2A9*	[1]
rs3733590	4	9987226	*SLC2A9*	[15]
rs4385059	4	9989233	*SLC2A9*	[6]
rs4502681	4	9990172	*SLC2A9*	[1]
rs17187075	4	9990328	*SLC2A9*	[1]
rs10011206	4	9991955	*SLC2A9*	[1]
rs7678012	4	9993772	*SLC2A9*	[1]
rs7663032	4	9993838	*SLC2A9*	[16]
rs6449213	4	9994215	*SLC2A9*	[17]

rs3775948	4	9995182	*SLC2A9*	[18]
rs12499857	4	9995376	*SLC2A9*	[1]
rs3796842	4	9995851	*SLC2A9*	[1]
rs9998739	4	9996509	*SLC2A9*	[1]
rs13111638	4	9996890	*SLC2A9*	[6]
rs4547795	4	9997060	*SLC2A9*	[1]
rs4529048	4	9997112	*SLC2A9*	[19]
rs3733588	4	9997303	*SLC2A9*	[6]
rs3733587	4	9997434	*SLC2A9*	[1]
rs7669607	4	9997801	*SLC2A9*	[6]
rs16891971	4	9998376	*SLC2A9*	[1]
rs10939650	4	9998440	*SLC2A9*	[12]
rs13113918	4	9998493	*SLC2A9*	[14]
rs10008035	4	9999335	*SLC2A9*	[1]
rs7696536	4	10000236	*SLC2A9*	[1]
rs7678287	4	10000501	*SLC2A9*	[20]
rs1014290	4	10001861	*SLC2A9*	[18]
rs7696895	4	10002425	*SLC2A9*	[1]
rs9991278	4	10002665	*SLC2A9*	[6]
rs4622999	4	10003395	*SLC2A9*	[1]
rs7657096	4	10004000	*SLC2A9*	[1]
rs17247314	4	10004743	*SLC2A9*	[1]
rs10023068	4	10004832	*SLC2A9*	[6]
rs6853437	4	10005435	*SLC2A9*	[6]
rs10022499	4	10006537	*SLC2A9*	[1]
rs9291640	4	10007086	*SLC2A9*	[6]
rs9291642	4	10007275	*SLC2A9*	[14]
rs4543113	4	10008305	*RP11-448G15.1*	[1]
rs6845554	4	10013173	*SLC2A9*	[1]
rs3756236	4	10013463	*SLC2A9*	[1]
rs6827754	4	10018153	*SLC2A9*	[6]
rs13128385	4	10019563	*SLC2A9*	[1]
rs13133766	4	10019732	*SLC2A9*	[1]

rs3775942	4	10020307	*SLC2A9*	[1]
rs2240720	4	10020480	*SLC2A9*	[1]
rs2240721	4	10020564	*SLC2A9*	[1]
rs2240723	4	10021151	*SLC2A9*	[1]
rs2240724	4	10021290	*SLC2A9*	[1]
rs6849273	4	10021595	*SLC2A9*	[1]
rs2276961	4	10022981	*SLC2A9*	[12]
rs12509955	4	10024303	*SLC2A9*	[6]
rs3775940	4	10025163	*SLC2A9*	[6]
rs6826764	4	10030794	*SLC2A9*	[6]
rs6856396	4	10031163	*SLC2A9*	[16]
rs12506455	4	10031569	*SLC2A9*	[1]
rs10939663	4	10032516	*SLC2A9*	[1]
rs12506122	4	10033538	*SLC2A9*	[1]
rs13146686	4	10034933	*SLC2A9*	[6]
rs11722930	4	10035454	*SLC2A9*	[1]
rs16892419	4	10035702	*SLC2A9*	[1]
rs16892420	4	10035715	*SLC2A9*	[1]
rs10006397	4	10036140	*SLC2A9*	[1]
rs11727199	4	10036190	*SLC2A9*	[1]
rs3733585	4	10036339	*SLC2A9*	[1]
rs3822250	4	10036630	*SLC2A9*	[1]
rs11731110	4	10037346	*SLC2A9*	[1]
rs10939665	4	10037628	*SLC2A9*	[1]
rs10012779	4	10038112	*SLC2A9*	[1]
rs13139055	4	10038924	*SLC2A9*	[1]
rs13115776	4	10040189	*SLC2A9*	[1]
rs16892474	4	10040220	*SLC2A9*	[1]
rs12508991	4	10041104	*SLC2A9*	[1]
rs10029311	4	10041134	*SLC2A9*	[1]
rs7679916	4	10042160	*SLC2A9*	[1]
rs7349721	4	10042562	*SLC2A9*	[1]
rs13101785	4	10042915	*SLC2A9*	[1]

rs13137343	4	10043028	*SLC2A9*	[1]
rs13110307	4	10044364	*SLC2A9*	[1]
rs13129453	4	10044784	*SLC2A9*	[1]
rs4529049	4	10045389	*SLC2A9*	[1]
rs4637402	4	10045430	*SLC2A9*	[8]
rs10939669	4	10045827	*SLC2A9*	[1]
rs11734375	4	10046298	*SLC2A9*	[21]
rs4608811	4	10049675	*SLC2A9*	[6]
rs733175	4	10050141	*SLC2A9*	[11]
rs6829727	4	10051672	*SLC2A9*	[6]
rs13120348	4	10053155	*SLC2A9*	[6]
rs7671266	4	10056376	*SLC2A9*	[6]
rs10516198	4	10059448	*5′ of SLC2A9*	[1]
rs714873	4	10059618	*5′ of SLC2A9*	[1]
rs6834555	4	10062326	*5′ of SLC2A9*	[11]
rs6820756	4	10062849	*5′ of SLC2A9*	[6]
rs12506004	4	10066870	*3′ of RP11-448G15.3*	[1]
rs16868313	4	10068064	*3′ of RP11-448G15.3*	[6]
rs3775938	4	10069531	*3′ of RP11-448G15.3*	[1]
rs7667452	4	10072912	*RP11-448G15.3*	[1]
rs4320137	4	10072969	*RP11-448G15.3*	[6]
rs4461524	4	10074170	*RP11-448G15.3*	[1]
rs11731597	4	10075485	*3′ of WDR1*	[6]
rs9926	4	10076860	*WDR1*	[1]
rs3775935	4	10077638	*WDR1*	[1]
rs2241469	4	10080462	*WDR1*	[1]
rs10516200	4	10082156	*WDR1*	[6]
rs3756230	4	10083829	*WDR1*	[1]
rs2241470	4	10084570	*WDR1*	[1]
rs2241473	4	10085949	*WDR1*	[1]
rs2241475	4	10086188	*WDR1*	[6]
rs3756227	4	10087995	*WDR1*	[1]

rs2241480	4	10089763	*WDR1*	[6]
rs734122	4	10089865	*WDR1*	[6]
rs3796822	4	10093651	*WDR1*	[1]
rs3822242	4	10094904	*WDR1*	[1]
rs3822239	4	10095539	*WDR1*	[1]
rs11727087	4	10096020	*WDR1*	[6]
rs3796818	4	10097976	*WDR1*	[1]
rs11726271	4	10098192	*WDR1*	[1]
rs2241482	4	10099814	*WDR1*	[1]
rs2241483	4	10099831	*WDR1*	[1]
rs2241486	4	10101083	*WDR1*	[1]
rs2241488	4	10101131	*WDR1*	[1]
rs6830786	4	10101443	*WDR1*	[1]
rs4697917	4	10101960	*WDR1*	[1]
rs16868326	4	10102593	*WDR1*	[1]
rs12499240	4	10103890	*WDR1*	[1]
rs717615	4	10104670	*WDR1*	[6]
rs717614	4	10104788	*WDR1*	[22]
rs3756224	4	10105739	*WDR1*	[23]
rs3756223	4	10105797	*WDR1*	[6]
rs12509714	4	10107091	*WDR1*	[6]
rs4697922	4	10110605	*WDR1*	[1]
rs4459989	4	10112602	*WDR1*	[1]
rs2241468	4	10113905	*WDR1*	[1]
rs4604059	4	10115065	*WDR1*	[6]
rs4393994	4	10115121	*WDR1*	[1]
rs12498927	4	10115523	*WDR1*	[6]
rs10939710	4	10116801	*WDR1*	[1]
rs35782983	4	10117728	*WDR1*	[12]
rs3822236	4	10119961	*5′ of WDR1*	[1]
rs12374320	4	10120609	*5′ of WDR1*	[1]
rs4697708	4	10121189	*5′ of WDR1*	[6]
rs3756215	4	10121260	*5′ of WDR1*	[1]

rs4697710	4	10122649	*5′ of WDR1*	[1]
rs6825888	4	10122734	*5′ of WDR1*	[6]
rs4235354	4	10122942	*5′ of WDR1*	[1]
rs4235355	4	10123078	*5′ of WDR1*	[1]
rs4235356	4	10123106	*5′ of WDR1*	[1]
rs4311315	4	10123191	*5′ of WDR1*	[1]
rs12506893	4	10123665	*5′ of WDR1*	[1]
rs10516201	4	10123941	*5′ of WDR1*	[6]
rs4697924	4	10124239	*5′ of WDR1*	[6]
rs4697926	4	10124567	*5′ of WDR1*	[6]
rs4444830	4	10124819	*5′ of WDR1*	[6]
rs4456954	4	10124838	*5′ of WDR1*	[1]
rs715979	4	10125242	*5′ of WDR1*	[1]
rs3886038	4	10125255	*5′ of WDR1*	[6]
rs7699512	4	10125808	*5′ of WDR1*	[1]
rs7699671	4	10125874	*5′ of WDR1*	[1]
rs11722989	4	10126139	*5′ of WDR1*	[6]
rs11723016	4	10126189	*5′ of WDR1*	[1]
rs6449286	4	10126699	*5′ of WDR1*	[1]
rs4619888	4	10126978	*5′ of WDR1*	[1]
rs4467562	4	10127141	*5′ of WDR1*	[1]
rs10001106	4	10127441	*5′ of WDR1*	[1]
rs11722643	4	10127484	*5′ of WDR1*	[1]
rs17250843	4	10127978	*5′ of WDR1*	[1]
rs715259	4	10128071	*5′ of WDR1*	[1]
rs715260	4	10128146	*5′ of WDR1*	[1]
rs7667775	4	10128536	*5′ of WDR1*	[1]
rs10155145	4	10128800	*5′ of WDR1*	[1]
rs12502342	4	10129916	*5′ of WDR1*	[1]
rs12502368	4	10129953	*5′ of WDR1*	[1]
rs12502556	4	10130505	*5′ of WDR1*	[1]
rs10009493	4	10132047	*5′ of WDR1*	[1]
rs12501597	4	10132137	*5′ of WDR1*	[1]

rs6449289	4	10132543	*5′ of WDR1*	[1]
rs881641	4	10133747	*5′ of WDR1*	[1]
rs881642	4	10133873	*5′ of WDR1*	[1]
rs881643	4	10134117	*5′ of WDR1*	[6]
rs17197769	4	10134336	*5′ of WDR1*	[1]
rs1109472	4	10134448	*5′ of WDR1*	[6]
rs11938608	4	10135849	*5′ of WDR1*	[1]
rs4358401	4	10136807	*5′ of WDR1*	[1]
rs873984	4	10137125	*5′ of WDR1*	[1]
rs4399989	4	10137388	*5′ of WDR1*	[1]
rs4315785	4	10137607	*5′ of WDR1*	[1]
rs4235357	4	10137756	*5′ of WDR1*	[1]
rs12507725	4	10137790	*5′ of WDR1*	[1]
rs17198113	4	10138470	*5′ of WDR1*	[6]
rs11732828	4	10138746	*5′ of WDR1*	[1]
rs10939722	4	10139047	*5′ of WDR1*	[1]
rs10939723	4	10139105	*5′ of WDR1*	[6]
rs17198197	4	10139157	*5′ of WDR1*	[1]
rs6449300	4	10139551	*5′ of WDR1*	[1]
rs6850516	4	10139978	*5′ of WDR1*	[1]
rs10025456	4	10140259	*5′ of WDR1*	[1]
rs10022911	4	10140551	*5′ of WDR1*	[13]
rs10020887	4	10140751	*5′ of WDR1*	[1]
rs10012288	4	10140953	*5′ of WDR1*	[1]
rs4533775	4	10141404	*5′ of WDR1*	[1]
rs17198547	4	10141419	*5′ of WDR1*	[6]
rs10805356	4	10141602	*5′ of WDR1*	[1]
rs4473652	4	10141617	*5′ of WDR1*	[1]
rs10028503	4	10141927	*5′ of WDR1*	[6]
rs10015494	4	10141990	*5′ of WDR1*	[1]
rs10028937	4	10142366	*5′ of WDR1*	[6]
rs10015872	4	10142410	*5′ of WDR1*	[6]
rs17251963	4	10142561	*5′ of WDR1*	[6]

rs10031453	4	10142659	*5′ of WDR1*	[1]
rs4697713	4	10143582	*5′ of WDR1*	[1]
rs4697930	4	10143594	*5′ of WDR1*	[1]
rs4697714	4	10143786	*5′ of WDR1*	[6]
rs4292329	4	10144459	*5′ of WDR1*	[1]
rs4697931	4	10145296	*5′ of WDR1*	[6]
rs4574408	4	10145568	*5′ of WDR1*	[6]
rs4640669	4	10145733	*5′ of WDR1*	[6]
rs4484300	4	10145794	*5′ of WDR1*	[6]
rs4401449	4	10145998	*5′ of WDR1*	[1]
rs10939730	4	10146049	*5′ of WDR1*	[1]
rs4697933	4	10146493	*5′ of WDR1*	[1]
rs4697715	4	10146654	*5′ of WDR1*	[1]
rs6855657	4	10148148	*5′ of WDR1*	[1]
rs10939732	4	10148390	*5′ of WDR1*	[1]
rs12507586	4	10148575	*5′ of WDR1*	[1]
rs4168	4	10148609	*5′ of WDR1*	[1]
rs11735668	4	10148655	*5′ of WDR1*	[6]
rs12508413	4	10148671	*5′ of WDR1*	[1]
rs6813334	4	10148753	*5′ of WDR1*	[1]
rs6813385	4	10148828	*5′ of WDR1*	[1]
rs6846402	4	10148881	*5′ of WDR1*	[1]
rs4697934	4	10149064	*5′ of WDR1*	[6]
rs4697936	4	10149595	*5′ of WDR1*	[1]
rs4697937	4	10149669	*5′ of WDR1*	[6]
rs6826693	4	10150739	*5′ of WDR1*	[1]
rs6840883	4	10150864	*5′ of WDR1*	[1]
rs11721682	4	10151147	*5′ of WDR1*	[6]
rs6827946	4	10151335	*5′ of WDR1*	[1]
rs6827496	4	10151656	*5′ of WDR1*	[1]
rs6847379	4	10151704	*5′ of WDR1*	[1]
rs4697940	4	10152329	*5′ of WDR1*	[1]
rs731069	4	10152431	*5′ of WDR1*	[1]

rs731070	4	10152582	*5′ of WDR1*	[1]
rs747357	4	10152878	*5′ of WDR1*	[1]
rs747356	4	10153051	*5′ of WDR1*	[1]
rs737601	4	10153233	*5′ of WDR1*	[1]
rs6814532	4	10154554	*5′ of WDR1*	[1]
rs11736560	4	10154872	*5′ of WDR1*	[1]
rs6851524	4	10155041	*5′ of WDR1*	[1]
rs10939741	4	10155346	*5′ of WDR1*	[1]
rs11932627	4	10156177	*5′ of WDR1*	[1]
rs12513376	4	10156222	*5′ of WDR1*	[1]
rs11929718	4	10156321	*5′ of WDR1*	[6]
rs12498256	4	10156544	*5′ of WDR1*	[1]
rs6823778	4	10158163	*5′ of WDR1*	[11]
rs6834055	4	10158511	*5′ of WDR1*	[1]
rs2241464	4	10158961	*5′ of WDR1*	[1]
rs2241465	4	10159020	*5′ of WDR1*	[1]
rs4697941	4	10160862	*5′ of WDR1*	[6]
rs17450260	4	10163789	*5′ of WDR1*	[1]
rs6816215	4	10164027	*5′ of WDR1*	[1]
rs17450372	4	10164344	*5′ of WDR1*	[1]
rs1009144	4	10164931	*5′ of WDR1*	[1]
rs17450434	4	10164961	*5′ of WDR1*	[1]
rs16894270	4	10165779	*5′ of WDR1*	[1]
rs11729371	4	10165957	*5′ of WDR1*	[6]
rs11724641	4	10166994	*5′ of WDR1*	[1]
rs7659717	4	10167217	*5′ of WDR1*	[1]
rs17385112	4	10167532	*5′ of WDR1*	[1]
rs17385294	4	10168447	*5′ of WDR1*	[1]
rs1001216	4	10168849	*5′ of WDR1*	[1]
rs1001217	4	10168999	*5′ of WDR1*	[1]
rs11734209	4	10170371	*5′ of WDR1*	[1]
rs874079	4	10171105	*5′ of WDR1*	[1]
rs7686718	4	10171487	*5′ of WDR1*	[1]

rs4697948	4	10171567	*5′ of WDR1*	[1]
rs4697950	4	10171644	*5′ of WDR1*	[11]
rs2080076	4	10171945	*5′ of WDR1*	[1]
rs2098234	4	10172113	*5′ of WDR1*	[1]
rs6835689	4	10172479	*5′ of WDR1*	[1]
rs55672974	4	10173696	*WDR1-*	[6]
rs10034405	4	10173696	*5′ of WDR1*	[8]
rs4697718	4	10174139	*5′ of WDR1*	[8]
rs4697719	4	10174158	*5′ of WDR1*	[6]
rs4697721	4	10174217	*5′ of WDR1*	[1]
rs4697954	4	10174529	*5′ of WDR1*	[8]
rs10017447	4	10175536	*5′ of WDR1*	[11]
rs10024152	4	10175689	*5′ of WDR1*	[8]
rs2903827	4	10175872	*5′ of WDR1*	[1]
rs2868414	4	10177120	*5′ of WDR1*	[1]
rs4697724	4	10177818	*5′ of WDR1*	[11]
rs17385872	4	10178168	*5′ of WDR1*	[6]
rs16894579	4	10178217	*5′ of WDR1*	[1]
rs10033955	4	10178556	*5′ of WDR1*	[1]
rs11737347	4	10178922	*5′ of WDR1*	[1]
rs7657551	4	10179309	*5′ of WDR1*	[1]
rs6449342	4	10179769	*5′ of WDR1*	[1]
rs4273473	4	10180622	*5′ of WDR1*	[6]
rs11724112	4	10180643	*5′ of WDR1*	[1]
rs6811287	4	10180823	*5′ of WDR1*	[1]
rs723663	4	10181374	*5′ of WDR1*	[6]
rs12500891	4	10181387	*5′ of WDR1*	[6]
rs4697956	4	10181672	*5′ of WDR1*	[1]
rs4697957	4	10182254	*5′ of WDR1*	[1]
rs4697958	4	10182556	*5′ of WDR1*	[1]
rs887735	4	10182845	*5′ of WDR1*	[6]
rs887734	4	10182913	*5′ of WDR1*	[11]
rs887733	4	10183108	*5′ of WDR1*	[1]

rs887732	4	10183117	*5′ of WDR1*	[1]
rs887731	4	10183186	*5′ of WDR1*	[1]
rs887729	4	10183611	*5′ of WDR1*	[6]
rs887728	4	10183798	*5′ of WDR1*	[1]
rs887727	4	10183819	*5′ of WDR1*	[1]
rs887725	4	10183895	*5′ of WDR1*	[1]
rs10025980	4	10185799	*5′ of WDR1*	[11]
rs11723976	4	10186251	*5′ of WDR1*	[11]
rs11724092	4	10186604	*5′ of WDR1*	[11]
rs11722345	4	10186799	*5′ of WDR1*	[1]
rs4697960	4	10187263	*5′ of WDR1*	[1]
rs4697961	4	10187373	*5′ of WDR1*	[11]
rs4697726	4	10187395	*5′ of WDR1*	[11]
rs56391253	4	10187580	*5′ of WDR1*	[11]
rs4697727	4	10187922	*5′ of WDR1*	[6]
rs956312	4	10188326	*5′ of WDR1*	[22]
rs956311	4	10188381	*5′ of WDR1*	[6]
rs4697962	4	10188832	*5′ of WDR1*	[11]
rs11721530	4	10189162	*5′ of WDR1*	[6]
rs62285986	4	10189213	*5′ of WDR1*	[11]
rs4697963	4	10189483	*5′ of WDR1*	[11]
rs4697964	4	10189812	*5′ of WDR1*	[1]
rs4697965	4	10190058	*5′ of WDR1*	[6]
rs4697966	4	10190074	*5′ of WDR1*	[6]
rs28496435	4	10190318	*5′ of WDR1*	[11]
rs11726987	4	10190792	*5′ of WDR1*	[11]
rs10000104	4	10191766	*5′ of WDR1*	[11]
rs2215691	4	10192108	*5′ of WDR1*	[11]
rs6449351	4	10192744	*5′ of WDR1*	[11]
rs2159865	4	10193287	*5′ of WDR1*	[11]
rs10489080	4	10193427	*5′ of WDR1*	[6]
rs2159864	4	10193604	*5′ of WDR1*	[1]
rs6832085	4	10194270	*5′ of WDR1*	[11]

rs6826383	4	10194504	*5′ of WDR1*	[1]
rs4697728	4	10196638	*5′ of WDR1*	[1]
rs917825	4	10196761	*5′ of WDR1*	[6]
rs1860903	4	10196853	*5′ of WDR1*	[1]
rs929575	4	10196886	*5′ of WDR1*	[1]
rs917823	4	10197225	*5′ of WDR1*	[1]
rs66769576	4	10197663	*5′ of WDR1*	[11]
rs6836606	4	10198086	*5′ of WDR1*	[11]
rs4697968	4	10198628	*5′ of WDR1*	[6]
rs4697969	4	10198981	*5′ of WDR1*	[8]
rs11726996	4	10199139	*5′ of WDR1*	[11]
rs2080075	4	10199448	*5′ of WDR1*	[1]
rs55878266	4	10199948	*5′ of WDR1*	[11]
rs55775442	4	10200204	*5′ of WDR1*	[11]
rs4697729	4	10200401	*5′ of WDR1*	[1]
rs4697730	4	10200496	*5′ of WDR1*	[6]
rs4697731	4	10200718	*5′ of WDR1*	[11]
rs4697971	4	10200860	*5′ of WDR1*	[1]
rs4697972	4	10201503	*5′ of WDR1*	[11]
rs1990469	4	10201652	*5′ of WDR1*	[11]
rs4697732	4	10201873	*5′ of WDR1*	[1]
rs2868416	4	10202574	*5′ of WDR1*	[1]
rs2868420	4	10202997	*5′ of WDR1*	[11]
rs4697973	4	10203152	*5′ of WDR1*	[11]
rs10489079	4	10203963	*5′ of WDR1*	[1]
rs6858209	4	10204157	*5′ of WDR1*	[1]
rs9283699	4	10204189	*5′ of WDR1*	[1]
rs10030776	4	10204475	*5′ of WDR1*	[1]
rs10030782	4	10204496	*5′ of WDR1*	[1]
rs9990501	4	10204593	*5′ of WDR1*	[11]
rs10939766	4	10204970	*5′ of WDR1*	[6]
rs16894893	4	10205595	*5′ of WDR1*	[1]
rs4697974	4	10205718	*5′ of WDR1*	[11]

rs231	4	10205848	*5′ of WDR1*	[1]
rs17455117	4	10206204	*5′ of WDR1*	[1]
rs6449355	4	10206731	*5′ of WDR1*	[1]
rs17389602	4	10206926	*5′ of WDR1*	[1]
rs7690319	4	10207061	*5′ of WDR1*	[11]
rs10026434	4	10208128	*5′ of WDR1*	[11]
rs11734623	4	10208303	*5′ of WDR1*	[11]
rs6826185	4	10208656	*5′ of WDR1*	[11]
rs6812780	4	10208725	*5′ of WDR1*	[6]
rs6826450	4	10208778	*5′ of WDR1*	[6]
rs6845818	4	10208794	*5′ of WDR1*	[6]
rs6855489	4	10208888	*5′ of WDR1*	[6]
rs11947517	4	10209251	*5′ of WDR1*	[6]
rs4697977	4	10210653	*5′ of WDR1*	[1]
rs2080072	4	10235622	*5′ of WDR1*	[1]
rs11734783	4	10240663	*5′ of WDR1*	[6]
rs917821	4	10241132	*5′ of WDR1*	[1]
rs11727366	4	10244841	*5′ of WDR1*	[1]
rs10023177	4	10244955	*5′ of WDR1*	[6]
rs1978274	4	10245087	*5′ of WDR1*	[1]
rs7675945	4	10245340	*5′ of WDR1*	[1]
rs6853056	4	10245898	*5′ of WDR1*	[6]
rs10010656	4	10246327	*5′ of WDR1*	[1]
rs11732042	4	10246448	*5′ of WDR1*	[1]
rs4697983	4	10247248	*5′ of WDR1*	[1]
rs10939801	4	10247512	*5′ of WDR1*	[6]
rs11730940	4	10247893	*5′ of WDR1*	[1]
rs10025702	4	10248174	*5′ of WDR1*	[6]
rs2024282	4	10249688	*5′ of WDR1*	[1]
rs2024281	4	10249751	*5′ of WDR1*	[1]
rs6449395	4	10249877	*5′ of WDR1*	[1]
rs7661555	4	10250026	*5′ of WDR1*	[6]
rs17392044	4	10250340	*5′ of WDR1*	[1]

rs1017124	4	10250399	*5′ of WDR1*	[6]
rs12509424	4	10250503	*5′ of WDR1*	[1]
rs1860896	4	10250777	*5′ of WDR1*	[1]
rs1860895	4	10250779	*5′ of WDR1*	[11]
rs16895216	4	10250983	*5′ of WDR1*	[1]
rs11735543	4	10251652	*5′ of WDR1*	[1]
rs11735623	4	10251925	*5′ of WDR1*	[11]
rs10029208	4	10252005	*5′ of WDR1*	[1]
rs6838644	4	10252871	*5′ of WDR1*	[1]
rs4522862	4	10253015	*5′ of WDR1*	[1]
rs4406017	4	10253169	*5′ of WDR1*	[1]
rs11724760	4	10254162	*5′ of WDR1*	[11]
rs4697984	4	10254550	*5′ of WDR1*	[1]
rs12513165	4	10256577	*5′ of WDR1*	[1]
rs2192101	4	10258057	*5′ of WDR1*	[1]
rs2192100	4	10258373	*5′ of WDR1*	[1]
rs4697986	4	10258968	*5′ of WDR1*	[1]
rs4697736	4	10259834	*5′ of WDR1*	[1]
rs17406107	4	10263380	*5′ of WDR1*	[1]
rs4697995	4	10264276	*5′ of WDR1*	[1]
rs929577	4	10264888	*5′ of WDR1*	[6]
rs759031	4	10265433	*5′ of WDR1*	[1]
rs17472370	4	10270131	*5′ of WDR1*	[1]
rs10489076	4	10270848	*5′ of WDR1*	[1]
rs13134726	4	10271119	*5′ of WDR1*	[1]
rs12505222	4	10271136	*5′ of WDR1*	[1]
rs7676442	4	10272591	*5′ of WDR1*	[1]
rs10489074	4	10272605	*5′ of WDR1*	[1]
rs10489073	4	10272788	*5′ of WDR1*	[6]
rs17407324	4	10273043	*5′ of WDR1*	[6]
rs10489072	4	10273244	*5′ of WDR1*	[1]
rs10939814	4	10273329	*5′ of WDR1*	[1]
rs10489071	4	10273549	*5′ of WDR1*	[1]

rs2192095	4	10274173	*5′ of WDR1*	[1]
rs4697998	4	10274626	*5′ of WDR1*	[1]
rs4697999	4	10274934	*5′ of WDR1*	[1]
rs17407555	4	10274994	*5′ of WDR1*	[1]
rs1860911	4	10275057	*5′ of WDR1*	[1]
rs1860910	4	10275470	*5′ of WDR1*	[1]
rs10805364	4	10275518	*5′ of WDR1*	[13]
rs6823180	4	10275831	*5′ of WDR1*	[1]
rs6833142	4	10275982	*5′ of WDR1*	[1]
rs10489070	4	10276352	*5′ of WDR1*	[24]
rs12510549	4	10276467	*5′ of WDR1*	[6]
rs4698000	4	10277467	*5′ of WDR1*	[1]
rs6836916	4	10277792	*5′ of WDR1*	[1]
rs11736814	4	10277869	*5′ of WDR1*	[1]
rs16895836	4	10278349	*5′ of WDR1*	[1]
rs7435841	4	10278552	*5′ of WDR1*	[1]
rs10489069	4	10278668	*5′ of WDR1*	[1]
rs10032742	4	10278893	*5′ of WDR1*	[1]
rs4698001	4	10279413	*5′ of WDR1*	[1]
rs1860909	4	10279557	*5′ of WDR1*	[1]
rs17474174	4	10281411	*3′ of ZNF518B*	[1]
rs2159868	4	10283560	*3′ of ZNF518B*	[1]
rs4698005	4	10283627	*3′ of ZNF518B*	[1]
rs4697740	4	10284565	*3′ of ZNF518B*	[1]
rs16895984	4	10284727	*3′ of ZNF518B*	[6]
rs4698009	4	10284993	*3′ of ZNF518B*	[6]
rs4698014	4	10286301	*3′ of ZNF518B*	[13]
rs17409460	4	10286427	*3′ of ZNF518B*	[1]
rs7685241	4	10286665	*3′ of ZNF518B*	[1]
rs12640013	4	10286687	*3′ of ZNF518B*	[1]
rs10939818	4	10286962	*3′ of ZNF518B*	[11]
rs17475334	4	10287170	*3′ of ZNF518B*	[1]
rs11932349	4	10287251	*3′ of ZNF518B*	[6]

rs10489068	4	10287268	*3′ of ZNF518B*	[6]
rs11945358	4	10287559	*3′ of ZNF518B*	[11]
rs17475461	4	10287677	*3′ of ZNF518B*	[1]
rs7670709	4	10288932	*3′ of ZNF518B*	[11]
rs11937220	4	10289874	*3′ of ZNF518B*	[6]
rs757628	4	10290297	*3′ of ZNF518B*	[11]
rs7692559	4	10290726	*3′ of ZNF518B*	[1]
rs6449438	4	10291063	*3′ of ZNF518B*	[1]
rs2024280	4	10291813	*3′ of ZNF518B*	[6]
rs2192094	4	10291968	*3′ of ZNF518B*	[1]
rs11730631	4	10292968	*3′ of ZNF518B*	[6]
rs11731652	4	10292984	*3′ of ZNF518B*	[1]
rs917827	4	10295500	*3′ of ZNF518B*	[1]
rs6839820	4	10296114	*3′ of ZNF518B*	[11]
rs11728055	4	10296298	*3′ of ZNF518B*	[6]
rs1860907	4	10296699	*3′ of ZNF518B*	[1]
rs6856707	4	10297330	*3′ of ZNF518B*	[11]
rs17410735	4	10297447	*3′ of ZNF518B*	[1]
rs4698017	4	10298094	*3′ of ZNF518B*	[11]
rs4697744	4	10298147	*3′ of ZNF518B*	[11]
rs11732729	4	10300316	*3′ of ZNF518B*	[1]
rs2080077	4	10300382	*3′ of ZNF518B*	[1]
rs2098236	4	10300472	*3′ of ZNF518B*	[1]
rs6834574	4	10300814	*3′ of ZNF518B*	[1]
rs10939829	4	10300819	*3′ of ZNF518B*	[1]
rs7683755	4	10301757	*3′ of ZNF518B*	[1]
rs10014800	4	10302493	*3′ of ZNF518B*	[11]
rs2868937	4	10303081	*3′ of ZNF518B*	[1]
rs4698023	4	10304726	*3′ of ZNF518B*	[1]
rs4698025	4	10305157	*3′ of ZNF518B*	[1]
rs7689060	4	10305463	*3′ of ZNF518B*	[6]
rs1468692	4	10305775	*3′ of ZNF518B*	[1]
rs991458	4	10308756	*3′ of ZNF518B*	[1]

rs9991653	4	10310495	*3′ of ZNF518B*	[1]
rs7436833	4	10311074	*3′ of ZNF518B*	[1]
rs6449449	4	10311707	*3′ of ZNF518B*	[1]
rs6449450	4	10311887	*3′ of ZNF518B*	[11]
rs12511337	4	10311972	*3′ of ZNF518B*	[6]
rs6449451	4	10312073	*3′ of ZNF518B*	[6]
rs6449452	4	10312112	*3′ of ZNF518B*	[1]
rs4698028	4	10312216	*3′ of ZNF518B*	[6]
rs4698029	4	10312798	*3′ of ZNF518B*	[6]
rs2192093	4	10313106	*3′ of ZNF518B*	[6]
rs6810699	4	10314025	*3′ of ZNF518B*	[6]
rs727995	4	10314177	*3′ of ZNF518B*	[1]
rs727996	4	10314188	*3′ of ZNF518B*	[1]
rs714436	4	10314667	*3′ of ZNF518B*	[13]
rs2868939	4	10314921	*3′ of ZNF518B*	[1]
rs17477561	4	10315096	*3′ of ZNF518B*	[6]
rs4698031	4	10315921	*3′ of ZNF518B*	[1]
rs6449453	4	10316834	*3′ of ZNF518B*	[1]
rs17418478	4	10316851	*3′ of ZNF518B*	[1]
rs7666514	4	10316879	*3′ of ZNF518B*	[1]
rs17418533	4	10316941	*3′ of ZNF518B*	[1]
rs6449454	4	10317381	*3′ of ZNF518B*	[1]
rs11722185	4	10317482	*3′ of ZNF518B*	[1]
rs1860905	4	10318836	*3′ of ZNF518B*	[1]
rs11737588	4	10319007	*3′ of ZNF518B*	[1]
rs4698033	4	10319571	*3′ of ZNF518B*	[1]
rs4697748	4	10319699	*3′ of ZNF518B*	[1]
rs12506560	4	10322032	*3′ of ZNF518B*	[1]
rs12506625	4	10322185	*3′ of ZNF518B*	[1]
rs993173	4	10323935	*3′ of ZNF518B*	[1]
rs9291683	4	10324160	*3′ of ZNF518B*	[11]
rs17478453	4	10324312	*3′ of ZNF518B*	[6]
rs993172	4	10324361	*3′ of ZNF518B*	[6]

rs1558489	4	10325489	*3′ of ZNF518B*	[1]
rs1558488	4	10327414	*3′ of ZNF518B*	[1]
rs4698036	4	10331294	*3′ of ZNF518B*	[13]
rs11729318	4	10336919	*3′ of ZNF518B*	[1]
rs17419612	4	10337263	*3′ of ZNF518B*	[1]
rs1964268	4	10337921	*3′ of ZNF518B*	[1]
rs13142790	4	10339040	*3′ of ZNF518B*	[1]
rs759024	4	10339095	*3′ of ZNF518B*	[1]
rs759022	4	10339426	*3′ of ZNF518B*	[1]
rs10489067	4	10340486	*3′ of ZNF518B*	[1]
rs2007103	4	10341255	*3′ of ZNF518B*	[1]
rs4306950	4	10341419	*3′ of ZNF518B*	[6]
rs10938761	4	10342723	*3′ of ZNF518B*	[1]
rs984723	4	10344624	*3′ of ZNF518B*	[6]
rs2052165	4	10345172	*3′ of ZNF518B*	[1]
rs1035052	4	10345339	*3′ of ZNF518B*	[1]
rs17420080	4	10345548	*3′ of ZNF518B*	[13]
rs13109847	4	10346245	*3′ of ZNF518B*	[6]
rs17479487	4	10346550	*3′ of ZNF518B*	[1]
rs2192084	4	10347051	*3′ of ZNF518B*	[1]
rs16897170	4	10347414	*3′ of ZNF518B*	[1]
rs2192083	4	10347764	*3′ of ZNF518B*	[1]
rs6849037	4	10348431	*3′ of ZNF518B*	[1]
rs4698037	4	10348895	*3′ of ZNF518B*	[6]
rs1544599	4	10349168	*3′ of ZNF518B*	[11]
rs17420450	4	10350765	*3′ of ZNF518B*	[1]
rs13145430	4	10350948	*3′ of ZNF518B*	[6]
rs17420513	4	10350975	*3′ of ZNF518B*	[1]
rs17420562	4	10351595	*3′ of ZNF518B*	[1]
rs4697750	4	10351706	*3′ of ZNF518B*	[1]
rs6853659	4	10351970	*3′ of ZNF518B*	[1]
rs4697751	4	10352506	*3′ of ZNF518B*	[1]
rs4698040	4	10352550	*3′ of ZNF518B*	[1]

rs4697752	4	10353381	*3′ of ZNF518B*	[1]
rs2098235	4	10354069	*3′ of ZNF518B*	[1]
rs10938768	4	10354341	*3′ of ZNF518B*	[1]
rs6838846	4	10355529	*3′ of ZNF518B*	[1]
rs11931317	4	10356348	*3′ of ZNF518B*	[1]
rs1860904	4	10357448	*3′ of ZNF518B*	[6]
rs7680825	4	10358790	*3′ of ZNF518B*	[1]
rs7681212	4	10358964	*3′ of ZNF518B*	[6]
rs13114042	4	10359104	*3′ of ZNF518B*	[1]
rs7661209	4	10359607	*3′ of ZNF518B*	[1]
rs11727261	4	10363482	*3′ of ZNF518B*	[1]
rs4235361	4	10365093	*3′ of ZNF518B*	[1]
rs4697753	4	10366453	*3′ of ZNF518B*	[1]
rs4698041	4	10368235	*3′ of ZNF518B*	[1]
rs9291406	4	10369564	*3′ of ZNF518B*	[1]
rs13122923	4	10369776	*3′ of ZNF518B*	[1]
rs6857135	4	10370585	*3′ of ZNF518B*	[1]
rs6813712	4	10370663	*3′ of ZNF518B*	[1]
rs6813919	4	10370791	*3′ of ZNF518B*	[1]
rs6849583	4	10372790	*3′ of ZNF518B*	[1]
rs13125564	4	10372914	*3′ of ZNF518B*	[1]
rs6851536	4	10372966	*3′ of ZNF518B*	[1]
rs11943393	4	10374005	*3′ of ZNF518B*	[1]
rs10938772	4	10375328	*3′ of ZNF518B*	[1]
rs11732092	4	10377405	*3′ of ZNF518B*	[11]
rs4698043	4	10378602	*3′ of ZNF518B*	[1]
rs7677806	4	10383005	*3′ of ZNF518B*	[6]
rs59420943	4	10384278	*3′ of ZNF518B*	[11]
rs4302456	4	10386674	*3′ of ZNF518B*	[1]
rs4302457	4	10386973	*3′ of ZNF518B*	[6]
rs9990427	4	10388313	*3′ of ZNF518B*	[11]
rs9990701	4	10388610	*3′ of ZNF518B*	[11]
rs11724536	4	10390289	*3′ of ZNF518B*	[1]

rs4607209	4	10400156	*3′ of ZNF518B*	[1]
rs10017305	4	10401223	*3′ of ZNF518B*	[1]
rs11943276	4	10403545	*3′ of ZNF518B*	[6]
rs7654258	4	10404512	*3′ of ZNF518B*	[1]
rs7677318	4	10406291	*3′ of ZNF518B*	[1]
rs4463062	4	10406994	*3′ of ZNF518B*	[6]
rs6819959	4	10407534	*3′ of ZNF518B*	[6]
rs4643800	4	10407572	*3′ of ZNF518B*	[13]
rs11728025	4	10408221	*3′ of ZNF518B*	[1]
rs7697246	4	10408757	*3′ of ZNF518B*	[1]
rs4698049	4	10409770	*3′ of ZNF518B*	[1]
rs7656072	4	10409841	*3′ of ZNF518B*	[1]
rs4698050	4	10410748	*3′ of ZNF518B*	[13]
rs11721485	4	10412554	*3′ of ZNF518B*	[1]
rs4610325	4	10413168	*3′ of ZNF518B*	[1]
rs7661365	4	10413908	*3′ of ZNF518B*	[1]
rs11736389	4	10416360	*3′ of ZNF518B*	[1]
rs13125855	4	10418078	*3′ of ZNF518B*	[1]
rs6858510	4	10432508	*3′ of ZNF518B*	[1]
rs7691990	4	10434459	*3′ of ZNF518B*	[1]
rs10003864	4	10437391	*3′ of ZNF518B*	[1]
rs4422413	4	10443384	*ZNF518B*	[1]
rs10938799	4	10443425	*ZNF518B*	[1]
rs3217	4	10444650	*ZNF518B*	[1]
rs10016022	4	10446906	*ZNF518B*	[12]
rs66538112	4	10447168	*ZNF518B*	[12]
rs10016702	4	10447640	*ZNF518B*	[1]
rs7674156	4	10452763	*ZNF518B*	[1]
rs12019277	4	10454994	*ZNF518B*	[1]
rs9996284	4	10469446	*5′ of ZNF518B*	[1]
rs10029818	4	10472670	*5′ of ZNF518B*	[1]
rs11732503	4	10482614	*3′ of CLNK*	[1]
rs11737650	4	10485014	*3′ of CLNK*	[1]

rs11733306	4	10488402	*CLNK*	[1]
rs11737601	4	10488568	*CLNK*	[1]
rs4541501	4	10488621	*CLNK*	[1]
rs13111270	4	10489007	*CLNK*	[1]
rs13130674	4	10489600	*CLNK*	[1]
rs7692088	4	10491040	*CLNK*	[1]
rs9790491	4	10493115	*CLNK*	[1]
rs13115661	4	10493961	*CLNK*	[1]
rs3749558	4	10494003	*CLNK*	[1]
rs7667644	4	10495072	*CLNK*	[1]
rs16869060	4	10495790	*CLNK*	[1]
rs13142053	4	10496241	*CLNK*	[1]
rs10033825	4	10496792	*CLNK*	[1]
rs7698826	4	10497423	*CLNK*	[1]
rs6819820	4	10498830	*CLNK*	[1]
rs12504795	4	10499344	*CLNK*	[1]
rs17467273	4	10500431	*CLNK*	[1]
rs13109939	4	10501625	*CLNK*	[1]
rs10488946	4	10502650	*CLNK*	[1]
rs6833095	4	10506599	*CLNK*	[1]
rs11734599	4	10507490	*CLNK*	[1]
rs887112	4	10507984	*CLNK*	[1]
rs13141385	4	10508404	*CLNK*	[1]
rs2868941	4	10509699	*CLNK*	[1]
rs11929850	4	10510050	*CLNK*	[1]
rs13108998	4	10510869	*CLNK*	[1]
rs13109005	4	10510881	*CLNK*	[1]
rs1004327	4	10511483	*CLNK*	[1]
rs12508358	4	10512238	*CLNK*	[1]
rs13125670	4	10513072	*CLNK*	[1]
rs12499142	4	10514238	*CLNK*	[1]
rs2868942	4	10514568	*CLNK*	[1]
rs16869379	4	10514681	*CLNK*	[1]

rs10034180	4	10518525	*CLNK*	[1]
rs16869430	4	10519426	*CLNK*	[1]
rs2041215	4	10519982	*CLNK*	[1]
rs7665423	4	10520600	*CLNK*	[1]
rs13125086	4	10520693	*CLNK*	[1]
rs16869474	4	10520972	*CLNK*	[1]
rs2012249	4	10521158	*CLNK*	[1]
rs2012237	4	10521221	*CLNK*	[1]
rs10001632	4	10521871	*CLNK*	[1]
rs2041216	4	10523090	*CLNK*	[1]
rs997219	4	10524671	*CLNK*	[1]
rs2108878	4	10527342	*CLNK*	[1]
rs12641877	4	10528226	*CLNK*	[1]
rs2286463	4	10532258	*CLNK*	[1]
rs2286465	4	10532634	*CLNK*	[1]
rs10030521	4	10536417	*CLNK*	[1]
rs10488948	4	10536544	*CLNK*	[1]
rs2108879	4	10538144	*CLNK*	[1]
rs7688620	4	10543318	*CLNK*	[1]
rs6856070	4	10545768	*CLNK*	[1]
rs11939512	4	10546359	*CLNK*	[1]
rs10938845	4	10551500	*CLNK*	[1]
rs1974584	4	10557861	*CLNK*	[1]
rs1558201	4	10557918	*CLNK*	[1]
rs4698068	4	10558992	*CLNK*	[1]
rs4698069	4	10559041	*CLNK*	[1]
rs9994202	4	10559396	*CLNK*	[1]
rs12643937	4	10577980	*CLNK*	[1]
rs12640119	4	10586384	*CLNK*	[1]
rs10488949	4	10591448	*CLNK*	[1]
rs2531178	4	10601134	*CLNK*	[1]
rs2531185	4	10605821	*CLNK*	[1]
rs6836007	4	10647584	*CLNK*	[1]

rs4593124	4	10648278	*CLNK*	[1]
rs12650571	4	10649456	*CLNK*	[1]
rs7696539	4	10649778	*CLNK*	[1]
rs10516207	4	10650693	*CLNK*	[1]
rs13107947	4	10651617	*CLNK*	[1]
rs13139842	4	10651701	*CLNK*	[1]
rs6822578	4	10652262	*CLNK*	[1]
rs4449408	4	10652413	*CLNK*	[1]
rs12233843	4	10655218	*CLNK*	[1]
rs10938919	4	10655503	*CLNK*	[1]
rs9683460	4	10656446	*CLNK*	[1]
rs6830367	4	10671323	*CLNK*	[1]
rs141646361	4	77676155	*SHROOM3*	[4]
rs17013187	4	88733531	*3′ of IBSP*	[23]
rs4466013	4	88760413	*MEPE*	[1]
rs17013282	4	88765873	*MEPE*	[23]
rs6854498	4	88776760	*3′ of MEPE*	[1]
rs6854361	4	88776864	*3′ of MEPE*	[1]
rs2169612	4	88817414	*3′ of MEPE*	[1]
rs12651696	4	88821873	*3′ of MEPE*	[1]
rs17013544	4	88823643	*3′ of MEPE*	[1]
rs17013545	4	88826327	*3′ of MEPE*	[1]
rs716202	4	88836002	*5′ of SPP1*	[1]
rs17842205	4	88849552	*5′ of SPP1*	[1]
rs10516796	4	88856733	*5′ of SPP1*	[1]
rs4693920	4	88864775	*5′ of SPP1*	[1]
rs2728123	4	88870795	*5′ of SPP1*	[1]
rs17013584	4	88872963	*5′ of SPP1*	[1]
rs2728119	4	88877531	*5′ of SPP1*	[1]
rs4490426	4	88880522	*5′ of SPP1*	[1]
rs10516798	4	88883537	*5′ of SPP1*	[1]
rs11730059	4	88887327	*5′ of SPP1*	[1]
rs12641001	4	88888940	*5′ of SPP1*	[1]

rs10516800	4	88891590	*5′ of SPP1*	[1]
rs6813526	4	88894235	*5′ of SPP1*	[1]
rs2853749	4	88897814	*SPP1*	[1]
rs7685225	4	88906458	*3′ of SPP1*	[1]
rs6818927	4	88907257	*3′ of SPP1*	[1]
rs4128340	4	88908136	*3′ of SPP1*	[1]
rs6532041	4	88909033	*3′ of SPP1*	[1]
rs6832511	4	88909088	*3′ of SPP1*	[1]
rs6838095	4	88909436	*3′ of SPP1*	[1]
rs12509864	4	88909766	*3′ of SPP1*	[1]
rs11938988	4	88910113	*3′ of SPP1*	[1]
rs2728116	4	88931050	*PKD2*	[1]
rs2725234	4	88932272	*PKD2*	[1]
rs12503776	4	88932464	*PKD2*	[1]
rs2728113	4	88939736	*PKD2*	[6]
rs2725227	4	88944511	*PKD2*	[1]
rs2467052	4	88949175	*PKD2*	[1]
rs13149278	4	88951390	*PKD2*	[1]
rs2725225	4	88952076	*PKD2*	[1]
rs2728110	4	88952883	*PKD2*	[1]
rs17786456	4	88957562	*PKD2*	[1]
rs2728109	4	88957723	*PKD2*	[6]
rs2725220	4	88959922	*PKD2*	[11]
rs7696304	4	88959998	*PKD2*	[1]
rs2725217	4	88960258	*PKD2*	[1]
rs2725215	4	88961571	*PKD2*	[6]
rs2725212	4	88968713	*PKD2*	[1]
rs2725211	4	88970375	*PKD2*	[23]
rs2728106	4	88972051	*PKD2*	[1]
rs2728104	4	88973006	*PKD2*	[1]
rs2725210	4	88973427	*PKD2*	[1]
rs2728099	4	88975738	*PKD2*	[6]
rs2725207	4	88979529	*PKD2*	[1]

rs2728133	4	88981690	*PKD2*	[1]
rs2728132	4	88982461	*PKD2*	[1]
rs2725205	4	88985711	*PKD2*	[1]
rs11938025	4	88987276	*PKD2*	[1]
rs4336187	4	88987916	*PKD2*	[1]
rs2725203	4	88995795	*PKD2*	[1]
rs2728121	4	88997102	*PKD2*	[1]
rs10965	4	88998083	*PKD2*	[1]
rs2728126	4	88999222	*3′ of PKD2*	[1]
rs2725201	4	88999306	*3′ of PKD2*	[11]
rs2728125	4	89001893	*3′ of PKD2*	[6]
rs199897813	4	89015728	*ABCG2*	[4]
rs2231164	4	89015857	*ABCG2*	[11]
rs2231156	4	89020427	*ABCG2*	[1]
rs4148157	4	89020934	*ABCG2*	[11]
rs4693924	4	89023224	*ABCG2*	[1]
rs2231148	4	89028478	*ABCG2*	[1]
rs2054576	4	89028775	*ABCG2*	[25]
rs12505410	4	89030841	*ABCG2*	[1]
rs2622621	4	89030920	*ABCG2*	[1]
rs13120400	4	89033527	*ABCG2*	[1]
rs1481012	4	89039082	*ABCG2*	[6]
rs2199936	4	89045331	*ABCG2*	[8]
rs2231142	4	89052323	*ABCG2*	[18]
rs4148155	4	89054667	*ABCG2*	[3]
rs4148152	4	89060909	*ABCG2*	[3]
rs13137622	4	89062513	*ABCG2*	[1]
rs3114018	4	89064581	*ABCG2*	[8]
rs3109823	4	89064602	*ABCG2*	[11]
rs6857600	4	89066075	*ABCG2*	[1]
rs2622626	4	89066715	*ABCG2*	[1]
rs6532049	4	89067526	*ABCG2*	[1]
rs17731799	4	89068455	*ABCG2*	[11]

rs2622624	4	89069406	*ABCG2*	[1]
rs2622604	4	89078924	*ABCG2*	[11]
rs2622605	4	89079386	*ABCG2*	[1]
rs3114020	4	89083666	*ABCG2*	[11]
rs2622609	4	89088475	*ABCG2*	[6]
rs10011796	4	89090877	*ABCG2*	[1]
rs10009618	4	89094008	*ABCG2*	[1]
rs2622629	4	89094064	*ABCG2*	[1]
rs1481014	4	89097151	*ABCG2*	[1]
rs1481017	4	89097477	*ABCG2*	[6]
rs11724427	4	89103172	*ABCG2*	[1]
rs6821227	4	89104688	*ABCG2*	[1]
rs6821239	4	89104751	*ABCG2*	[1]
rs9784454	4	89107490	*ABCG2*	[1]
rs6532053	4	89109259	*ABCG2*	[1]
rs2127863	4	89111468	*ABCG2*	[1]
rs4560364	4	89116178	*ABCG2*	[1]
rs13108900	4	89116702	*ABCG2*	[1]
rs10856870	4	89119659	*ABCG2*	[1]
rs4693930	4	89122833	*ABCG2*	[1]
rs10023457	4	89134704	*ABCG2*	[1]
rs6819328	4	89137318	*ABCG2*	[1]
rs2869736	4	89138377	*ABCG2*	[1]
rs4693935	4	89139275	*ABCG2*	[1]
rs2904185	4	89139832	*ABCG2*	[1]
rs4491984	4	89140356	*ABCG2*	[1]
rs1904903	4	89145274	*ABCG2*	[1]
rs9307048	4	89149570	*ABCG2*	[1]
rs10032109	4	89152063	*ABCG2*	[1]
rs4693941	4	89156899	*5′ of ABCG2*	[1]
rs1114568	4	89159819	*5′ of ABCG2*	[1]
rs13120254	4	89160561	*5′ of ABCG2*	[1]
rs13120819	4	89160677	*5′ of ABCG2*	[1]

rs6532058	4	89161902	*5′ of ABCG2*	[1]
rs997630	4	89163853	*5′ of ABCG2*	[1]
rs4693942	4	89165192	*5′ of ABCG2*	[1]
rs6532061	4	89165613	*5′ of ABCG2*	[1]
rs17013965	4	89170730	*3′ of PPM1K*	[1]
rs11729997	4	89171059	*3′ of PPM1K*	[1]
rs6853928	4	89172568	*3′ of PPM1K*	[1]
rs4693946	4	89219705	*RP11-10L7.1*	[1]
rs17013995	4	89227094	*RP11-10L7.1*	[1]
rs7676986	4	89227673	*RP11-10L7.1*	[1]
rs1545207	4	89239492	*RP11-10L7.1*	[1]
rs17014018	4	89248035	*RP11-10L7.1*	[1]
rs4693211	4	89249061	*RP11-10L7.1*	[1]
rs4693950	4	89249444	*RP11-10L7.1*	[1]
rs12512051	4	89252068	*RP11-10L7.1*	[1]
rs151134704	4	103537592	*NFKB1*	[4]
rs75869162	5	16617922	*CTC-461F20.1*	[26]
rs151305324	5	72419433	*TMEM171*	[4]
rs17632159	5	72431482	*RP11-232L2.2*	[1]
rs575416	5	72437534	*RP11-232L2.2*	[1]
rs622704	5	72439029	*RP11-232L2.2*	[1]
rs636266	5	72439774	*RP11-232L2.2*	[1]
rs484573	5	72441107	*RP11-232L2.2*	[1]
rs587189	5	72441476	*RP11-232L2.2*	[1]
rs527511	5	72446334	*RP11-232L2.2*	[1]
rs522152	5	72452355	*5′ of AC116345.1*	[1]
rs814174	6	7025868	*3′ of snoU13*	[1]
rs3863225	6	7032317	*3′ of snoU13*	[1]
rs9505008	6	7033860	*3′ of snoU13*	[1]
rs13212734	6	7037637	*3′ of snoU13*	[1]
rs10458103	6	7037738	*3′ of snoU13*	[1]
rs13202703	6	7038265	*3′ of snoU13*	[1]
rs13207230	6	7041700	*5′ of snoU13*	[1]

rs17584570	6	7044167	*5′ of snoU13*	[1]
rs1286005	6	7046452	*5′ of snoU13*	[1]
rs6929119	6	7046594	*5′ of snoU13*	[1]
rs9328398	6	7046763	*5′ of snoU13*	[1]
rs1286003	6	7046998	*5′ of snoU13*	[1]
rs560131	6	7047411	*5′ of snoU13*	[1]
rs7743312	6	7049929	*5′ of snoU13*	[1]
rs7769136	6	7052225	*5′ of snoU13*	[1]
rs592377	6	7060902	*5′ of snoU13*	[1]
rs2148088	6	7061912	*5′ of snoU13*	[1]
rs6936076	6	7063271	*5′ of snoU13*	[1]
rs2326878	6	7075094	*5′ of RREB1*	[1]
rs611555	6	7075822	*5′ of RREB1*	[1]
rs6912908	6	7080522	*5′ of RREB1*	[1]
rs501510	6	7081840	*5′ of RREB1*	[1]
rs9405325	6	7082633	*5′ of RREB1*	[1]
rs13213992	6	7083218	*5′ of RREB1*	[1]
rs4959424	6	7084857	*5′ of RREB1*	[1]
rs675209	6	7102084	*5′ of RREB1*	[1]
rs2842895	6	7106316	*5′ of RREB1*	[1]
rs1285874	6	7115533	*RREB1*	[1]
rs1285875	6	7115927	*RREB1*	[1]
rs11755724	6	7118990	*RREB1*	[1]
rs6933716	6	7125709	*RREB1*	[1]
rs687467	6	7128076	*RREB1*	[1]
rs622404	6	7130596	*RREB1*	[1]
rs630258	6	7134401	*RREB1*	[1]
rs1285879	6	7137363	*RREB1*	[1]
rs665723	6	7139064	*RREB1*	[1]
rs4585612	6	7153152	*RREB1*	[1]
rs1334577	6	7211751	*RREB1*	[1]
rs17762454	6	7213200	*RREB1*	[1]
rs143231463	6	7226810	*RREB1*	[4]

rs141635364	6	7231115	*RREB1*	[4]
rs301395	6	25486626	*LRRC16A*	[1]
rs4712944	6	25488265	*LRRC16A*	[1]
rs12528639	6	25491807	*LRRC16A*	[1]
rs1034050	6	25492364	*LRRC16A*	[1]
rs12526321	6	25494503	*LRRC16A*	[1]
rs17252870	6	25494666	*LRRC16A*	[1]
rs301393	6	25500709	*LRRC16A*	[1]
rs17253044	6	25503735	*LRRC16A*	[1]
rs407934	6	25504562	*LRRC16A*	[1]
rs1747568	6	25506072	*LRRC16A*	[1]
rs572657	6	25513147	*LRRC16A*	[1]
rs1408280	6	25513240	*LRRC16A*	[1]
rs1830798	6	25513276	*LRRC16A*	[1]
rs301390	6	25514216	*LRRC16A*	[1]
rs301383	6	25515234	*LRRC16A*	[1]
rs301382	6	25515450	*LRRC16A*	[1]
rs301381	6	25515576	*LRRC16A*	[1]
rs150551	6	25533930	*LRRC16A*	[1]
rs969297	6	25543639	*LRRC16A*	[1]
rs2064128	6	25550139	*LRRC16A*	[1]
rs169946	6	25552143	*LRRC16A*	[1]
rs3804133	6	25552868	*LRRC16A*	[1]
rs214055	6	25560264	*LRRC16A*	[1]
rs3788994	6	25587836	*LRRC16A*	[1]
rs13212936	6	25588815	*LRRC16A*	[1]
rs1028318	6	25590224	*LRRC16A*	[1]
rs2077393	6	25607365	*LRRC16A*	[8]
rs742132	6	25607571	*LRRC16A*	[3]
rs6903765	6	25609738	*LRRC16A*	[1]
rs6908390	6	25609756	*LRRC16A*	[1]
rs3804105	6	25612683	*LRRC16A*	[1]
rs10946785	6	25617068	*LRRC16A*	[1]

rs1997672	6	25617544	*LRRC16A*	[1]
rs7761700	6	25618191	*LRRC16A*	[1]
rs2281070	6	25622116	*3′ of LRRC16A*	[1]
rs12183240	6	25624416	*3′ of LRRC16A*	[1]
rs12201186	6	25625595	*3′ of LRRC16A*	[1]
rs10223506	6	25627216	*3′ of LRRC16A*	[1]
rs9467538	6	25628688	*3′ of LRRC16A*	[1]
rs2223323	6	25628852	*3′ of LRRC16A*	[1]
rs2205933	6	25628924	*3′ of LRRC16A*	[1]
rs1980449	6	25629658	*3′ of LRRC16A*	[1]
rs1980450	6	25629747	*3′ of LRRC16A*	[1]
rs1980451	6	25630091	*3′ of LRRC16A*	[1]
rs1980452	6	25630136	*3′ of LRRC16A*	[1]
rs9467541	6	25630549	*3′ of LRRC16A*	[1]
rs2072844	6	25632041	*3′ of LRRC16A*	[1]
rs4140641	6	25635400	*3′ of LRRC16A*	[1]
rs11969981	6	25650817	*5′ of SCGN*	[1]
rs12528182	6	25651197	*5′ of SCGN*	[1]
rs12524760	6	25651263	*5′ of SCGN*	[1]
rs4140640	6	25651650	*5′ of SCGN*	[1]
rs2294344	6	25652225	*5′ of SCGN*	[1]
rs2072846	6	25655104	*SCGN*	[1]
rs10484591	6	25656661	*SCGN*	[1]
rs4712950	6	25657590	*SCGN*	[1]
rs2205936	6	25685492	*SCGN*	[1]
rs9366627	6	25686405	*SCGN*	[1]
rs9379778	6	25687579	*SCGN*	[1]
rs4711093	6	25687895	*SCGN*	[1]
rs2294346	6	25689487	*SCGN*	[1]
rs17492659	6	25691362	*SCGN*	[1]
rs4419666	6	25693274	*SCGN*	[1]
rs4409177	6	25694491	*SCGN*	[1]
rs3922681	6	25698481	*SCGN*	[1]

rs9467570	6	25699283	*SCGN*	[1]
rs11794	6	25701718	*SCGN*	[1]
rs6908713	6	25703611	*3′ of SCGN*	[1]
rs9467573	6	25704190	*3′ of SCGN*	[1]
rs9467574	6	25705718	*3′ of SCGN*	[1]
rs4711094	6	25706174	*3′ of SCGN*	[1]
rs4395714	6	25708714	*3′ of SCGN*	[1]
rs6456688	6	25708960	*3′ of SCGN*	[1]
rs6456693	6	25710087	*3′ of SCGN*	[1]
rs6912391	6	25710763	*3′ of SCGN*	[1]
rs6456694	6	25712894	*3′ of SCGN*	[1]
rs4236036	6	25712985	*3′ of SCGN*	[1]
rs9379782	6	25715023	*3′ of HIST1H2AA*	[1]
rs4132072	6	25717843	*3′ of HIST1H2AA*	[1]
rs9461204	6	25718466	*3′ of HIST1H2AA*	[1]
rs7450798	6	25719280	*3′ of HIST1H2AA*	[1]
rs9348692	6	25720812	*3′ of HIST1H2AA*	[1]
rs9379783	6	25725207	*3′ of HIST1H2AA*	[1]
rs9379784	6	25725506	*3′ of HIST1H2AA*	[1]
rs12215823	6	25726074	*3′ of HIST1H2AA*	[1]
rs9379785	6	25726213	*3′ of HIST1H2AA*	[1]
rs9358871	6	25726675	*HIST1H2AA*	[1]
rs4711095	6	25726774	*HIST1H2AA*	[1]
rs4711096	6	25727054	*5′ of HIST1H2BA*	[1]
rs4712959	6	25727057	*5′ of HIST1H2BA*	[1]
rs4712960	6	25727265	*HIST1H2BA*	[1]
rs4712961	6	25727334	*HIST1H2BA*	[1]
rs9358872	6	25727517	*HIST1H2BA*	[1]
rs17320558	6	25727527	*HIST1H2BA*	[1]
rs17267614	6	25730027	*3′ of HIST1H2BA*	[1]
rs9379786	6	25730255	*3′ of HIST1H2BA*	[1]
rs9358873	6	25731783	*3′ of HIST1H2BA*	[1]
rs4711097	6	25732314	*3′ of HIST1H2BA*	[1]

rs4464787	6	25733558	*3′ of HIST1H2BA*	[1]
rs9461210	6	25734073	*3′ of HIST1H2BA*	[1]
rs4360128	6	25734558	*3′ of HIST1H2BA*	[1]
rs3923725	6	25734920	*3′ of HIST1H2BA*	[1]
rs3922699	6	25734969	*3′ of HIST1H2BA*	[1]
rs6924794	6	25735874	*3′ of HIST1H2BA*	[1]
rs3922842	6	25736564	*3′ of HIST1H2BA*	[1]
rs9358875	6	25738008	*3′ of HIST1H2BA*	[1]
rs9358876	6	25738956	*3′ of HIST1H2BA*	[1]
rs9393661	6	25739080	*3′ of HIST1H2BA*	[1]
rs9356986	6	25739119	*3′ of HIST1H2BA*	[1]
rs9356987	6	25739187	*3′ of HIST1H2BA*	[1]
rs9358877	6	25739228	*3′ of HIST1H2BA*	[1]
rs12663099	6	25739518	*3′ of HIST1H2BA*	[1]
rs9393662	6	25739670	*3′ of HIST1H2BA*	[1]
rs9358878	6	25740273	*3′ of HIST1H2BA*	[1]
rs4712963	6	25740502	*3′ of HIST1H2BA*	[1]
rs9366629	6	25741216	*3′ of HIST1H2BA*	[1]
rs6456697	6	25741434	*5′ of SLC17A4*	[1]
rs4711103	6	25742224	*5′ of SLC17A4*	[1]
rs9467591	6	25742699	*5′ of SLC17A4*	[1]
rs9358880	6	25743161	*5′ of SLC17A4*	[1]
rs9393665	6	25744284	*5′ of SLC17A4*	[1]
rs1937131	6	25744831	*5′ of SLC17A4*	[1]
rs7754296	6	25744878	*5′ of SLC17A4*	[1]
rs7754733	6	25745049	*5′ of SLC17A4*	[1]
rs7754814	6	25745243	*5′ of SLC17A4*	[1]
rs7775354	6	25745266	*5′ of SLC17A4*	[1]
rs9379789	6	25745579	*5′ of SLC17A4*	[1]
rs6941933	6	25745702	*5′ of SLC17A4*	[1]
rs6923367	6	25745852	*5′ of SLC17A4*	[1]
rs2154219	6	25749957	*5′ of SLC17A4*	[1]
rs10498728	6	25751320	*5′ of SLC17A4*	[1]

rs12207270	6	25751721	*5′ of SLC17A4*	[1]
rs9348694	6	25753640	*5′ of SLC17A4*	[1]
rs1892256	6	25754271	*5′ of SLC17A4*	[1]
rs13194155	6	25755508	*SLC17A4*	[1]
rs16890999	6	25755583	*SLC17A4*	[1]
rs9358886	6	25758253	*SLC17A4*	[1]
rs17268697	6	25758448	*SLC17A4*	[1]
rs2000351	6	25759783	*SLC17A4*	[1]
rs7770037	6	25760591	*SLC17A4*	[1]
rs4236040	6	25761209	*SLC17A4*	[1]
rs4712969	6	25764192	*SLC17A4*	[1]
rs2186087	6	25764842	*SLC17A4*	[1]
rs1317816	6	25765390	*SLC17A4*	[1]
rs6937800	6	25765943	*SLC17A4*	[1]
rs6902211	6	25767437	*SLC17A4*	[1]
rs3778272	6	25767661	*SLC17A4*	[1]
rs2275904	6	25768085	*SLC17A4*	[1]
rs2275905	6	25768106	*SLC17A4*	[1]
rs1892248	6	25768914	*SLC17A4*	[1]
rs1892250	6	25769024	*SLC17A4*	[1]
rs1892251	6	25769349	*SLC17A4*	[1]
rs2328892	6	25769510	*SLC17A4*	[1]
rs1937127	6	25769872	*SLC17A4*	[1]
rs2328893	6	25770239	*SLC17A4*	[1]
rs4712970	6	25770707	*SLC17A4*	[1]
rs4712971	6	25771900	*SLC17A4*	[1]
rs4712972	6	25772047	*SLC17A4*	[1]
rs1892252	6	25772639	*SLC17A4*	[1]
rs2275906	6	25773809	*SLC17A4*	[1]
rs6456701	6	25774130	*SLC17A4*	[1]
rs17269374	6	25775242	*SLC17A4*	[1]
rs1954598	6	25776060	*SLC17A4*	[1]
rs2154218	6	25776290	*SLC17A4*	[1]

rs11754288	6	25776949	*SLC17A4*	[12]
rs9356988	6	25777481	*SLC17A4*	[1]
rs3949215	6	25777497	*SLC17A4*	[1]
rs12201071	6	25778055	*SLC17A4*	[1]
rs1317510	6	25778924	*SLC17A4*	[1]
rs1141034	6	25780332	*SLC17A4*	[1]
rs6910549	6	25780811	*SLC17A4*	[1]
rs7749149	6	25781139	*SLC17A4*	[1]
rs10946798	6	25781853	*3′ of SLC17A4*	[1]
rs1892253	6	25782314	*3′ of SLC17A1*	[1]
rs3923	6	25783315	*SLC17A1*	[1]
rs3757131	6	25783909	*SLC17A1*	[1]
rs12662869	6	25784481	*SLC17A1*	[1]
rs13197601	6	25785935	*SLC17A1*	[1]
rs13213957	6	25786226	*SLC17A1*	[1]
rs3799344	6	25786993	*SLC17A1*	[1]
rs2096386	6	25787817	*SLC17A1*	[1]
rs9393670	6	25789061	*SLC17A1*	[1]
rs9393671	6	25789189	*SLC17A1*	[1]
rs10214468	6	25789390	*SLC17A1*	[1]
rs13200921	6	25790378	*SLC17A1*	[1]
rs3799346	6	25791354	*SLC17A1*	[1]
rs1165157	6	25792250	*SLC17A1*	[1]
rs942377	6	25792292	*SLC17A1*	[1]
rs1165156	6	25792978	*SLC17A1*	[1]
rs12209856	6	25793673	*SLC17A1*	[1]
rs2762353	6	25794431	*SLC17A1*	[1]
rs1165155	6	25795577	*SLC17A1*	[1]
rs1165215	6	25798932	*SLC17A1*	[1]
rs1165211	6	25800922	*SLC17A1*	[1]
rs1165209	6	25801319	*SLC17A1*	[27]
rs1324082	6	25801971	*SLC17A1*	[1]
rs12191655	6	25803095	*SLC17A1*	[1]

rs1165208	6	25803904	*SLC17A1*	[1]
rs9467604	6	25805002	*SLC17A1*	[1]
rs9461216	6	25808743	*SLC17A1*	[1]
rs9467606	6	25809218	*SLC17A1*	[1]
rs9467609	6	25809751	*SLC17A1*	[1]
rs1359231	6	25809798	*SLC17A1*	[1]
rs1575535	6	25809909	*SLC17A1*	[1]
rs1575534	6	25811404	*SLC17A1*	[1]
rs10498730	6	25812069	*SLC17A1*	[1]
rs942378	6	25812459	*SLC17A1*	[1]
rs9467613	6	25812641	*SLC17A1*	[1]
rs1165196	6	25813150	*SLC17A1*	[2]
rs7753366	6	25817518	*SLC17A1*	[1]
rs1165153	6	25817789	*SLC17A1*	[1]
rs1185567	6	25818588	*SLC17A1*	[1]
rs1183200	6	25818646	*SLC17A1*	[1]
rs12182983	6	25818755	*SLC17A1*	[1]
rs1165152	6	25818766	*SLC17A1*	[1]
rs6913879	6	25820428	*SLC17A1*	[1]
rs17270561	6	25820439	*SLC17A1*	[1]
rs6939997	6	25821224	*SLC17A1*	[1]
rs6940698	6	25821580	*SLC17A1*	[1]
rs1165151	6	25821616	*SLC17A1*	[1]
rs1185976	6	25822363	*SLC17A1*	[1]
rs3799352	6	25822620	*SLC17A1*	[1]
rs13201341	6	25822661	*SLC17A1*	[1]
rs1183201	6	25823444	*SLC17A1*	[8]
rs12211184	6	25823774	*SLC17A1*	[1]
rs1165181	6	25825390	*SLC17A1*	[1]
rs6456703	6	25826119	*SLC17A1*	[1]
rs1408268	6	25826986	*SLC17A1*	[1]
rs1165178	6	25827516	*SLC17A1*	[1]
rs765285	6	25828242	*SLC17A1*	[1]

rs13199775	6	25828782	*SLC17A1*	[1]
rs12200962	6	25828986	*SLC17A1*	[1]
rs13200784	6	25829633	*SLC17A1*	[1]
rs1165177	6	25829659	*SLC17A1*	[1]
rs1165176	6	25830298	*SLC17A1*	[1]
rs2070642	6	25831212	*SLC17A1*	[1]
rs1185569	6	25831603	*SLC17A1*	[1]
rs1185568	6	25834428	*SLC17A3*	[1]
rs1184803	6	25834658	*SLC17A3*	[1]
rs1185978	6	25835895	*SLC17A3*	[1]
rs9461219	6	25836927	*SLC17A3*	[1]
rs1165182	6	25837829	*SLC17A3*	[1]
rs6905614	6	25840485	*SLC17A3*	[1]
rs1408273	6	25840946	*SLC17A3*	[1]
rs1324088	6	25841122	*SLC17A3*	[1]
rs1324087	6	25841408	*SLC17A3*	[1]
rs4712976	6	25842203	*SLC17A3*	[1]
rs9393672	6	25842605	*SLC17A3*	[8]
rs3887266	6	25843746	*SLC17A3*	[1]
rs1165148	6	25844710	*SLC17A3*	[1]
rs942379	6	25849620	*SLC17A3*	[8]
rs1165189	6	25849779	*SLC17A3*	[1]
rs9467621	6	25851338	*SLC17A3*	[1]
rs1165187	6	25851369	*SLC17A3*	[1]
rs9467622	6	25854644	*SLC17A3*	[1]
rs1780969	6	25858432	*SLC17A3*	[1]
rs1179087	6	25858704	*SLC17A3*	[1]
rs1177441	6	25859413	*SLC17A3*	[1]
rs1165168	6	25859508	*SLC17A3*	[1]
rs1182814	6	25859554	*SLC17A3*	[1]
rs1408271	6	25859621	*SLC17A3*	[1]
rs1165167	6	25860688	*SLC17A3*	[1]
rs1165165	6	25862466	*SLC17A3*	[1]

rs1165164	6	25863481	*SLC17A3*	[1]
rs1165162	6	25863605	*SLC17A3*	[1]
rs17271121	6	25863650	*SLC17A3*	[1]
rs1165161	6	25864362	*SLC17A3*	[1]
rs1165160	6	25864456	*SLC17A3*	[1]
rs1165159	6	25864625	*SLC17A3*	[1]
rs13211947	6	25864818	*SLC17A3*	[1]
rs1184498	6	25864882	*SLC17A3*	[1]
rs1165158	6	25864898	*SLC17A3*	[1]
rs1165207	6	25865266	*SLC17A3*	[1]
rs1165206	6	25867566	*SLC17A3*	[1]
rs1184804	6	25868226	*SLC17A3*	[1]
rs548987	6	25869371	*SLC17A3*	[1]
rs523383	6	25869848	*SLC17A3*	[1]
rs1165205	6	25870542	*SLC17A3*	[3]
rs555460	6	25870655	*SLC17A3*	[1]
rs556339	6	25870745	*SLC17A3*	[1]
rs972087	6	25872579	*SLC17A3*	[1]
rs501220	6	25873025	*SLC17A3*	[1]
rs9467626	6	25873746	*SLC17A3*	[1]
rs13198474	6	25874423	*SLC17A3*	[1]
rs1165201	6	25874823	*SLC17A3*	[1]
rs12664474	6	25876089	*SLC17A3*	[1]
rs603089	6	25877970	*SLC17A3*	[1]
rs11964886	6	25878379	*SLC17A3*	[1]
rs11969868	6	25878506	*SLC17A3*	[1]
rs629835	6	25879330	*SLC17A3*	[1]
rs645279	6	25880494	*SLC17A3*	[1]
rs531750	6	25882642	*5′ of SLC17A3*	[1]
rs599444	6	25883714	*5′ of SLC17A3*	[1]
rs629444	6	25885814	*5′ of SLC17A3*	[1]
rs537581	6	25886497	*5′ of SLC17A3*	[1]
rs483906	6	25888094	*5′ of SLC17A3*	[1]

rs9379800	6	25889099	*5′ of SLC17A3*	[1]
rs518700	6	25889553	*5′ of SLC17A3*	[1]
rs9467632	6	25889706	*5′ of SLC17A3*	[1]
rs6910741	6	25895575	*5′ of SLC17A3*	[1]
rs13220488	6	25896907	*5′ of SLC17A3*	[1]
rs428469	6	25898624	*3′ of SLC17A2*	[1]
rs16891142	6	25899139	*3′ of SLC17A2*	[1]
rs13207673	6	25901133	*3′ of SLC17A2*	[1]
rs9379801	6	25901711	*3′ of SLC17A2*	[1]
rs9356991	6	25901758	*3′ of SLC17A2*	[1]
rs6910138	6	25904352	*3′ of SLC17A2*	[1]
rs7748167	6	25904652	*3′ of SLC17A2*	[1]
rs9461222	6	25905111	*3′ of SLC17A2*	[1]
rs9467635	6	25909166	*3′ of SLC17A2*	[1]
rs3799371	6	25912816	*3′ of SLC17A2*	[1]
rs6932113	6	25913098	*SLC17A2*	[1]
rs6938233	6	25914077	*SLC17A2*	[1]
rs2071301	6	25914263	*SLC17A2*	[1]
rs1865760	6	25916979	*SLC17A2*	[1]
rs9295675	6	25918473	*SLC17A2*	[1]
rs3752421	6	25918688	*SLC17A2*	[1]
rs17526722	6	25918855	*SLC17A2*	[1]
rs13195279	6	25919431	*SLC17A2*	[1]
rs9467636	6	25919549	*SLC17A2*	[1]
rs7749342	6	25920265	*SLC17A2*	[1]
rs9358893	6	25921761	*SLC17A2*	[1]
rs3799372	6	25922311	*SLC17A2*	[1]
rs1540273	6	25924158	*SLC17A2*	[1]
rs2071297	6	25924513	*SLC17A2*	[1]
rs7770139	6	25925823	*SLC17A2*	[1]
rs3734523	6	25925987	*SLC17A2*	[1]
rs10484431	6	25926674	*SLC17A2*	[1]
rs17586553	6	25928775	*SLC17A2*	[1]

rs3799373	6	25929173	*SLC17A2*	[1]
rs199741	6	25931577	*5′ of SLC17A2*	[1]
rs199737	6	25933538	*5′ of SLC17A2*	[1]
rs442601	6	25934524	*5′ of SLC17A2*	[1]
rs9461224	6	25936402	*5′ of SLC17A2*	[1]
rs199736	6	25936787	*5′ of SLC17A2*	[1]
rs9393676	6	25936944	*5′ of SLC17A2*	[1]
rs9295678	6	25937033	*5′ of SLC17A2*	[1]
rs1436307	6	25939723	*5′ of SLC17A2*	[1]
rs199734	6	25940393	*5′ of SLC17A2*	[1]
rs9379806	6	25940958	*5′ of SLC17A2*	[1]
rs9358896	6	25943197	*5′ of SLC17A2*	[1]
rs9295680	6	25944103	*5′ of SLC17A2*	[1]
rs9295681	6	25944117	*5′ of SLC17A2*	[1]
rs2051541	6	25945211	*5′ of SLC17A2*	[1]
rs1436306	6	25948421	*5′ of TRIM38*	[1]
rs1541987	6	25950397	*5′ of TRIM38*	[1]
rs199726	6	25953360	*5′ of TRIM38*	[1]
rs2876693	6	25954434	*5′ of TRIM38*	[1]
rs9467646	6	25957408	*5′ of TRIM38*	[1]
rs9467647	6	25957642	*5′ of TRIM38*	[1]
rs10946800	6	25957773	*5′ of TRIM38*	[1]
rs12195653	6	25958621	*5′ of TRIM38*	[1]
rs9467652	6	25959127	*5′ of TRIM38*	[1]
rs199739	6	25960509	*5′ of TRIM38*	[1]
rs129129	6	25961029	*5′ of TRIM38*	[1]
rs12210098	6	25963966	*TRIM38*	[1]
rs1436310	6	25969958	*TRIM38*	[1]
rs1436309	6	25970109	*TRIM38*	[1]
rs115810	6	25975883	*TRIM38*	[1]
rs1130000	6	25985396	*3′ of TRIM38*	[1]
rs2032450	6	25993114	*3′ of TRIM38*	[1]
rs9467656	6	25993559	*3′ of TRIM38*	[1]

rs2013063	6	25994098	*3′ of TRIM38*	[1]
rs12216125	6	25997458	*3′ of TRIM38*	[1]
rs199753	6	26001888	*3′ of HIST1H1A*	[1]
rs9393681	6	26008260	*3′ of HIST1H1A*	[1]
rs9467658	6	26010881	*3′ of HIST1H1A*	[1]
rs199752	6	26012875	*3′ of HIST1H1A*	[1]
rs199751	6	26015583	*3′ of HIST1H1A*	[1]
rs199750	6	26016462	*3′ of HIST1H1A*	[1]
rs9461230	6	26019240	*5′ of HIST1H1A*	[1]
rs2157050	6	26020431	*5′ of HIST1H3A*	[1]
rs9467664	6	26021813	*5′ of HIST1H4A*	[1]
rs9379818	6	26023206	*3′ of HIST1H4A*	[1]
rs9358901	6	26024436	*3′ of HIST1H4A*	[1]
rs2032449	6	26026599	*3′ of HIST1H4B*	[1]
rs3752419	6	26027433	*HIST1H4B*	[1]
rs1540276	6	26028819	*5′ of HIST1H4B*	[1]
rs2213284	6	26031868	*HIST1H3B*	[1]
rs2230655	6	26033506	*HIST1H2AB*	[1]
rs4401650	6	26035208	*5′ of HIST1H2AB*	[1]
rs1540275	6	26036476	*5′ of HIST1H2AB*	[1]
rs7753826	6	26042239	*3′ of HIST1H2BB*	[1]
rs2032447	6	26044369	*5′ of HIST1H2BB*	[1]
rs7756117	6	26046565	*3′ of HIST1H3C*	[1]
rs7772312	6	26049616	*3′ of HIST1H3C*	[1]
rs9379820	6	26049924	*3′ of HIST1H3C*	[1]
rs10425	6	26056549	*HIST1H1C*	[1]
rs807214	6	26061769	*5′ of HIST1H1C*	[1]
rs9358903	6	26061949	*5′ of HIST1H1C*	[1]
rs807212	6	26065621	*5′ of HIST1H1C*	[1]
rs9295684	6	26069669	*5′ of HIST1H1C*	[1]
rs9295685	6	26071725	*5′ of HIST1H1C*	[1]
rs9295687	6	26082710	*5′ of HFE*	[1]
rs4529296	6	26083135	*5′ of HFE*	[1]

rs9379825	6	26083871	*5′ of HFE*	[1]
rs9467672	6	26083904	*5′ of HFE*	[1]
rs9295688	6	26084217	*5′ of HFE*	[1]
rs2006736	6	26086017	*5′ of HFE*	[1]
rs1800702	6	26086463	*5′ of HFE*	[1]
rs2794720	6	26087202	*5′ of HFE*	[1]
rs2794719	6	26088890	*HFE*	[1]
rs2071303	6	26091336	*HFE*	[1]
rs1572982	6	26094367	*HFE*	[1]
rs12346	6	26097046	*HFE*	[1]
rs6918586	6	26097384	*HFE*	[1]
rs1150660	6	26101439	*5′ of HIST1H4C*	[1]
rs198857	6	26102417	*5′ of HIST1H4C*	[1]
rs198856	6	26102708	*5′ of HIST1H4C*	[1]
rs198854	6	26104057	*5′ of HIST1H4C*	[1]
rs198853	6	26104096	*5′ of HIST1H4C*	[1]
rs198852	6	26104448	*HIST1H4C*	[1]
rs198848	6	26106325	*3′ of HIST1H1T*	[1]
rs198845	6	26107790	*HIST1H1T*	[1]
rs198844	6	26108282	*HIST1H1T*	[1]
rs198838	6	26113340	*3′ of HIST1H2BC*	[1]
rs198837	6	26113398	*3′ of HIST1H2BC*	[1]
rs198836	6	26113616	*3′ of HIST1H2BC*	[1]
rs198834	6	26114372	*3′ of HIST1H2BC*	[1]
rs13161	6	26114702	*3′ of HIST1H2BC*	[1]
rs198828	6	26119459	*HIST1H2BC*	[1]
rs198827	6	26121002	*HIST1H2BC*	[1]
rs198826	6	26121153	*HIST1H2BC*	[1]
rs198825	6	26122502	*HIST1H2BC*	[1]
rs198821	6	26123629	*HIST1H2BC*	[1]
rs198820	6	26124243	*5′ of HIST1H2BC*	[1]
rs198815	6	26127271	*HIST1H2AC*	[1]
rs198814	6	26127759	*HIST1H2AC*	[1]

rs198809	6	26128766	*HIST1H2AC*	[1]
rs138551969	6	43748486	*VEGFA*	[4]
rs729761	6	43804571	*3′ of RP11-344J7.2*	[1]
rs2396083	6	43804808	*3′ of RP11-344J7.2*	[1]
rs744103	6	43805362	*3′ of RP11-344J7.2*	[1]
rs881858	6	43806609	*3′ of RP11-344J7.2*	[1]
rs9472135	6	43809802	*3′ of RP11-344J7.2*	[1]
rs9369425	6	43810974	*3′ of RP11-344J7.2*	[1]
rs9369427	6	43811430	*3′ of RP11-344J7.2*	[1]
rs9472138	6	43811762	*3′ of RP11-344J7.2*	[1]
rs10456526	6	43814625	*3′ of RP11-344J7.2*	[1]
rs6937438	6	43815364	*3′ of RP11-344J7.2*	[1]
rs9321453	6	134773554	*RP11-557H15.3*	[28]
rs2240466	7	72856269	*BAZ1B*	[1]
rs1178979	7	72856430	*BAZ1B*	[1]
rs1178977	7	72857049	*BAZ1B*	[1]
rs714052	7	72864869	*BAZ1B*	[1]
rs2074755	7	72877166	*BAZ1B*	[1]
rs12056034	7	72878645	*BAZ1B*	[1]
rs6976930	7	72885810	*BAZ1B*	[1]
rs200548390	7	72892517	*BAZ1B*	[4]
rs17145713	7	72904810	*BAZ1B*	[1]
rs13244268	7	72911843	*BAZ1B*	[1]
rs7811265	7	72934510	*BAZ1B*	[1]
rs11983997	7	72939244	*5′ of BAZ1B*	[1]
rs17145732	7	72970268	*BCL7B*	[1]
rs13233571	7	72971231	*BCL7B*	[1]
rs12539316	7	72977898	*5′ of BCL7B*	[1]
rs17145738	7	72982874	*3′ of TBL2*	[1]
rs13232120	7	72983310	*3′ of TBL2*	[1]
rs14415	7	72984780	*TBL2*	[1]
rs2286276	7	72987354	*TBL2*	[1]
rs11974409	7	72989390	*TBL2*	[1]

rs1051921	7	73007943	*MLXIPL*	[1]
rs17145750	7	73026378	*MLXIPL*	[1]
rs7798357	7	73032265	*MLXIPL*	[1]
rs7785479	7	73032835	*MLXIPL*	[1]
rs7800944	7	73035857	*MLXIPL*	[29]
rs799166	7	73051932	*5′ of MLXIPL*	[1]
rs10480300	7	151406005	*PRKAG2*	[1]
rs17786744	8	23777006	*5′ of STC1*	[1]
rs2941454	8	76442347	*HNF4G*	[1]
rs1515016	8	76444129	*HNF4G*	[1]
rs1515017	8	76444404	*HNF4G*	[1]
rs1473951	8	76445102	*HNF4G*	[1]
rs938343	8	76446033	*HNF4G*	[1]
rs2006717	8	76446509	*HNF4G*	[1]
rs2941462	8	76447633	*HNF4G*	[1]
rs2977926	8	76448847	*HNF4G*	[1]
rs2943543	8	76448956	*HNF4G*	[1]
rs2943547	8	76451098	*HNF4G*	[1]
rs2977928	8	76451580	*HNF4G*	[1]
rs2943549	8	76452313	*HNF4G*	[1]
rs2943551	8	76452421	*HNF4G*	[1]
rs2943552	8	76452939	*HNF4G*	[1]
rs2943553	8	76453492	*HNF4G*	[1]
rs2977932	8	76456641	*HNF4G*	[1]
rs2943554	8	76458962	*HNF4G*	[1]
rs1800922	8	76459726	*HNF4G*	[1]
rs2977937	8	76460589	*HNF4G*	[1]
rs2977938	8	76461226	*HNF4G*	[1]
rs2056090	8	76462397	*HNF4G*	[1]
rs2272667	8	76463397	*HNF4G*	[1]
rs2943592	8	76464840	*HNF4G*	[1]
rs1800924	8	76465522	*HNF4G*	[1]
rs2941465	8	76465909	*HNF4G*	[1]

rs2943591	8	76465910	*HNF4G*	[1]
rs2941468	8	76468139	*HNF4G*	[1]
rs1805098	8	76468282	*HNF4G*	[1]
rs2941469	8	76469031	*HNF4G*	[1]
rs2941470	8	76470020	*HNF4G*	[1]
rs2941471	8	76470404	*HNF4G*	[1]
rs2941473	8	76472560	*HNF4G*	[1]
rs2943611	8	76473939	*HNF4G*	[1]
rs2941475	8	76474404	*HNF4G*	[1]
rs2943612	8	76474559	*HNF4G*	[1]
rs2977944	8	76474718	*HNF4G*	[1]
rs2977945	8	76475125	*HNF4G*	[1]
rs1805100	8	76476396	*HNF4G*	[1]
rs2272669	8	76476457	*HNF4G*	[1]
rs2941477	8	76476670	*HNF4G*	[1]
rs2941479	8	76476959	*HNF4G*	[1]
rs2941480	8	76477122	*HNF4G*	[1]
rs2941481	8	76477368	*HNF4G*	[1]
rs1515020	8	76477590	*HNF4G*	[1]
rs2941484	8	76478768	*HNF4G*	[1]
rs2943539	8	76479839	*3′ of HNF4G*	[1]
rs2941489	8	76482263	*3′ of HNF4G*	[1]
rs1913640	8	76483008	*3′ of HNF4G*	[1]
rs1913641	8	76483239	*3′ of HNF4G*	[1]
rs1464092	8	76483560	*3′ of HNF4G*	[1]
rs1399564	8	76483856	*3′ of HNF4G*	[1]
rs1399567	8	76484097	*3′ of HNF4G*	[1]
rs2941492	8	76487628	*3′ of HNF4G*	[1]
rs10957767	8	76492185	*3′ of HNF4G*	[1]
rs1817003	8	76499260	*3′ of HNF4G*	[1]
rs2122262	8	76503582	*3′ of HNF4G*	[1]
rs2922783	8	76506358	*3′ of HNF4G*	[1]
rs2943570	8	76506385	*3′ of HNF4G*	[1]

rs2943573	8	76507001	*3′ of HNF4G*	[1]
rs2012410	8	76509248	*3′ of HNF4G*	[1]
rs1375646	8	76517117	*3′ of HNF4G*	[1]
rs2922790	8	76518326	*3′ of HNF4G*	[1]
rs1449555	8	76518933	*3′ of HNF4G*	[1]
rs962711	8	76519323	*3′ of HNF4G*	[1]
rs962712	8	76519490	*3′ of HNF4G*	[1]
rs2941422	8	76519834	*3′ of HNF4G*	[1]
rs2941423	8	76519969	*3′ of HNF4G*	[1]
rs1913638	8	76523922	*3′ of HNF4G*	[1]
rs2943574	8	76524852	*3′ of HNF4G*	[1]
rs2941427	8	76524901	*3′ of HNF4G*	[1]
rs2922794	8	76525574	*3′ of HNF4G*	[1]
rs2941428	8	76525586	*3′ of HNF4G*	[1]
rs2941429	8	76529765	*3′ of HNF4G*	[1]
rs2922774	8	76531881	*3′ of HNF4G*	[1]
rs1839330	8	76532640	*3′ of HNF4G*	[1]
rs2922758	8	76542446	*3′ of HNF4G*	[1]
rs2941444	8	76559040	*3′ of HNF4G*	[1]
rs2167065	8	76560454	*3′ of HNF4G*	[1]
rs2060603	8	76564169	*3′ of HNF4G*	[1]
rs2060602	8	76564405	*3′ of HNF4G*	[1]
rs2035166	8	76749722	*5′ of AC016194.1*	[1]
rs1531460	8	76752696	*3′ of snoU13*	[1]
rs1531461	8	76752763	*3′ of snoU13*	[1]
rs2927234	8	76754904	*3′ of snoU13*	[1]
rs2977332	8	76754934	*3′ of snoU13*	[1]
rs2927235	8	76755997	*3′ of snoU13*	[1]
rs11785824	8	76756551	*3′ of snoU13*	[1]
rs2927237	8	76757519	*3′ of snoU13*	[1]
rs2927239	8	76758822	*3′ of snoU13*	[1]
rs1599589	8	76761762	*3′ of snoU13*	[1]
rs10087208	8	76762380	*3′ of snoU13*	[1]

rs1381666	8	76767476	*3′ of snoU13*	[1]
rs1381665	8	76767584	*3′ of snoU13*	[1]
rs1462441	8	76768738	*3′ of snoU13*	[1]
rs7827221	8	76773218	*3′ of snoU13*	[1]
rs877282	10	771532	*5′ of RP11-164C1.2*	[13]
rs11815391	10	52578738	*A1CF*	[1]
rs10821871	10	52584110	*RP11-449O16.2*	[1]
rs10821877	10	52603659	*A1CF*	[1]
rs10994720	10	52612101	*A1CF*	[1]
rs10994731	10	52614189	*A1CF*	[1]
rs4567398	10	52616922	*A1CF*	[1]
rs12768968	10	52621752	*A1CF*	[1]
rs17592117	10	52643038	*A1CF*	[1]
rs10994856	10	52645248	*A1CF*	[1]
rs10994860	10	52645424	*A1CF*	[1]
rs10821905	10	52646093	*5′ of A1CF*	[1]
rs3808919	10	52646344	*5′ of A1CF*	[1]
rs10761587	10	52649193	*5′ of A1CF*	[1]
rs4290173	10	52649771	*5′ of A1CF*	[1]
rs11599171	10	61394137	*3′ of SLC16A9*	[1]
rs2893807	10	61399990	*3′ of SLC16A9*	[1]
rs3763747	10	61412335	*SLC16A9*	[1]
rs12356193	10	61413353	*SLC16A9*	[8]
rs2242206	10	61414011	*SLC16A9*	[1]
rs1904071	10	61418691	*SLC16A9*	[1]
rs4948351	10	61425189	*SLC16A9*	[1]
rs11006679	10	61428646	*SLC16A9*	[1]
rs10826342	10	61433292	*SLC16A9*	[1]
rs1171606	10	61434519	*SLC16A9*	[1]
rs12772586	10	61440304	*SLC16A9*	[1]
rs1171660	10	61443808	*SLC16A9*	[1]
rs1171659	10	61444364	*SLC16A9*	[1]
rs1171658	10	61444748	*SLC16A9*	[1]

rs753763	10	61445412	*SLC16A9*	[1]
rs753762	10	61445594	*SLC16A9*	[1]
rs1171652	10	61449512	*SLC16A9*	[1]
rs7094971	10	61449564	*SLC16A9*	[1]
rs1171650	10	61450153	*SLC16A9*	[1]
rs1171648	10	61450932	*SLC16A9*	[1]
rs1171647	10	61452002	*SLC16A9*	[1]
rs1171645	10	61452592	*SLC16A9*	[1]
rs1171643	10	61452752	*SLC16A9*	[1]
rs1171620	10	61464507	*SLC16A9*	[1]
rs1171619	10	61465838	*SLC16A9*	[1]
rs1171618	10	61465941	*SLC16A9*	[1]
rs1171617	10	61467182	*SLC16A9*	[1]
rs1171616	10	61468589	*SLC16A9*	[1]
rs1171615	10	61469090	*SLC16A9*	[1]
rs1171614	10	61469538	*SLC16A9*	[1]
rs1171610	10	61477697	*SLC16A9*	[1]
rs1171609	10	61479385	*SLC16A9*	[1]
rs2671613	10	61486286	*SLC16A9*	[1]
rs1171582	10	61494037	*SLC16A9*	[1]
rs2090123	10	61525567	*5′ of RP11-59J5.1*	[1]
rs143709408	11	2549229	*KCNQ1*	[4]
rs147445322	11	2869033	*KCNQ1-AS1*	[4]
rs141208451	11	45538920	*RP11-958J22.1*	[26]
rs77459372	11	63762330	*OTUB1*	[11]
rs12418845	11	63873673	*FLRT1*	[1]
rs2186571	11	63915700	*MACROD1*	[30]
rs79382056	11	64154676	*3′ of AP003774.6*	[11]
rs7938871	11	64223527	*3′ of AP003774.4*	[1]
rs620692	11	64224628	*3′ of AP003774.4*	[1]
rs488998	11	64225239	*3′ of AP003774.4*	[1]
rs7936265	11	64225632	*3′ of AP003774.4*	[1]
rs4247633	11	64226279	*3′ of AP003774.4*	[1]

rs4247634	11	64226488	*3′ of AP003774.4*	[1]
rs1003697	11	64233783	*3′ of AP003774.4*	[1]
rs1000662	11	64233819	*3′ of AP003774.4*	[1]
rs685272	11	64237033	*3′ of AP003774.4*	[1]
rs547484	11	64237121	*3′ of AP003774.4*	[1]
rs490581	11	64238796	*3′ of AP003774.4*	[1]
rs192133	11	64240541	*3′ of AP003774.4*	[1]
rs171284	11	64240650	*3′ of AP003774.4*	[1]
rs240696	11	64240751	*3′ of AP003774.4*	[1]
rs475414	11	64241844	*3′ of AP003774.4*	[1]
rs522485	11	64244194	*5′ of AP005273.1*	[1]
rs606496	11	64249560	*5′ of AP005273.1*	[1]
rs525192	11	64250465	*5′ of AP005273.1*	[1]
rs634497	11	64251221	*5′ of AP005273.1*	[1]
rs2845635	11	64252970	*5′ of AP005273.1*	[1]
rs2845637	11	64253620	*5′ of AP005273.1*	[1]
rs2845638	11	64253704	*5′ of AP005273.1*	[1]
rs17299124	11	64256859	*5′ of AP005273.1*	[1]
rs589852	11	64257440	*5′ of AP005273.1*	[1]
rs604182	11	64258282	*5′ of AP005273.1*	[1]
rs615311	11	64258505	*5′ of AP005273.1*	[1]
rs617104	11	64258890	*5′ of AP005273.1*	[1]
rs1210879	11	64259806	*5′ of AP005273.1*	[1]
rs662228	11	64260107	*5′ of AP005273.1*	[1]
rs661292	11	64260478	*5′ of AP005273.1*	[1]
rs1210877	11	64265411	*5′ of AP005273.1*	[1]
rs7104498	11	64266244	*5′ of AP005273.1*	[1]
rs4930402	11	64269938	*AP005273.1*	[1]
rs7483260	11	64273034	*3′ of AP005273.1*	[1]
rs7939462	11	64299330	*5′ of SLC22A11*	[1]
rs12417589	11	64301713	*5′ of SLC22A11*	[1]
rs11231808	11	64302341	*5′ of SLC22A11*	[1]
rs10792438	11	64302526	*5′ of SLC22A11*	[1]

rs1939120	11	64304715	*5′ of SLC22A11*	[1]
rs7936185	11	64305452	*5′ of SLC22A11*	[1]
rs4930420	11	64307057	*5′ of SLC22A11*	[1]
rs7479811	11	64308784	*5′ of SLC22A11*	[1]
rs6591859	11	64311848	*5′ of SLC22A11*	[1]
rs4930423	11	64311897	*5′ of SLC22A11*	[1]
rs12362644	11	64312153	*5′ of SLC22A11*	[1]
rs11231816	11	64312403	*5′ of SLC22A11*	[1]
rs7124676	11	64313291	*5′ of SLC22A11*	[1]
rs4930426	11	64315914	*5′ of SLC22A11*	[1]
rs3759053	11	64323080	*5′ of SLC22A11*	[1]
rs7940321	11	64324120	*SLC22A11*	[1]
rs693591	11	64325069	*SLC22A11*	[1]
rs3782099	11	64327710	*SLC22A11*	[1]
rs7943154	11	64327844	*SLC22A11*	[1]
rs17300741	11	64331462	*SLC22A11*	[8]
rs17372915	11	64331568	*SLC22A11*	[1]
rs1783811	11	64333296	*SLC22A11*	[1]
rs2078267	11	64334114	*SLC22A11*	[1]
rs528211	11	64351721	*5′ of SLC22A12*	[1]
rs505802	11	64357072	*5′ of SLC22A12*	[8]
rs524023	11	64358265	*SLC22A12*	[1]
rs9734313	11	64358311	*SLC22A12*	[11]
rs3825018	11	64358809	*SLC22A12*	[1]
rs12800450	11	64359221	*SLC22A12*	[28]
rs3825016	11	64359286	*SLC22A12*	[1]
rs149722479	11	64360260	*SLC22A12*	[4]
rs11231825	11	64360274	*SLC22A12*	[11]
rs10897518	11	64360705	*SLC22A12*	[27]
rs121907892	11	64361219	*SLC22A12*	[31]
rs475688	11	64364291	*SLC22A12*	[1]
rs893006	11	64365796	*SLC22A12*	[1]

rs150255373	11	64366298	*SLC22A12*	[12]
rs147647315	11	64367854	*SLC22A12*	[12]
rs7932437	11	64373504	*3′ of NRXN2*	[1]
rs4930556	11	64377726	*NRXN2*	[1]
rs7121121	11	64381132	*NRXN2*	[1]
rs2277311	11	64387932	*NRXN2*	[1]
rs2360873	11	64391157	*NRXN2*	[1]
rs10897521	11	64391910	*NRXN2*	[1]
rs10792443	11	64395252	*NRXN2*	[1]
rs948689	11	64400719	*NRXN2*	[1]
rs1207226	11	64402027	*NRXN2*	[1]
rs11231845	11	64407419	*NRXN2*	[1]
rs2959654	11	64412086	*NRXN2*	[1]
rs1212146	11	64412877	*NRXN2*	[1]
rs7128467	11	64415111	*AP001092.4*	[1]
rs3825074	11	64415767	*AP001092.4*	[1]
rs555456	11	64418259	*AP001092.4*	[1]
rs2285339	11	64418305	*AP001092.4*	[1]
rs526338	11	64418900	*AP001092.4*	[12]
rs2269730	11	64423831	*AP001092.4*	[1]
rs7117423	11	64425120	*AP001092.4*	[1]
rs530775	11	64426933	*AP001092.4*	[1]
rs544838	11	64429059	*NRXN2*	[1]
rs492175	11	64433078	*NRXN2*	[1]
rs2666559	11	64439227	*NRXN2*	[1]
rs506338	11	64440920	*NRXN2*	[3]
rs530252	11	64443465	*NRXN2*	[1]
rs11602411	11	64449223	*NRXN2*	[1]
rs10128590	11	64449425	*NRXN2*	[1]
rs17146216	11	64451281	*NRXN2*	[1]
rs519090	11	64456240	*NRXN2*	[1]
rs504915	11	64464085	*NRXN2*	[11]

rs471618	11	64465403	*NRXN2*	[11]
rs490192	11	64471369	*NRXN2*	[1]
rs551890	11	64477999	*NRXN2*	[1]
rs478607	11	64478063	*NRXN2*	[1]
rs480617	11	64478320	*NRXN2*	[1]
rs2666558	11	64481579	*NRXN2*	[1]
rs487662	11	64481880	*NRXN2*	[1]
rs500531	11	64487019	*NRXN2*	[1]
rs7126110	11	64520255	*PYGM*	[1]
rs606458	11	64546391	*5′ of SF1*	[28]
rs493573	11	64557054	*MAP4K2*	[28]
rs10897526	11	64559898	*MAP4K2*	[11]
rs559566	11	64581658	*5′ of MEN1*	[1]
rs55975541	11	64597201	*CDC42BPG*	[11]
rs188780113	11	64678544	*ATG2A*	[31]
rs1466462	11	65419364	*3′ of SIPA1*	[1]
rs12289836	11	65436888	*5′ of RELA*	[1]
rs7947604	11	65442059	*5′ of RELA*	[1]
rs7934036	11	65451020	*5′ of Metazoa_SRP*	[1]
rs1074156	11	65458169	*5′ of Metazoa_SRP*	[1]
rs1074155	11	65458197	*5′ of Metazoa_SRP*	[1]
rs7115734	11	65458964	*5′ of Metazoa_SRP*	[1]
rs10896032	11	65459419	*5′ of Metazoa_SRP*	[1]
rs6591188	11	65467953	*3′ of Metazoa_SRP*	[1]
rs9666878	11	65475816	*5′ of KAT5*	[1]
rs4645933	11	65485120	*KAT5*	[1]
rs11227279	11	65495211	*5′ of RNASEH2C*	[1]
rs4014195	11	65506822	*5′ of RP11-770G2.2*	[1]
rs489574	11	65542739	*3′ of AP5B1*	[1]
rs11227299	11	65549570	*5′ of AP5B1*	[1]
rs642803	11	65560620	*OVOL1*	[1]
rs644740	11	65561468	*OVOL1*	[1]
rs44205	11	65564786	*3′ of OVOL1*	[1]

rs557675	11	65566719	*3′ of OVOL1*	[1]
rs7976059	12	52251272	*3′ of RP11-923I11.5*	[1]
rs7485577	12	57616061	*NXPH4*	[1]
rs7301155	12	57622821	*5′ of SHMT2*	[1]
rs11172134	12	57645789	*RP11-123K3.4*	[27]
rs11172147	12	57696677	*R3HDM2*	[1]
rs4760355	12	57725197	*R3HDM2*	[1]
rs11609805	12	57735045	*R3HDM2*	[1]
rs4760254	12	57766392	*R3HDM2*	[1]
rs4760278	12	57771153	*R3HDM2*	[1]
rs11613352	12	57792580	*R3HDM2*	[1]
rs1106766	12	57809456	*R3HDM2*	[7]
rs11614506	12	57815675	*R3HDM2*	[1]
rs7964492	12	57823585	*R3HDM2*	[1]
rs3741414	12	57844049	*INHBC*	[1]
rs3809114	12	57848639	*INHBE*	[1]
rs12229654	12	111414461	*3′ of RP1-46F2.2*	[4]
rs3184504	12	111884608	*SH2B3*	[1]
rs4766578	12	111904371	*ATXN2*	[1]
rs10774625	12	111910219	*ATXN2*	[1]
rs653178	12	112007756	*ATXN2*	[1]
rs11065987	12	112072424	*3′ of BRAP*	[1]
rs3782886	12	112110489	*BRAP*	[4]
rs11066015	12	112168009	*ACAD10*	[4]
rs671	12	112241766	*ALDH2*	[15]
rs2074356	12	112645401	*C12orf51*	[4]
rs11066280	12	112817783	*C12orf51*	[4]
rs7953704	12	122625992	*MLXIP*	[1]
rs35258188	15	53997338	*WDR72*	[4]
rs1394125	15	76158983	*UBE2Q2*	[1]
rs1976748	15	76160951	*UBE2Q2*	[1]
rs12437816	15	76194538	*3′ of UBE2Q2*	[1]

rs10444856	15	76232422	*NRG4*	[1]
rs1874953	15	76236837	*NRG4*	[1]
rs11634028	15	76276150	*NRG4*	[1]
rs10851884	15	76290042	*NRG4*	[1]
rs11072566	15	76293971	*NRG4*	[1]
rs4886755	15	76298132	*NRG4*	[1]
rs10851885	15	76304503	*NRG4*	[1]
rs12440225	15	76313121	*NRG4*	[1]
rs4966014	15	99248018	*IGF1R*	[1]
rs11858316	15	99249029	*IGF1R*	[1]
rs7174918	15	99251356	*IGF1R*	[1]
rs8032477	15	99254554	*IGF1R*	[1]
rs3803476	15	99256570	*IGF1R*	[1]
rs2018860	15	99258710	*IGF1R*	[1]
rs11634241	15	99259016	*IGF1R*	[1]
rs8038015	15	99263274	*IGF1R*	[1]
rs11857366	15	99263801	*IGF1R*	[1]
rs932071	15	99268030	*IGF1R*	[1]
rs4616271	15	99268259	*IGF1R*	[1]
rs4965430	15	99268850	*IGF1R*	[1]
rs6598541	15	99271135	*IGF1R*	[1]
rs875686	15	99272519	*IGF1R*	[1]
rs11633717	15	99273032	*IGF1R*	[1]
rs7166287	15	99273075	*IGF1R*	[1]
rs4966019	15	99274326	*IGF1R*	[1]
rs11633294	15	99275008	*IGF1R*	[1]
rs907808	15	99280254	*IGF1R*	[1]
rs1567811	15	99281833	*IGF1R*	[1]
rs3743263	15	99282558	*IGF1R*	[1]
rs3743264	15	99282587	*IGF1R*	[1]
rs1007212	15	99283918	*IGF1R*	[1]
rs2871974	15	99284074	*IGF1R*	[1]
rs8037467	15	99284652	*IGF1R*	[1]

rs4966020	15	99284680	*IGF1R*	[1]
rs4966021	15	99285056	*IGF1R*	[1]
rs4966022	15	99286284	*IGF1R*	[1]
rs12908437	15	99287375	*IGF1R*	[1]
rs2137683	15	99289638	*IGF1R*	[1]
rs11632952	15	99290975	*IGF1R*	[1]
rs7166999	15	99292805	*IGF1R*	[1]
rs6598542	15	99293056	*IGF1R*	[1]
rs6598543	15	99293552	*IGF1R*	[1]
rs12898337	15	99294355	*IGF1R*	[1]
rs12898502	15	99294452	*IGF1R*	[1]
rs4966024	15	99295570	*IGF1R*	[1]
rs8041224	15	99297665	*IGF1R*	[1]
rs143193096	15	99459327	*IGF1R*	[4]
rs150801101	15	99500588	*IGF1R*	[4]
rs188709583	16	20348705	*UMOD*	[4]
rs143583842	16	20352584	*UMOD*	[4]
rs139607138	16	20352615	*UMOD*	[4]
rs732021	16	69542478	*3′ of CYB5B*	[1]
rs7193778	16	69563890	*5′ of NFAT5*	[1]
rs200933617	16	69681065	*NFAT5*	[4]
rs889472	16	79645989	*5′ of MAF*	[32]
rs7188445	16	79734987	*RP11-345M22.1*	[1]
rs150320659	16	89703753	*DPEP1*	[4]
rs142226072	16	89703959	*DPEP1*	[4]
rs4617927	17	53356126	*HLF*	[1]
rs3829577	17	53360264	*HLF*	[1]
rs1477141	17	53361838	*HLF*	[1]
rs7224610	17	53364788	*HLF*	[1]
rs3794748	17	53365172	*HLF*	[1]
rs10852974	17	53372566	*HLF*	[1]
rs3829578	17	53381510	*HLF*	[1]
rs4793788	17	53381796	*HLF*	[1]

rs11650989	17	59449636	*BCAS3*	[1]
rs9905274	17	59450441	*BCAS3*	[1]
rs9895661	17	59456589	*BCAS3*	[1]
rs2079742	17	59465697	*BCAS3*	[1]
rs11079428	17	59466701	*BCAS3*	[1]
rs141310123	18	77171390	*NFATC1*	[4]
rs201178535	19	38591803	*SIPA1L3*	[4]
rs142085340	19	38597178	*SIPA1L3*	[4]
rs115276476	19	38631910	*SIPA1L3*	[4]
rs187171029	19	53599256	*ZNF160*	[26]
rs141158222	20	52570005	*BCAS1*	[4]
rs61754122	20	52645167	*BCAS1*	[4]
rs11554266	20	57478846	*GNAS*	[4]

SNP: single nucleotide polymorphism; Chr: chromosome.

[a]Positions of the SNP in hg19.

References

1. Köttgen, A.; Albrecht, E.; Teumer, A.; Vitart, V.; Krumsiek, J.; Hundertmark, C.; Pistis, G.; Ruggiero, D.; O'Seaghdha, C.M.; Haller, T., et al. Genome-wide association analyses identify 18 new loci associated with serum urate concentrations. *Nat. Genet.* 2013, *45*, 145-154.
2. Giri, A.K.; Banerjee, P.; Chakraborty, S.; Kauser, Y.; Undru, A.; Roy, S.; Parekatt, V.; Ghosh, S.; Tandon, N.; Bharadwaj, D. Genome wide association study of uric acid in Indian population and interaction of identified variants with Type 2 diabetes. *Sci. Rep.* 2016, *6*, 21440.
3. Yang, B.; Mo, Z.; Wu, C.; Yang, H.; Yang, X.; He, Y.; Gui, L.; Zhou, L.; Guo, H.; Zhang, X., et al. A genome-wide association study identifies common variants influencing serum uric acid concentrations in a Chinese population. BMC. Med. Genomics. 2014, 7, 10.
4. Yasukochi, Y.; Sakuma, J.; Takeuchi, I.; Kato, K.; Oguri, M.; Fujimaki, T.; Horibe, H.; Yamada, Y. Identification of CDC42BPG as a novel susceptibility locus for hyperuricemia in a Japanese population. Mol. Genet. Genomics. 2018, 293, 371-379.
5. Son, C.N.; Bang, S.Y.; Cho, S.K.; Sung, Y.K.; Kim, T.H.; Bae, S.C.; Jun, J.B. The frequency of single nucleotide polymorphisms and their association with uric acid concentration based on data from genome-wide association studies in the Korean population. Rheumatol. Int. 2014, 34, 777-783.
6. Wei, W.H.; Guo, Y.; Kindt, A.S.; Merriman, T.R.; Semple, C.A.; Wang, K.; Haley, C.S. Abundant local interactions in the 4p16.1 region suggest functional mechanisms underlying SLC2A9 associations with human serum uric acid. Hum. Mol. Genet. 2014, 23, 5061-5068.
7. Yang, Q.; Köttgen, A.; Dehghan, A.; Smith, A.V.; Glazer, N.L.; Chen, M.H.; Chasman, D.I.; Aspelund, T.; Eiriksdottir, G.; Harris, T.B., et al. Multiple genetic loci influence serum urate levels and their relationship with gout and cardiovascular disease risk factors. Circ. Cardiovasc. Genet. 2010, 3, 523-530.
8. Kolz, M.; Johnson, T.; Sanna, S.; Teumer, A.; Vitart, V.; Perola, M.; Mangino, M.; Albrecht, E.; Wallace, C.; Farrall, M., et al. Meta-analysis of 28,141 individuals identifies common variants within five new loci that influence uric acid concentrations. PLoS. Genet. 2009, 5, e1000504.
9. Lee, Y.H.; Song, G.G. Pathway analysis of genome-wide association studies on uric acid concentrations. Hum. Immunol. 2012, 73, 805-810.

10. Voruganti, V.S.; Franceschini, N.; Haack, K.; Laston, S.; MacCluer, J.W.; Umans, J.G.; Comuzzie, A.G.; North, K.E.; Cole, S.A. Replication of the effect of SLC2A9 genetic variation on serum uric acid levels in American Indians. Eur. J. Hum. Genet. 2014, 22, 938-943.
11. Lee, J.; Lee, Y.; Park, B.; Won, S.; Han, J.S.; Heo, N.J. Genome-wide association analysis identifies multiple loci associated with kidney disease-related traits in Korean populations. PLoS. One. 2018, 13, e0194044.
12. Tin, A.; Li, Y.; Brody, J.A.; Nutile, T.; Chu, A.Y.; Huffman, J.E.; Yang, Q.; Chen, M.H.; Robinson-Cohen, C.; Macé, A., et al. Large-scale whole-exome sequencing association studies identify rare functional variants influencing serum urate levels. Nat. Commun. 2018, 9, 4228.
13. Li, W.D.; Jiao, H.; Wang, K.; Zhang, C.K.; Glessner, J.T.; Grant, S.F.; Zhao, H.; Hakonarson, H.; Arlen Price, R. A genome wide association study of plasma uric acid levels in obese cases and never-overweight controls. Obesity (Silver Spring). 2013, 21, E490-494.
14. Sarzynski, M.A.; Jacobson, P.; Rankinen, T.; Carlsson, B.; Sjöström, L.; Bouchard, C.; Carlsson, L.M. Changes in uric acid levels following bariatric surgery are not associated with SLC2A9 variants in the Swedish Obese Subjects Study. PLoS. One. 2012, 7, e51658.
15. Zhang, D.; Yang, M.; Zhou, D.; Li, Z.; Cai, L.; Bao, Y.; Li, H.; Shan, Z.; Liu, J.; Lv, D., et al. The polymorphism rs671 at ALDH2 associated with serum uric acid levels in Chinese Han males: A genome-wide association study. Gene. 2018, 651, 62-69.
16. Charles, B.A.; Shriner, D.; Doumatey, A.; Chen, G.; Zhou, J.; Huang, H.; Herbert, A.; Gerry, N.P.; Christman, M.F.; Adeyemo, A., et al. A genome-wide association study of serum uric acid in African Americans. BMC. Med. Genomics. 2011, 4, 17.
17. Dehghan, A.; Köttgen, A.; Yang, Q.; Hwang, S.J.; Kao, W.L.; Rivadeneira, F.; Boerwinkle, E.; Levy, D.; Hofman, A.; Astor, B.C., et al. Association of three genetic loci with uric acid concentration and risk of gout: a genome-wide association study. Lancet. 2008, 372, 1953-1961.
18. Rivera-Paredez, B.; Macías-Kauffer, L.; Fernandez-Lopez, J.C.; Villalobos-Comparán, M.; Martinez-Aguilar, M.M.; de la Cruz-Montoya, A.; Ramírez-Salazar, E.G.; Villamil-Ramírez, H.; Quiterio, M.; Ramírez-Palacios, P., et al. Influence of Genetic and Non-Genetic Risk Factors for Serum Uric Acid Levels and Hyperuricemia in Mexicans. Nutrients. 2019, 11, E1336.
19. Sull, J.W.; Park, E.J.; Lee, M.; Jee, S.H. Effects of SLC2A9 variants on uric acid levels in a Korean population. Rheumatol. Int. 2013, 33, 19-23.
20. Macias-Kauffer, L.R.; Villamil-Ramírez, H.; León-Mimila, P.; Jacobo-Albavera, L.; Posadas-Romero, C.; Posadas-Sánchez, R.; López-Contreras, B.E.; Morán-Ramos, S.; Romero-Hidalgo, S.; Acuña-Alonzo, V., et al. Genetic contributors to serum uric acid levels in Mexicans and their effect on premature coronary artery disease. Int. J. Cardiol. 2019, 279, 168-173.
21. Döring, A.; Gieger, C.; Mehta, D.; Gohlke, H.; Prokisch, H.; Coassin, S.; Fischer, G.; Henke, K.; Klopp, N.; Kronenberg, F., et al. SLC2A9 influences uric acid concentrations with pronounced sex-specific effects. Nat. Genet. 2008, 40, 430-436.
22. Li, S.; Sanna, S.; Maschio, A.; Busonero, F.; Usala, G.; Mulas, A.; Lai, S.; Dei, M.; Orrù, M.; Albai, G., et al. The GLUT9 gene is associated with serum uric acid levels in Sardinia and Chianti cohorts. PLoS. Genet. 2007, 3, e194.
23. Chiang, K.M.; Tsay, Y.C.; Vincent Ng, T.C.; Yang, H.C.; Huang, Y.T.; Chen, C.H.; Pan, W.H. Is Hyperuricemia, an Early-Onset Metabolic Disorder, Causally Associated with Cardiovascular Disease Events in Han Chinese? J. Clin. Med. 2019, 8, E1202.
24. McArdle, P.F.; Parsa, A.; Chang, Y.P.; Weir, M.R.; O'Connell, J.R.; Mitchell, B.D.; Shuldiner, A.R. Association of a common nonsynonymous variant in GLUT9 with serum uric acid levels in old order amish. Arthritis. Rheum. 2008, 58, 2874-2881.
25. Bhatnagar, V.; Richard, E.L.; Wu, W.; Nievergelt, C.M.; Lipkowitz, M.S.; Jeff, J.; Maihofer, A.X.; Nigam, S.K. Analysis of ABCG2 and other urate transporters in uric acid homeostasis in chronic kidney disease: potential role of remote sensing and signaling. Clin. Kidney. J. 2016, 9, 444-453.
26. Nagy, R.; Boutin, T.S.; Marten, J.; Huffman, J.E.; Kerr, S.M.; Campbell, A.; Evenden, L.; Gibson, J.; Amador, C.; Howard, D.M., et al. Exploration of haplotype research consortium imputation for genome-wide association studies in 20,032 Generation Scotland participants. Genome. Med. 2017, 9, 23.
27. Huffman, J.E.; Albrecht, E.; Teumer, A.; Mangino, M.; Kapur, K.; Johnson, T.; Kutalik, Z.; Pirastu, N.; Pistis, G.; Lopez, L.M., et al. Modulation of genetic associations with serum urate levels by body-mass-index in humans. PLoS. One. 2015, 10, e0119752.

28. Tin, A.; Woodward, O.M.; Kao, W.H.; Liu, C.T.; Lu, X.; Nalls, M.A.; Shriner, D.; Semmo, M.; Akylbekova, E.L.; Wyatt, S.B., et al. Genome-wide association study for serum urate concentrations and gout among African Americans identifies genomic risk loci and a novel URAT1 loss-of-function allele. Hum. Mol. Genet. 2011, 20, 4056-4068.
29. Larsson, S.C.; Carlström, M. Coffee consumption and gout: a Mendelian randomisation study. Ann. Rheum. Dis. 2018, 77, 1544-1546.
30. Kenny, E.E.; Kim, M.; Gusev, A.; Lowe, J.K.; Salit, J.; Smith, J.G.; Kovvali, S.; Kang, H.M.; Newton-Cheh, C.; Daly, M.J., et al. Increased power of mixed models facilitates association mapping of 10 loci for metabolic traits in an isolated population. Hum. Mol. Genet. 2011, 20, 827-839.
31. Yamada, Y.; Sakuma, J.; Takeuchi, I.; Yasukochi, Y.; Kato, K.; Oguri, M.; Fujimaki, T.; Horibe, H.; Muramatsu, M.; Sawabe, M., et al. Identification of C21orf59 and ATG2A as novel determinants of renal function-related traits in Japanese by exome-wide association studies. Oncotarget. 2017, 8, 45259-45273.
32. Okada, Y.; Sim, X.; Go, M.J.; Wu, J.Y.; Gu, D.; Takeuchi, F.; Takahashi, A.; Maeda, S.; Tsunoda, T.; Chen, P., et al. Meta-analysis identifies multiple loci associated with kidney function-related traits in east Asian populations. Nat. Genet. 2012, 44, 904-909.

Table S4. Causal inference tests of rs186201319 and cg03081134 (*RECK*), rs539604468 and cg01680773 (*NPC2*).

Variant	CpG site	Gene	P_{SM}	P_{SG}	P_{CIT}
rs186201319	cg03081134	*RECK*	2.92×10^{-145}	1.74×10^{-3}	0.148
rs539604468	cg01680773	*NPC2*	8.95×10^{-152}	4.45×10^{-2}	0.093

P_{SM}: *P* value of variant effects on CpG methylation; P_{SG}: *P* value of variant effects on gout; P_{CIT}: *P* value of causal inference test.

Table S5. Transcription factors identified to bind cg26201826 (*PGGT1B*) by MoLoTool.

TF	Motif	*P*
NFIC	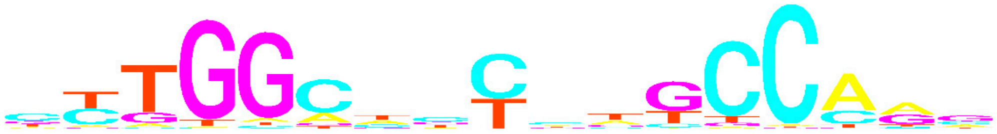	4.72×10^{-4}
NR2C2	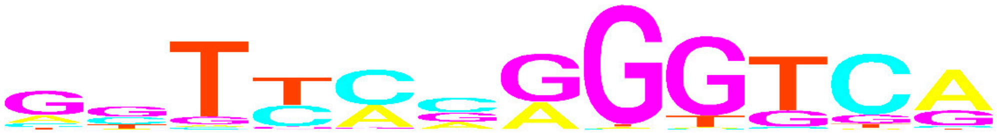	2.14×10^{-5}
TFAP2D	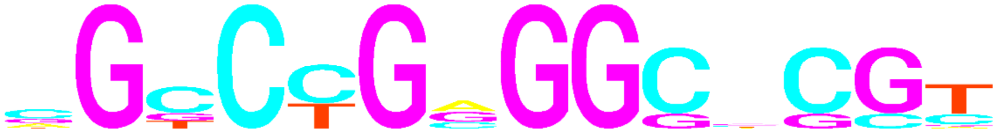	4.96×10^{-4}

TF: transcription factor.

Table S6. Transcription factors binding cg26201826 (*PGGT1B*) in ReMap.

TF	Cell
BRD4	MOLM-14, MV4-11
CDK8	MOLM-14
CDK9	MV4-11
CTCF	THP-1
INTS13 (*C12orf11*)	monocyte
KMT2A	MV4-11, THP-1
MEF2C	MOLM-13
MLLT1	MV4-11
MYB	THP-1
RAD21	monocyte-derived macrophage
RBP2	U-937
RelA(*p65*)	monocyte

RUNX1	MV4-11, THP-1
SPI1	macrophage, monocyte, monocyte-derived macrophage, THP-1, U-937
STAT1	CD14+ cells
TRIM24	MOLM-13
VDR	THP-1

TF: transcription factor.

Table S7. Transcription factors identified to bind cg20419410 (*INSIG1*) by MoLoTool.

TF	Motif	*P*
SP1	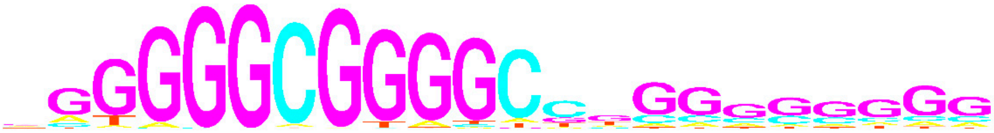	4.47×10^{-5}
SPZ1	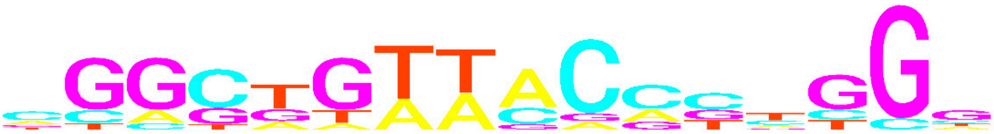	3.14×10^{-4}
THAP1	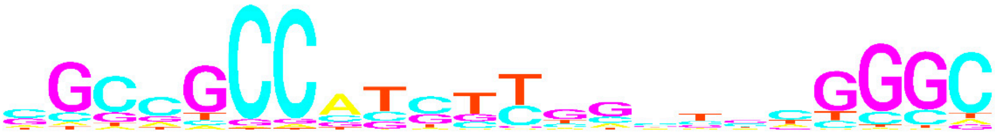	2.76×10^{-4}
THAP11	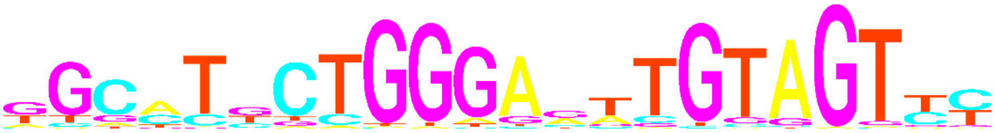	2.79×10^{-4}
ZBTB14	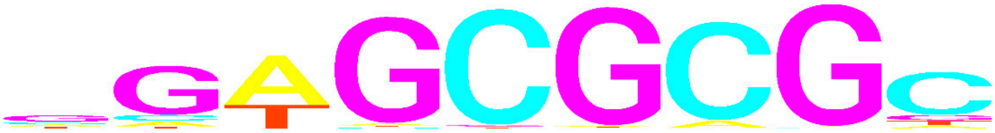	1.47×10^{-4}
ZIC2	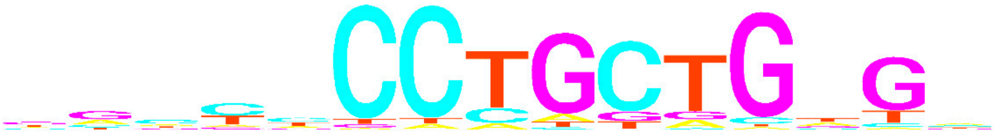	1.85×10^{-4}
ZIC3	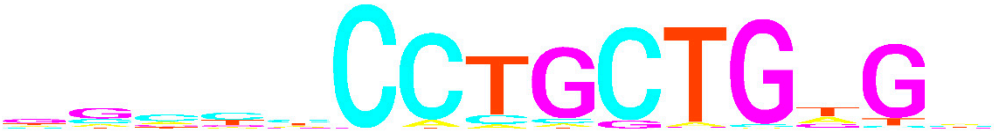	2.09×10^{-4}
ZNF76	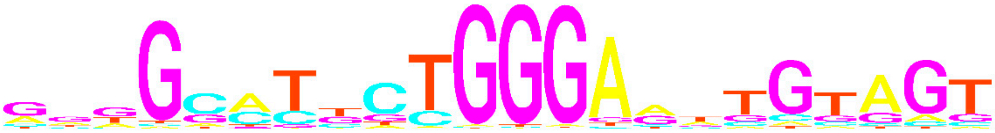	2.03×10^{-4}
ZNF143	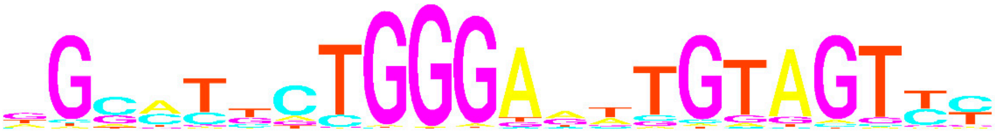	4.68×10^{-4}
ZNF350	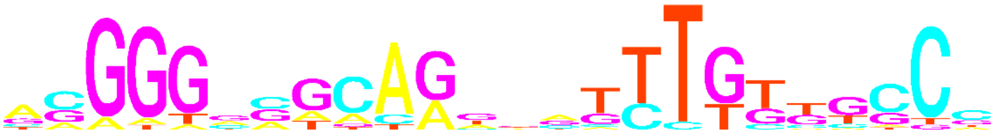	3.44×10^{-4}

TF: transcription factor.

Table S8. Transcription factors binding cg20419410 (*INSIG1*) in ReMap.

TF	Cell
BRD4	MOLM-14, MV4-11
CDK8	MOLM-14
CEBPB	MV4-11
MED1	MOLM-14
MLLT1	MV4-11
RAD21	monocyte-derived macrophage
SP140	macrophage
STAT1	CD14+ cells
TRIM24	MOLM-13
VDR	THP-1

TF: transcription factor.

Table S9. Transcription factors identified to bind cg17618153 (*ANGPTL2*) by MoLoTool.

TF	Motif	*P*
OSR2	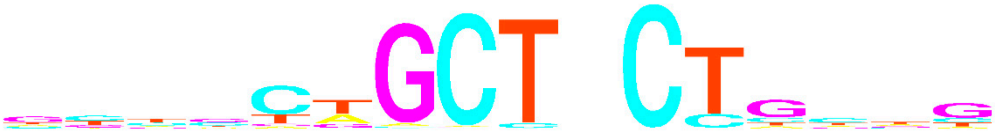	4.51×10^{-5}
ZNF214	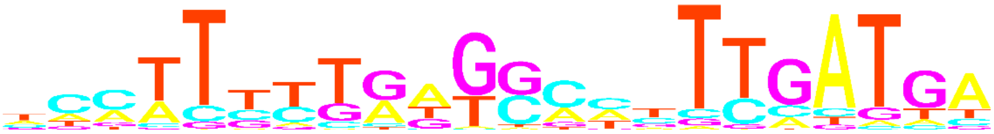	4.99×10^{-5}

TF: transcription factor.

Table S10. Transcription factors identified to bind cg15686135 (*JNK1*) by MoLoTool.

TF	Motif	*P*
ELF1	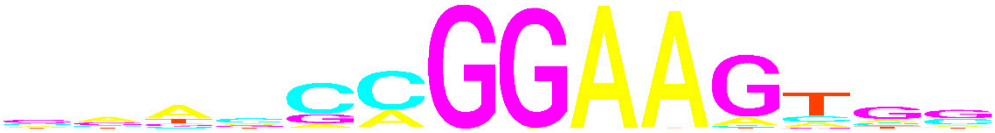	2.66×10^{-4}
ELK1	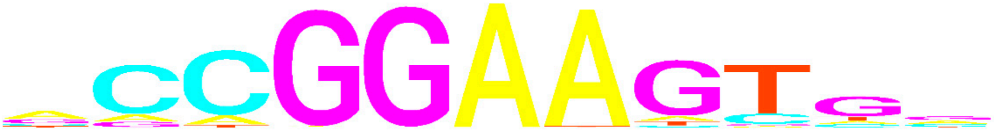	8.20×10^{-5}
ELK3	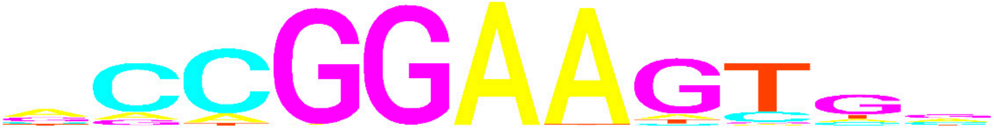	4.81×10^{-4}
ELK4	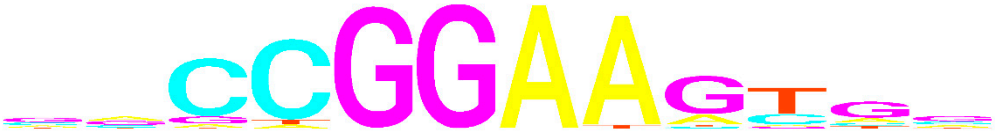	9.33×10^{-5}
ETV1	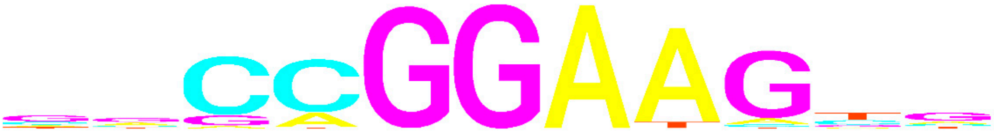	3.11×10^{-4}

GABPA	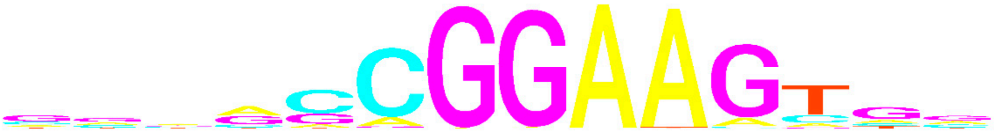	1.06×10^{-4}
OVOL2	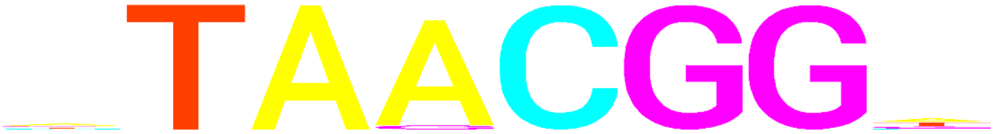	2.59×10^{-4}
SPI1	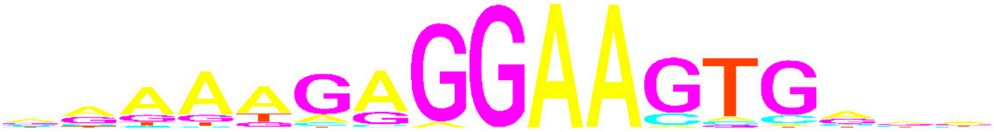	3.83×10^{-4}
SPIB	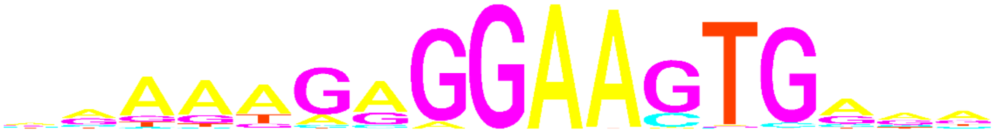	1.20×10^{-4}
ZBTB7B	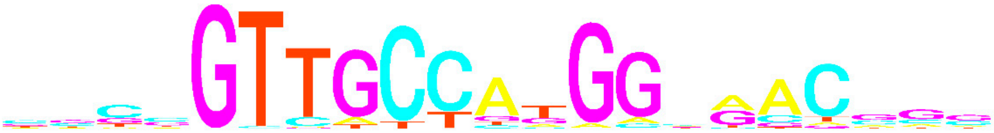	4.28×10^{-4}
ZNF341	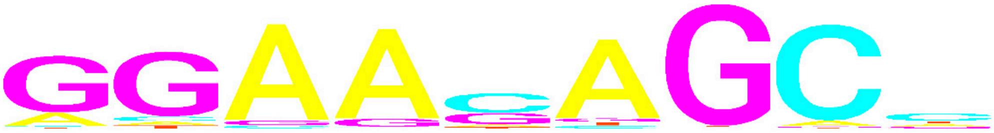	2.97×10^{-4}

TF: transcription factor.

Table S11. Transcription factors identified to bind cg14167017 (*UBAP1*) by MoLoTool.

TF	Motif	*P*
CENPB	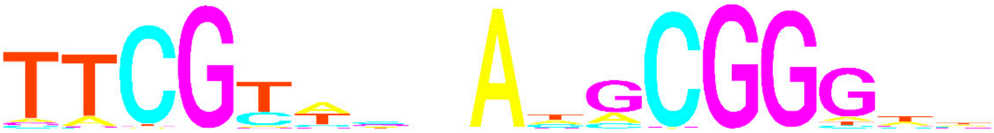	1.60×10^{-4}
ZBTB33	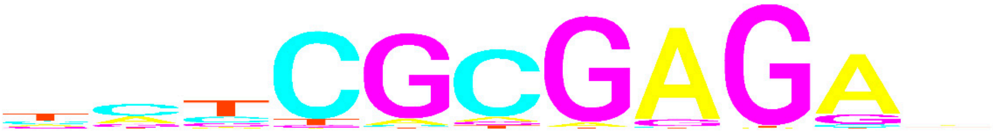	2.58×10^{-5}

TF: transcription factor.

Table S12. Transcription factors binding cg14167017 (*UBAP1*) in ReMap.

TF	Cell
ATF2	macrophage
BRD4	MOLM-14, MV4-11
CDK8	MOLM-14
CEBPA	MV4-11
CEBPB	MV4-11, THP-1
CTCF	THP-1
FOS	MV4-11
INTS13 (*C12orf11*)	monocyte
MEF2C	MOLM-13
MLLT1	MV4-11
RAD21	monocyte-derived macrophage, THP-1

RelA(*p65*)	monocyte
RUNX1	MV4-11, THP-1
RXR	macrophage
SMC1A	monocyte
SPI1	macrophage, monocyte, monocyte-derived macrophage, THP-1, U-937
STAT1	CD14+ cells
TRIM24	MOLM-13
VDR	THP-1

TF: transcription factor.

Table S13. Transcription factors identified to bind cg11988568 (*RAPTOR*) by MoLoTool.

TF	Motif	*P*
E2F4	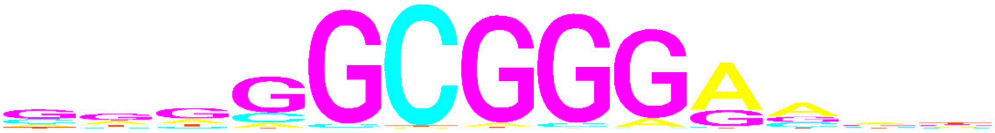	1.36×10^{-4}
GCM2	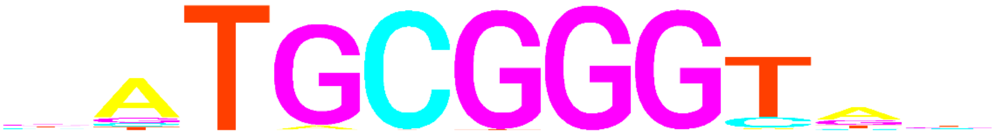	4.03×10^{-4}
KLF3	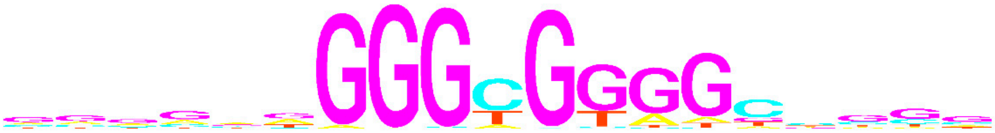	2.13×10^{-4}
KLF6	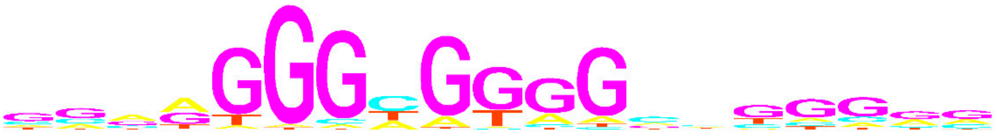	4.06×10^{-4}
KLF9	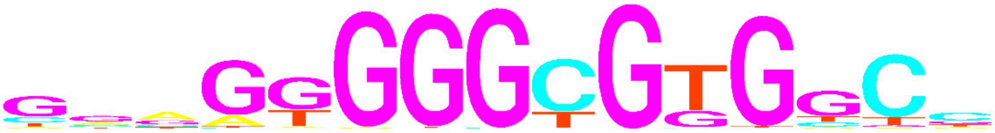	1.29×10^{-4}
MAZ	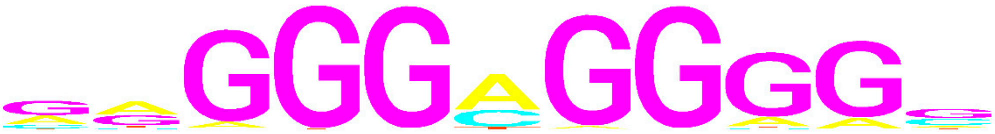	3.33×10^{-4}
NR0B1	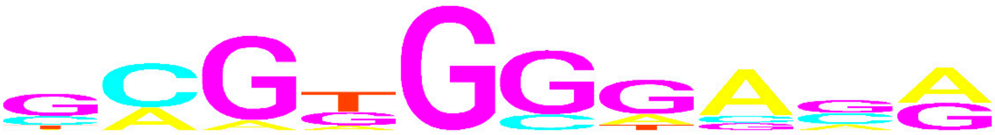	3.26×10^{-4}
NR1H4(*FXR*)	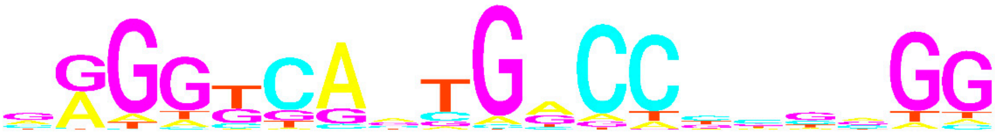	2.29×10^{-4}
PAX1	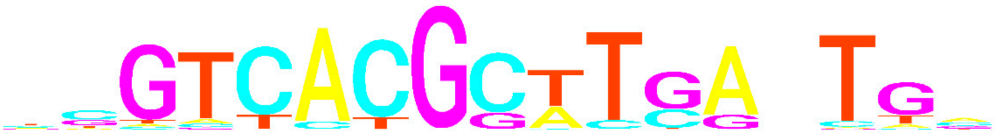	2.26×10^{-4}

REST	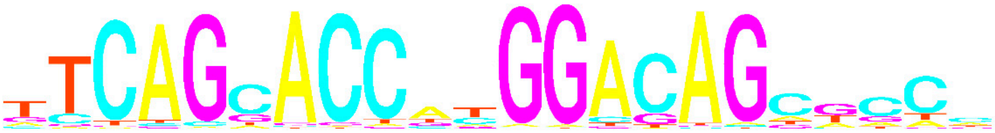	4.97×10^{-4}
SP1	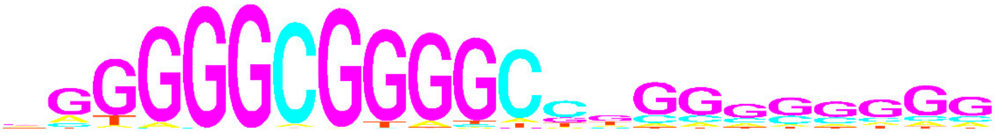	8.79×10^{-5}
SP2	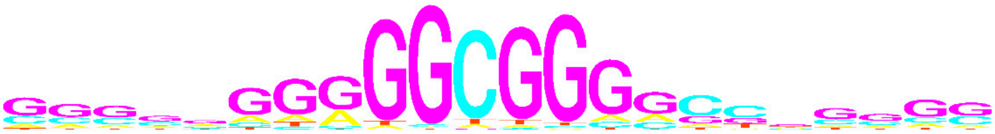	4.62×10^{-6}
SP3	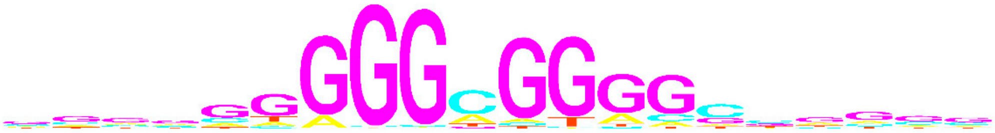	2.84×10^{-4}
SP4	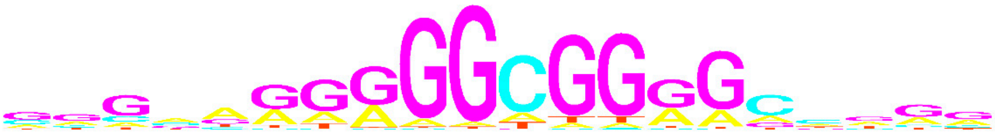	1.99×10^{-4}
TFAP2D	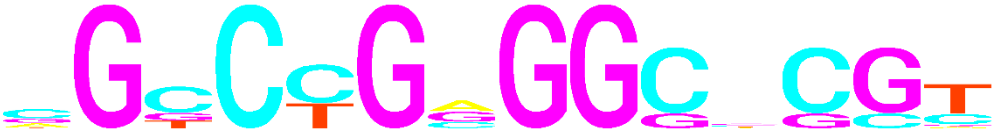	3.01×10^{-4}
ZBTB14	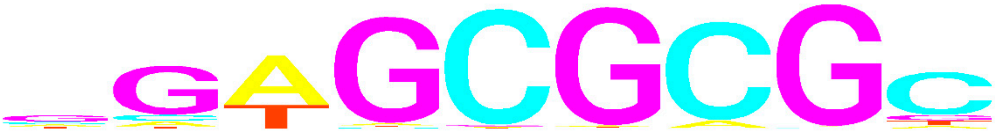	2.50×10^{-4}
ZNF768	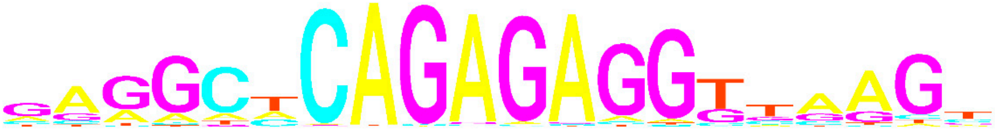	2.62×10^{-4}
ZNF816 (*ZNF816A*)	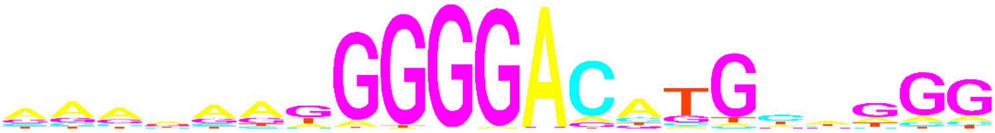	7.64×10^{-5}

TF: transcription factor.

Table S14. Transcription factors binding cg11988568 (*RAPTOR*) in ReMap.

TF	Cell
BRD4	MOLM-14, MV4-11
CDK8	MOLM-14
CTCF	U-937
KMT2A	MV4-11
MLLT1	MV4-11
RAD21	monocyte-derived macrophage
RBP2	U-937
SP140	macrophage
SPI1	macrophage, monocyte, THP-1
TRIM24	MOLM-13
VDR	THP-1
ZFX	NOMO1

TF: transcription factor.

Table S15. Transcription factors identified to bind cg16745952 (*CNTN5*) by MoLoTool.

TF	Motif	*P*
BHLHE40	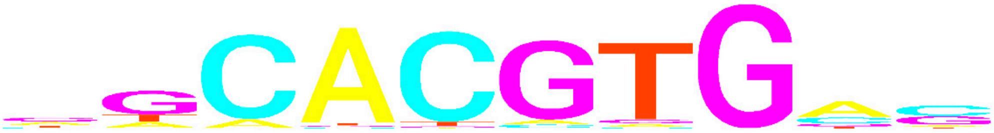	2.79×10^{-4}
CLOCK	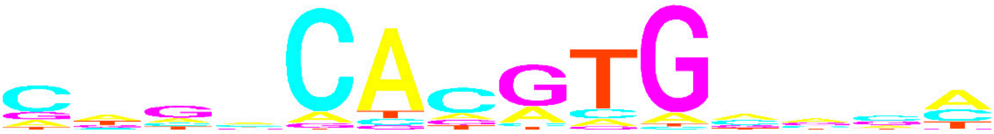	2.03×10^{-4}
HES1	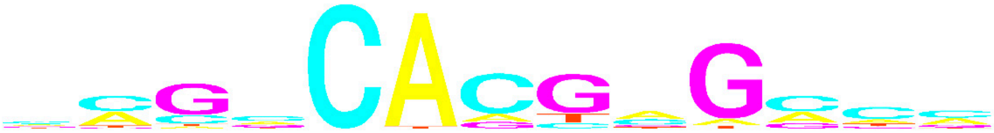	2.17×10^{-4}
MAX	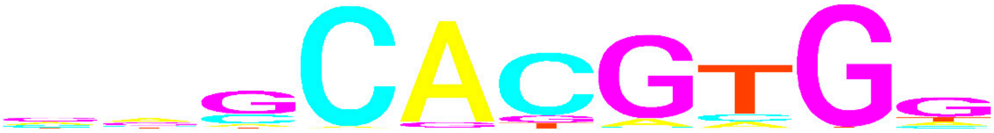	4.32×10^{-4}
MYCN	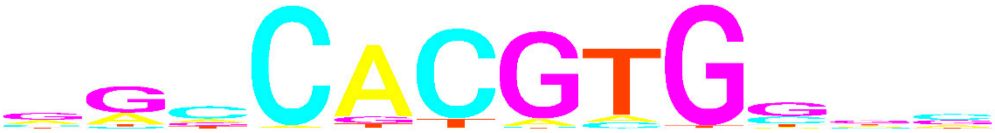	3.24×10^{-4}
ZBTB33	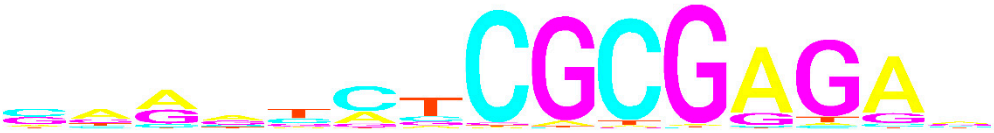	8.40×10^{-6}
ZNF586	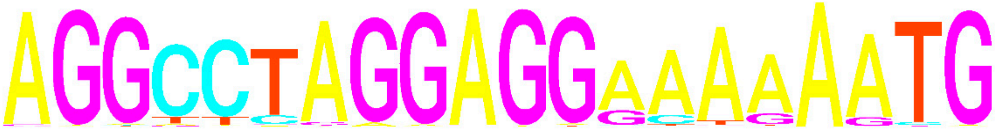	2.72×10^{-5}
ZNF770	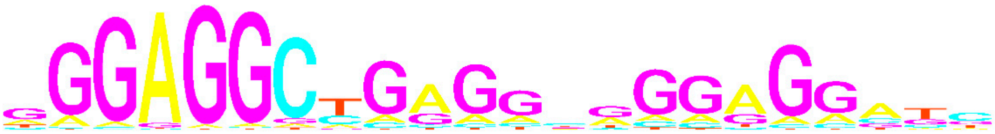	2.29×10^{-4}

TF: transcription factor.

Table S16. CpG sites located in transcription factors which are associated with gout at nominal significance ($p < 0.05$).

TF	CpG site	Δβ[a]	*P*	FDR	Chr	Position[b]	Genomic features
CDK8							
	cg21540848	0.18%	2.93×10^{-2}	8.09×10^{-1}	13	26827827	TSS1500
	cg00022633	0.21%	7.00×10^{-3}	7.24×10^{-1}	13	26827877	TSS1500
	cg23571288	0.24%	4.27×10^{-3}	6.61×10^{-1}	13	26828759	5'UTR
CEBPB							
	cg17779707	0.29%	2.80×10^{-2}	8.08×10^{-1}	20	48807327	TSS200
CLOCK							
	cg11569219	0.16%	4.52×10^{-2}	8.26×10^{-1}	4	56413215	TSS1500
	cg07236904	0.08%	1.60×10^{-2}	7.89×10^{-1}	4	56413229	TSS1500
CTCF							

cg07967402	0.08%	4.33×10^{-2}	8.25×10^{-1}	16	67595843	TSS1500
cg27250362	0.55%	7.78×10^{-3}	7.32×10^{-1}	16	67597316	5'UTR
E2F4						
cg10089978	0.11%	3.10×10^{-2}	8.11×10^{-1}	16	67225962	TSS200
ELF1						
cg19515798	-1.31%	3.52×10^{-2}	8.18×10^{-1}	13	41557187	TSS1500
ELK3						
cg26194015	0.11%	2.05×10^{-2}	7.91×10^{-1}	12	96588125	TSS200
cg25127026	0.76%	1.47×10^{-2}	7.80×10^{-1}	12	96606023	5'UTR
FOS						
cg03509965	0.07%	1.39×10^{-2}	7.74×10^{-1}	14	75743987	TSS1500
cg13819869	0.10%	2.14×10^{-2}	7.91×10^{-1}	14	75745020	TSS1500
cg13519079	0.06%	4.26×10^{-2}	8.22×10^{-1}	14	75745447	TSS200
HES1						
cg26348180	1.04%	1.70×10^{-2}	7.91×10^{-1}	3	193852754	TSS1500
cg17460737	0.26%	2.29×10^{-2}	7.98×10^{-1}	3	193852991	TSS1500
cg25466368	0.27%	2.83×10^{-2}	8.08×10^{-1}	3	193853978	5'UTR
			INTS13(C12orf11)			
cg02301006	0.22%	2.17×10^{-2}	7.93×10^{-1}	12	27091323	TSS200
KLF3						
cg01986205	0.21%	3.81×10^{-2}	8.21×10^{-1}	4	38665594	TSS200
MAZ						
cg09291817	0.18%	4.42×10^{-2}	8.25×10^{-1}	16	29816730	TSS1500
MEF2C						
cg15796366	0.70%	3.73×10^{-2}	8.19×10^{-1}	5	88120826	TSS1500
cg27557048	0.27%	3.40×10^{-3}	6.50×10^{-1}	6	135501969	TSS1500
NR1H4						
cg04250561	-0.62%	4.00×10^{-2}	8.22×10^{-1}	12	100896223	TSS1500
cg05108417	-0.38%	2.34×10^{-2}	7.98×10^{-1}	12	100897076	TSS200
NR2C2						
cg02081905	0.14%	1.02×10^{-2}	7.53×10^{-1}	3	14988669	TSS1500
OSR2						
cg14548272	0.76%	2.88×10^{-2}	8.08×10^{-1}	8	99961122	5'UTR
PAX1						
cg26463200	0.70%	6.67×10^{-3}	7.19×10^{-1}	20	21685401	TSS1500
REST						
cg11939399	1.30%	2.53×10^{-2}	8.02×10^{-1}	4	57773300	TSS1500
cg09070522	0.64%	4.17×10^{-2}	8.22×10^{-1}	4	57774717	5'UTR
cg05246887	0.20%	2.07×10^{-2}	7.91×10^{-1}	4	57775294	5'UTR
RXRA[c]						
cg13978325	0.48%	2.49×10^{-2}	8.00×10^{-1}	9	137218288	TSS200
RXRG[c]						
cg22758486	-1.29%	4.10×10^{-2}	8.22×10^{-1}	1	165415170	TSS1500
SP1						
cg13510262	0.29%	4.99×10^{-3}	6.81×10^{-1}	12	53774037	TSS1500
SP2						
cg24687229	0.35%	3.97×10^{-3}	6.58×10^{-1}	17	45973466	TSS200
SP3						
cg23039033	0.20%	4.94×10^{-2}	8.31×10^{-1}	2	174830966	TSS1500
SP4						
cg20441701	0.62%	4.70×10^{-2}	8.29×10^{-1}	7	21466380	TSS1500
STAT1						
cg25579739	0.06%	4.79×10^{-2}	8.31×10^{-1}	2	191878969	5'UTR
TFAP2D						
cg11790335	0.16%	2.87×10^{-2}	8.08×10^{-1}	6	50679857	TSS1500
THAP1						
cg11242773	-1.00%	4.10×10^{-2}	8.22×10^{-1}	8	42698994	TSS1500
TRIM24						
cg13935558	1.16%	4.96×10^{-2}	8.31×10^{-1}	7	138144067	TSS1500
ZBTB14						
cg10091115	-0.27%	3.24×10^{-2}	8.11×10^{-1}	18	5297658	TSS1500
ZBTB7B						
cg16484021	0.96%	1.26×10^{-2}	7.66×10^{-1}	1	154978318	5'UTR
cg22505006	0.72%	3.76×10^{-2}	8.19×10^{-1}	1	154981829	5'UTR
ZNF143						
cg10400440	0.16%	7.32×10^{-3}	7.26×10^{-1}	11	9482413	TSS200
ZNF341						
cg10875346	0.10%	2.38×10^{-3}	6.05×10^{-1}	20	32319743	TSS200

ZNF586						
cg03584535	0.62%	9.82×10^{-3}	7.51×10^{-1}	19	58280832	TSS200
ZNF768						
cg07936430	0.81%	1.16×10^{-2}	7.61×10^{-1}	16	30538244	TSS1500

TF:transcription factor; FDR:false discovery rate; Chr: chromosome. [a]Methylation levels of gout minus methylation levels of non-gout estimated with linear regression, after adjusting for sex, age, smoking history (total pack-years), smoking status, alcohol consumption,and cell subsets. [b]Positions of the CpG sites in hg19. [c]Because *RXRA*, *RXRB*, and *RXRG* are called collectively as *RXR* in ReMap, we use CpG sites located in *RXRA*, *RXRB*, and *RXRG* to represent methylation of *RXR*.

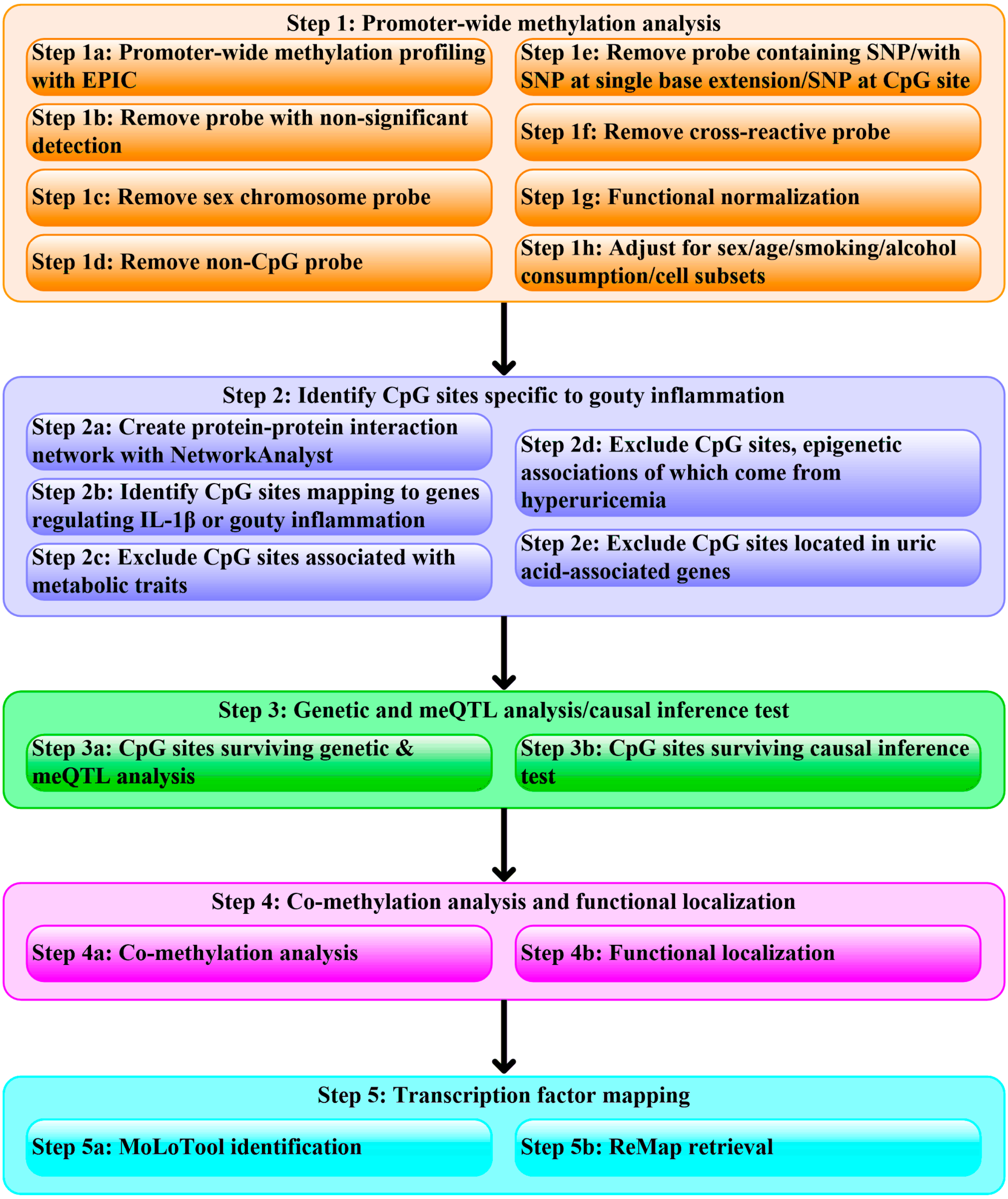

Figure S1. Flowchart of the analysis pipeline. Arrows indicate the direction of the workflow of the study design. Five major steps are represented by different colors. The boxes indicate the analyses taken within each step. The study cohort first undergoes promoter-wide methylation profiling with EPIC array (Step 1a). After removing probes with non-significant detection (Step 1b), sex chromosome

probes (Step 1c), non-CpG probes (Step 1d), probes containing single nucleotide polymorphism (SNP) or with SNP at single base extension or SNP at CpG site (Step 1e), cross-reactive probes (Step 1f), remaining probes are subjected to functional normalization (Step 1g) and linear regression, adjusting for sex, age, smoking history (total pack-years), smoking status, alcohol consumption, and cell subsets (Step 1h). Probes that are significantly associated with gout enter following analyses (Step 2-5). In Step 2a, we create protein-protein interaction network with NetworkAnalyst to gain insight about the biologic network of gout (Step 2a). We then identify CpG sites that are mapped to genes regulating interleukin-1β (IL-1β) or gouty inflammation (Step 2b). We further exclude CpG sites that are associated with metabolic traits (Step 2c), epigenetic associations of which come from hyperuricemia (Step 2d), or which are located in uric acid-associated genes (Step 2e). For the remaining CpG sites, genetic and methylation quantitative trait loci (meQTL) analyses (Step 3a) with additional causal inference tests (Step 3b) are conducted to identify CpG sites whose epigenetic relationships with gout are not confounded by genetic factors. Co-methylation analysis (Step 4a) and functional localization (Step 4b) are performed to reveal regulatory potential of CpG sites. We finally utilize MoLoTool (Step 5a) and ReMap (Step 5b) to identify potential transcription factors mediating the relationship between CpG sites and gout (Step 5).

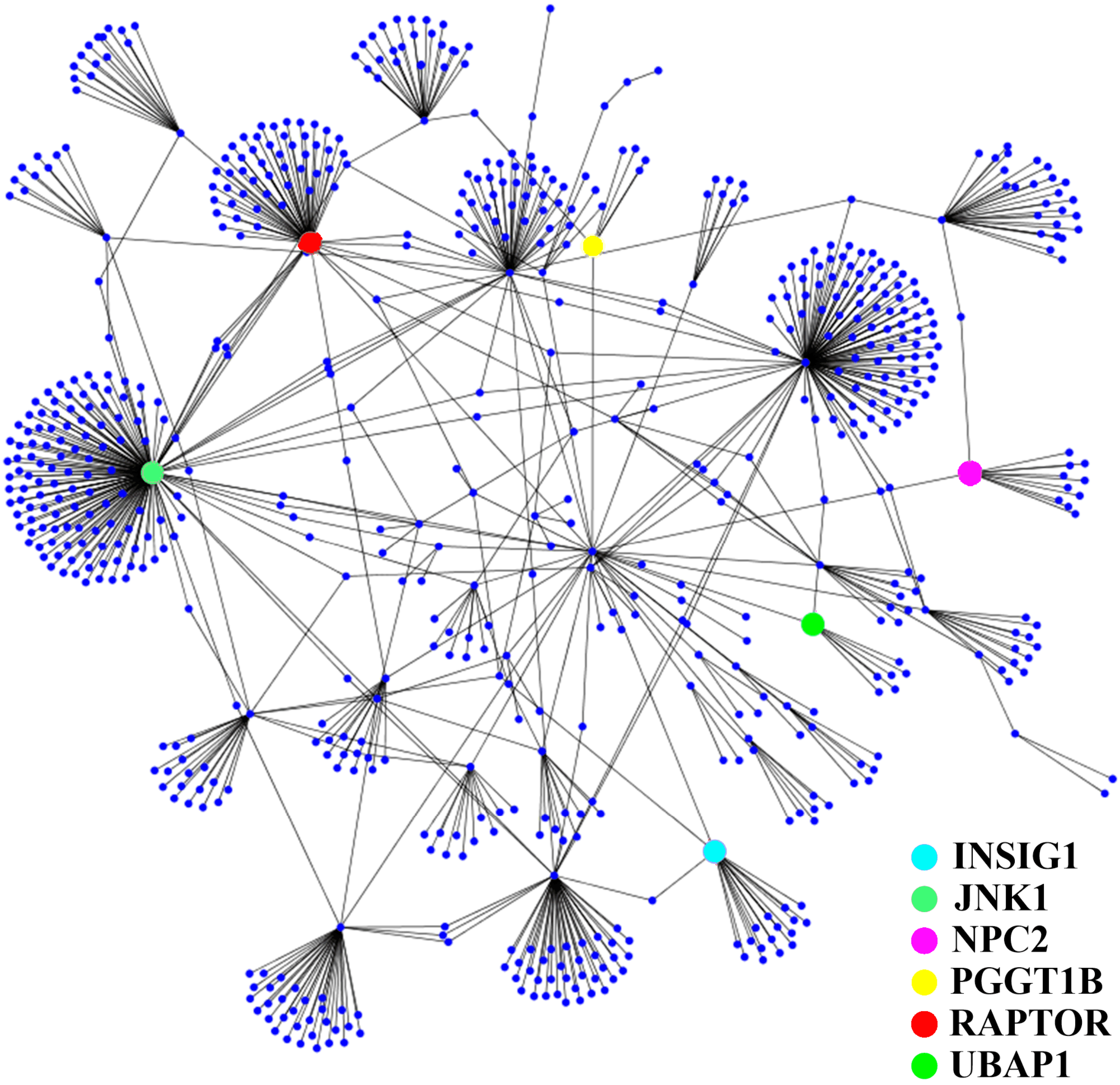

Figure S2. Protein-protein interaction network created by NetworkAnalyst. The protein-protein interaction network reveals several hub genes regulate interleukin-1β (IL-1β) (Table 1). Hub genes regulating IL-1β are highlighted with distinct hues.

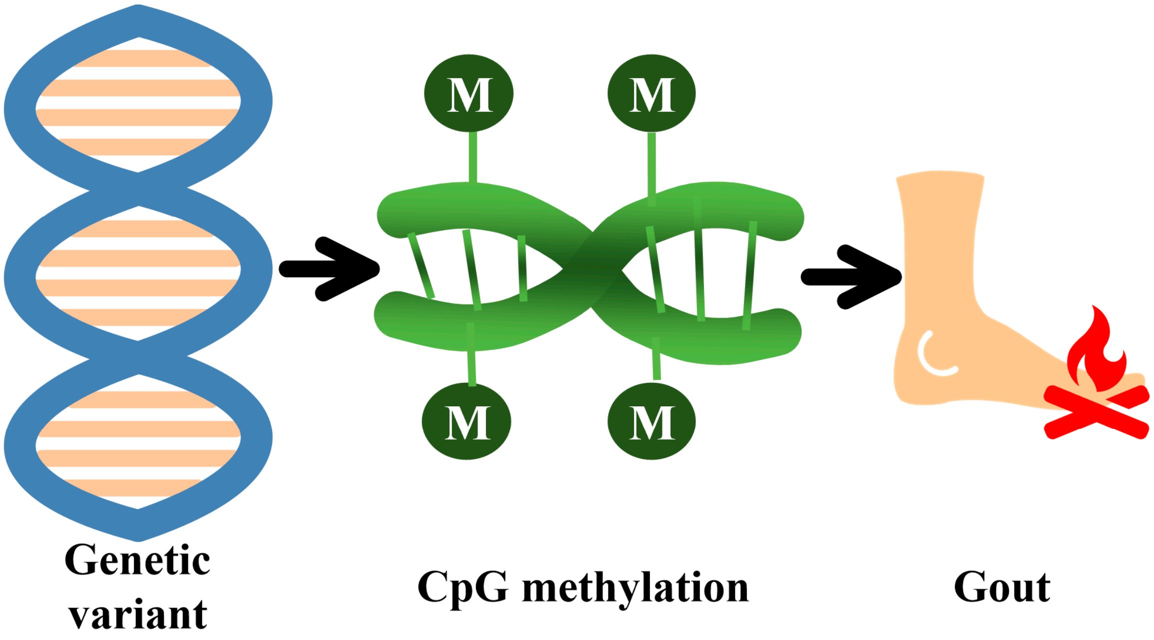

Figure S3. Identification of methylation mediating associations between genetic variant and gout. The schematic representation shows a causality model where association between genetic variant and gout is caused by methylation-mediated relationship, in which genmetic variant influences CpG methylation level that in turn affects risk of gout. The causal inference test attempts to identify such causal network.

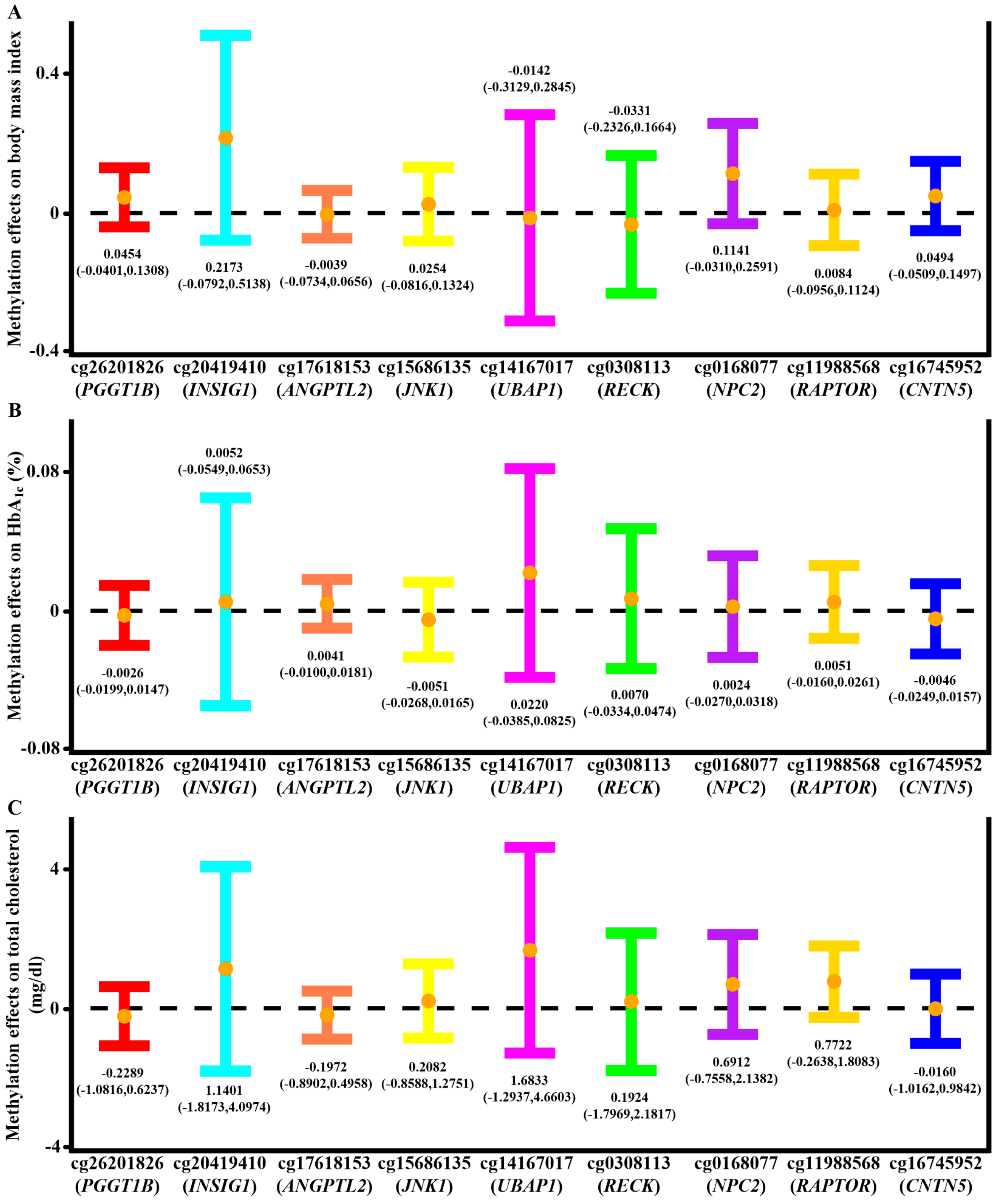

Figure S4. Associations of *PGGT1B*, *INSIG1*, *ANGPTL2*, *JNK1*, *UBAP1*, *RECK*, *NPC2*, *RAPTOR*, and *CNTN5* methylation with metabolic traits. Forest plots of associations between cg26201826 (*PGGT1B*), cg20419410 (*INSIG1*), cg17618153 (*ANGPTL2*), cg15686135 (*JNK1*), cg14167017 (*UBAP1*), cg0308113 (*RECK*), cg0168077 (*NPC2*), cg11988568 (*RAPTOR*), and cg16745952 (*CNTN5*) methylation and (A) body mass index, (B) HbA_{1c} levels, and (C) total cholesterol levels. Circles represent effect size estimates, with error bars representing respective 95% confidence interval. The effect size estimates and corresponding 95% confidence intervals are also listed. The associations between CpG methylation and body mass index, HbA_{1c} levels, and total cholesterol levels are calculated with multiple regression, correcting for sex, age, smoking history (total pack-years), smoking status, alcohol consumption, and blood cell subsets.

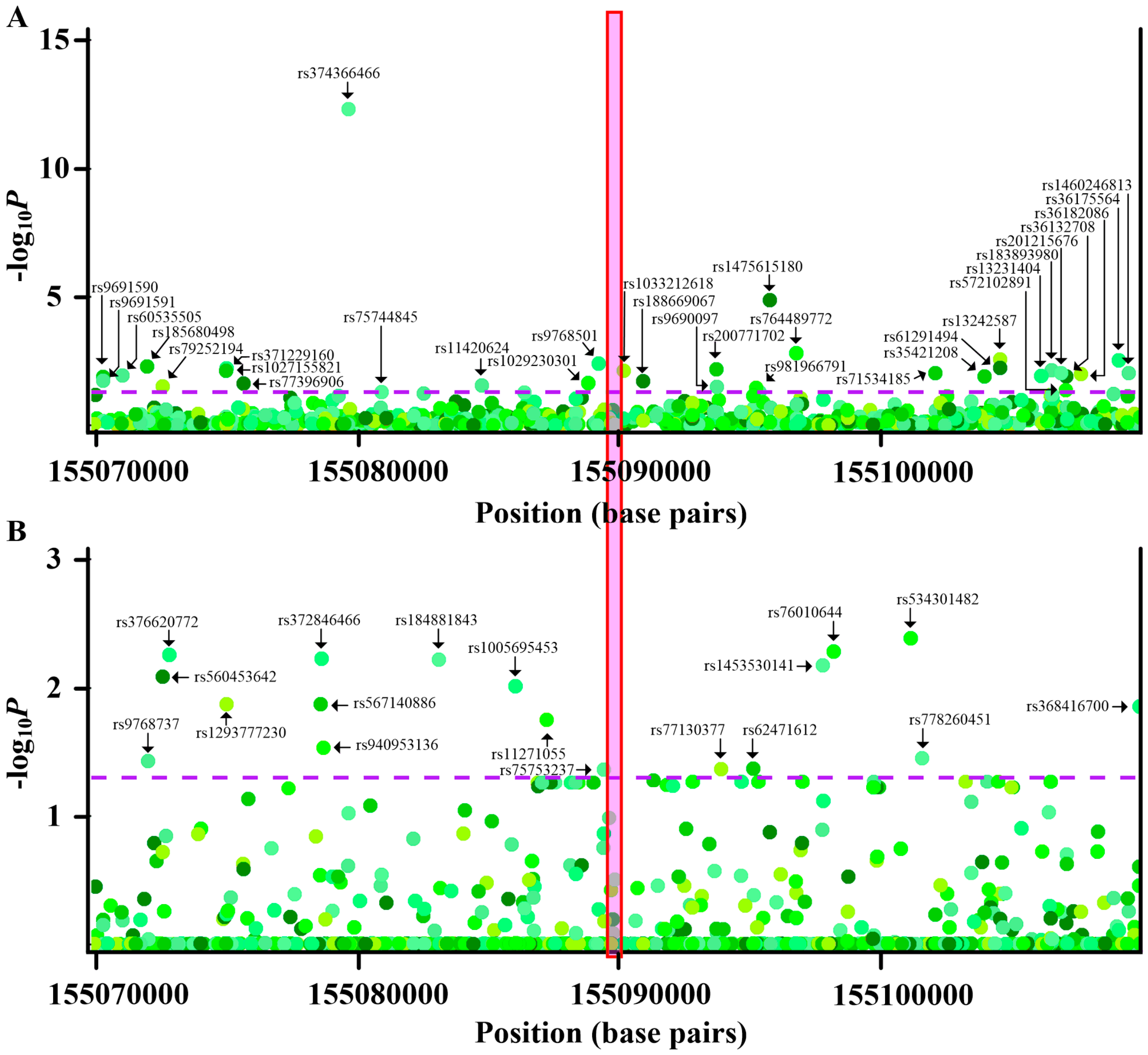

Figure S5. Genetic and methyl-quantitative trait locus (meQTL) analysis of cg20419410 (*INSIG1*). (A) Regional association plots of nearby variants with methylation levels of cg20419410. X-axis represents positions on the respective chromosome. Y axis represents minus $\log_{10}P$ of associations between variants and cg20419410 methylation. The variants with *P* values less than threshold are labeled with corresponding rs number. The associations between variants and CpG methylation are calculated with multiple regression, correcting for sex, age, smoking history (total pack-years), smoking status, alcohol consumption, and blood cell subsets. (B) Regional association plots of nearby variants with gout. X-axis represents positions on the respective chromosome. Y axis represents minus $\log_{10}P$ of associations between variants and gout. The variants with *P* values less than threshold are labeled with corresponding rs number. Every point is one variant colored with a respective hue, with different colors implying different variants. The dashed purple lines indicate the significance threshold ($P = 0.05$), and the red box highlights the location of cg20419410. The associations between variants and gout are calculated with multiple regression, correcting for sex, age, smoking history (total pack-years), smoking status, alcohol consumption, and blood cell subsets.

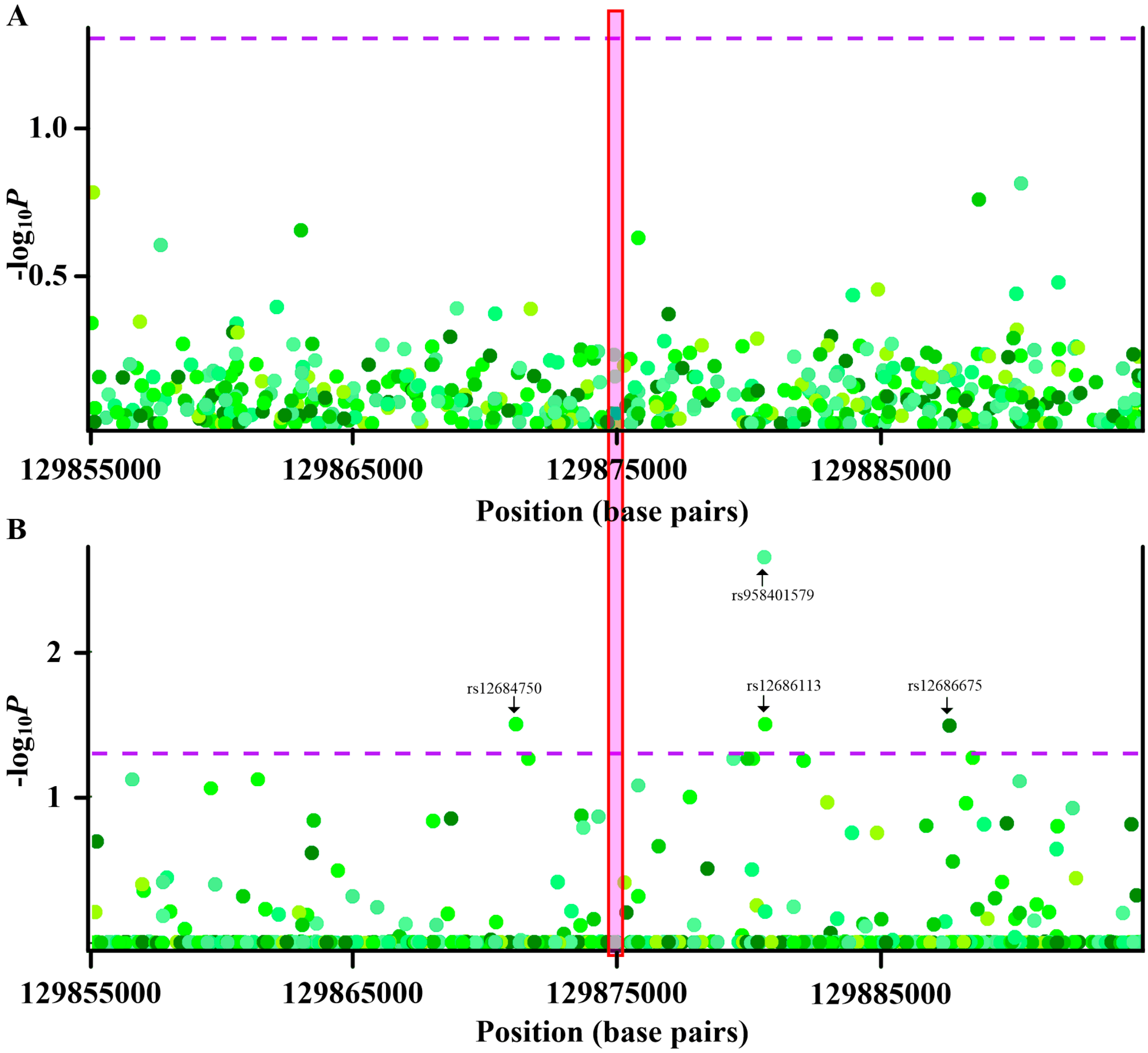

Figure S6. Genetic and methyl-quantitative trait locus (meQTL) analysis of cg17618153 (*ANGPTL2*). (A) Regional association plots of nearby variants with methylation levels of cg17618153. X-axis represents positions on the respective chromosome. Y axis represents minus $\log_{10}P$ of associations between variants and cg17618153 methylation. The associations between variants and CpG methylation are calculated with multiple regression, correcting for sex, age, smoking history (total pack-years), smoking status, alcohol consumption, and blood cell subsets. (B) Regional association plots of nearby variants with gout. X-axis represents positions on the respective chromosome. Y axis represents minus $\log_{10}P$ of associations between variants and gout. The variants with *P* values less than threshold are labeled with corresponding rs number. Every point is one variant colored with a respective hue, with different colors implying different variants. The dashed purple lines indicate the significance threshold ($P = 0.05$), and the red box highlights the location of cg17618153. The associations between variants and gout are calculated with multiple regression, correcting for sex, age, smoking history (total pack-years), smoking status, alcohol consumption, and blood cell subsets.

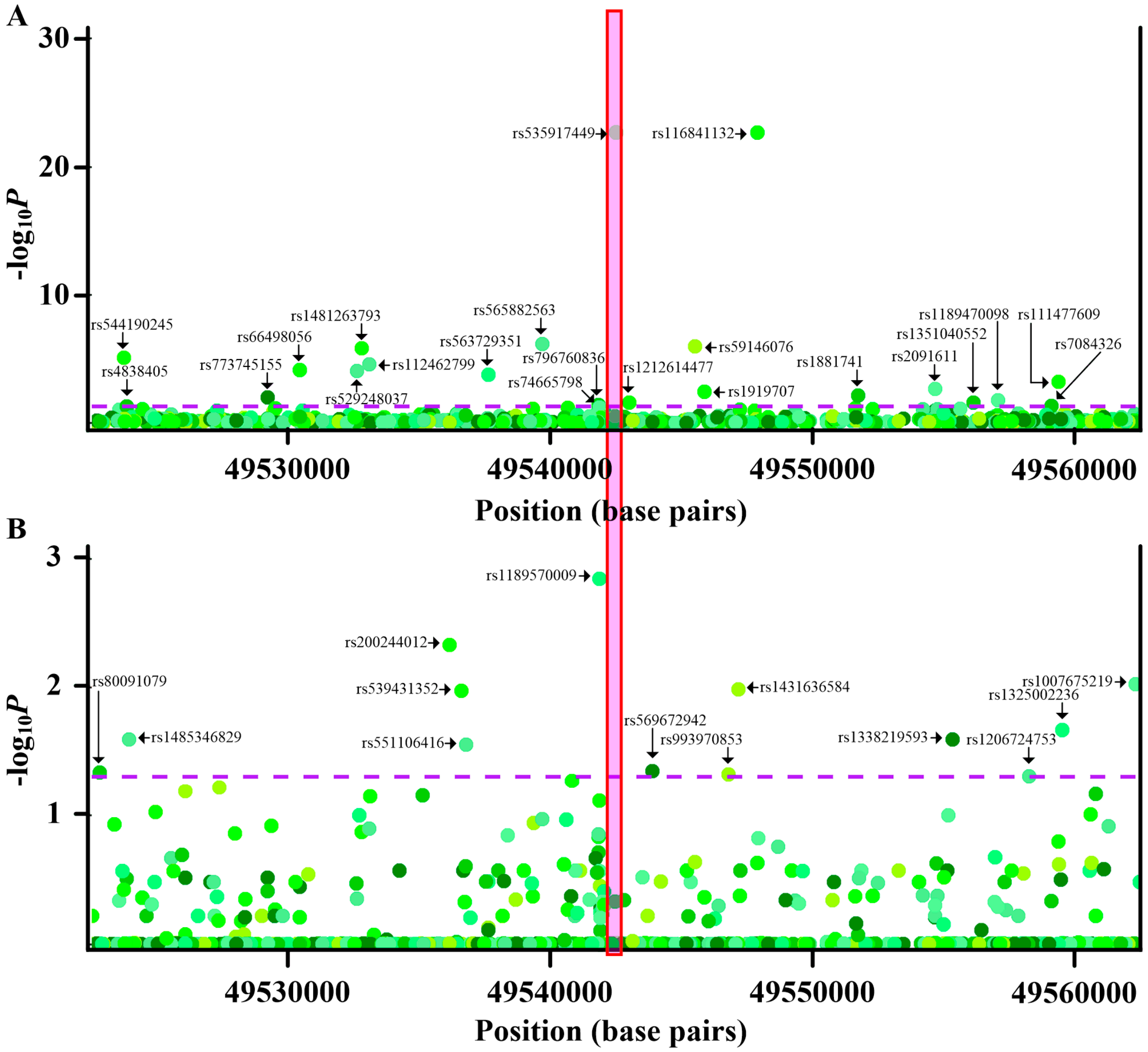

Figure S7. Genetic and methyl-quantitative trait locus (meQTL) analysis of cg15686135 (*JNK1*). (A) Regional association plots of nearby variants with methylation levels of cg15686135. X-axis represents positions on the respective chromosome. Y axis represents minus $\log_{10}P$ of associations between variants and cg15686135 methylation. The variants with *P* values less than threshold are labeled with corresponding rs number. The associations between variants and CpG methylation are calculated with multiple regression, correcting for sex, age, smoking history (total pack-years), smoking status, alcohol consumption, and blood cell subsets. (B) Regional association plots of nearby variants with gout. X-axis represents positions on the respective chromosome. Y axis represents minus $\log_{10}P$ of associations between variants and gout. The variants with *P* values less than threshold are labeled with corresponding rs number. Every point is one variant colored with a respective hue, with different colors implying different variants. The dashed purple lines indicate the significance threshold ($P = 0.05$), and the red box highlights the location of cg15686135. The associations between variants and gout are calculated with multiple regression, correcting for sex, age, smoking history (total pack-years), smoking status, alcohol consumption, and blood cell subsets.

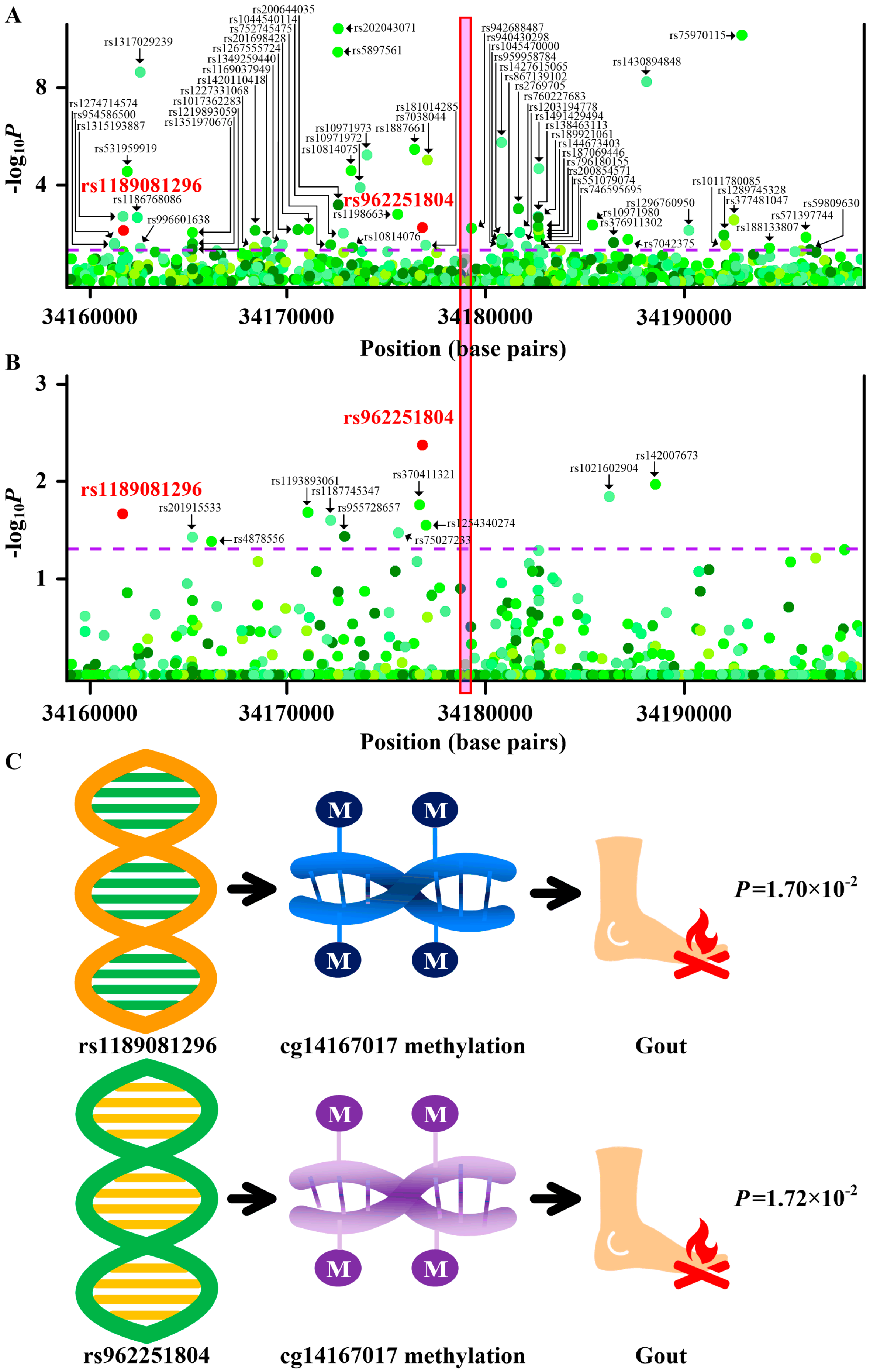
A
-log10P
8
4
rs1317029239
rs1274714574
rs954586500
rs1315193887
rs531959919
rs1189081296
rs1186768086
rs996601638
rs200644035
rs1044540114
rs752745475
rs201698428
rs1267555724
rs1349259440
rs1169037949
rs1420110418
rs1227331068
rs1017362283
rs1219893059
rs1351970676
rs202043071
rs5897561
rs10971973
rs10971972
rs10814075
rs1887661
rs181014285
rs7038044
rs962251804
rs1198663
rs10814076
rs942688487
rs940430298
rs1045470000
rs959958784
rs1427615065
rs867139102
rs2769705
rs760227683
rs1203194778
rs1491429494
rs138463113
rs189921061
rs144673403
rs187069446
rs796180155
rs200854571
rs551079074
rs746595695
rs75970115
rs1430894848
rs1011780085
rs1289745328
rs377481047
rs1296760950
rs10971980
rs376911302
rs7042375
rs59809630
rs571397744
rs188133807
34160000
34170000
34180000
34190000
Position (base pairs)
B
-log10P
3
2
1
rs962251804
rs1189081296
rs201915533
rs4878556
rs1193893061
rs1187745347
rs955728657
rs370411321
rs1254340274
rs75027233
rs1021602904
rs142007673
34160000
34170000
34180000
34190000
Position (base pairs)
C
M
rs1189081296
cg14167017 methylation
Gout
P=1.70×10-2
rs962251804
cg14167017 methylation
Gout
P=1.72×10-2

Figure S8. Genetic and methyl-quantitative trait locus (meQTL) analysis and causal inference test of cg14167017 (*UBAP1*). (A) Regional association plots of nearby variants with methylation levels of cg14167017. X-axis represents positions on the respective chromosome. Y axis represents minus $\log_{10}P$ of associations between variants and cg14167017 methylation. The variants with *P* values less than threshold are labeled with corresponding rs number. The associations between variants and CpG methylation are calculated with multiple regression, correcting for sex, age, smoking history (total pack-years), smoking status, alcohol consumption, and blood cell subsets. (B) Regional association plots of nearby variants with gout. X-axis represents positions on the respective chromosome. Y axis represents minus $\log_{10}P$ of associations between variants and gout. The variants with *P* values less than threshold are labeled with corresponding rs number. Every point is one variant colored with a respective hue, with different colors implying different variants. The dashed purple lines indicate the significance threshold ($P = 0.05$), and the red box highlights the location of cg14167017. Variants of concomitant associations with cg14167017 methylation and gout (rs1189081296, rs962251804) are marked with red color. The associations between variants and gout are calculated with multiple regression, correcting for sex, age, smoking history (total pack-years), smoking status, alcohol consumption, and blood cell subsets. (C) For variants of concomitant associations with cg14167017 methylation and gout (rs1189081296, rs962251804), causal inference test indicates cg14167017 methylation mediates relationship between variants (rs1189081296, rs962251804) and gout.

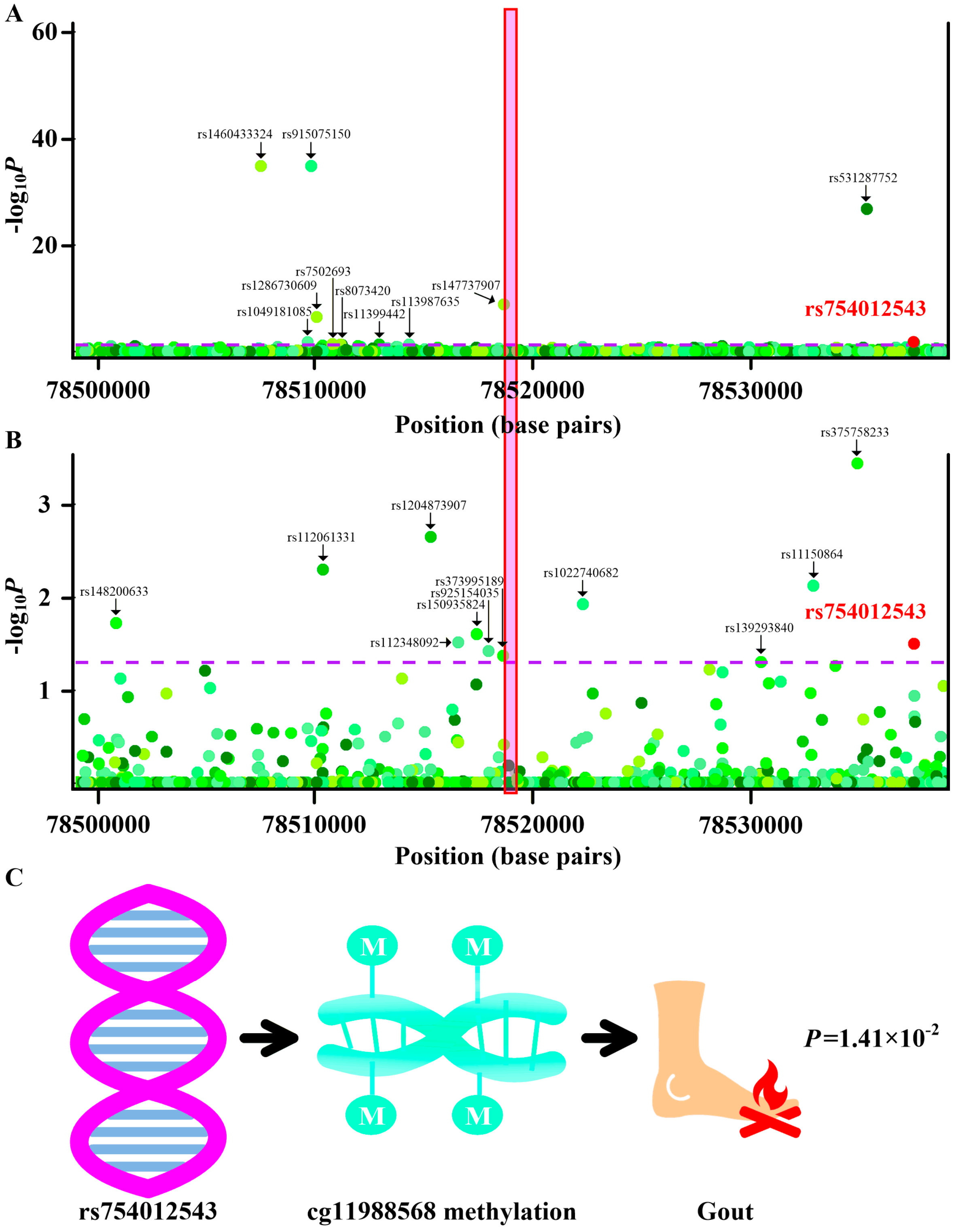

Figure S9. Genetic and methyl-quantitative trait locus (meQTL) analysis and causal inference test of cg11988568 (*RAPTOR*). (A) Regional association plots of nearby variants with methylation levels of cg11988568. X-axis represents positions on the respective chromosome. Y axis represents minus $\log_{10}P$ of associations between variants and cg11988568 methylation. The variants with *P* values less than threshold are labeled with corresponding rs number. The associations between variants and CpG methylation are calculated with multiple regression, correcting for sex, age, smoking history (total pack-years), smoking status, alcohol consumption, and blood cell subsets. (B) Regional association plots of nearby variants with gout. X-axis represents positions on the respective chromosome. Y axis represents minus $\log_{10}P$ of associations between variants and gout. The variants with *P* values less than threshold are labeled with corresponding rs number. Every point is one variant colored with a

respective hue, with different colors implying different variants. The dashed purple lines indicate the significance threshold (*P* = 0.05), and the red box highlights the location of cg11988568. The variant of concomitant associations with cg11988568 methylation and gout (rs754012543) is marked with red color. The associations between variants and gout are calculated with multiple regression, correcting for sex, age, smoking history (total pack-years), smoking status, alcohol consumption, and blood cell subsets. (C) For variant of concomitant associations with cg11988568 methylation and gout (rs754012543), causal inference test indicates cg11988568 methylation mediates relationship between variant (rs754012543) and gout.

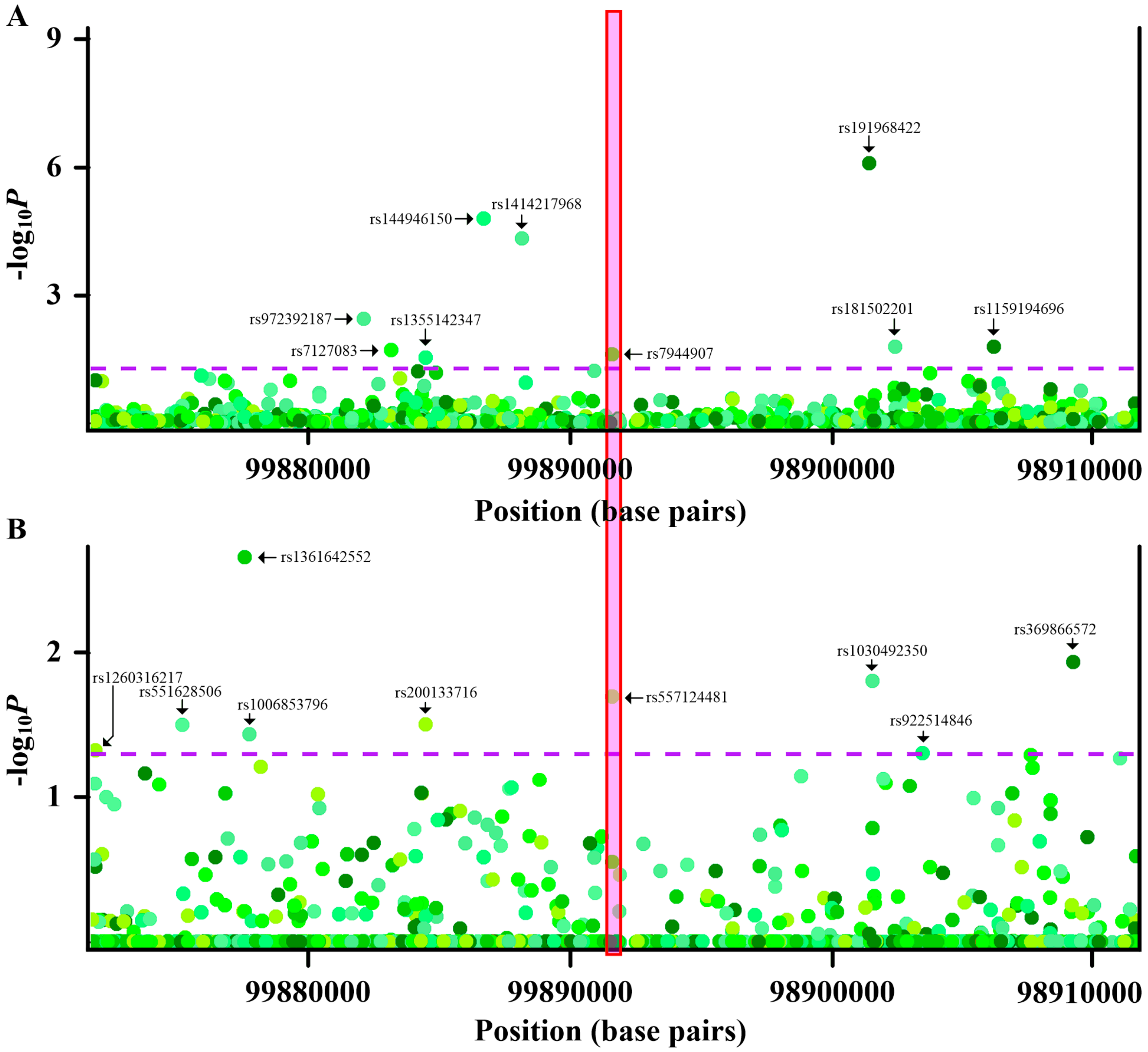

Figure S10. Genetic and methyl-quantitative trait locus (meQTL) analysis of cg16745952 (*CNTN5*). (A) Regional association plots of nearby variants with methylation levels of cg16745952. X-axis represents positions on the respective chromosome. Y axis represents minus $\log_{10}P$ of associations between variants and cg16745952 methylation. The variants with *P* values less than threshold are labeled with corresponding rs number. The associations between variants and CpG methylation are calculated with multiple regression, correcting for sex, age, smoking history (total pack-years), smoking status, alcohol consumption, and blood cell subsets. (B) Regional association plots of nearby variants with gout. X-axis represents positions on the respective chromosome. Y axis represents minus $\log_{10}P$ of associations between variants and gout. The variants with *P* values less than threshold are labeled with corresponding rs number. Every point is one variant colored with a respective hue, with different colors implying different variants. The dashed purple lines indicate the significance threshold (*P* = 0.05), and the red box highlights the location of cg16745952. The associations between variants and gout are calculated with multiple regression, correcting for sex, age, smoking history (total pack-years), smoking status, alcohol consumption, and blood cell subsets.

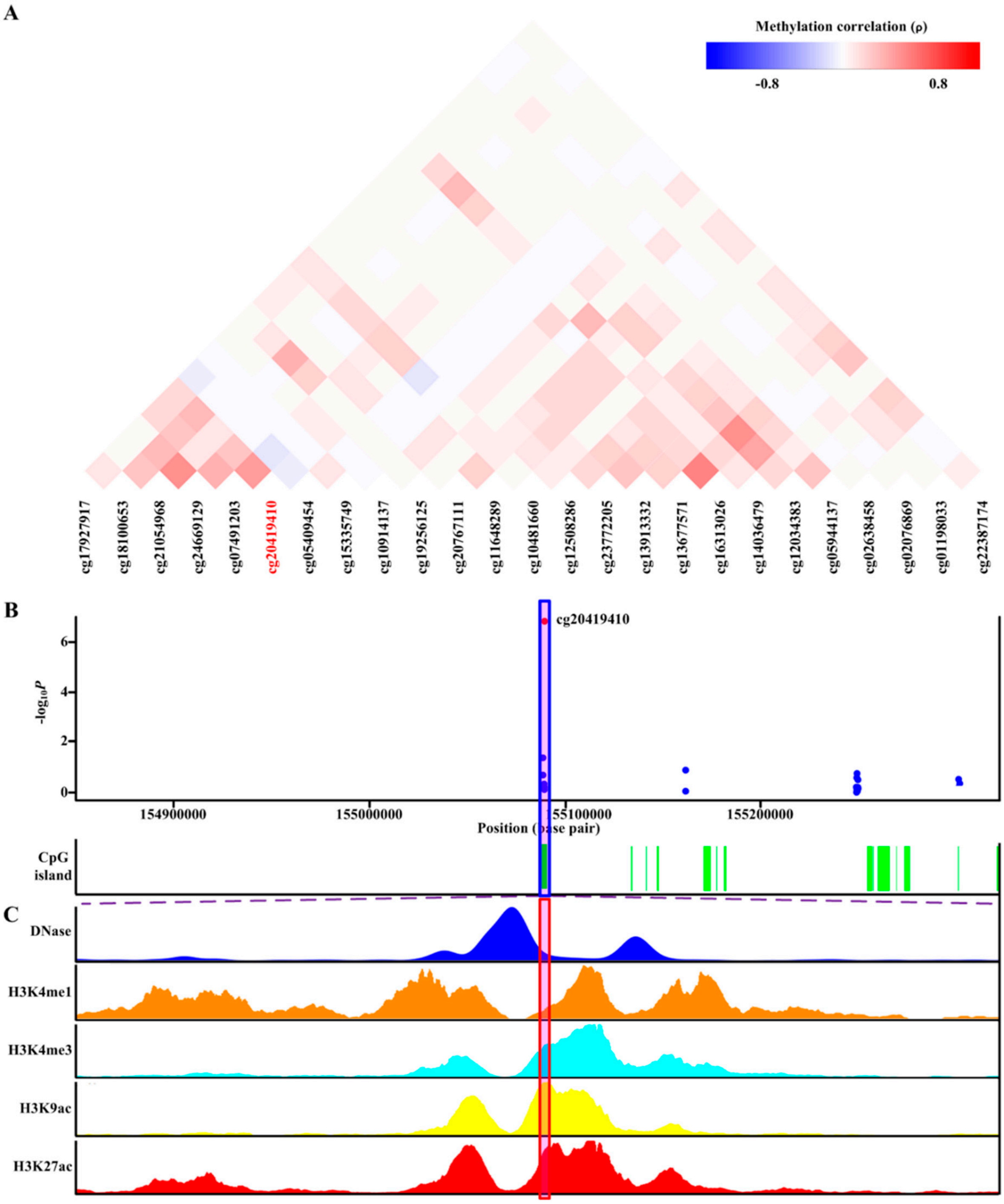

Figure S11. Co-methylation analysis and functional localization of cg20419410 (*INSIG1*). (A) Patterns of co-methylation at the CpG sites surrounding cg20419410. (B) Regional association results along with position of nearby CpG islands (green). cg20419410 (highlighted in shaded box) is located in CpG islands. (C) Functional annotation of cg20419410. DNase hypersensitive sites derived by DNase-seq (DNase Track) and histone marks surrounding cg20419410 (H3K4me1, H3K4me3, H3K9ac, and H3K27ac Tracks) in monocytes are shown. DNase hypersensitivity, H3K4me1, H3K4me3, H3K9ac, and H3K27ac histone marks are associated with active regulatory elements.

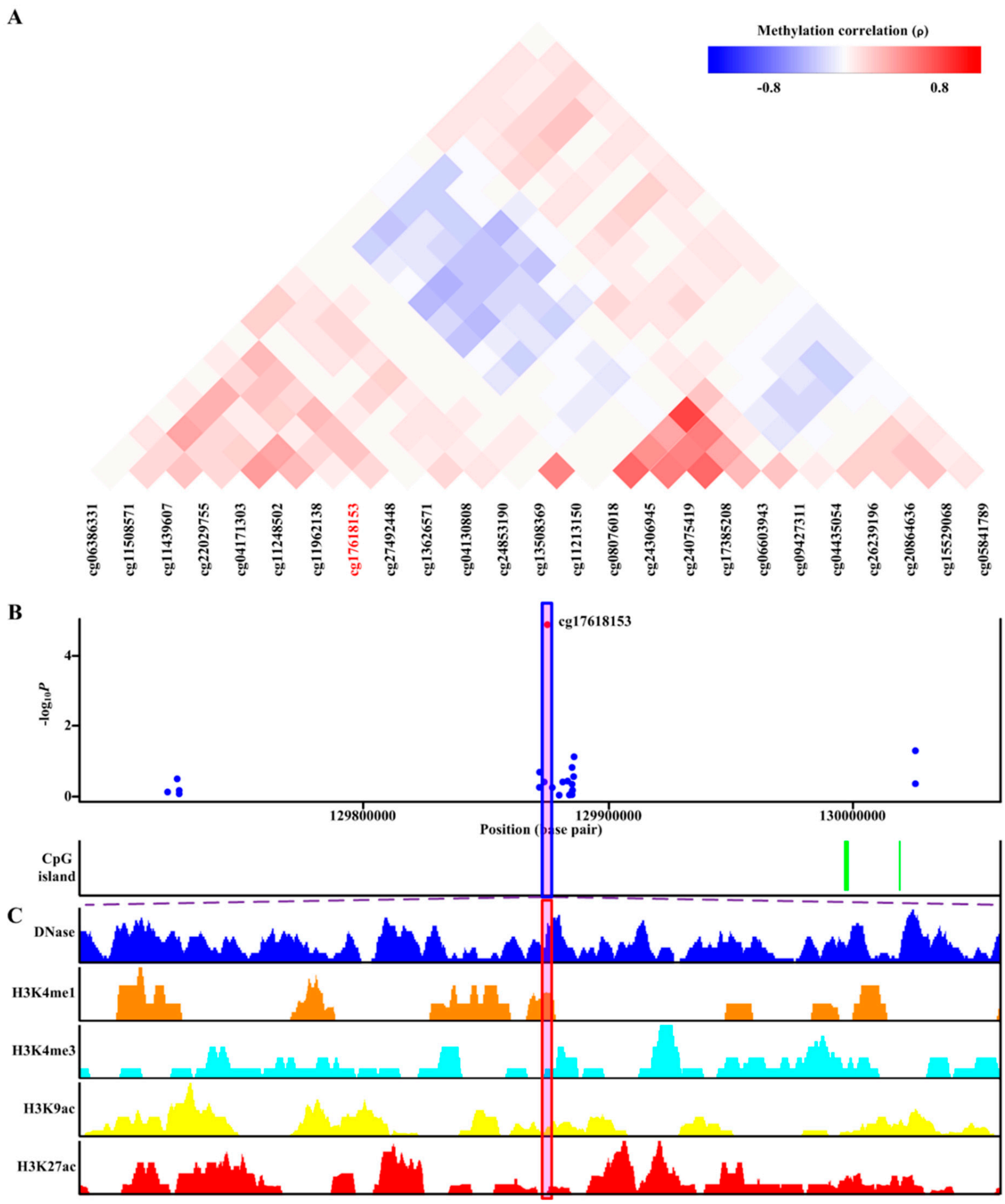

Figure S12. Co-methylation analysis and functional localization of cg17618153 (*ANGPTL2*). (A) Patterns of co-methylation at the CpG sites surrounding cg17618153. (B) Regional association results along with position of nearby CpG islands (green). (C) Functional annotation of cg17618153. DNase hypersensitive sites derived by DNase-seq (DNase Track) and histone marks surrounding cg17618153 (H3K4me1, H3K4me3, H3K9ac, and H3K27ac Tracks) in monocytes are shown. DNase hypersensitivity, H3K4me1, H3K4me3, H3K9ac, and H3K27ac histone marks are associated with active regulatory elements.

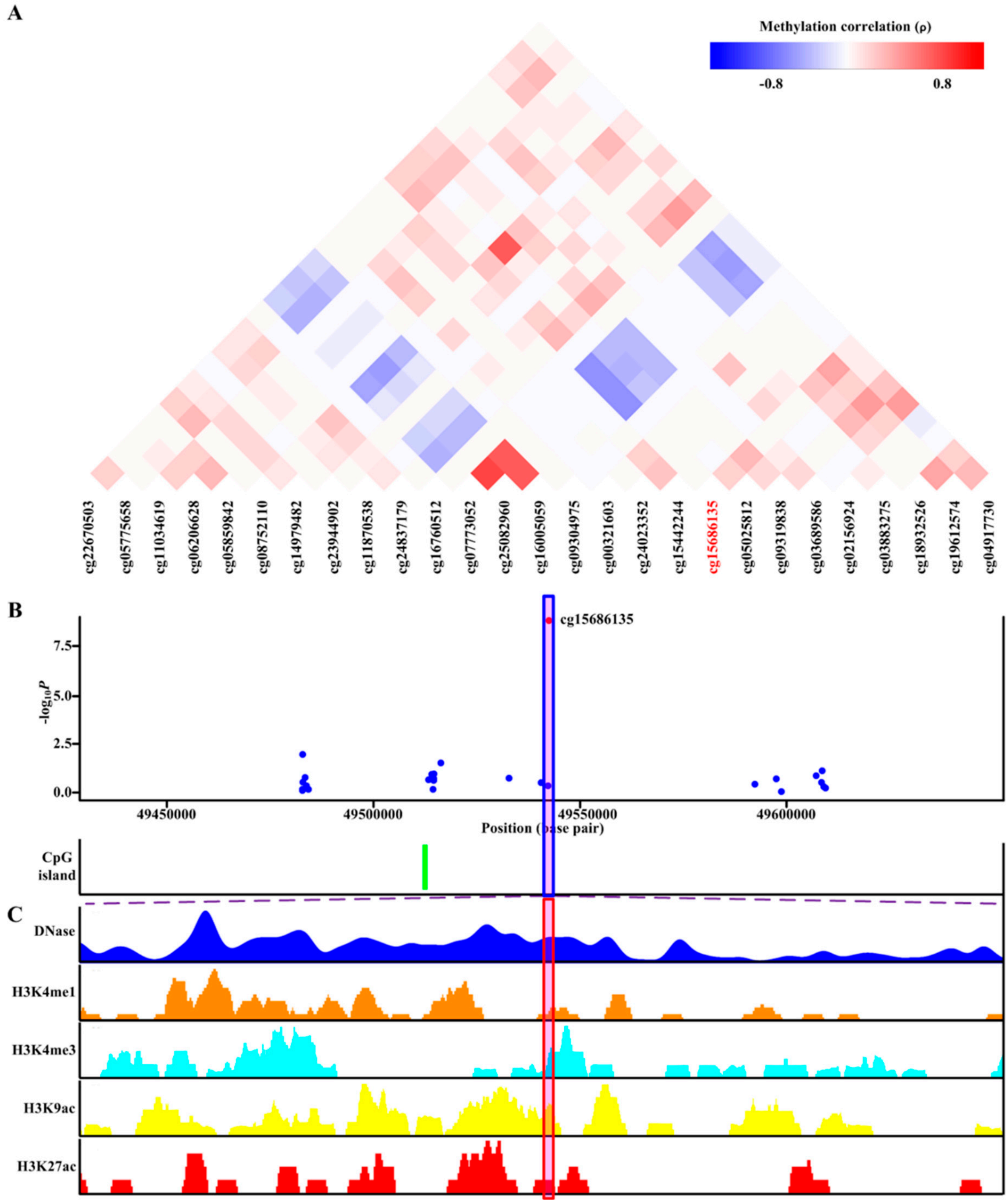

Figure S13. Co-methylation analysis and functional localization of cg15686135 (*JNK1*). (A) Patterns of co-methylation at the CpG sites surrounding cg15686135. (B) Regional association results along with position of nearby CpG islands (green). (C) Functional annotation of cg15686135. DNase hypersensitive sites derived by DNase-seq (DNase Track) and histone marks surrounding cg15686135 (H3K4me1, H3K4me3, H3K9ac, and H3K27ac Tracks) in monocytes are shown. DNase hypersensitivity, H3K4me1, H3K4me3, H3K9ac, and H3K27ac histone marks are associated with active regulatory elements.

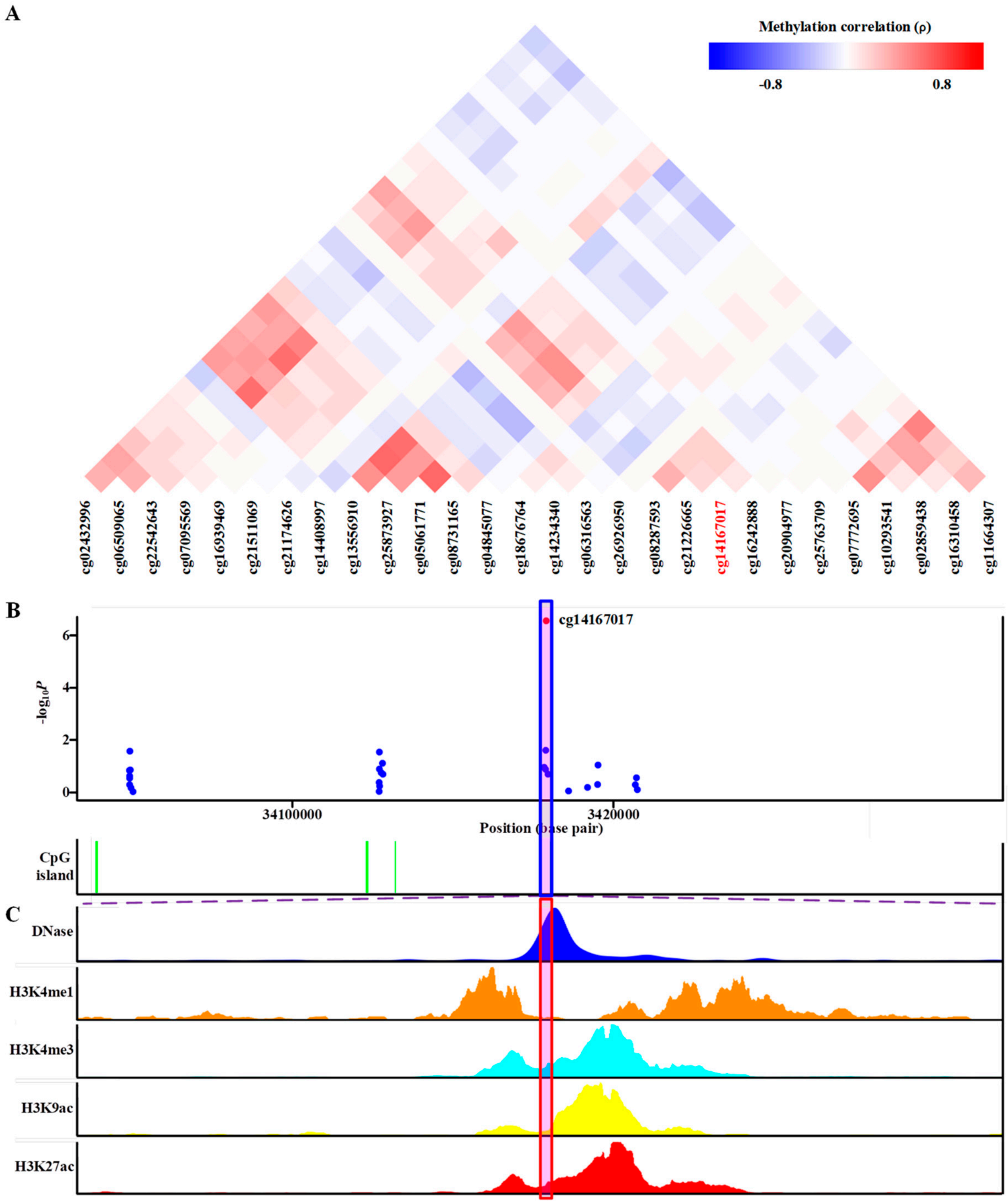

Figure S14. Co-methylation analysis and functional localization of cg14167017 (*UBAP1*). (A) Patterns of co-methylation at the CpG sites surrounding cg14167017. (B) Regional association results along with position of nearby CpG islands (green). (C) Functional annotation of cg14167017. DNase hypersensitive sites derived by DNase-seq (DNase Track) and histone marks surrounding cg14167017 (H3K4me1, H3K4me3, H3K9ac, and H3K27ac Tracks) in monocytes are shown. DNase hypersensitivity, H3K4me1, H3K4me3, H3K9ac, and H3K27ac histone marks are associated with active regulatory elements.

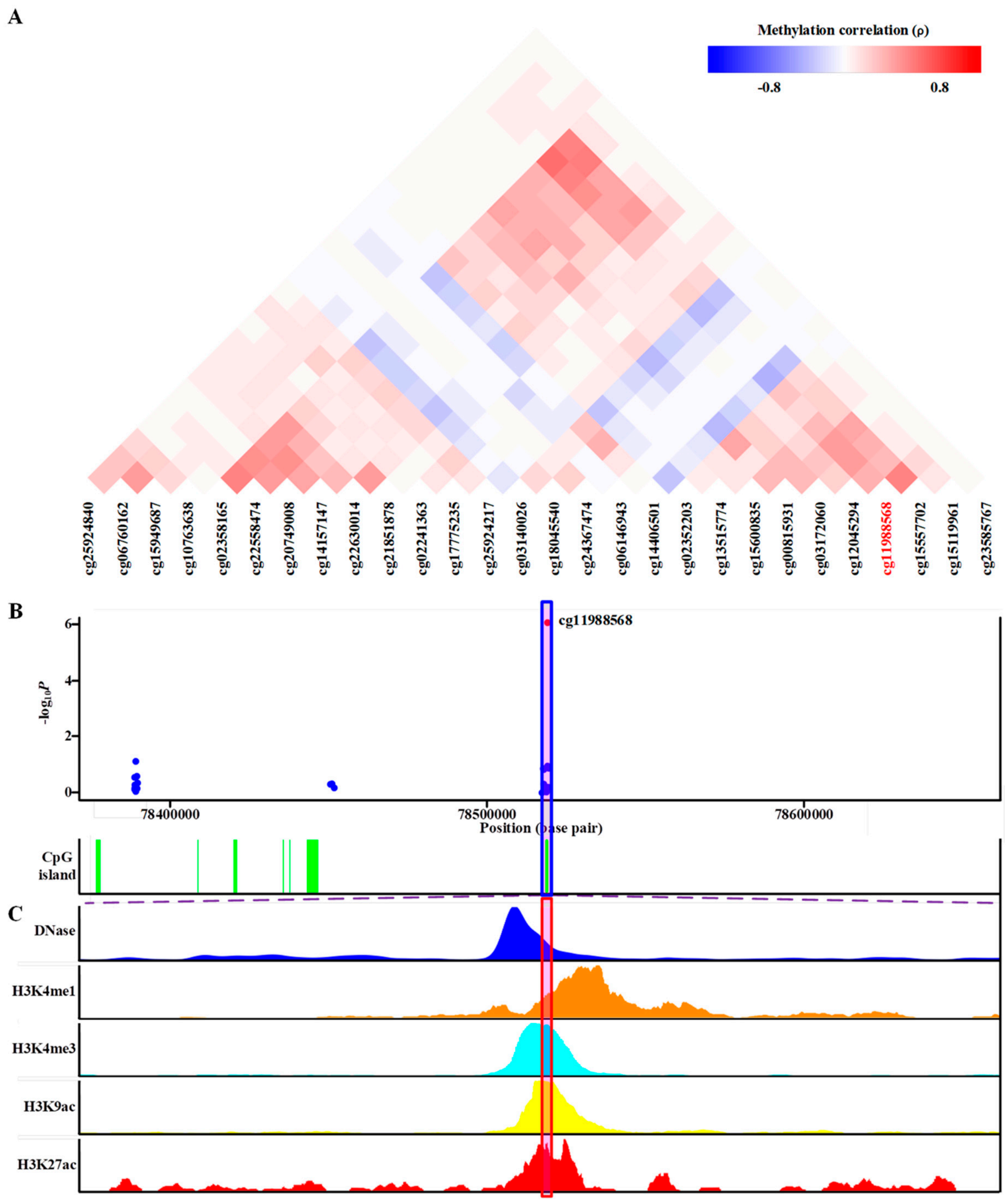

Figure S15. Co-methylation analysis and functional localization of cg11988568 (*RAPTOR*). (A) Patterns of co-methylation at the CpG sites surrounding cg11988568. (B) Regional association results along with position of nearby CpG islands (green). cg11988568 (highlighted in shaded box) is located in CpG islands. (C) Functional annotation of cg11988568. DNase hypersensitive sites derived by DNase-seq (DNase Track) and histone marks surrounding cg11988568 (H3K4me1, H3K4me3, H3K9ac, and H3K27ac Tracks) in monocytes are shown. DNase hypersensitivity, H3K4me1, H3K4me3, H3K9ac, and H3K27ac histone marks are associated with active regulatory elements.

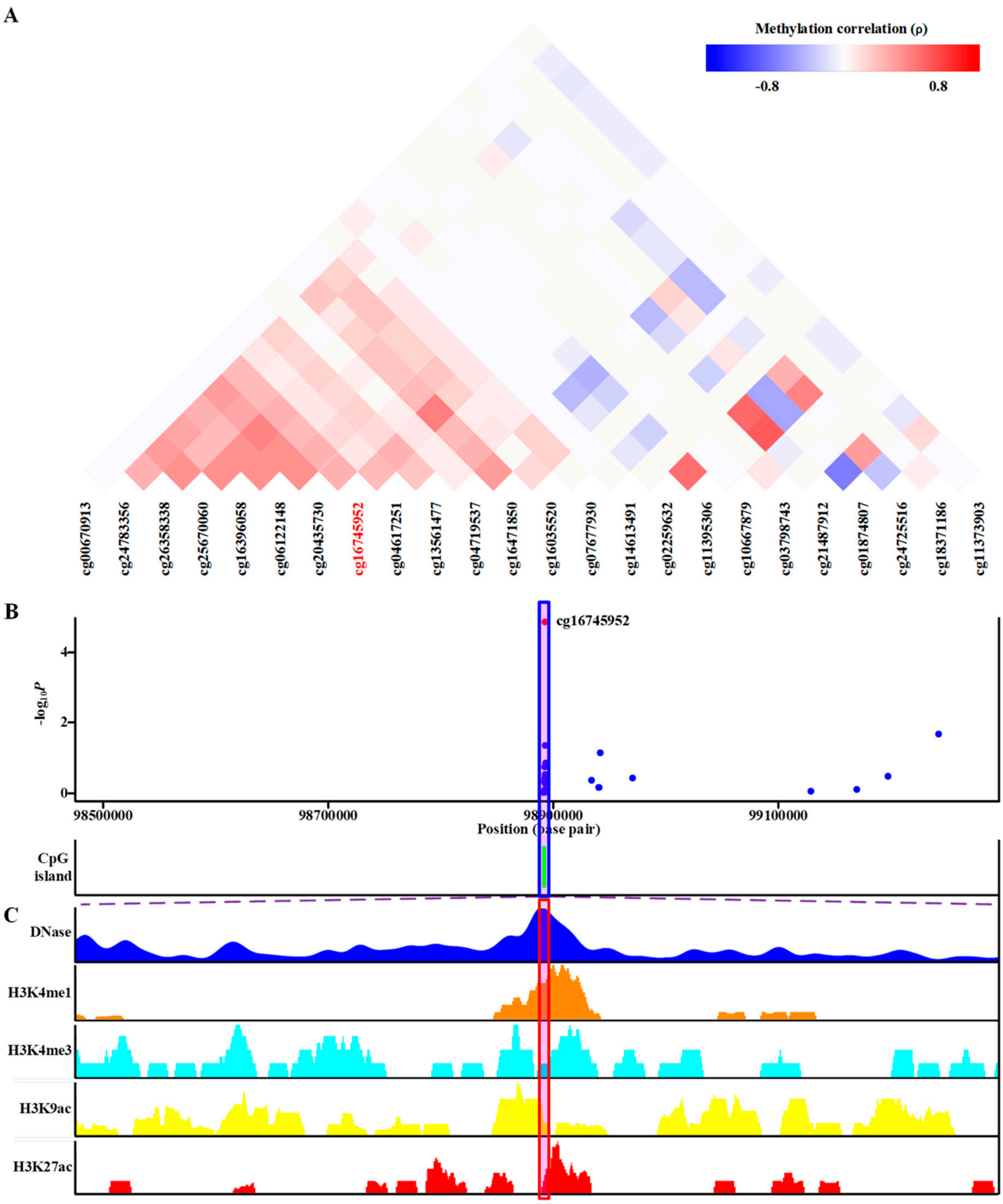

Figure S16. Co-methylation analysis and functional localization of cg16745952 (*CNTN5*). (A) Patterns of co-methylation at the CpG sites surrounding cg16745952. (B) Regional association results along with position of nearby CpG islands (green). cg16745952 (highlighted in shaded box) is located in CpG islands. (C) Functional annotation of cg16745952. DNase hypersensitive sites derived by DNase-seq (DNase Track) and histone marks surrounding cg16745952 (H3K4me1, H3K4me3, H3K9ac, and H3K27ac Tracks) in monocytes are shown. DNase hypersensitivity, H3K4me1, H3K4me3, H3K9ac, and H3K27ac histone marks are associated with active regulatory elements.

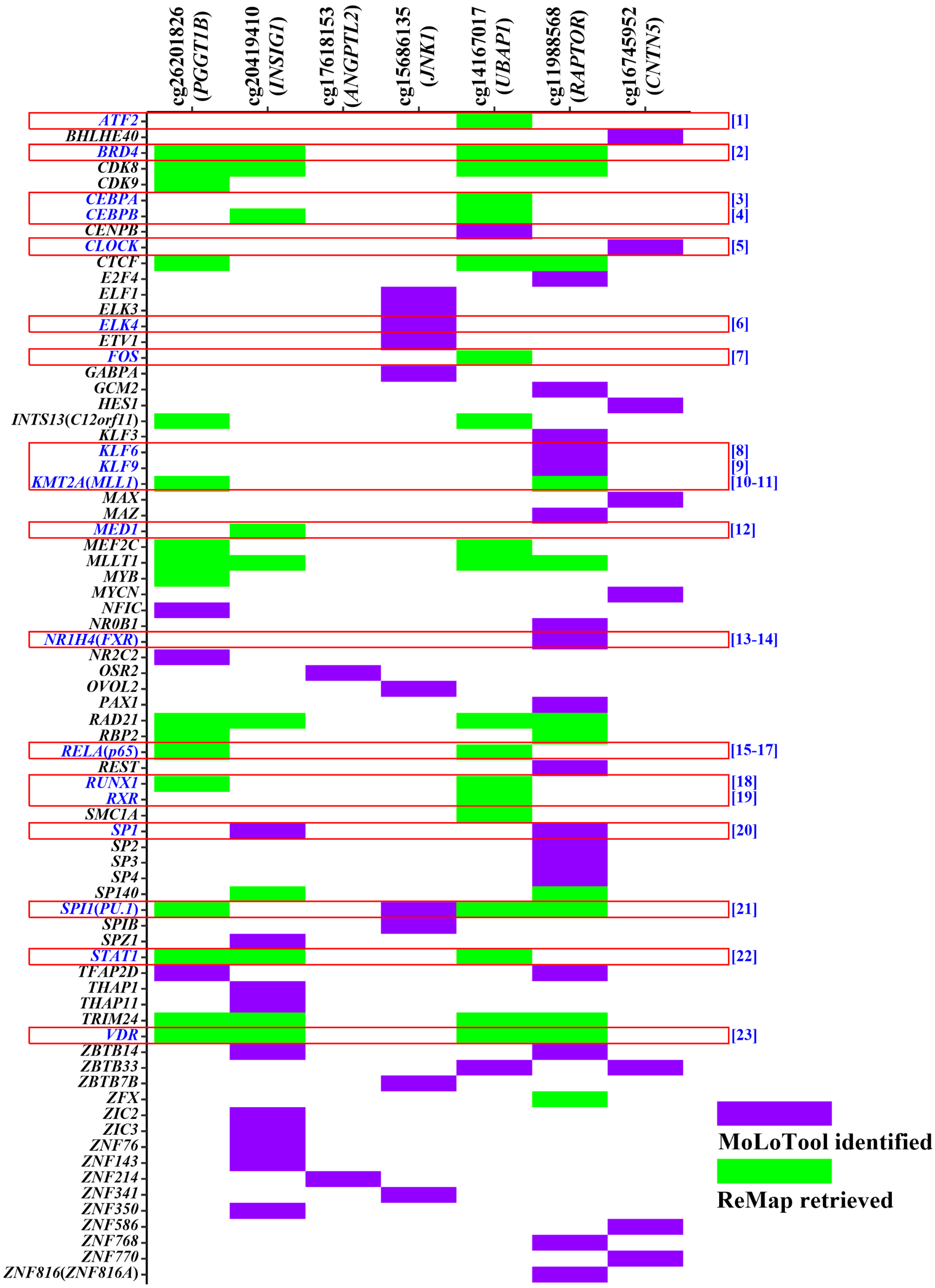

Figure S17. Potential transcription factors binding of cg26201826 (*PGGT1B*), cg20419410 (*INSIG1*), cg17618153 (*ANGPTL2*), cg15686135 (*JNK1*), cg14167017 (*UBAP1*), cg11988568 (*RAPTOR*), and cg16745952 (*CNTN5*) according to MoLoTool and ReMap. Potential binding of transcription factors to respective CpG sites according to MoLoTool are highlighted with purple while binding of transcription factors to respective CpG sites according to ReMap are highlighted with green. For example, *BHLHE40* binds cg16745952 (*CNTN5*) in MoLoTool (purple), *CDK9* binds cg26201826 (*PGGT1B*) according to ReMap (green). Some transcription factors (*ATF2*, *BRD4*, *CEBPA*, *CEBPB*,

CLOCK, *ELK4*, *FOS*, *KLF6*, *KLF9*, *KMT2A*, *MED1*, *NR1H4*(*FXR*), *RELA*(*p65*), *RUNX1*, *RXR*, *SP1*, *SPI1*, *STAT1*, and *VDR*) shown to regulate interleukin-1β (IL-1β) in the literature are highlighted (red box). Respective supporting articles [1–23] are listed in the following references.

References

1. Reimold, A.M.; Kim, J.; Finberg, R.; Glimcher, L.H. Decreased immediate inflammatory gene induction in activating transcription factor-2 mutant mice. *Int. Immunol.* **2001**, *13*, 241-248.
2. Guo, N.H.; Zheng, J.F.; Zi, F.M.; Cheng, J. I-BET151 suppresses osteoclast formation and inflammatory cytokines secretion by targetting BRD4 in multiple myeloma. *Biosci. Rep.* **2019**, *39*, BSR20181245.
3. Zhou, J.; Li, H.; Xia, X.; Herrera, A.; Pollock, N.; Reebye, V.; Sodergren, M.H.; Dorman, S.; Littman, B.H.; Doogan, D., et al. Anti-inflammatory Activity of MTL-CEBPA, a Small Activating RNA Drug, in LPS-Stimulated Monocytes and Humanized Mice. *Mol. Ther.* **2019**, *27*, 999-1016.
4. Gorgoni, B.; Maritano, D.; Marthyn, P.; Righi, M.; Poli, V. C/EBP beta gene inactivation causes both impaired and enhanced gene expression and inverse regulation of IL-12 p40 and p35 mRNAs in macrophages. *J. Immunol.* **2002**, *168*, 4055-4062.
5. Tang, X.; Guo, D.; Lin, C.; Shi, Z.; Qian, R.; Fu, W.; Liu, J.; Li, X.; Fan, L. hCLOCK induction by hypoxia promotes inflammatory responses by activating the NF-κB pathway. *Mol Med Rep* **2017**, *15*, 1401-1406.
6. Li, N.; Chen, J.; Zhao, J.; Wang, T. MicroRNA-3188 targets ETS-domain protein 4 and participates in RhoA/ROCK pathway to regulate the development of atherosclerosis. *Pharmazie.* **2017**, *72*, 687-693.
7. Hop, H.T.; Arayan, L.T.; Huy, T.X.N.; Reyes, A.W.B.; Vu, S.H.; Min, W.; Lee, H.J.; Rhee, M.H.; Chang, H.H.; Kim, S. The Key Role of c-Fos for Immune Regulation and Bacterial Dissemination in *Brucella* Infected Macrophage. *Front. Cell. Infect. Microbiol.* **2018**, *8*, 287.
8. Date, D.; Das, R.; Narla, G.; Simon, D.I.; Jain, M.K.; Mahabeleshwar, G.H. Kruppel-like transcription factor 6 regulates inflammatory macrophage polarization. *J. Biol. Chem.* **2014**, *289*, 10318-10329.
9. Ai, F.; Zhao, G.; Lv, W.; Liu, B.; Lin, J. Dexamethasone induces aberrant macrophage immune function and apoptosis. *Oncol. Rep.* **2020**, *43*, 427-436.
10. Yang, W.; Trahan, G.D.; Howell, E.D.; Speck, N.A.; Jones, K.L.; Gillen, A.E.; Riemondy, K.; Hesselberth, J.; Bryder, D.; Ernst, P. Enhancing Hematopoiesis from Murine Embryonic Stem Cells through MLL1-Induced Activation of a Rac/Rho/Integrin Signaling Axis. *Stem. Cell. Reports.* **2020**, *14*, 285-299.
11. Capell, B.C.; Drake, A.M.; Zhu, J.; Shah, P.P.; Dou, Z.; Dorsey, J.; Simola, D.F.; Donahue, G.; Sammons, M.; Rai, T.S., et al. MLL1 is essential for the senescence-associated secretory phenotype. *Genes. Dev.* **2016**, *30*, 321-336.
12. Liang, B.; Liu, E. Abstract 457: Transcription Coactivator MED1 Protects Against Atherosclerosis by Modulation of Macrophage Polarization. *Arterioscler. Thromb. Vasc. Biol.* **2016,** *36,* A457.
13. Kim, I.; Morimura, K.; Shah, Y.; Yang, Q.; Ward, J.M.; Gonzalez, F.J. Spontaneous hepatocarcinogenesis in farnesoid X receptor-null mice. *Carcinogenesis.* **2007**, *28*, 940-946.
14. Vavassori, P.; Mencarelli, A.; Renga, B.; Distrutti, E.; Fiorucci, S. The bile acid receptor FXR is a modulator of intestinal innate immunity. *J. Immunol.* **2009**, *183*, 6251-6261.
15. Weichert, W.; Boehm, M.; Gekeler, V.; Bahra, M.; Langrehr, J.; Neuhaus, P.; Denkert, C.; Imre, G.; Weller, C.; Hofmann, H.P., et al. High expression of RelA/p65 is associated with activation of nuclear factor-kappaB-dependent signaling in pancreatic cancer and marks a patient population with poor prognosis. *Br. J. Cancer.* **2007**, *97*, 523-530.
16. Qian, T.; Wang, K.; Cui, J.; He, Y.; Yang, Z. Angiopoietin-Like Protein 7 Promotes an Inflammatory Phenotype in RAW264.7 Macrophages Through the P38 MAPK Signaling Pathway. *Inflammation.* **2016**, *39*, 974-985.
17. Wang, M.; Kong, X.; Xie, Y.; He, C.; Wang, T.; Zhou, H. Role of TLR-4 in anti-β2-glycoprotein I-induced activation of peritoneal macrophages and vascular endothelial cells in mice. *Mol. Med. Rep.* **2019**, *19*, 4353-4363.
18. Luo, M.C.; Zhou, S.Y.; Feng, D.Y.; Xiao, J.; Li, W.Y.; Xu, C.D.; Wang, H.Y.; Zhou, T. Runt-related Transcription Factor 1 (RUNX1) Binds to p50 in Macrophages and Enhances TLR4-triggered Inflammation and Septic Shock. *J. Biol. Chem.* **2016**, *291*, 22011-22020.
19. Desreumaux, P.; Dubuquoy, L.; Nutten, S.; Peuchmaur, M.; Englaro, W.; Schoonjans, K.; Derijard, B.; Desvergne, B.; Wahli, W.; Chambon, P., et al. Attenuation of colon inflammation through activators of the retinoid X receptor (RXR)/peroxisome proliferator-activated receptor gamma (PPARgamma) heterodimer. A basis for new therapeutic strategies. *J. Exp. Med.* **2001**, *193*, 827-838.

20. Liu, L.; Li, Y.; Van Eldik, L.J.; Griffin, W.S.; Barger, S.W. S100B-induced microglial and neuronal IL-1 expression is mediated by cell type-specific transcription factors. *J. Neurochem.* **2005**, *92*, 546-553.
21. Liu, C.; Wang, M.; Sun, W.; Cai, F.; Geng, S.; Su, X.; Shi, Y. PU.1 serves a critical role in the innate defense against Aspergillus fumigatus via dendritic cell-associated C-type lectin receptor-1 and toll-like receptors-2 and 4 in THP-1-derived macrophages. *Mol. Med. Rep.* **2017**, *15*, 4084-4092.
22. Liu, Y.; Tang, H.; Liu, X.; Chen, H.; Feng, N.; Zhang, J.; Wang, C.; Qiu, M.; Yang, J.; Zhou, X. Frontline Science: Reprogramming COX-2, 5-LOX, and CYP4A-mediated arachidonic acid metabolism in macrophages by salidroside alleviates gouty arthritis. *J. Leukoc. Biol.* **2019**, *105*, 11-24.
23. Yang, Y.; Liu, X.; Yin, W.; Xie, D.; He, W.; Jiang, G.; Fan, J. 5-Aza-2'-deoxycytidine enhances the antimicrobial response of vitamin D receptor against Mycobacterium tuberculosis. *RSC. Adv.* **2016,** 6, 61740.

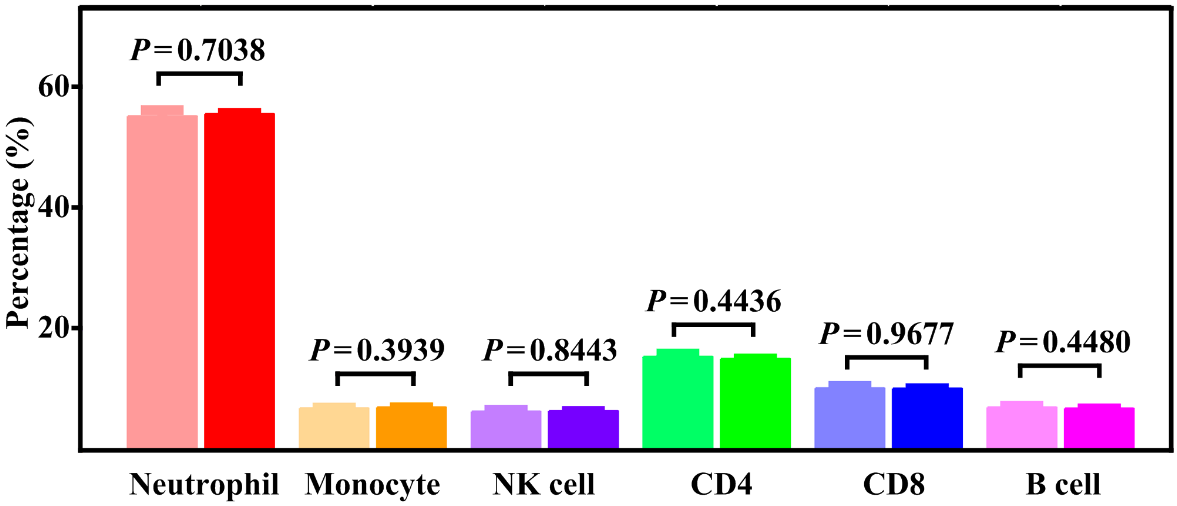

Figure S18. Cell subsets frequencies in gout and non-gout. The cell subsets percentages calculated with minfi in gout and non-gout. The percentages of cell subsets in gout are shown in light color, while the percentages of cell subsets in non-gout are shown in dark color.

A

GEO dataset	Probe ID	Comparison	P
GDS3203 – Liu et al	207452_s_at	Fresh monocyte and differentiated macrophage	4.31×10^{-4}

B

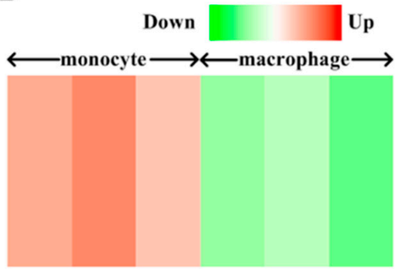

Figure S19. ***CNTN5*** **downregulated in differentiated macrophage.** (A) The details of source expression dataset for *CNTN5*. (B) Heatmap reveals the scaled expression of *CNTN5* in differentiated macrophages and monocytes obtained from GDS3203. Samples are shown in columns.
